# Structural and mechanistic insights into caseinolytic protease inhibition for antimicrobial development against *Pseudomonas plecoglossicida*

**DOI:** 10.1371/journal.ppat.1013909

**Published:** 2026-02-12

**Authors:** Jingjie Chen, Ping Zhang, Hongxin Guan, Bing Gong, Xiaoding Li, Zekai Li, Fan Li, Biao Zhou, Xuemin Chen, Xinhua Chen, Songying Ouyang, Yong-An Zhang

**Affiliations:** 1 National Key Laboratory of Agricultural Microbiology, Hubei Hongshan Laboratory, Engineering Research Center of Green Development for Conventional Aquatic Biological Industry in the Yangtze River Economic Belt, Ministry of Education, College of Fisheries, Huazhong Agricultural University, Wuhan, China; 2 Key Laboratory of Marine Biotechnology of Fujian Province, College of Marine Sciences, Fujian Agriculture and Forestry University, Fuzhou, China; 3 Key Laboratory of Microbial Pathogenesis and Interventions of Fujian Province University, College of Life Sciences, Fujian Normal University, Fuzhou, China; 4 Guangzhou Institutes of Biomedicine and Health Chinese Academy of Sciences, Guangzhou, China; 5 School of Life Sciences, Anhui University, Hefei, China; Amity University, INDIA

## Abstract

The caseinolytic protease (ClpP) is an emerging antibacterial target. *Pseudomonas plecoglossicida* (*Pp*), a pathogen causing visceral white spot disease in *Larimichthys crocea*, encodes two ClpP paralogs, *Pp*ClpP1 and *Pp*ClpP2. This study characterizes their distinct structural and functional properties. Phylogenetic and biochemical analysis revealed that *Pp*ClpP2 functions as a canonical serine protease with high peptidase activity, while *Pp*ClpP1 is evolutionarily divergent, exhibiting low inherent activity due to an unconventional Ser-His-Pro catalytic triad and a truncated N-terminal domain. Cryo-EM structure determination of *Pp*ClpP1 confirmed a homotetradecameric assembly with a dilated axial pore and a non-canonical catalytic geometry. In contrast, AlphaFold-predicted *Pp*ClpP2 displayed a compact structure with a canonical Ser-His-Asp triad. The subunits formed a stable heterotetradecamer (*Pp*ClpP1P2) with enhanced proteolytic activity compared to individual homotetradecameric. Pull-down assays demonstrated that *Pp*ClpP2, but not *Pp*ClpP1, specifically interacts with the unfoldase *Pp*ClpX, and the *Pp*ClpP1P2 heterotetradecamer further augmented *Pp*ClpX-mediated degradation of model substrates. Notably, the proteasome inhibitor bortezomib (BTZ) selectively inhibited *Pp*ClpP1 by binding to a unique pocket near the active site without engaging the catalytic serine, thereby suppressing bacterial growth in a *Pp*ClpP1-dependent manner. This study elucidates the structural basis of functional divergence between *Pp*ClpP paralogs, highlights their synergistic interplay in proteolysis, and identifies *Pp*ClpP1 as a druggable target for antibacterial development.

## Author summary

*Pseudomonas plecoglossicida* is a bacterial pathogen that causes devastating visceral white spot disease in the economically important large yellow croaker (*Larimichthys crocea*). The bacterium encodes two variants of ClpP, a key protease that regulates protein turnover and bacterial fitness. Here, we show that these two variants, *Pp*ClpP1 and *Pp*ClpP2, have distinct structural and functional roles. While *Pp*ClpP2 acts as a typical, highly active protease, *Pp*ClpP1 has low intrinsic activity due to an unusual catalytic triad and a truncated structure. Strikingly, when combined, they form a hybrid complex with enhanced degradation capacity. We further find that only *Pp*ClpP2 interacts with the unfoldase *Pp*ClpX, which helps deliver substrates for breakdown. Importantly, the bortezomib selectively blocks *Pp*ClpP1—but not *Pp*ClpP2—by binding near its active site, and this inhibition suppresses bacterial growth. Our work reveals how these two protease variants cooperate in *P. plecoglossicida* and identifies *Pp*ClpP1 as a promising target for developing specific antibiotics against this aquaculture pathogen.

## 1. Introduction

*Pseudomonas plecoglossicida* (*P. plecoglossicida*, *Pp*), an aerobic Gram-negative bacterium, is the causative agent of visceral white spot disease in *Larimichthys crocea* [1]*.* The increasing prevalence of antimicrobial resistance in this pathogen highlights the urgent need for novel therapeutic approaches. Caseinolytic protease (Clp), a family of highly conserved multi-subunit enzymes found in both bacteria and eukaryotes, have recently emerged as attractive targets for antibacterial drug development [2,3]. Modulation of ClpP protease activity through either activation or inhibition can disrupt proteolytic homeostasis [4–6], effectively eliminating pathogenic bacteria, including biofilm embedded and persister cell populations [7].

The ClpP complex forms a tetradecameric barrel structure comprising two stacked heptameric rings that associate with hexameric AAA+ unfoldases to mediate substrate unfolding and translocation into the proteolytic chamber. As a serine protease, ClpP exhibits limited inherent activity, primarily cleaving short peptide substrates such as Suc-Leu-Tyr-AMC [8]. While most bacteria possess a single *clpP* gene, certain pathogens including *M. tuberculosis*, *L. monocytogenes*, and *P. aeruginosa* harbor two *clpP* paralogs [9–12]. These ClpP1/P2 systems exhibit remarkable structural diversity across species, forming either homotypic heptamers, inactive homotypic tetradecamers, active homotypic tetradecamers, or active heterotypic tetradecamers [13–16]. Notably, *P. plecoglossicida* encodes two ClpP paralogs (*clpP1* and *clpP2*) along with two unfoldases (*clpA* and *clpX*). Intriguingly, *clpP2* is co-localized with *clpX* in an operon, suggesting potential functional coupling between *Pp*ClpP2 and *Pp*ClpX, while *clpP1* resides at a distinct chromosomal locus separate from *clpA*. The oligomerization states, three-dimensional architecture, and functional dynamics of these *Pp*ClpP variants remain to be fully elucidated.

The catalytic triad (Ser-His-Asp/Asn) constitutes the essential proteolytic center of ClpP. In the tetradecameric assembly, these catalytic residues are sequestered within the barrel interior. Proteolytic activity requires alignment of the catalytic triad through conformational changes of the handle domain that forms the interface between heptamer rings. Inactive tetradecamers maintain misaligned catalytic residues, with proper orientation typically induced by AAA+ unfoldase (ClpX) binding at hydrophobic grooves formed between adjacent monomers [17]. This unfoldase interface represents an attractive target for antimicrobial development. For instance, acyldepsipeptides (ADEPs) and activators of cylindrical proteases (ACPs) compete with unfoldases for ClpP binding, often resulting in catalytic triad alignment and constitutive activation [18,19]. The proteasome inhibitor bortezomib binds directly to the active-site serine of *T. thermophilus* ClpP (*Tt*ClpP), mimicking a peptide substrate and inducing allosteric activation [20]. However, whether small molecule modulators can similarly dysregulate *Pp*ClpP function remains unknown.

Here, we characterized both *Pp*ClpP subunits from *P. plecoglossicida*. Biochemical analyses demonstrated that *Pp*ClpP1 possesses relatively low peptidase activity, while *Pp*ClpP2 exhibits significantly greater catalytic efficiency. Cryo-EM structural analysis of *Pp*ClpP1 revealed a canonical homotetradecameric architecture featuring two notable structural features: (1) an unconventional Ser-His-Pro catalytic triad configuration, and (2) an unusually dilated axial pore. Through structural modeling of *Pp*ClpP2, we identified key recognition elements for *Pp*ClpX interaction that are conspicuously absent in *Pp*ClpP1. *In vitro* interaction assays demonstrated that *Pp*ClpP1 and *Pp*ClpP2 form a heterotetradecamer (*Pp*ClpP1P2), which displays significantly augmented peptidase activity compared to individual homotetradecamer. The unfoldase *Pp*ClpX specifically engages the complex through recognition of *Pp*ClpP2, forming active proteases with either *Pp*ClpP2 alone (*Pp*ClpP2-ClpX) or the *Pp*ClpP1P2 heterotetradecamer (*Pp*ClpP1P2-ClpX). Notably, the proteolytic activity of the *Pp*ClpP1P2-ClpX complex exceeded that of the *Pp*ClpP2-ClpX complex, indicating that the heterotypic association enhances the functional output of the protease. Using a multidisciplinary approach combining biochemical assays, isothermal titration calorimetry (ITC), and molecular docking, we elucidated the molecular mechanism underlying bortezomib-mediated inhibition of *Pp*ClpP1. The therapeutic potential of this inhibition was confirmed through genetic knockout experiments, which demonstrated significant suppression of *P. plecoglossicida* growth upon bortezomib treatment. To our knowledge, this study represents the first structural characterization of *Pp*ClpP and identification of a novel ClpP inhibition mechanism effective against *P. plecoglossicida*.

## 2. Materials and methods

### 2.1. Bacterial strains

The *P. plecoglossicida* wild-type (WT) strain PQLYC4 was isolated from diseased *Larimichthys crocea* with visceral white nodules disease [21], and cultured in trypticase soy broth (TSB, Hopebio, China) or trypticase soy agar (TSA) at 28°C. *E. coli* strains DH5α, BL21DE3 (Invitrogen), and S17-1 (AngYuBio) were cultured in Luria-Bertani broth (LB, Hopebio, China) or on LB agar at 37°C.

### 2.2. Construction of Δ*Pp*ClpP1 and Δ*Pp*ClpP2 *P. plecoglossicida*

The Δ*Pp*ClpP1 and Δ*Pp*ClpP2 mutants were generated through homologous recombination using a suicide vector strategy [22]. The 500 bp upstream (N-terminal) and downstream flanking regions of the *Pp*ClpP1 and *Pp*ClpP2 open reading frame were amplified using primer sets Δ*Pp*ClpP1-U F/R, Δ*Pp*ClpP2-U F/R, Δ*Pp*ClpP1-D F/R, and Δ*Pp*ClpP2-D F/R, respectively. These fragments were subsequently fused by overlap extension PCR using external primers Δ*Pp*ClpP1-U F, Δ*Pp*ClpP2-U F, Δ*Pp*ClpP1-D R, and Δ*Pp*ClpP2-D R. The resulting fusion product was cloned into the suicide vector pEX18Tc at KpnI/HindIII restriction sites, generating the recombinant plasmid pEX18Tc-*Pp*ClpP1 or pEX18Tc-*Pp*ClpP2. After transformation into *E. coli* S17-1, positive clones were selected on LB agar supplemented with 10 μg/ml tetracycline. Conjugation was performed to transfer pEX18Tc-*Pp*ClpP1 or pEX18Tc-*Pp*ClpP2 from *E. coli* S17-1 to wild-type *P. plecoglossicida*. Primary recombinants were selected on Tryptic Soy Agar (TSA) plates containing both tetracycline (10 μg/ml) and ampicillin (100 μg/ml), taking advantage of the native ampicillin resistance of wild-type *P. plecoglossicida*. Secondary recombinants were then isolated on TSA plates containing 12% (w/v) sucrose to select for vector loss. Mutant validation was performed using two PCR strategies: (1) amplification across the deletion junction using primers Δ*Pp*ClpP1-U F/Δ*Pp*ClpP1-D R or Δ*Pp*ClpP2-U F/Δ*Pp*ClpP2-D R, and (2) internal verification using primer set *Pp*ClpP1-F/R or *Pp*ClpP2-F/R. All PCR products were confirmed by Sanger sequencing. Primer sequences were designed based on the complete genome sequence of *P. plecoglossicida* strain PQLYC4 (NCBI accession PRJNA612395), with all oligonucleotides listed in S1 Table.

### 2.3. Bacterial growth curve and plate titration assays

To evaluate the effect of bortezomib (BTZ) on bacterial growth, *P. plecoglossicida* strains (PQLYC4-WT and knockout PQLYC4-Δ*Pp*ClpP1/Δ*Pp*ClpP2) were cultured to an initial optical density at 600 nm (OD₆₀₀) of 0.1, followed by treatment with BTZ at two-fold serial dilution concentrations. Growth was monitored spectrophotometrically (OD_600_) hourly for 15 h to generate growth curves. For plate titration assays, bacterial cultures were adjusted to OD_600_ ≈ 0.4 with double distilled water, and 10-fold serial dilution was performed on TSB containing 1.25 μM BTZ, then plated on TSA plates to determine the number of surviving colony forming bacteria.

### 2.4. Bacterial motility assays

Wild-type, Δ*Pp*ClpP1 and Δ*Pp*ClpP2 knockout strains were revived from glycerol stocks stored at –80°C by streaking onto TSA plates. The plates were incubated at 28°C for 24–48 h. A single colony was then inoculated into TSB medium and cultured with shaking at 200 rpm and 28°C until the OD₆₀₀ reached approximately 0.6. Swimming and swarming motilities were assessed using freshly prepared semi-solid agar plates. For swimming motility, 0.3% agar was used, while 0.6% agar was employed for swarming motility. Bacterial cultures were spot-inoculated at the center of the plates. The inoculated plates were incubated upright at 28°C. Phenotypes were observed and photographed after 24 h for swimming motility and 48 h for swarming motility.

### 2.5. Plasmid construction and protein expression

*Pp*ClpP1 (1–183, AXM94812) and *Pp*ClpP2 (1–206, WP_003259400.1) and *Pp*ClpX (QLB56231.1) DNA were directly amplified with a primer set (S1 Table) from *P. plecoglossicida* PQLYC4 strain cDNA using RT-PCR, and cloned into pET28a (N-terminal with 6 × His) expression vector using BamHI and XhoI restriction enzymes. Full-length sequences *Pp*ClpP1 and *Pp*ClpP2 were constructed on the pET-Duet vector (for coexpression of *Pp*ClpP1-His_6_ and *Pp*ClpP2-StrepII). Site-directed mutagenesis of *Pp*ClpP1 and *Pp*ClpP2 were performed using the DpnI method. The active site mutants of *Pp*ClpP1 included S73A, H96A, P151A, and P151D; the BTZ-binding site mutants of *Pp*ClpP1 included G44A, E45A, C46A, S47A, F98A, H99A, W100A, T101A, S117A, and D121A. The active site mutants of *Pp*ClpP2 included S114A, H139A, D188A, and D188P. All the expression plasmids were expressed in *E. coli* BL21 cells following overnight expression at 20°C after induction with 1 mM isopropyl-β-D-thiogalactopyranoside (IPTG). Cells were pelleted by centrifugation at 8000 *g* for 30 min and stored at -20°C until puriﬁcation.

### 2.6. Protein puriﬁcation

The cell pellet was thawed and resuspended in lysis buffer (50 mM Tris pH 7.5, 150 mM NaCl, and 2 mM β-mercaptoethanol with a PMSF (Phenylmethylsulfonyl fluoride). Cells were lysed mechanically by three passes through a high-pressure instrument. Cell lysate was clariﬁed via centrifugation at 17,000 *g* for 30 min and the supernatant ﬁltered using a 0.45 μm ﬁlter (EMD Millipore). Protein were purified using native NiNTA or Strep affinity chromatography followed by Size-Exclusion Chromatography (SEC) using a Superdex 200 Increase columns (Cytiva Bio-technology) and a final SEC buffer containing 50 mM Tris pH 7.5, 150 mM NaCl, and 5% glycerol.

### 2.7. Real-time quantitative PCR

Culture *P. plecoglossicida* PQLYC4 in TSB medium and collect cells at different growth stages with OD values (0.1, 0.8, 1.5, and 2.0). According to the manufacturer’s instructions, the cultured cells were then sampled for total RNA extraction using the Eastep super total RNA extraction kit (Promega). The cDNA was synthesized using GoScript reverse transcription mix (Promega). The real-time PCR analysis was carried out using TB Green mix (Takara) and speciﬁc primers (S1 Table) in a QuantStudio 5 real-time PCR system (Thermo Fisher Scientiﬁc). Gene expression levels were normalized against the reference gene *Pp*GyrB using the 2^-ΔΔCt^ method. All data were obtained from three independent experiments, and each analysis was performed in triplicate.

### 2.8. Sequence alignment and phylogenetic tree analysis

Protein sequences were aligned using the CLUSTALW-Multiple Sequence Alignment website (https://www.genome.jp/tools-bin/clustalw), as well as the ESPript 3.0 server Aligned Sequences website (https://espript.ibcp.fr/ESPript/ESPript/). Amino acids have been colored with similarity coloring scheme % MultAlin, Global score 0.7. Phylogenetic trees were constructed using MEGA 11 software for data processing and visualization.

### 2.9. Size-exclusion chromatography

Size-exclusion chromatography were performed with a HiLoad 16/60 Superdex 200 column (Cytiva Bio-technology) on an ÄKTApuriﬁer System with UV detector (UPC 900, P900, Box900, Frac950, Cytiva Bio-technology). We injected 2 mg of protein via a 1 mL sample loop with the respective buffer at a ﬂow rate of 0.5 mL/min.

### 2.10. Analytical ultracentrifugation

Sedimentation velocity experiments were conducted using a Beckman Proteomlab XL-I analytical ultracentrifuge equipped with scanning UV/visible optics. An-60 Ti four-hole rotor and cells with 12 mm charcoal centerpieces and quartz windows were used. The assay volume was 400 μL with a protein concentration set to 0.8 mg/mL and samples were analyzed at 30,000 rpm and 16°C scanning continuously until complete sedimentation was achieved. The data were then analyzed using a continuous c(s) distribution and SEDFIT version 13.0b software [23].

### 2.11. Peptidase activity

All assays of peptidase activity were performed at 37°C for 2 h, in black 96-well plates using a Plate Reader Varioskan LUX (Thermo Scientific). Each well contained 200 μM Suc-LLVY-AMC (MedChemExpress, HY-P1002) fluorogenic peptide, 1 μM recombinant protein in 100 μL of buffer (20 mM HEPES, pH 7.5, 100 mM KCl, 5% glycerin). DMSO as the experimental control group, and DMSO concentration never exceeded 2%. The reaction was initiated by the addition of the enzyme, and peptidase activity was followed in the linear range by monitoring the rate of production of fluorescent 7-amino-4-methylcoumarin-amc from peptide-AMC substrates at 460 nm (excitation at 380 nm) [24]. The deviation of fluorescence value in three independent measurements was not ＞ 5%.

### 2.12. Cryo-EM sample preparation and data collection

Frozen-hydrated specimens were prepared using a Vitrobot Mark IV plunger (Thermo Fisher Scientific). *Pp*ClpP1 protein (as shown in S1C Fig) was loaded at the concentration of 0.5 mg/mL onto a freshly glow-discharged (30 s at 15 mA) holey carbon grid (Quantifoil Cu R1.2/1.3). The excess solution from the grid was blotted for 4 s at 100% humidity and 4°C before the grid was plunged into liquid ethane. For structure determination, the frozen grids were loaded into a 200 kV Glacios electron microscope at School of life sciences, Anhui university for automated image acquisition with EPU [25]. Movies were recorded on a Falcon 4 camera equipped with Slectris energy filter (±5 eV) at 165K nominal magnification (calibrated pixel size of 0.698 Å) and defocus values ranging from -0.4 to -2.6 μm. During data collection, the total dose was 50 e^−^/A^2^. The detailed collection statistics are shown in S2 Table.

### 2.13. Structure analysis and model refinement

Cryo-EM analysis was performed using CryoSPARC [26]. All frames in each collected movie were aligned and summed using Patch Motion Correction, and CTF estimation were made using Patch CTF Estimation. Blob Picker and Template Picker were used for particle picking, and particles were extracted using a box size of 300 * 300 pixels [27]. 2D classifications and 3D classifications were used to remove junk particles and select the most homogeneous particles for in-depth 3D structural analyses. The final 3D reconstruction for each class was done using Non-Uniform Refinement, and the resulting map was post-processed using DeepEMhancer.

The reported resolution is based on the “gold standard” refinement procedure and the 0.143 Fourier Shell Correlation (FSC) criterion [28]. Local resolution was estimated using Local Resolution Estimation. For model building, the AlphaFold-predicted tetradecameric structure of *Pp*ClpP1 was used as initial models to fit into the maps using Chimera, and the resulting models were manually adjusted and rebuilt according to the cryo-EM map in COOT [29]. Phenix real-space refinement was used to refine the models [30]. The refinement statistics are shown in S2 Table. The detailed classifications and map qualities of *Pp*ClpP1 are shown in the S2 Fig.

### 2.14. Nano differential scanning fluorimetry

Protein stability was determined using the nano differential scanning ﬂuorimetry (nanoDSF) method based on intrinsic tryptophan or tyrosine ﬂuorescence [31]. All nanoDSF assays were performed using the Prometheus NT.48 instrument (NanoTemper Technologies). *Pp*ClpP1 protein samples (10 μl at 1 mg/mL) and *Pp*ClpP1-bortezomib (1 mM) complex were loaded into standard-grade nanoDSF capillaries, placed on the prometheus capillary holder, and subjected to a temperature ramping of 1°C/min from 20 to 94.8°C. The melting point (Tm) onset (°C) and Tm (°C) values, which indicate the structural stability of the samples, were obtained by monitoring the intrinsic tryptophan and tyrosine ﬂuorescence at the emission wavelengths of 330 and 350 nm. The ratio of the ﬂuorescence intensities (F350 nm/F330 nm) was plotted versus temperature or time to generate an unfolding curve. The thermal stability of a sample was described by the thermal unfolding transition midpoint Tm (°C), at which half of the protein population is unfolded. The Tm value corresponded to the inﬂection point of the unfolding curve and was determined via the derivative of the curve.

### 2.15. Isothermal titration calorimetry

The afﬁnity experiments were performed in a Nano ITC (low volume) (TA Instruments) [32]. Protein samples were prepared as above. All samples for the assays were prepared in the buffer containing 50 mM Tris (pH 7.5) and 150 mM NaCl. To measure the afﬁnity between bortezomib and *Pp*ClpP1 or *Pp*ClpP2, 300 μM bortezomib in the syringe was titrated into a sample cell containing 30 μM recombinant protein *Pp*ClpP1 or *Pp*ClpP2. All experiments were carried out at 25°C. Data correction and analyses were performed in NanoAnalyze software (TA Instruments).

### 2.16. Molecular simulation of interaction between bortezomib and *Pp*ClpP1

Molecular docking was performed to analyze the mechanism of the binding that occurs between bortezomib and *Pp*ClpP1 monomer by Autodock Vina (Scripps Research Institute, La Jolla, CA, USA) [33,34]. Energy minimization of the protein and ligand were performed by Molecular Operating Environment (MOE) software. It shows ligand-binding ﬂexibility with the binding pocket residues. The lowest energy conformations were used for analysis and a picture was generated by Pymol software.

### 2.17. Pull-down analysis of *Pp*ClpX with *Pp*ClpP proteases

Protein interactions between the unfoldase *Pp*ClpX and different *Pp*ClpP proteases were analyzed by pull-down assays. His-tagged *Pp*ClpX (65–414 aa) and the *Pp*ClpP1P2 complex were expressed as described in Materials and methods section 2.5. To obtain Strep-tagged *Pp*ClpP1 and *Pp*ClpP2, the corresponding genes were cloned into pET-28a (with the His-tag removed and an N-terminal Strep-tag inserted). For His pull-down assays, protein complexes were incubated with Ni-NTA agarose purification resin (Cytiva). Resins were washed with buffer containing 50 mM Tris-HCl (pH 7.5), 150 mM NaCl, and 0.2% Triton X-100, and bound proteins were eluted with elution buffer (300 mM imidazole, 50 mM Tris-HCl [pH 7.5], 150 mM NaCl, 0.2% Triton X-100). For Strep pull-down assays, protein complexes were incubated with Strep agarose purification resin (Yeasen Biotechnology Co., Ltd.). After washing with the same buffer as above, proteins were eluted with buffer containing 20 mM D-biotin, 50 mM Tris-HCl (pH 7.5), 150 mM NaCl, and 0.2% Triton X-100. All samples were separated by SDS-PAGE and visualized by Coomassie blue staining.

### 2.18. Degradation assay of GFP-ssrA and FITC-labeled casein

Degradation assays were performed using GFP-ssrA or FITC-labeled casein as substrates. Each 50 μL reaction contained 1 μM of the specified protein components (BSA, *Pp*ClpP1, *Pp*ClpP2, *Pp*ClpP1P2, *Pp*ClpX, *Pp*ClpX + *Pp*ClpP1, *Pp*ClpX + *Pp*ClpP2, or *Pp*ClpX + *Pp*ClpP1P2 complex) were mixed with 50 μL of 1 μM GFP-ssrA or FITC-casein substrate. Reactions were carried out in a buffer containing 25 mM HEPES-KOH (pH 7.5), 150 mM KCl, 20 mM MgCl₂, and 2 mM ATP, and incubated at 37°C for 2 h. Fluorescence was measured using a Molecular Devices FlexStation 3 microplate reader, with an excitation wavelength of 470 nm and an emission wavelength of 510 nm for GFP-ssrA, an excitation wavelength of 498 nm and an emission wavelength of 517 nm for FITC-casein. The GFP-ssrA fusion protein was constructed by fusing the *E. coli* ssrA sequence to the GFP sequence, cloned into a pET-28a vector, and purified using an *E. coli* prokaryotic expression system. FITC-labeled casein (C275894) was commercially obtained from Aladdin.

### 2.19. Statistical analysis

Statistical analysis was performed using the IBM SPSS Statistics 2.0 software platform with one-way ANOVA. A *p* value <0.05 was considered statistically signiﬁcant.

## 3. Results

### 3.1. Characterization and enzymatic activity of ClpP proteases from *P. plecoglossicida*

**Fig 1:**
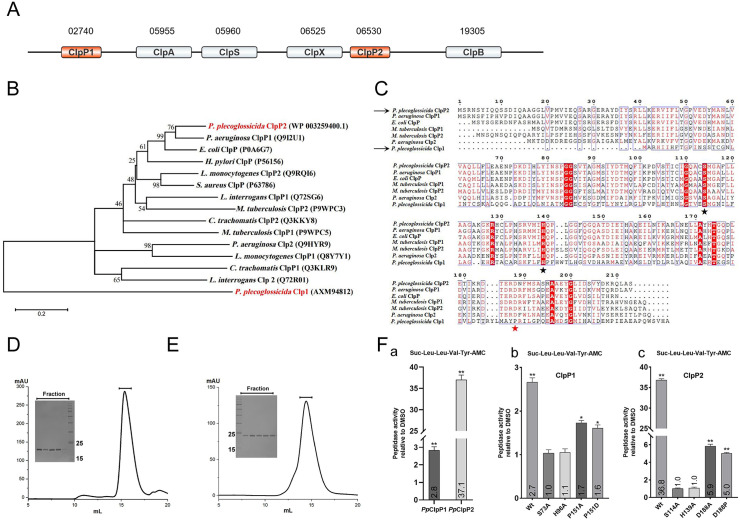
Characterization and enzymatic activity of ClpP proteases from *P. plecoglossicida.* **(A)** Schematic representation of a *P. plecoglossicida* gene cluster encoding Clp protein family components, numbers correspond to genomic loci of the proteins. **(B)** Phylogenetic tree of ClpP proteins constructed using neighbor-joining method based on multiple sequence alignment **(**S1A Fig). Bootstrap values (>50%) indicate branch support, with species and protein accessions labeled accordingly. **(C)** Representative multiple sequence alignment of selected ClpP homologs highlighting catalytic residues: Serine-S and Histidine-H marked with black asterisks, while Aspartic-D and Proline-P are indicated by red asterisks. Size-exclusion chromatography (SEC) elution profiles of recombinant *Pp*ClpP1 **(D)** and *Pp*ClpP2 **(E)**. **(F)** Assessment of peptidase activity in *P. plecoglossicida* ClpP. **(a)** Comparative analysis of peptidase activity in *Pp*ClpP1 versus *Pp*ClpP2. **(b)** Peptidase activity of *Pp*ClpP1 catalytic triad mutants. **(c)** Peptidase activity of *Pp*ClpP2 catalytic triad mutants. Values indicated at the base of each bar denote the fold-change in fluorescence intensity relative to the DMSO-treated control. Bars represent the means of three independent experiments ± SD. All experiments were performed in triplicate. *p < 0.05, **p < 0.01.

Many bacterial *clpP1* and *clpP2* genes are located at distinct genomic loci and the paralogs appear to serve varying functions in vivo [13,16,35]. Similarly, *P.* *plecoglossicida* PQLYC4 strain encodes two distinct ClpP protease proteolytic subunits, designated as *Pp*ClpP1 and *Pp*ClpP2 (Fig 1A). Phylogenetic analysis revealed that *Pp*ClpP2 clusters closely with canonical ClpP homologs, showing particularly high similarity to *P. aeruginosa* ClpP1 (Fig 1B). In contrast, *Pp*ClpP1 forms an evolutionarily divergent clade, suggesting either functional specialization or acquisition of unique regulatory mechanisms during evolution. Sequence alignment demonstrated that *Pp*ClpP1 contains an N-terminal truncation (>15 amino acid truncation) compared to typical ClpP proteases (S1A Fig). Most notably, *Pp*ClpP1 possesses a non-canonical catalytic triad configuration (Ser-His-Pro), differing from both the conserved Ser-His-Asp triad (Fig 1C) or the rare Ser-His-Asn triad (S1A Fig) variant found in some bacterial species. Transcript analysis showed significantly higher basal expression levels of *Pp*ClpP2 compared to *Pp*ClpP1 (S1B Fig), indicating potential differential roles in cellular metabolism.

To investigate the functional consequences of these structural variations, we performed comparative biochemical analyses. We conducted in vitro analytical ultracentrifugation (AUC) assays using recombinant proteins expressed and purified in *E. coli* (Fig 1D and 1E). AUC confirmed that both proteases form canonical tetradecamers (S1C and S1D Fig). Enzymatic characterization using the fluorogenic substrate Suc-LLVY-AMC revealed that *Pp*ClpP2 exhibits substantially higher proteolytic activity, demonstrating a 13-fold greater catalytic efficiency compared to *Pp*ClpP1 (Fig 1F-a). To investigate whether the unique proline residue in the catalytic triad contributes to the low peptidase activity of *Pp*ClpP1, we generated amino acid substitutions of the catalytic triad residues in both *Pp*ClpP1 and *Pp*ClpP2 and assessed their enzymatic activity. Substitution of the catalytic serine-S or histidine-H with alanine-A completely abolished peptidase activity in both proteases, confirming their essential roles. In contrast, replacing the proline-P in *Pp*ClpP1 with either alanine-A or aspartate-D only partially reduced activity, retaining approximately 60% of wild-type levels (Fig 1F-b). Similarly, mutating the aspartate-D in *Pp*ClpP2 to alanine-A or proline-P did not eliminate activity, with residual levels of approximately 16% and 13%, respectively (Fig 1F-c). These results establish that while the catalytic triad is essential for proteolytic function in both proteases, the serine and histidine residues are absolutely indispensable, whereas the third position (proline in *Pp*ClpP1, aspartate in *Pp*ClpP2) modulates but is not strictly required for activity.

Our findings reveal fundamental functional differences between the two ClpP paralogs in *P. plecoglossicida*. Whereas *Pp*ClpP2 maintains the conserved structural and functional characteristics typical of bacterial ClpP proteases, *Pp*ClpP1 exhibits multiple atypical features, including phylogenetic divergence, N-terminal truncation, and a novel catalytic triad configuration. These distinctive attributes position *Pp*ClpP1 as an intriguing subject for future studies on protease evolution and non-canonical mechanisms of proteolytic regulation.

### 3.2. Overall structure of *P. plecoglossicida* ClpP1

**Fig 2:**
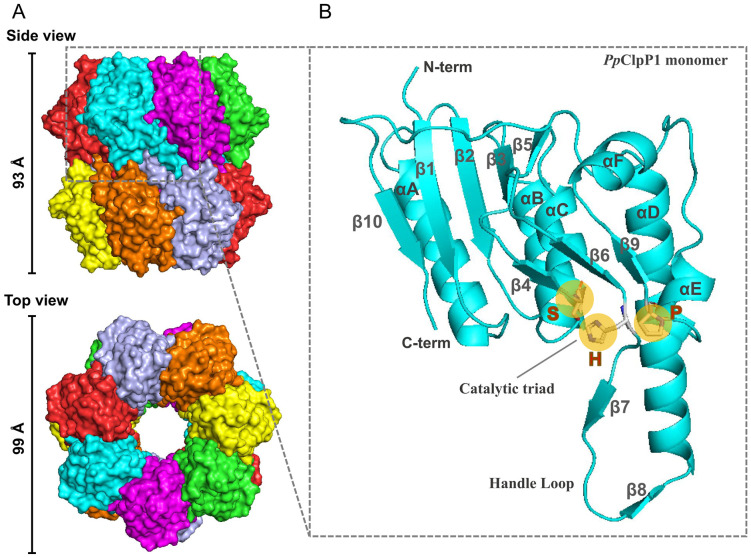
Structural architecture of *Pp*ClpP1. **(A)** Surface representation of the tetradecameric *Pp*ClpP1 complex (PDB ID 9UXT) showing top and side views, with each monomer in the heptameric rings colored distinctly for clarity. **(B)** Ribbon diagram of an individual subunit highlighting the secondary structure elements, including six α-helices (denoted with α-letters) and ten β-strands (denoted with β-numbers), with the catalytic triad (S-H-P) prominently marked by yellow circles.

To elucidate the structural features of *Pp*ClpP1 and *Pp*ClpP2, we attempted to determine their three-dimensional structures using both X-ray crystallography and cryo-electron microscopy (Cryo-EM). However, we only succeeded in solving the structure of *Pp*ClpP1. We determined its Cryo-EM structure at an average resolution of 3.07 Å using recombinant protein (S2A–S2F Fig). The asymmetric unit consists of two opposing heptameric rings forming a barrel-shaped homotetradecamer with dimensions of 93 Å in height and 99 Å in diameter (Figs 2A and S3A). Each monomer adopts the canonical ClpP fold, featuring five parallel β-strands (β1-β2-β4-β6-β9) flanked by four α-helices (αA-αD) arranged perpendicular to the β-sheet plane (Fig 2B) [36–38]. Notably, the αD helix forms a distinctive handle domain together with β7 and β8. As predicted by sequence alignment (Fig 1C), structural analysis confirmed the presence of an unprecedented catalytic triad (S73-H96-P151) within the proteolytic chamber. This geometric perturbation at the catalytic center likely underlies the observed attenuation of proteolytic activity. Specifically, a Pro in this position is unable to provide electrostatic stabilization to the positive charge of the doubly protonated form of the His, which acts as the proton acceptor of the catalytic Ser during its activation. The tetradecameric interface is stabilized by interlocking handle domains from opposing heptamers (S3B-a Fig), involving: (1) interactions between two disordered domains (S3B-b Fig), and (2) contacts between αD-helices (S3B-c Fig). The heptameric interfaces feature front-to-back interactions where α-helices of one subunit align with β-sheets of another (S3C-a Fig), with each α-helix and corresponding β-sheet forming three distinct interaction regions (S3C-b–S3C-d Fig).

### 3.3. Distinguishing features of *P. plecoglossicida* ClpP1

**Fig 3:**
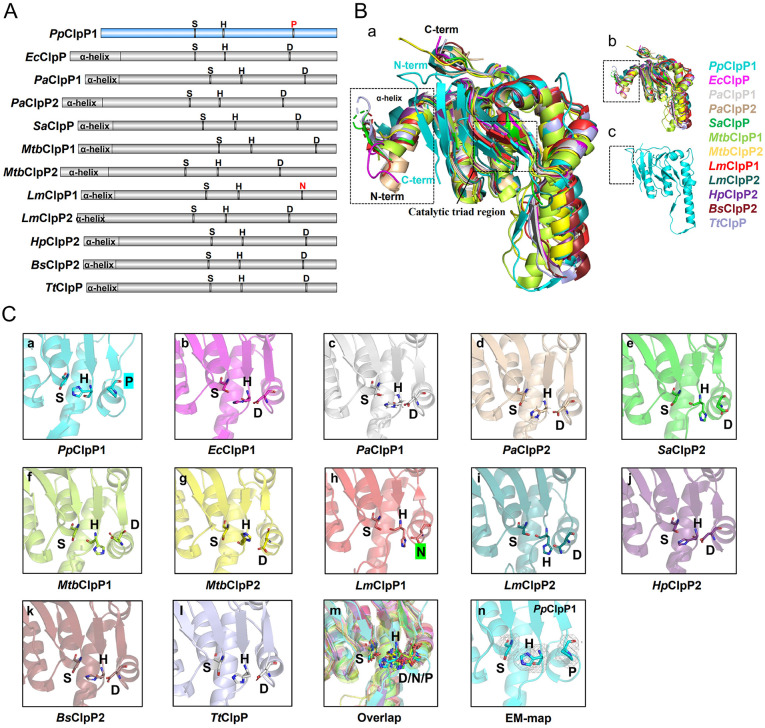
Contrasting structural features of *Pp*ClpP1 compared to homologs. **(A)** Domain architecture comparison showing sequence length and catalytic active sites between *Pp*ClpP1 (blue) and other structurally characterized ClpP homologs (gray). **(B)** a, Structural superposition of *Pp*ClpP1 monomer with representative ClpP structures from *E. coli* (*Ec*ClpP1, 3HLN), *P. aeruginosa* (*Pa*ClpP1/2, 7M1M/7M1L), *S. aureus* (*Sa*ClpP, 3V5E), *M. Tuberculosis* (*Mtb*ClpP1/P2, 6VGK), *L. monocytogenes* (*Lm*ClpP1/2, 4JCT/4JCQ), *H. pylori* (*Hp*ClpP2, 2ZL0), *B. subtilis* (*Bs*ClpP2, 7FEQ), and *T. thermophilus* (*Tt*ClpP2, 6HWM). b-c, Dashed boxes highlight the N-terminal α-helical regions (absent in *Pp*ClpP1) and catalytic triad sites, with each homolog colored distinctly. **(C)** Detailed views of catalytic triad configurations in various ClpP homologs (a-l) and their structural superposition (m), demonstrating the unique Ser-His-Pro arrangement in *Pp*ClpP1 compared to conventional Ser-His-Asp/Asn triads in other species, (n) Cryo-EM density for the catalytic triad of *Pp*ClpP1. The comparative analysis reveals *Pp*ClpP1’s distinctive structural adaptations at both N-terminal and active site regions.

Comparative structural analysis with ClpP homologs reveals two defining characteristics that distinguish *Pp*ClpP1. First, *Pp*ClpP1 possesses the shortest polypeptide chain among characterized ClpP proteases (Fig 3A), lacking the N-terminal α-helical domain (Fig 3B). This truncation results in a dramatically expanded axial pore (42.7 Å diameter) formed by the heptameric ring assembly, significantly larger than those reported for other ClpPs (S4A Fig). Since the N-terminal domain normally constrains pore size and mediates interactions with pore-2 loops of ClpX/C/A unfoldases, its absence likely affects substrate processing and unfoldases recognition [39,40]. Second, while maintaining the conserved spatial arrangement of catalytic residues (Fig 3B and 3C), *Pp*ClpP1 uniquely substitutes the conventional aspartate/asparagine with proline (S-H-P triad) - a configuration previously undocumented in bacterial ClpP proteases (Fig 3C). Structural alignment shows that Ser73 and His96 maintain positions similar to those in other ClpPs (Fig 3C-m), while Pro151 occupies a distinct spatial orientation compared to catalytic aspartates in *P. aeruginosa* ClpP1/ClpP2 (S4B Fig). Although this proline substitution represents a moderate structural change, it causes significant divergence in the orientation of the catalytic triad’s charge-relay system. This altered geometry likely explains *Pp*ClpP1’s reduced activity, as the S-H-P configuration may maintain the protease in a suboptimal activation state.

### 3.4. Structural comparison of *P. plecoglossicida* ClpP1 and ClpP2

**Fig 4:**
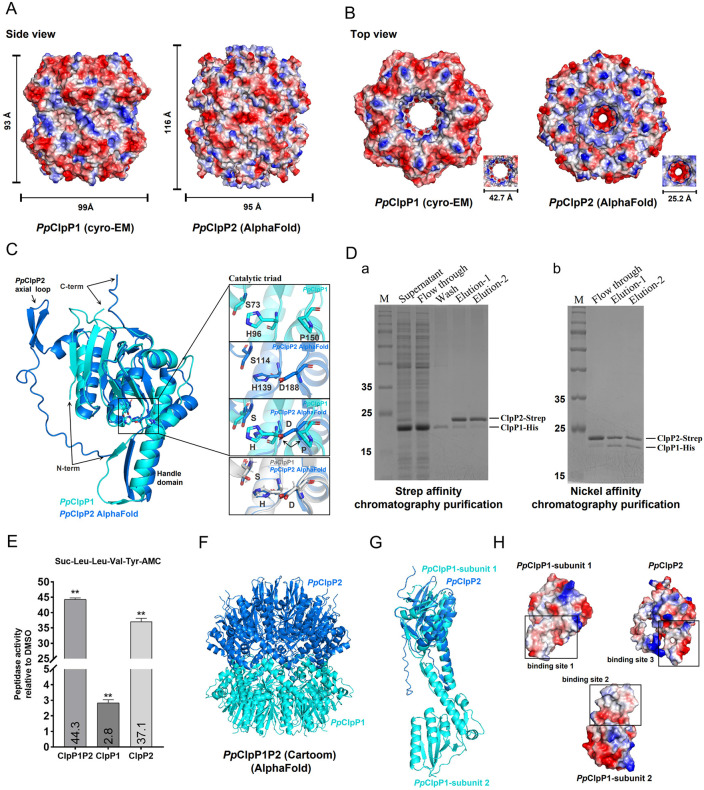
Distinct structural features and functional interplay between *Pp*ClpP1 and *Pp*ClpP2. **(A)** Side views of electrostatic potential surfaces for the *Pp*ClpP1 tetradecamer (left) and the predicted *Pp*ClpP2 tetradecamer (right) generated by AlphaFold. **(B)** Top views of electrostatic potential surfaces for the *Pp*ClpP1 tetradecamer (left) and the predicted *Pp*ClpP2 tetradecamer (right). Inset images illustrate the axial pore diameter sizes of *Pp*ClpP1 (42.7 Å) and *Pp*ClpP2 (25.2 Å), respectively. **(C)** Structural alignment of *Pp*ClpP1 (Cryo-EM structure) with *Pp*ClpP2 (predicted model) highlighting domain organization (left) and catalytic triad configurations (right), including superposition with *P. aeruginosa* ClpP1 (*Pa*ClpP1). **(D)** Purification of the co-expressed *Pp*ClpP1-*Pp*ClpP2 complex. **(a)** Schematic of the initial Strep-tag affinity purification. Supernatant: supernatant after *E. colicell* lysis; Flow-through: flow-through fraction from the Streptactin resin; Wash: washing with buffer (150 mM NaCl, 50 mM Tris-HCl, pH 8.0); Elution: elution with buffer containing 2.5 mM D-desthiobiotin, 150 mM NaCl, 100 mM Tris-HCl, pH 8.0. **(b)** Subsequent Ni-NTA affinity purification of the eluate from panel a. Flow-through: flow-through fraction from the Ni-NTA resin; Elution: elution with buffer containing 300 mM imidazole, 150 mM NaCl, 50 mM Tris-HCl, pH 8.0. **(E)** Comparative analysis of the peptidase activities among the *Pp*ClpP1P2 heterotetradecamer, the *Pp*ClpP1 homotetradecamer, and the *Pp*ClpP2 homotetradecamer. **(F)** The structure model of *Pp*ClpP1P2 heterotetradecamer predicted using AlphaFold. **(G)** Cartoon model depicting the interaction between a single *Pp*ClpP1 subunit and a single *Pp*ClpP2 subunit. **(H)** Analysis of the electrostatic potential at the homomeric (*Pp*ClpP1-*Pp*ClpP1) and heteromeric (*Pp*ClpP1-*Pp*ClpP2) interaction interfaces.

Complementing our structural characterization of *Pp*ClpP1, we employed computational approaches to investigate its paralog *Pp*ClpP2. Using AlphaFold prediction [41], we generated a structural model of *Pp*ClpP2 based on fragments from homologous PDB structures (S5A Fig), the model showed high confidence (pLDDT > 90, ipTM = 0.87, pTM = 0.88), supporting its reliability for structural analysis. Three-dimensional comparisons revealed significant architectural differences between the two paralogs. The predicted *Pp*ClpP2 structure exhibits distinct dimensional characteristics, with increased longitudinal spacing (116 Å) and reduced transverse dimensions (95 Å) compared to *Pp*ClpP1 (Fig 4A). Notably, the axial pore diameter of *Pp*ClpP2 heptamers (25.2 Å) is substantially narrower than that of *Pp*ClpP1 (42.7 Å) (Fig 4B). This constrained geometry likely enhances substrate stabilization and proteolytic efficiency through a more confined channel architecture. Key structural differences extend to functional domains. Unlike *Pp*ClpP1, which lacks N-terminal helices and has an atypical catalytic triad (Fig 3B), *Pp*ClpP2 maintains a flexible N-terminal architecture featuring: a mini-helix, dual β-strands, and axial loop (Fig 4C-left). *Pp*ClpP2 adopts a compact S-H-D configuration resembling the activated state of *P. aeruginosa* ClpP1 (*Pa*ClpP1), this contrasts with *Pp*ClpP1’s atypical S-H-P arrangement (Fig 4C-right). The canonical triad architecture of *Pa*ClpP1 correlates with high peptidase activity [42], explaining *Pp*ClpP2’s significantly greater enzymatic efficiency compared to *Pp*ClpP1.

### 3.5. Assembly and synergistic activation of the *Pp*ClpP1P2 heterotetradecameric complex

The potential formation of heterotetradecameric complexes between *Pp*ClpP1 and *Pp*ClpP2 in vivo represents an important biological question. Our co-purification experiments revealed stable complex formation when both subunits were co-expressed in *E. coli* (Fig 4D), AUC analysis further indicated that the *Pp*ClpP1P2 complex has a molecular mass consistent with a heterotetradecameric assembly (S5B Fig). Notably, the heterotetradecamer exhibited significantly higher peptidase activity than either *Pp*ClpP1 or *Pp*ClpP2 alone (Fig 4E), suggesting that complex formation may induce conformational changes in *Pp*ClpP1 that enhance its proteolytic efficiency.

To elucidate the structural basis of heterocomplex assembly, we predicted the *Pp*ClpP1P2 heterotetradecamer structure using AlphaFold (Fig 4F). The model showed high confidence (pLDDT > 90, ipTM = 0.81, pTM = 0.82), supporting its reliability for structural analysis. Structural alignment demonstrated significant overlap between the handle domains of *Pp*ClpP2 and *Pp*ClpP1 (Fig 4G). The binding interface between *Pp*ClpP2 (binding site 3) and *Pp*ClpP1 subunit 2 (binding site 2) is predominantly hydrophobic (Fig 4H), resembling the hydrophobic character of the *Pp*ClpP1 homomeric interface (binding site 1). This conserved hydrophobic nature likely facilitates stable hetero-oligomerization through similar interaction mechanisms as those maintaining homo-oligomeric assemblies.

### 3.6. *Pp*ClpX recognizes *Pp*ClpP2 to drive proteolysis enhanced by *Pp*ClpP1 heterocomplex formation

**Fig 5:**
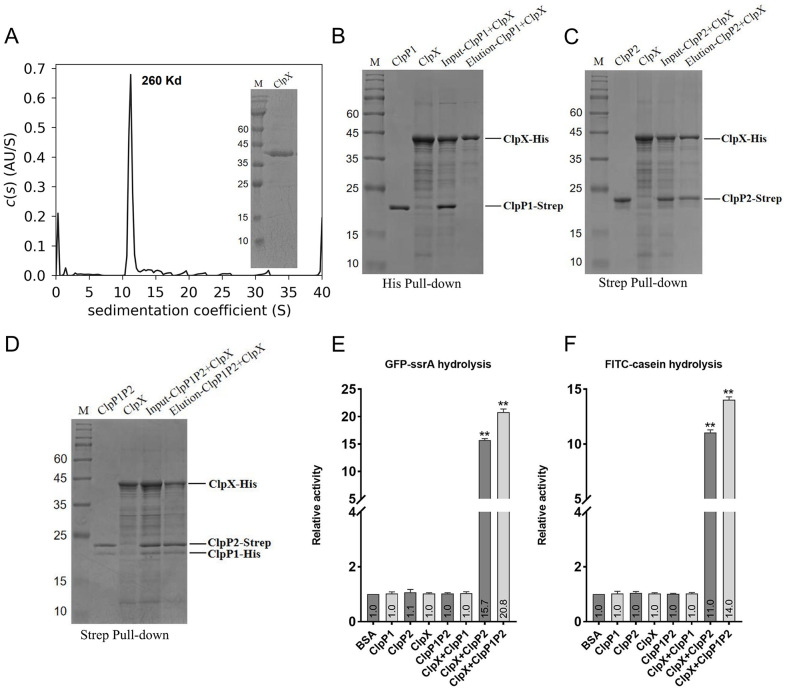
Biochemical and functional characterization of *Pp*ClpX interactions with homomeric *Pp*ClpP1, *Pp*ClpP2, and the heteromeric *Pp*ClpP1P2 Complex. **(A)** Purification and analytical ultracentrifugation (AUC) analysis of the truncated *Pp*ClpX (65-414 aa). The sedimentation profile indicates that *Pp*ClpX elutes as a stable hexamer. An SDS-PAGE analysis of the purified protein is shown on the right. **(B-D)** Pull-down assays assessing the interaction between *Pp*ClpX and different *Pp*ClpP isoforms. **(B)** His pull-down assay with *Pp*ClpX-His, *Pp*ClpP1-Strep can not co-elute with *Pp*ClpX-His. **(C)** Strep pull-down assay with *Pp*ClpP2-Strep, *Pp*ClpX-His was specifically pulled down by *Pp*ClpP2-Strep. **(D)** Strep pull-down assay with the *Pp*ClpP1P2 heterocomplex, *Pp*ClpX-His was pulled down by the *Pp*ClpP1P2 heterocomplex. **(E-F)** Degradation assays of fluorescent substrates by the respective active protease complexes. Degradation of GFP-ssrA (E) or FITC-casein **(F)**. Significant protease activity is observed only in the presence of both *Pp*ClpX (full-length) and *Pp*ClpP2 or the *Pp*ClpP1P2 heterocomplex. Values indicated at the base of each bar denote the fold-change in fluorescence intensity relative to the BSA-treated control. Bars represent the means of three independent experiments ± SD. All experiments were performed in triplicate. **p < 0.01.

Based on structural analysis, we hypothesized that *Pp*ClpP1, unlike *Pp*ClpP2, lacks the N-terminal domain necessary for interaction with unfoldases such as ClpX or ClpC. To validate this hypothesis biochemically, we expressed and purified a truncated variant of *Pp*ClpX (residues 65–414, lacking the unstructured N-terminal region, with a molecular weight of approximately 43 kDa). AUC analysis confirmed that the truncated *Pp*ClpX protein assembles into a Homohexamer (Fig 5A). Subsequently, pull-down assays demonstrated that whereas *Pp*ClpP2 interacts with *Pp*ClpX, no interaction was detected between *Pp*ClpP1 and *Pp*ClpX (Fig 5B and 5C), thus confirming our structural prediction. Furthermore, we verified that the heteromeric *Pp*ClpP1P2 complex retains the ability to interact with *Pp*ClpX (Fig 5D). The interaction is a prerequisite for forming the active Clp proteasome, in which the unfoldase recognizes specific substrates (such as ssrA-tagged proteins) and translocates them into the degradation chamber of ClpPs for hydrolysis.

We further evaluated the proteolytic activity of the *Pp*ClpX (full-length) in complex with different *Pp*ClpP assemblies (ClpP1, ClpP2, or the ClpP1P2 heterocomplex) using two model substrates: GFP-ssrA and FITC-casein. In the absence of *Pp*ClpX, neither *Pp*ClpP1, *Pp*ClpP2, *Pp*ClpX, nor *Pp*ClpP1P2 alone degraded GFP-ssrA or FITC-casein (Fig 5E and 5F). In contrast, both the *Pp*ClpX + *Pp*ClpP2 and *Pp*ClpX + *Pp*ClpP1P2 complexes efficiently degraded these substrates, indicating that *Pp*ClpX functions specifically through recognition of *Pp*ClpP2 to activate proteolysis. Notably, the proteolytic activity of *Pp*ClpX + *Pp*ClpP1P2 was slightly higher than that of *Pp*ClpX + *Pp*ClpP2, suggesting that the heterocomplex *Pp*ClpP1P2 possess enhanced hydrolytic capability compared to *Pp*ClpP2 alone.

### 3.7. Bortezomib inhibits *Pp*ClpP1 peptidase activity

**Fig 6:**
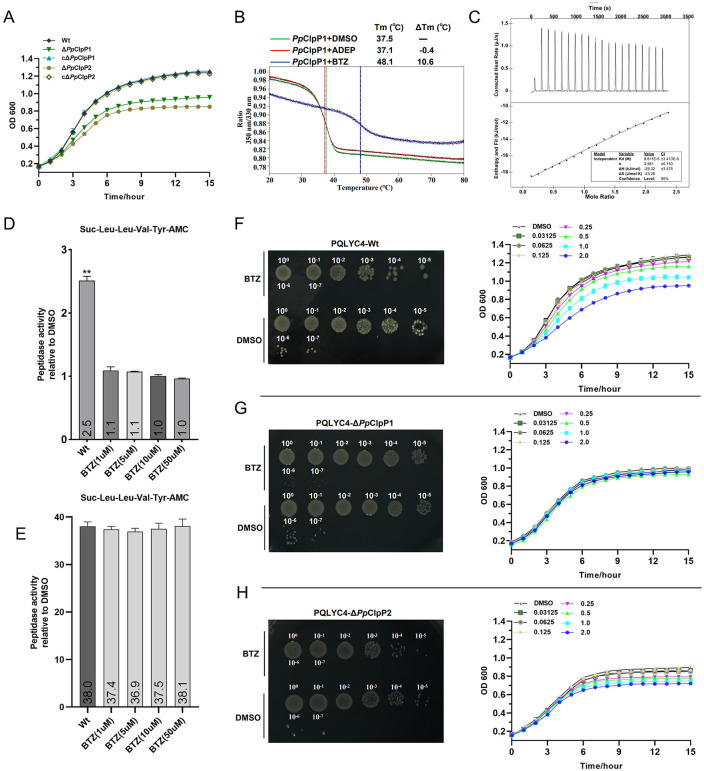
Characterization of BTZ-mediated inhibition of *Pp*ClpP1 and its antibacterial effects. **(A)** Determination of the growth curves of the wild-type *P. plecoglossicida* PQLYC4, Δ*Pp*ClpP1, Δ*Pp*ClpP2, and complemented strains (designated as cΔ*Pp*ClpP1and cΔ*Pp*ClpP2). **(B)** Differential scanning fluorimetry (DSF) analysis of *Pp*ClpP1 in the presence of small molecules (ADEP and BTZ). **(C)** Isothermal titration calorimetry (ITC) binding isotherm for BTZ (300 μM) titrated into *Pp*ClpP1 (30 μM), with derived binding parameters (Kd = 98 ± 3.41 μM). **(D)** Effect of BTZ on the peptidase activity of *Pp*ClpP1. **(E)** Effect of BTZ on the peptidase activity of *Pp*ClpP2. BTZ sensitivity assays of wild-type *P. plecoglossicida* PQLYC4 **(F)**, Δ*Pp*ClpP1 **(G)**, and Δ*Pp*ClpP2 mutant strains **(H)**. Left, the bacterial cultures were adjusted to OD_600_ ≈ 0.4 with double distilled water, and 10-fold serial dilution was performed on TSB (with 1.25 μM BTZ), and plated on TSB agar to determine the number of surviving colony forming bacteria. Right, when freshly cultured bacteria grew to an OD_600_ value of 0.1, add BTZ small molecules at different concentrations (μM), spectrophotometry was used to measure the OD_600_ value at appropriate intervals. Bars represent the means of three independent experiments ± SD. All experiments were performed in triplicate.

Our biochemical assays confirmed that both *Pp*ClpP1 and *Pp*ClpP2 possess peptidase activity, indicating their significant roles in bacterial physiology. We constructed knockout mutants of *P. plecoglossicida* PQLYC4 by deleting the *clpP1* and *clpP2* genes individually (S5C and S5D Fig). Deletion of either gene resulted in a significant growth defect compared to the wild-type strain. Notably, the growth impairment observed in the Δ*Pp*ClpP2 mutant was more severe than that in the Δ*Pp*ClpP1 mutant (Fig 6A). And a significant deficiency in both swimming and swarming motilities were observed in the Δ*Pp*ClpP1 and Δ*Pp*ClpP2 strains (S5E and S5F Fig). These findings demonstrate that both *Pp*ClpP1 and *Pp*ClpP2 are indispensable for the normal growth and bacterial motility of *P. plecoglossicida*.

Numerous studies have demonstrated that various small molecules can modulate ClpP protease activity, either activating or inhibiting its function [18–20,43]. In our systematic screening of *Pp*ClpP modulators for structural studies, we evaluated two chemically distinct compounds: the natural product acyldepsipeptide (ADEP) [44,45], and the proteasome inhibitor bortezomib (BTZ) [46], both previously identified as ClpP complex modulators and proposed as potential antimicrobial candidates. Differential scanning fluorimetry (DSF) revealed that BTZ induced significant thermal stabilization of *Pp*ClpP1 (ΔTm = +10.6°C, Fig 6B), while ADEP showed no stabilizing effect. Isothermal titration calorimetry (ITC) confirmed direct BTZ-*Pp*ClpP1 binding with moderate affinity (Kd = 98 ± 3.41 μM, Fig 6C). BTZ, an N-protected dipeptide boronic acid that forms covalent adducts with catalytic serines or threonines, demonstrated potent inhibition of *Pp*ClpP1 peptidase activity. At 1 μM concentration, the BTZ completely abolished the Suc-LLVY-AMC hydrolysis (Fig 6D). In contrast, both DSF (S5G Fig) and ITC (S5H Fig) analyses revealed that BTZ does not interact with *Pp*ClpP2, and its addition did not affect the peptidase activity of *Pp*ClpP2 (Fig 6E). Notably, BTZ exhibited opposing effects on the peptidase activity of different ClpP homologs: it functioned as an activator for *Tt*ClpP [20] but as an inhibitor for *Pp*ClpP1.

Given that BTZ inhibits the enzymatic activity of *Pp*ClpP1, we investigated its direct impact on bacterial growth. Bacterial plate titration assays revealed that BTZ (at 1 μM) significantly suppressed the growth of *P. plecoglossicida* (Fig 6F-left). Consistent with this, bacterial growth curve analysis demonstrated that BTZ inhibits bacterial growth in a concentration-dependent manner (Fig 6F-right). To ascertain the specificity of BTZ, we tested its effect on isogenic knockout mutants. BTZ failed to inhibit the growth of the Δ*Pp*ClpP1 mutant (Fig 6G), whereas it retained a potent bacteriostatic effect on the Δ*Pp*ClpP2 mutant (Fig 6H). Collectively, these results indicated that the antibacterial activity of BTZ against *P. plecoglossicida* is specifically mediated through targeting *Pp*ClpP1, and that BTZ inhibits bacterial growth by inhibiting the peptidase activity of *Pp*ClpP1.

### 3.8. Binding mode of bortezomib to *Pp*ClpP1

**Fig 7:**
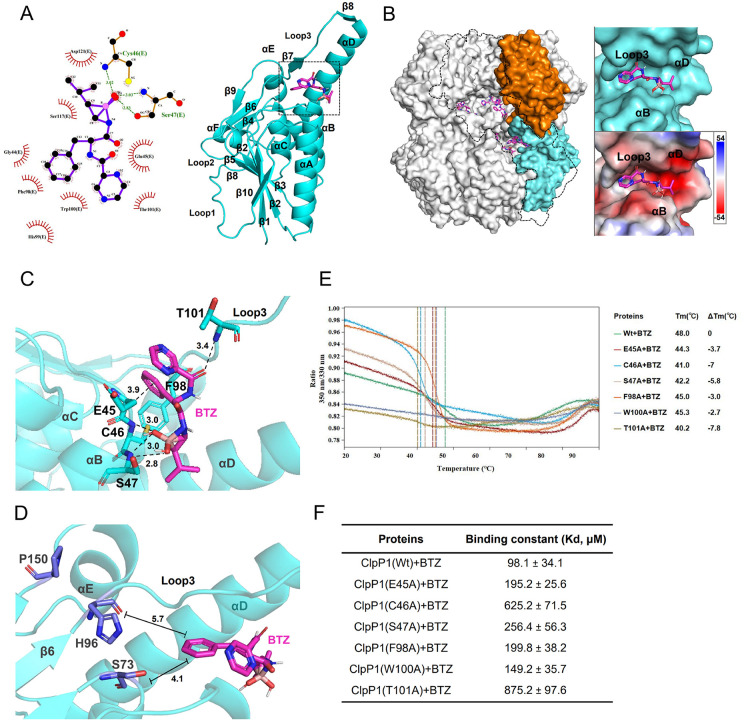
Elucidation of the BTZ binding mode within *Pp*ClpP1. **(A)** 2D-molecular docking (left) and 3D-molecular docking (right) between BTZ and the *Pp*ClpP1 monomer. **(B)** The surface structure diagram of the *Pp*ClpP1 tetradecamer, with orange and cyan representing the *Pp*ClpP1 monomers in the two heptameric rings, respectively. The black dashed lines outline the removed two *Pp*ClpP1 monomers (left). Depiction of the hydrophobic binding pocket that BTZ binds in the context of the *Pp*ClpP1 monomer. Red sticks are the small molecule BTZ. **(C)** Substrate-binding pocket of *Pp*ClpP1, the residues involved in the binding to the BTZ are shown as sticks. **(D)** Enlarged view of the catalytic triad and BTZ in the *Pp*ClpP1 monomer. The black measurement lines represent the average distances between residues S73/H96 and the benzene ring on BTZ, which are 4.1 Å and 5.7 Å respectively. **(E)** Differential scanning fluorimetry (DSF) analysis of various *Pp*ClpP1 mutant proteins with BTZ. The ΔTm value represents the temperature difference between the melting temperature (Tm) of the *Pp*ClpP1 mutant + BTZ and that of the wild-type *Pp*ClpP1 + BTZ. **(F)** The binding affinity of BTZ to wild-type *Pp*ClpP1 and its mutants was quantified by isothermal titration calorimetry (ITC). The derived Kd values are shown within. Results are representative of those from three independent experiments. Data represent mean values ± SD.

To elucidate the structural mechanism of BTZ-mediated inhibition, we performed molecular docking simulations with the *Pp*ClpP1 monomer, which identified a cluster of key potential interacting residues (G44, E45, C46, S47, F98, H99, W100, T101, S117, and D121, Fig 7A-left). Initial simulations positioned BTZ within a hydrophobic pocket formed by loop 3 and helices B/D of individual subunits (Fig 7A-right). However, complete occupancy of the tetradecameric lumen prevented clear visualization of ligand-binding conformations. Selective removal of two opposing monomers revealed BTZ distributed along the proteolytic chamber axis, with predominant binding at the loop 3 and helix B/D interface (Fig 7B). High-resolution docking models identified three key interaction networks (Fig 7C): (1) Hydrogen bonds between BTZ’s boronic acid group and Cys46/Ser47; (2) Hydrogen bonds between BTZ’s pyrazine ring and Thr101; (3) Hydrophobic interactions between BTZ’s phenyl group and Glu45/Phe98. Notably, BTZ’s phenyl ring occupies a position 4.1 Å from catalytic Ser73 and 5.7 Å from His96－ sufficiently proximal to sterically hinder substrate access yet distant from direct catalytic triad engagement (Fig 7D). To delineate the precise contribution of specific amino acids to BTZ binding, we generated a series of alanine-scanning mutants: *Pp*ClpP1 (G44A, E45A, C46A, S47A, F98A, H99A, W100A, T101A, S117A, and D121A). Subsequent analysis by DSF (Fig 7E) and ITC (Figs 7F and S6A–S6F) assays confirmed that several of these residues are critical for BTZ binding. Specifically, mutations of E45, C46, S47, F98, W100, and T101 to alanine significantly diminished binding affinity, highlighting their importance for BTZ binding.

### 3.9. Comparative analysis of ligand-ClpP binding modes across species

**Fig 8:**
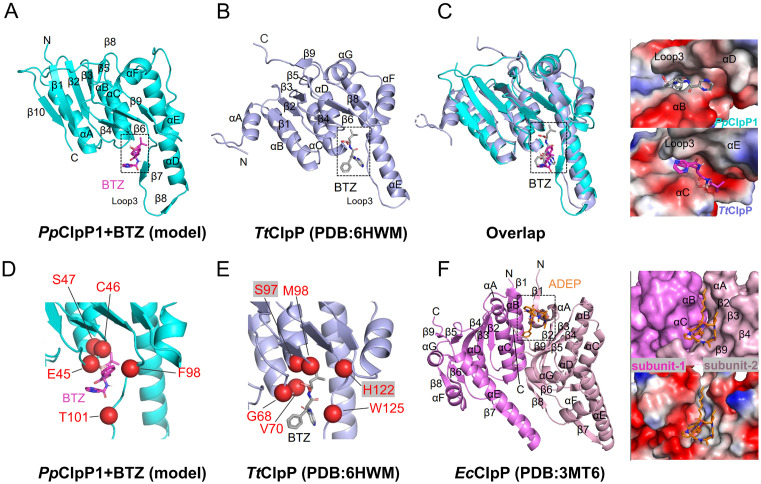
Comparative analysis of ligand binding modes across ClpP homologs. **(A)** Cartoon representation of the *Pp*ClpP1 monomer in BTZ-bound (marked red) and **(B)** *Tt*ClpP BTZ-bound (marked gray) states. **(C)** Superposition of BTZ-bound *Pp*ClpP (cyan) and *Tt*ClpP (light blue) reveals distinct binding geometries (left), with electrostatic potential maps illustrating differential interaction patterns (right). Detailed views of binding interfaces demonstrate residue-specific contacts (red spheres) in *Pp*ClpP1 **(D)** versus *Tt*ClpP **(E)**. **(F)** Comparative analysis includes ADEP-bound (orange) *Ec*ClpP dimer, showing inter-subunit binding (violet/light pink subunits) with surface and electrostatic potential representations (right) that contrast sharply with BTZ’s monomer-restricted binding mode.

The structural uniqueness of *Pp*ClpP1 prompted a detailed comparison of ligand-binding mechanisms across species. Structural alignment of our BTZ-bound *Pp*ClpP1 model (Fig 8A) with the *Tt*ClpP-BTZ complex (Fig 8B) revealed both conserved and divergent features. While BTZ occupies similar positions within the hydrophobic vestibule of both proteases, its orientation differs significantly - the boronic acid moiety faces loop3 in *Pp*ClpP1 but projects outward in *Tt*ClpP (Fig 8C). More importantly, the key residues mediating BTZ binding are completely distinct between *Pp*ClpP1 and *Tt*ClpP (Figs 8D, 8E and S7A). In *Tt*ClpP, BTZ directly engages catalytic triad residues S97 (through hydrogen bonding) and H122 (via π-cation interaction), thereby stabilizing the charge-relay system and enhancing proteolytic activity (Fig 8E) [20]. The distinct binding mode of BTZ to *Pp*ClpP1, compared to *Tt*ClpP, arises from structural differences in the active site. *Tt*ClpP possesses a cavity (S7B Fig) that enables the boronic acid group of BTZ to extend toward and interact with the catalytic residues S97 and H122 (S7C Fig). Conversely, the absence of this cavity in *Pp*ClpP1 creates steric hindrance (S7D Fig), preventing the boronic acid group from positioning itself near the catalytic triad (S7E Fig). This binding mode of BTZ fails to stabilize the catalytic triad of *Pp*ClpP1. Instead, interactions with residues surrounding the triad may alter its conformation, resulting in reduced or complete loss of enzymatic activity (S7F Fig).

ADEPs represent another important class of ClpP modulators with fundamentally different binding topologies. Structural studies of *E. coli* ClpP (*Ec*ClpP) [18] show that ADEPs occupy an inter-subunit hydrophobic cleft formed by α-helices B-C of one monomer and α-helices A, β-strands 2–4/9 of an adjacent monomer (Figs 8F and S7G), a binding mode conserved in *Bs*ClpP and *Mtb*ClpP1 (S7H Fig and S7I). This dual-subunit engagement contrasts sharply with BTZ’s monomer-restricted binding and drives distinct antimicrobial mechanisms. ADEP binding induces allosteric pore dilation through N-terminal helix displacement, simultaneously activating uncontrolled proteolysis while blocking Clp-ATPase docking. In contrast, BTZ directly modulates catalytic triad conformation, bypassing ATPase-mediated regulation. These differential binding modes define two distinct antibacterial strategies: ADEP-mediated proteostasis dysregulation versus BTZ-driven direct enzymatic modulation.

## 4. Discussion

The Clp protease systems constitute essential cytosolic proteolytic complexes in bacteria, orchestrating pivotal physiological processes including protein homeostasis maintenance, stress response regulation, and virulence factor modulation. Mounting evidence positions ClpP as a promising therapeutic target against multidrug-resistant bacterial pathogens, with recent pharmacological studies demonstrating its druggability in both Gram-positive and Gram-negative species [47,48]. Consequently, the development of ClpP-specific small-molecules disrupting proteolytic activity has emerged as an innovative antibacterial strategy, offering potential to circumvent conventional antibiotic resistance mechanisms [3,4]. *P*. *plecoglossicida* is an important pathogen for aquaculture and causes high mortality in various marine fishes. Despite the therapeutic potential of targeting its Clp protease system, the structural and functional features of its two key paralogs, *Pp*ClpP1 and *Pp*ClpP2, are not fully resolved. In this work, we sought to provide a detailed spatial and functional understanding of ClpP from *P*. *plecoglossicida* to pursue the design of therapeutic molecules and to better understand the biological function of the *Pseudomonas* Clp system.

The *Pp*ClpP1 protease emerged as a phylogenetically distinct entity, characterized by an unusually truncated N-terminal domain and a putative non-canonical catalytic triad (Ser-His-Pro). *Pp*ClpP1 was substantially less active than *Pp*ClpP2 in peptidase assays, suggesting underlying structural divergence. While canonical ClpP proteases universally employed a conserved Ser-His-Asp catalytic triad, certain bacterial homologs (e.g., ClpP1 in *Listeria monocytogenes*) exhibited rare Ser-His-Asn configurations [37]. Site-directed mutagenesis of the catalytic triad indicated that the serine and histidine residues perform an indispensable catalytic function in both *Pp*ClpP1 and *Pp*ClpP2. However, the identity of the third residue (proline) within the triad was not the primary determinant for the lower catalytic efficiency of *Pp*ClpP1, suggesting that other intrinsic structural or electronic factors were responsible for the observed activity difference. The cryo-EM structure of *Pp*ClpP1 revealed a homotetradecamer with a unique Ser-His-Pro catalytic triad, a novel configuration among ClpP proteases. Comparison with other ClpP structures indicated conserved geometry for the serine and histidine, but a marked divergence in the position of the proline residue.

*Pp*ClpP1 exhibited unique architectural divergence through its terminal domain organization. Unlike canonical ClpP proteases that utilized an N-terminal axial loop for ClpX unfoldase recognition via pore-2 loop interactions [39,40,49], *Pp*ClpP1 completely lacked this critical interaction module. Altogether, the N-terminal domains of *Pp*ClpP1 were consistent with the structures of *Pa*ClpP2 [42], lacked the structure involved in ClpX binding. The C-terminal mini-helix was atypical but not unprecedented observation. For *Pp*ClpP1, a C-terminal mini-helix was also lacking. These terminal truncations correlated with distinctive axial pore geometry. *Pp*ClpP1 heptamers maintained an expanded axial pore diameter of 42.7 Å, exceeding all structurally characterized homologs. The increase in lumen width suggested compromised substrate sequestration efficiency, potentially leading to reduce dwell time of substrate peptides in widened pores. The observed attenuation of proteolytic activity for *Pp*ClpP1 likely stems from this structural relaxation.

In many bacteria, *clpP1* and *clpP2* genes resided at distinct genomic loci and encode paralogs with divergent *in vivo* functions [13–16,42]. A notable example was *P. aeruginosa*, in which *clpP1* and *clpP2* occupied separate genomic locations, with *clpP1* co-localized with *clpX* in an operon [42]. This genomic organization underpinned a functional hierarchy: *Pa*ClpX specifically activated and interacted with *Pa*ClpP1, which can assemble into homotetradecamers or heterotetradecamers with *Pa*ClpP2. Similarly, in *P. plecoglossicida*, *Pp*ClpP2 was co-transcribed with *Pp*ClpX in an operon, while *Pp*ClpP1 was located at separate genomic loci.

The AlphaFold-predicted structure of the *Pp*ClpP2 homotetradecamer revealed conserved N-terminal axial and C-terminal mini-loop motifs, which shared strong architectural similarity with the ClpX recognition regions of *Pa*ClpP1 [42]. This structural complementarity was functionally validated by pull-down assays, which demonstrated specific binding between *Pp*ClpX and *Pp*ClpP2, but not *Pp*ClpP1. Functionally, the assembly of the *Pp*ClpP1P2 heterotetradecamer significantly enhanced peptidase activity, and the resulting *Pp*ClpP1P2–*Pp*ClpX complex exhibited superior hydrolytic capacity compared to the *Pp*ClpP2–*Pp*ClpX. Based on these structural and biochemical observations, we proposed a model in which *Pp*ClpP1 adopts a latent, low-activity conformation in its homotetradecameric state. Integration into the heterocomplex with *Pp*ClpP2 likely induce conformational rearrangement, leading to allosteric activation and a synergistic enhancement of proteolytic function in the assembled protease machinery.

Small molecules have emerged as important modulators of ClpP protease activity, functioning either as activators or inhibitors. ADEPs, for instance, induced profound functional reprogramming of the ClpP complex by disrupting its interaction with AAA+ unfoldase partners. This leaded to uncontrolled proteolysis of unfolded substrates and accumulation of toxic proteins in various bacterial species including *E. coli*, *M. tuberculosis*, and *B. subtilis* [19,43,50]. In contrast, certain activators such as BTZ and N-blocked peptide aldehydes bypassed allosteric regulation mechanisms and bind directly to the protease active site [20,43,48]. However, their effects exhibited significant species specificity. While BTZ strongly inhibited *Pp*ClpP1 at 1 μM concentration, it showed no effect on *Pp*ClpP2. Conversely, BTZ activated *Tt*ClpP at 12.5 μM [20], highlighting the functional divergence among ClpP homologs. The structural basis for this differential response lies in distinct active site architectures. *Tt*ClpP possessed a characteristic cavity that accommodates the boronic acid group of BTZ, enabling productive interactions with catalytic residues S97 and H122. In *Pp*ClpP1, however, the absence of this cavity created steric constraints that prevent proper positioning of the boronic acid moiety near the catalytic triad. This structural variation explained why BTZ cannot adopt the same binding conformation in *Pp*ClpP1 as observed in the *Tt*ClpP-BTZ complex.

## 5. Conclusion

**Fig 9:**
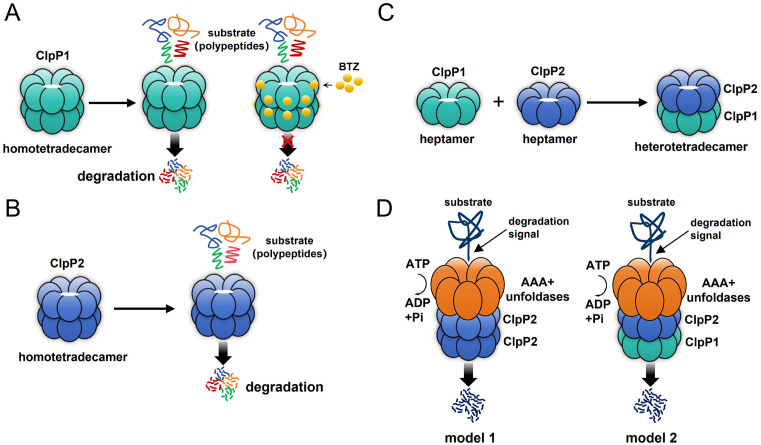
Assembly model of ClpP family proteins in *P*. *plecoglossicida.* **(A)** The ClpP1 assembles in a homotetradecameric form and is capable of exhibiting low peptidase activity. However, BTZ can bind to ClpP1 and inhibit its peptidase activity. **(B)** The ClpP2 assembles in a homotetradecameric form and is capable of exhibiting high peptidase activity. **(C)** The formation of a *P*. *plecoglossicida* ClpP1P2 heterotetradecamer. **(D)** Potential assembly mode of the *P*. *plecoglossicida* ClpP protein complex with AAA+ unfoldases. The assembly mode of the ClpP2 homotetradecamer and AAA+ unfoldases complex (model 1), the assembly mode of the ClpP1P2 heterotetradecamer and AAA+ unfoldases complex (model 2).

This study comprehensively characterized two distinct ClpP protease paralogs, *Pp*ClpP1 and *Pp*ClpP2, in *P. plecoglossicida* PQLYC4. Phylogenetic and sequence analyses revealed that *Pp*ClpP2 is a canonical ClpP protease, while *Pp*ClpP1 is evolutionarily divergent, featuring an N-terminal truncation and a novel catalytic triad configuration (Ser-His-Pro) instead of the conserved Ser-His-Asp. Biochemically, *Pp*ClpP2 exhibited significantly higher (~13-fold) peptidase activity than *Pp*ClpP1. Mutagenesis confirmed the serine and histidine in the triad are indispensable for both proteases, but the proline in *Pp*ClpP1 (or aspartate in *Pp*ClpP2) is not absolutely essential. The cryo-EM structure of *Pp*ClpP1 confirmed its homotetradecameric assembly and the unprecedented Ser-His-Pro catalytic triad. The N-terminal truncation resulted in a dramatically expanded axial pore. AlphaFold modeling of *Pp*ClpP2 indicated a narrower pore and a canonical Ser-His-Asp triad. The two paralogs could form a stable heterotetradecamer (*Pp*ClpP1P2) with proteolytic activity higher than either homotetradecamer alone. Crucially, only *Pp*ClpP2, which possesses the N-terminal domain, interacted with the unfoldase *Pp*ClpX. The *Pp*ClpP1P2 heterotetradecamer also interacted with *Pp*ClpX and, when assembled with it, showed enhanced protein degradation activity. Furthermore, the proteasome inhibitor BTZ specifically inhibited *Pp*ClpP1’s peptidase activity by binding to a unique pocket without directly engaging the catalytic serine, but it did not affect *Pp*ClpP2. This inhibition was mediated through interactions with specific residues (e.g., E45, C46) surrounding the active site. BTZ’s antibacterial effect on *P. plecoglossicida* was specifically mediated through targeting *Pp*ClpP1, as it inhibited wild-type and Δ*Pp*ClpP2 strains but not the Δ*Pp*ClpP1 mutant. Molecular docking revealed a binding mode for BTZ in *Pp*ClpP1 distinct from its activating role in other ClpPs like *T. tengcongensis* ClpP, primarily due to structural differences in the active site cavity. In conclusion, the research delineates fundamental structural and functional distinctions between the two ClpP paralogs in *P. plecoglossicida*, reveals their capacity to form a more active heterocomplex, and identifies *Pp*ClpP1 as a unique antibacterial target for BTZ. As illustrated in Fig 9, we propose some models summarizing the distinct functions and assembly mechanisms of *Pp*ClpP1 and *Pp*ClpP2 in *P. plecoglossicida*.

## Supporting information

S1 Fig Sequence conservation, gene expression, and oligomeric state analysis of *Pp*ClpP1 and *Pp*ClpP2. (A) Multiple sequence alignment of homologous ClpP proteins was created using the ESPript 3.0 server Aligned Sequences tool. The catalytic triad residues (Ser, His) are marked with black asterisks, the residues (Pro, Asn or Asp) are marked with red asterisk. (B) Relative mRNA expression levels of *Pp*ClpP1 and *Pp*ClpP2 at different growth phases of *P. plecoglossicida* were determined by real-time qPCR. (C, D) Sedimentation velocity analytical ultracentrifugation (AUC) analysis of the homotetradecameric states of *Pp*ClpP1 and *Pp*ClpP2.
(TIF)

S2 Fig Cryo-EM data processing workflow and structural determination of *Pp*ClpP1. (A) Schematic summary of *Pp*ClpP1 Cryo-EM data processing pipeline in CryoSparc. (B) Representative micrograph after motion correction and selection. (C) Representative 2D class averages. (D) FSC curves for the final reconstruction with reported resolution at FSC = 0.143 shown by the blue horizontal line. (E) Cartoon representation of the *Pp*ClpP1 tetradecamer. (F) The cryo-EM density map for the *Pp*ClpP1 monomeric unit.
(TIF)

S3 Fig Oligomeric structure and assembly interfaces of the *Pp*ClpP1 tetradecamer. (A) Structure of the tetradecameric *Pp*ClpP1 asymmetric unit illustrated as ribbon drawing. (B) The *Pp*ClpP1 tetradecamerization interface. (a) Oligomerization of heptamers into tetradecamers involves the handle domains of monomers from opposing heptameric rings interdigitating as shown in dashed boxes. The magnified views of the binding interface are shown in the (b) and (c), in which the potential interacting residues are represented by a stick model. (C) The *Pp*ClpP1 heptamerization interface. (a) Oligomerization into heptamers entails aligned α-helices of one subunit interfacing with the aligned β-sheets and disorder of another shown in green. The magnified views of the binding interface are shown in the (b) and (c) and (d), in which the potential interacting residues are represented by a stick model.
(TIF)

S4 Fig Comparative structural analysis of conserved ClpP tetradecamer architecture. (A) Side view of conserved ClpP tetradecameric architecture showing dimensional measurements of heptameric ring diameters and axial pore sizes across homologs. (B) Structural alignment of catalytic triads from *Pp*ClpP1, *Pa*ClpP1, and *Pa*ClpP2, with catalytic residues (Ser-His-Asp/Pro) represented as stick models.
(TIF)

S5 Fig Distinct properties of *Pp*ClpP paralogs revealed by structural prediction, genetic knockout, and phenotypic assays. (A) AlphaFold-predicted tetradecameric structure of *Pp*ClpP2 shown as ribbon diagram, demonstrating conserved oligomeric architecture. The modeled structures were supported by high prediction confidence scores, with an ipTM of 0.87, a pTM of 0.88, and a plDDT value exceeding 90. (B) AUC analysis revealed that the molecular weight of the *Pp*ClpP1P2 complex is approximately 300 kDa, which is consistent with the theoretical molecular weight of a tetradecameric complex (20 × 7 + 23 × 7 = 301 kDa). (C) Genotype confirmation of the knockout mutant strain. Confirmation of gene knockout by PCR with pairs of primers designed to target outside of the deletion domain. Lane M: DNA marker (DL2000); Lane 1: The 1552 bp fragment amplified from genomic DNA of wild-type *P. plecoglossicida* with primer set 18TcΔ*Pp*ClpP1-U F/R. Lane 2: The 552 bp fragment amplified from *P. plecoglossicida* strain with primer *Pp*ClpP1-F/R. Lane 3: The 1000 bp fragment amplified from Δ*Pp*ClpP1 strain with primer set Δ*Pp*ClpP1-U F/R. Lane 4: The 0 bp fragment amplified from Δ*Pp*ClpP1 strain with primer set *Pp*ClpP1-F/R. (D) Lane 1: The 1642 bp fragment amplified from genomic DNA of wild-type *P. plecoglossicida* with primer set 18TcΔ*Pp*ClpP2-U F/R. Lane 2: The 642 bp fragment amplified from *P. plecoglossicida* strain with primer *Pp*ClpP2-F/R. Lane 3: The 1000 bp fragment amplified from Δ*Pp*ClpP2 strain with primer set Δ*Pp*ClpP2-U F/R. Lane 4: The 0 bp fragment amplified from Δ*Pp*ClpP2 strain with primer set *Pp*ClpP2-F/R. (E-F) Swimming and swarming motility of *Pseudomonas plecoglossicida* wild-type and mutant strains and complemented strains. (G) DSF analysis revealed no significant thermal shift in *Pp*ClpP2 upon the addition of BTZ, suggesting a lack of direct interaction between them. (H) ITC binding isotherm for BTZ (300 μM) titrated into *Pp*ClpP2 (30 μM), the resulting binding isotherm indicated no observable interaction between the two molecules.
(TIF)

S6 Fig The original results of the binding affinity of ClpP1 mutants to BTZ. Isothermal titration calorimetry (ITC) binding isotherm for BTZ (300 μM) titrated into *Pp*ClpP1 mutants (30 μM), with derived binding parameters: (A) ClpP1 (E45A) + BTZ, Kd = 195.2 ± 25.6 μM; (B) ClpP1 (C46A) + BTZ, Kd = 625.2 ± 71.5; (C) ClpP1 (S47A) + BTZ, Kd = 256.4 ± 56.3; (D) ClpP1 (F98A) + BTZ, Kd = 199.8 ± 38.2; (E) ClpP1 (W100A) + BTZ, Kd = 149.2 ± 35.7; (F) ClpP1 (T101A) + BTZ, Kd = 875.2 ± 97.6. These were the original data for results summarized in Fig 7F. The binding affinity are also shown within. Data represent mean values ± s.d.
(TIF)

S7 Fig Comparative structural analysis of small-molecule binding sites in *Pp*ClpP1 and other bacterial ClpP proteases. (A) Multiple sequence alignment of *Pp*ClpP1 and *Tt*ClpP, the alignment was created using the ESPript 3.0 server Aligned Sequences tool. Amino acids have been colored with similarity coloring scheme % MultAlin, Global score 0.7. Residues for the binding of *Pp*ClpP1 and *Tt*ClpP to bortezomib are highlighted in blue and green, respectively. (B) Structure of the *Tt*ClpP (PDB: 6HWN) monomer in the apo state. Left, cartoon diagram. Right, surface diagram, the region where BTZ binds forms a deep cavity, with residues S97 and H122 located near its base. (C) Structure of the *Tt*ClpP (PDB: 6HWM) monomer in complex with BTZ. Left, cartoon diagram. Right, surface diagram, the boronic acid group of BTZ projects into the binding cavity, forming interactions with S97 and H122. (D) Structure of the *Pp*ClpP1 monomer. Left, cartoon diagram. Right, surface diagram, the BTZ-binding site in *Pp*ClpP1 forms a distinct groove. (E) Predicted structural model of the *Pp*ClpP1 monomer in complex with BTZ. Left, cartoon diagram. Right, surface diagram. BTZ is predicted to bind within this groove. (F-I) Comparative structural analysis reveals distinct binding modes of small-molecule modulators across bacterial species: (F) *Pp*ClpP1-BTZ complex showing intra-subunit binding; (G) *E. coli* ClpP (*Ec*ClpP) in complex with ADEP1; (H) *B. subtilis* ClpP (*Bs*ClpP) in complex with ADEP2; and (I) *M. tuberculosis* ClpP1/2 (*Mtb*ClpP1/2) in complex with ADEP, demonstrating conserved inter-subunit binding pockets for acyldepsipeptides (ADEPs) that contrast with BTZ’s unique binding topology in *Pp*ClpP1.
(TIF)

S1 Table Oligonucleotide primers used in this study. (DOCX)

S2 Table Cryo-EM statistics and model refinement of *Pp*ClpP1 tetradecamer. (DOCX)

S1 File Minimal data set. (ZIP)
