# Supplementary figures and images for: Structural and mechanistic insights into caseinolytic protease inhibition for antimicrobial development against Pseudomonas plecoglossicida (part 1 of 2)
Source: PLoS Pathog. 2026 Feb 12;22(2):e1013909. doi: 10.1371/journal.ppat.1013909 (PMC12900304; doi:10.1371/journal.ppat.1013909)

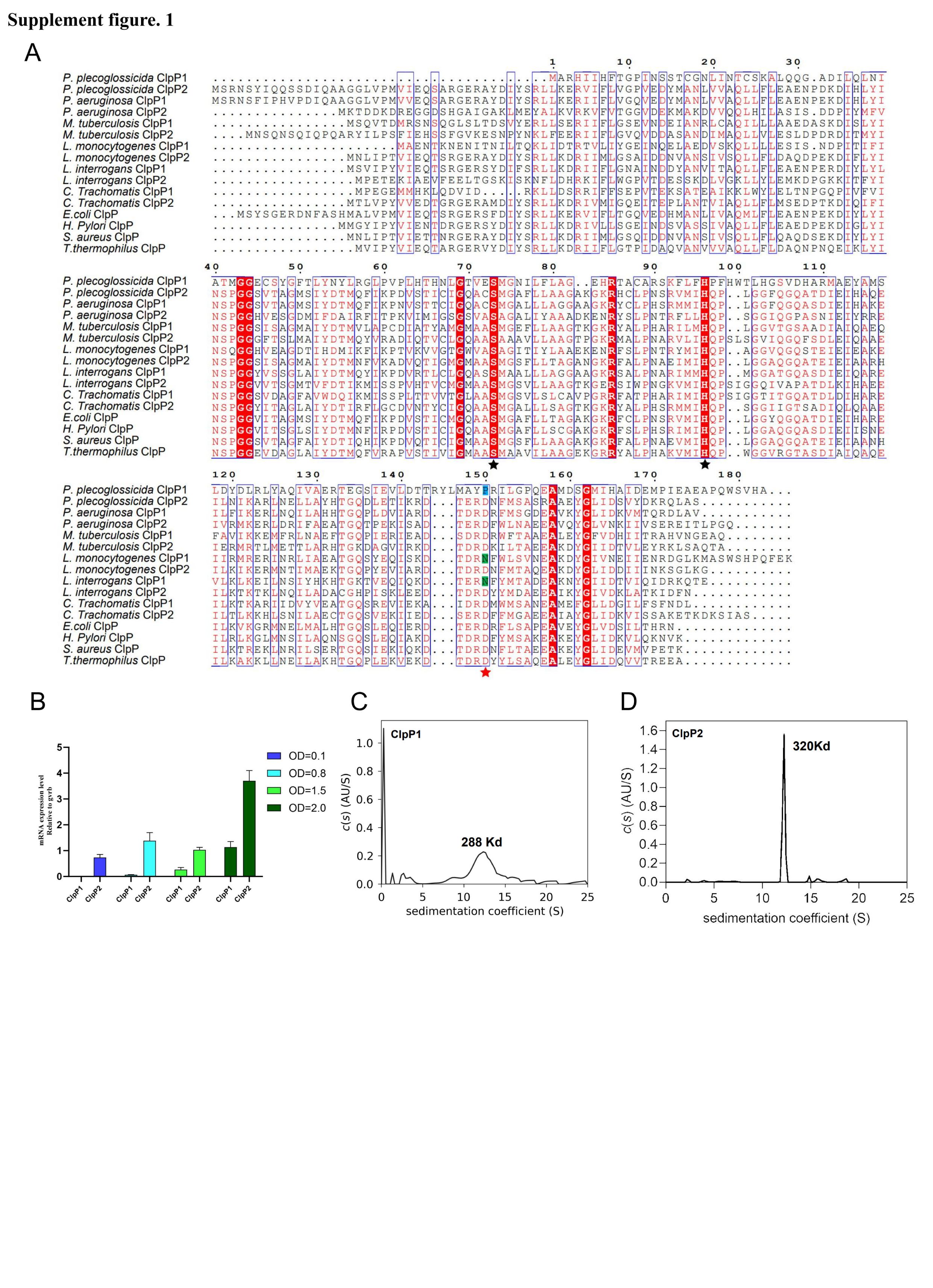

Supplement: S1 Fig — (A) Multiple sequence alignment of homologous ClpP proteins was created using the ESPript 3.0 server Aligned Sequences tool. The catalytic triad residues (Ser, His) are marked with black asterisks, the residues (Pro, Asn or Asp) are marked with red asterisk. (B) Relative mRNA expression levels of PpClpP1 and PpClpP2 at different growth phases of P. plecoglossicida were determined by real-time qPCR. (C, D) Sedimentation velocity analytical ultracentrifugation (AUC) analysis of the homotetradecameric states of PpClpP1 and PpClpP2. (TIF) [file ppat.1013909.s001.tif]

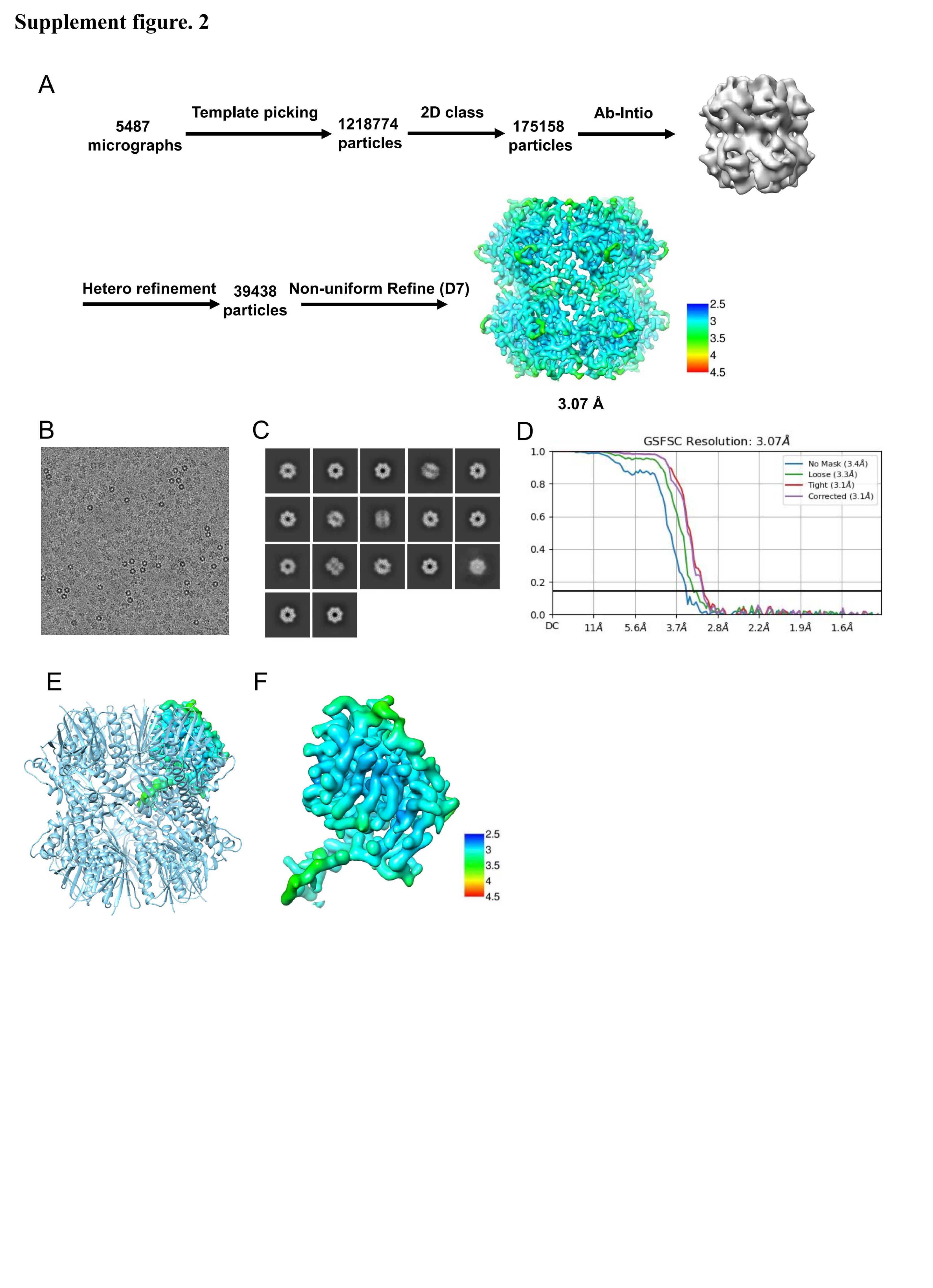

Supplement: S2 Fig — (A) Schematic summary of PpClpP1 Cryo-EM data processing pipeline in CryoSparc. (B) Representative micrograph after motion correction and selection. (C) Representative 2D class averages. (D) FSC curves for the final reconstruction with reported resolution at FSC = 0.143 shown by the blue horizontal line. (E) Cartoon representation of the PpClpP1 tetradecamer. (F) The cryo-EM density map for the PpClpP1 monomeric unit. (TIF) [file ppat.1013909.s002.tif]

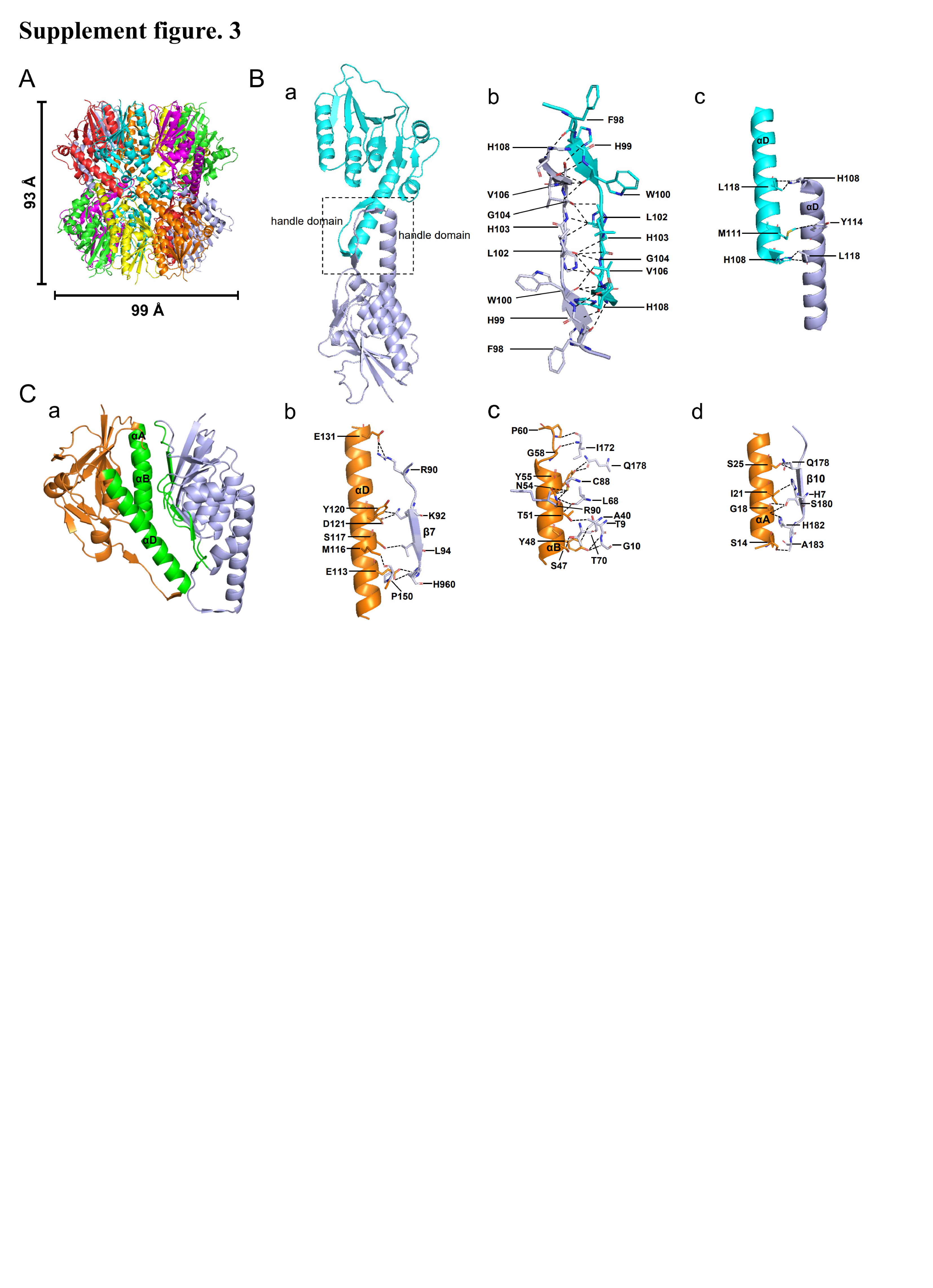

Supplement: S3 Fig — (A) Structure of the tetradecameric PpClpP1 asymmetric unit illustrated as ribbon drawing. (B) The PpClpP1 tetradecamerization interface. (a) Oligomerization of heptamers into tetradecamers involves the handle domains of monomers from opposing heptameric rings interdigitating as shown in dashed boxes. The magnified views of the binding interface are shown in the (b) and (c), in which the potential interacting residues are represented by a stick model. (C) The PpClpP1 heptamerization interface. (a) Oligomerization into heptamers entails aligned α-helices of one subunit interfacing with the aligned β-sheets and disorder of another shown in green. The magnified views of the binding interface are shown in the (b) and (c) and (d), in which the potential interacting residues are represented by a stick model. (TIF) [file ppat.1013909.s003.tif]

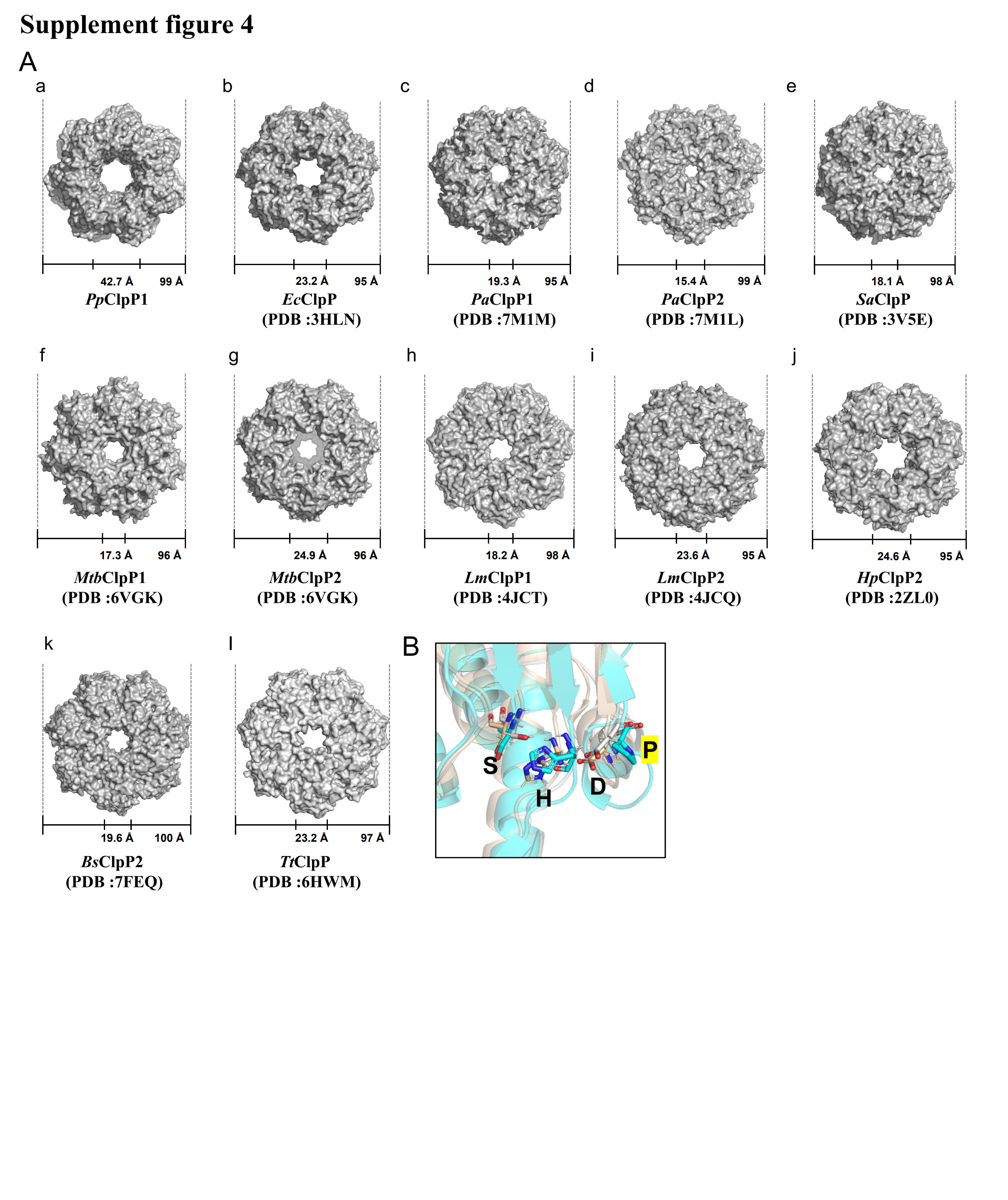

Supplement: S4 Fig — (A) Side view of conserved ClpP tetradecameric architecture showing dimensional measurements of heptameric ring diameters and axial pore sizes across homologs. (B) Structural alignment of catalytic triads from PpClpP1, PaClpP1, and PaClpP2, with catalytic residues (Ser-His-Asp/Pro) represented as stick models. (TIF) [file ppat.1013909.s004.tif]

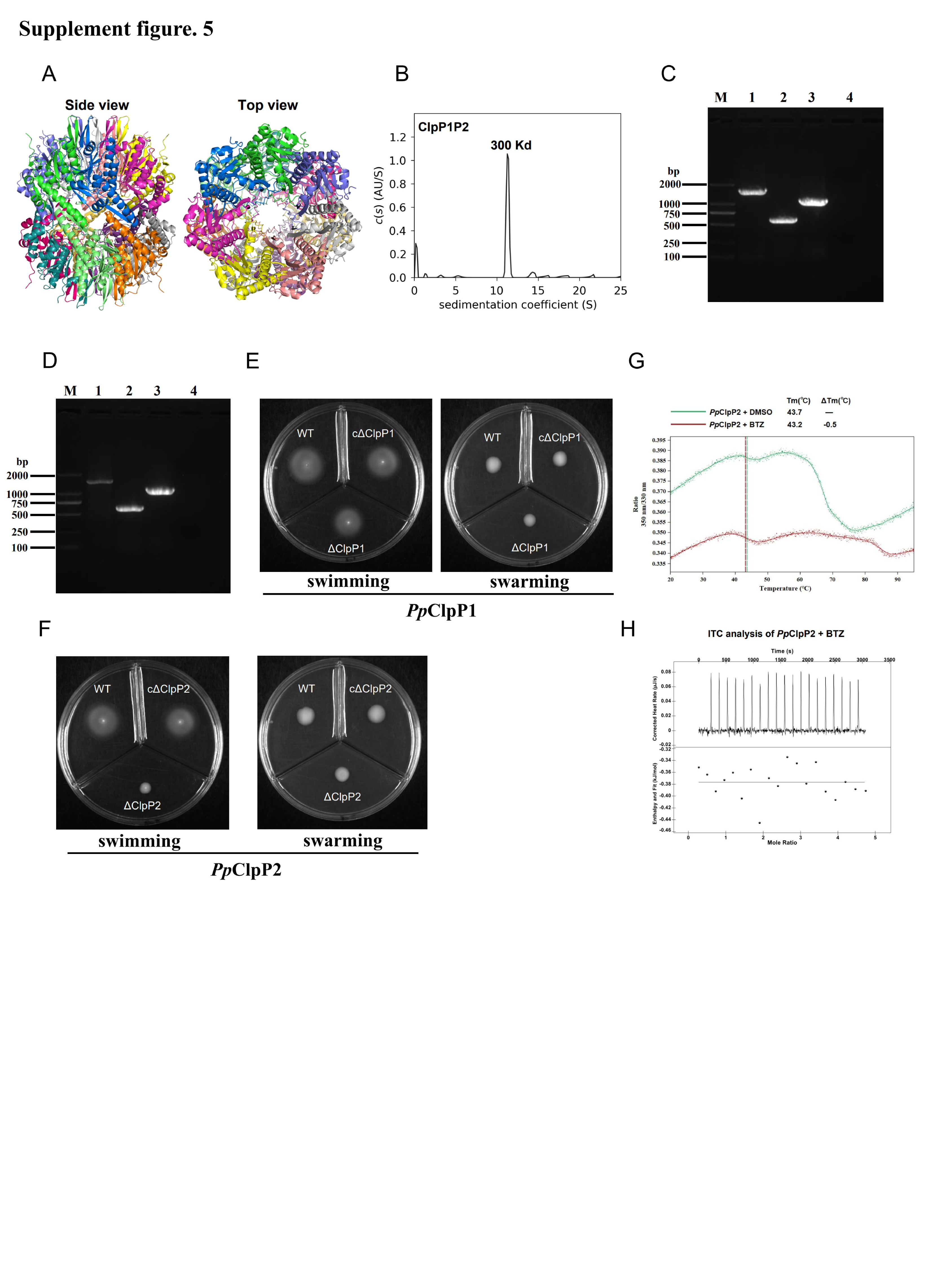

Supplement: S5 Fig — (A) AlphaFold-predicted tetradecameric structure of PpClpP2 shown as ribbon diagram, demonstrating conserved oligomeric architecture. The modeled structures were supported by high prediction confidence scores, with an ipTM of 0.87, a pTM of 0.88, and a plDDT value exceeding 90. (B) AUC analysis revealed that the molecular weight of the PpClpP1P2 complex is approximately 300 kDa, which is consistent with the theoretical molecular weight of a tetradecameric complex (20 × 7 + 23 × 7 = 301 kDa). (C) Genotype confirmation of the knockout mutant strain. Confirmation of gene knockout by PCR with pairs of primers designed to target outside of the deletion domain. Lane M: DNA marker (DL2000); Lane 1: The 1552 bp fragment amplified from genomic DNA of wild-type P. plecoglossicida with primer set 18TcΔPpClpP1-U F/R. Lane 2: The 552 bp fragment amplified from P. plecoglossicida strain with primer PpClpP1-F/R. Lane 3: The 1000 bp fragment amplified from ΔPpClpP1 strain with primer set ΔPpClpP1-U F/R. Lane 4: The 0 bp fragment amplified from ΔPpClpP1 strain with primer set PpClpP1-F/R. (D) Lane 1: The 1642 bp fragment amplified from genomic DNA of wild-type P. plecoglossicida with primer set 18TcΔPpClpP2-U F/R. Lane 2: The 642 bp fragment amplified from P. plecoglossicida strain with primer PpClpP2-F/R. Lane 3: The 1000 bp fragment amplified from ΔPpClpP2 strain with primer set ΔPpClpP2-U F/R. Lane 4: The 0 bp fragment amplified from ΔPpClpP2 strain with primer set PpClpP2-F/R. (E-F) Swimming and swarming motility of Pseudomonas plecoglossicida wild-type and mutant strains and complemented strains. (G) DSF analysis revealed no significant thermal shift in PpClpP2 upon the addition of BTZ, suggesting a lack of direct interaction between them. (H) ITC binding isotherm for BTZ (300 μM) titrated into PpClpP2 (30 μM), the resulting binding isotherm indicated no observable interaction between the two molecules. (TIF) [file ppat.1013909.s005.tif]

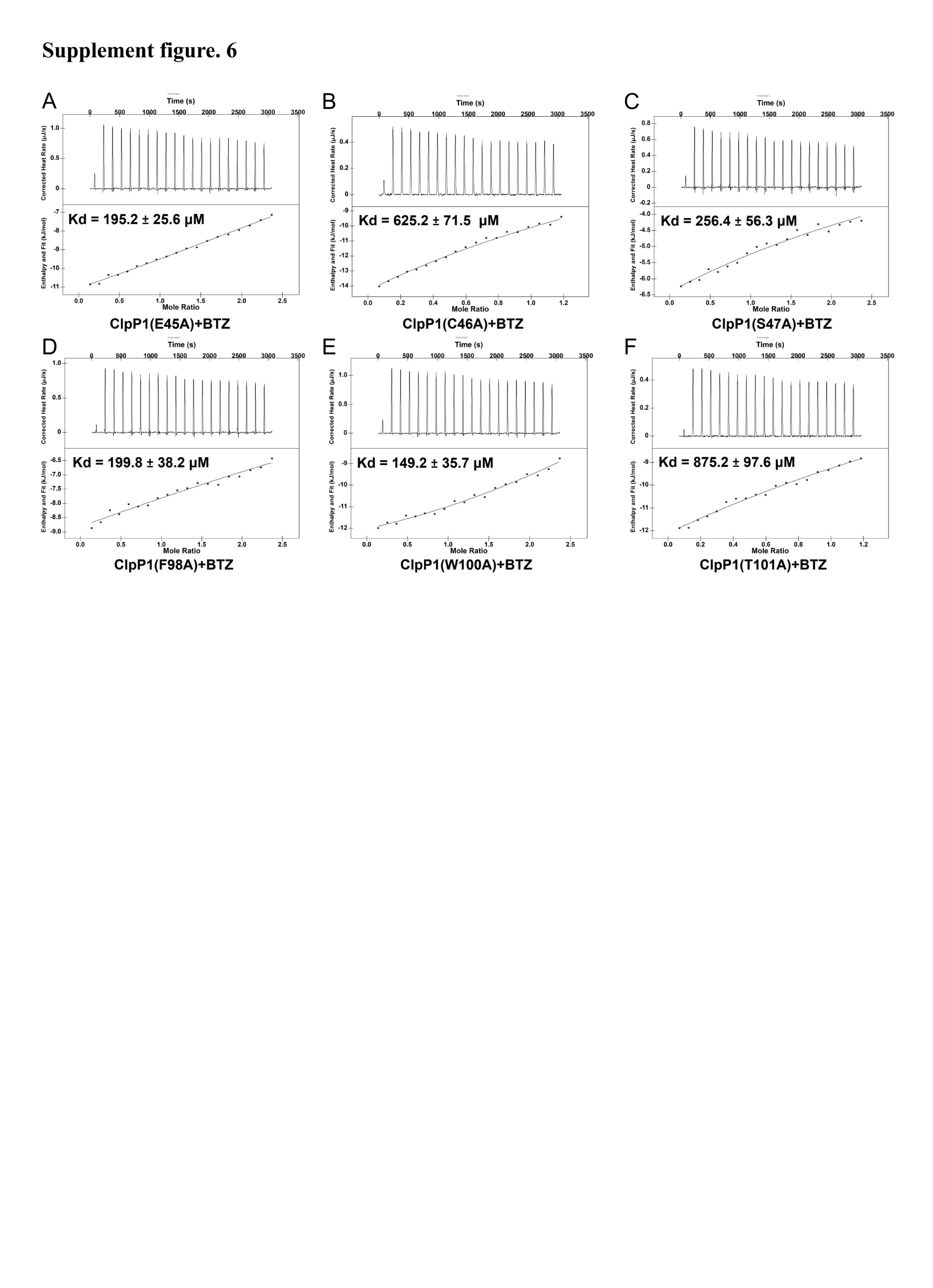

Supplement: S6 Fig — Isothermal titration calorimetry (ITC) binding isotherm for BTZ (300 μM) titrated into PpClpP1 mutants (30 μM), with derived binding parameters: (A) ClpP1 (E45A) + BTZ, Kd = 195.2 ± 25.6 μM; (B) ClpP1 (C46A) + BTZ, Kd = 625.2 ± 71.5; (C) ClpP1 (S47A) + BTZ, Kd = 256.4 ± 56.3; (D) ClpP1 (F98A) + BTZ, Kd = 199.8 ± 38.2; (E) ClpP1 (W100A) + BTZ, Kd = 149.2 ± 35.7; (F) ClpP1 (T101A) + BTZ, Kd = 875.2 ± 97.6. These were the original data for results summarized in Fig 7F. The binding affinity are also shown within. Data represent mean values ± s.d. (TIF) [file ppat.1013909.s006.tif]

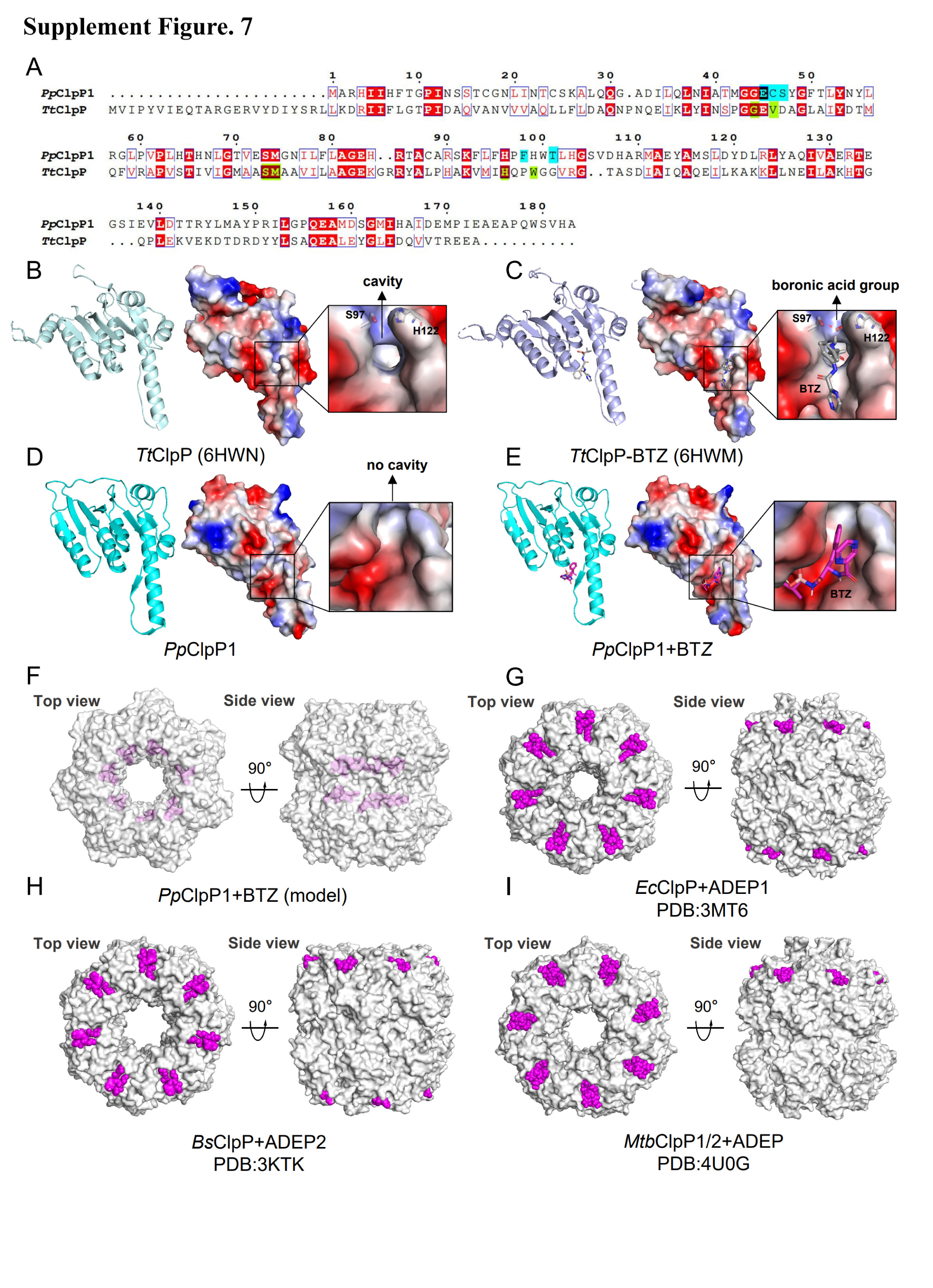

Supplement: S7 Fig — (A) Multiple sequence alignment of PpClpP1 and TtClpP, the alignment was created using the ESPript 3.0 server Aligned Sequences tool. Amino acids have been colored with similarity coloring scheme % MultAlin, Global score 0.7. Residues for the binding of PpClpP1 and TtClpP to bortezomib are highlighted in blue and green, respectively. (B) Structure of the TtClpP (PDB: 6HWN) monomer in the apo state. Left, cartoon diagram. Right, surface diagram, the region where BTZ binds forms a deep cavity, with residues S97 and H122 located near its base. (C) Structure of the TtClpP (PDB: 6HWM) monomer in complex with BTZ. Left, cartoon diagram. Right, surface diagram, the boronic acid group of BTZ projects into the binding cavity, forming interactions with S97 and H122. (D) Structure of the PpClpP1 monomer. Left, cartoon diagram. Right, surface diagram, the BTZ-binding site in PpClpP1 forms a distinct groove. (E) Predicted structural model of the PpClpP1 monomer in complex with BTZ. Left, cartoon diagram. Right, surface diagram. BTZ is predicted to bind within this groove. (F-I) Comparative structural analysis reveals distinct binding modes of small-molecule modulators across bacterial species: (F) PpClpP1-BTZ complex showing intra-subunit binding; (G) E. coli ClpP (EcClpP) in complex with ADEP1; (H) B. subtilis ClpP (BsClpP) in complex with ADEP2; and (I) M. tuberculosis ClpP1/2 (MtbClpP1/2) in complex with ADEP, demonstrating conserved inter-subunit binding pockets for acyldepsipeptides (ADEPs) that contrast with BTZ’s unique binding topology in PpClpP1. (TIF) [file ppat.1013909.s007.tif]

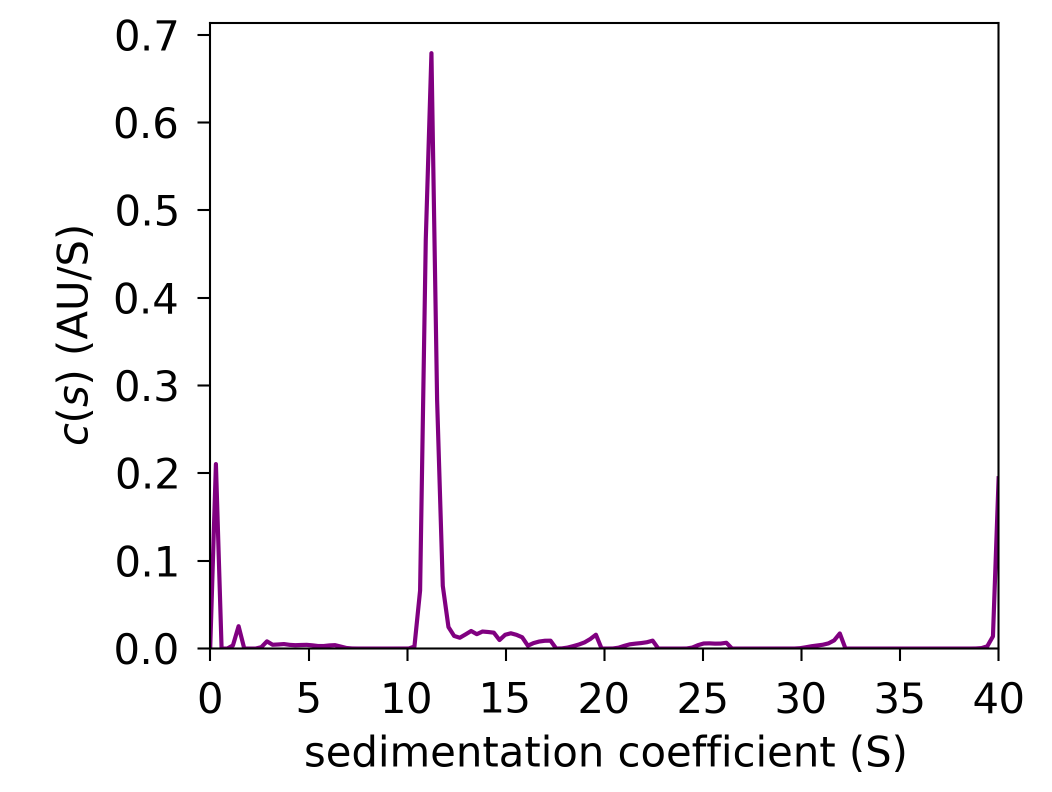

Supplement: S1 File — (ZIP) [file ppat.1013909.s010.zip › Fig 5/Fig 5A-AUC.tif]

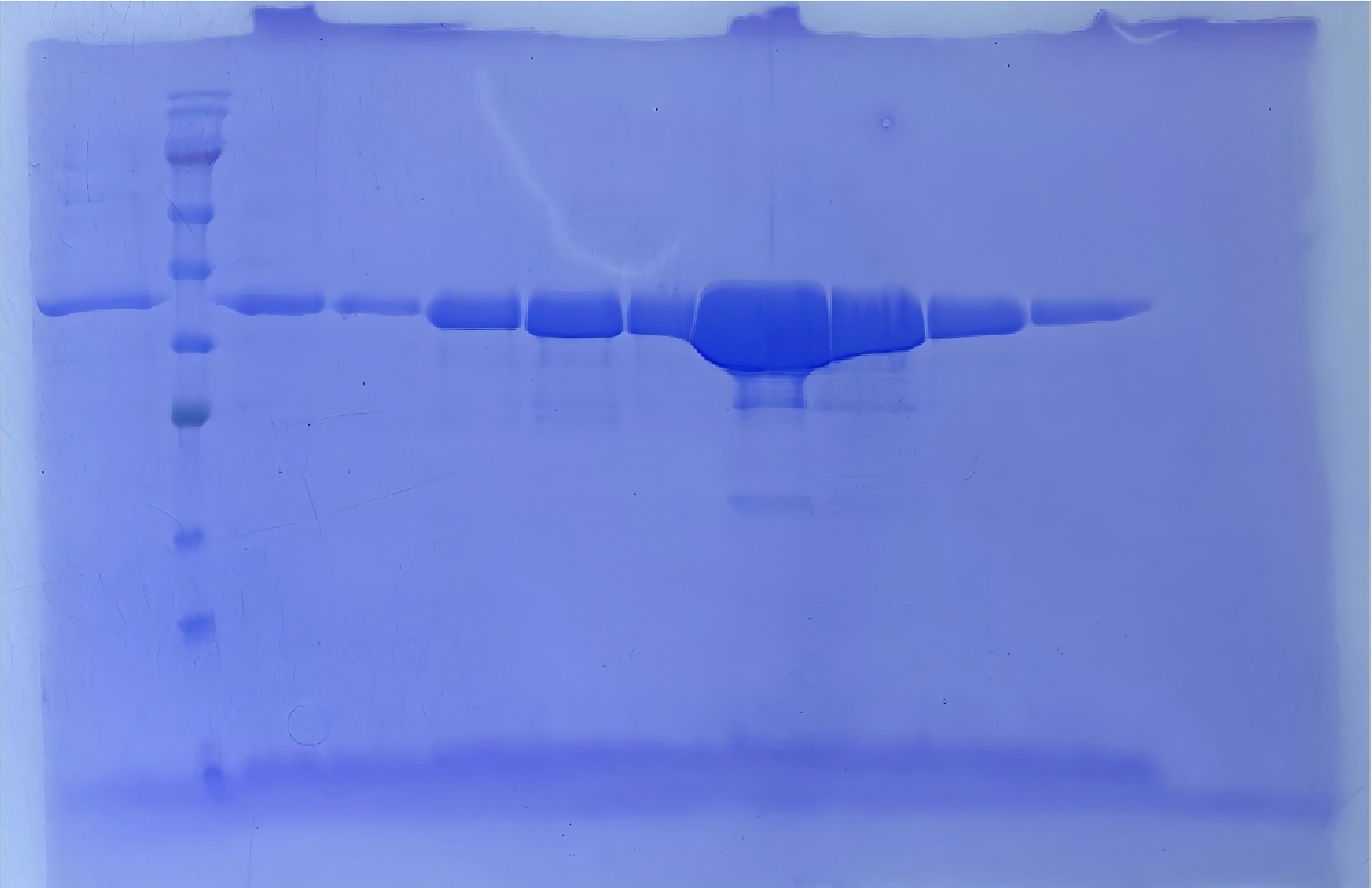

Supplement: S1 File — (ZIP) [file ppat.1013909.s010.zip › Fig 5/Fig 5A-gel.jpg]

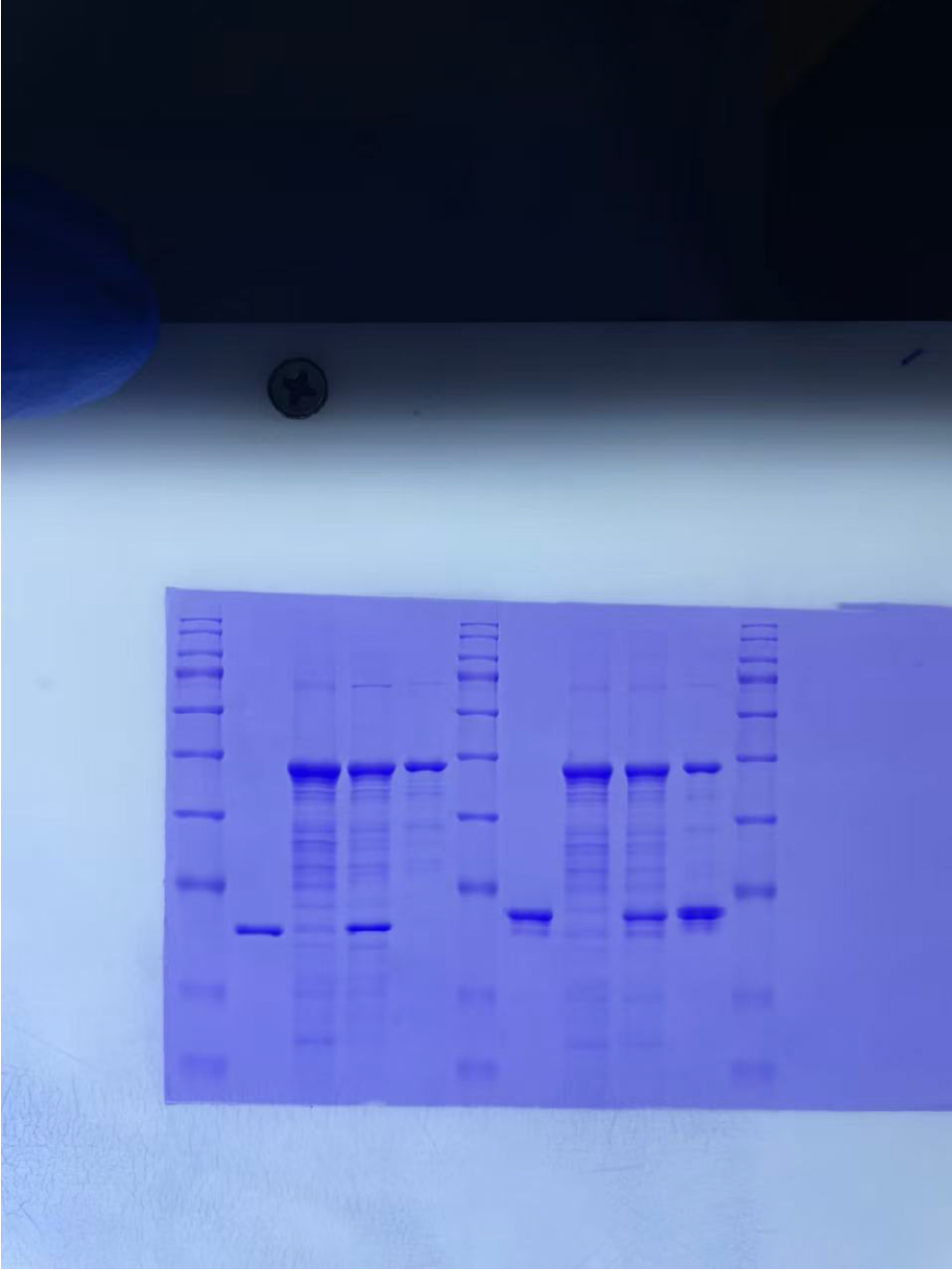

Supplement: S1 File — (ZIP) [file ppat.1013909.s010.zip › Fig 5/Fig 5B-gel.jpg]

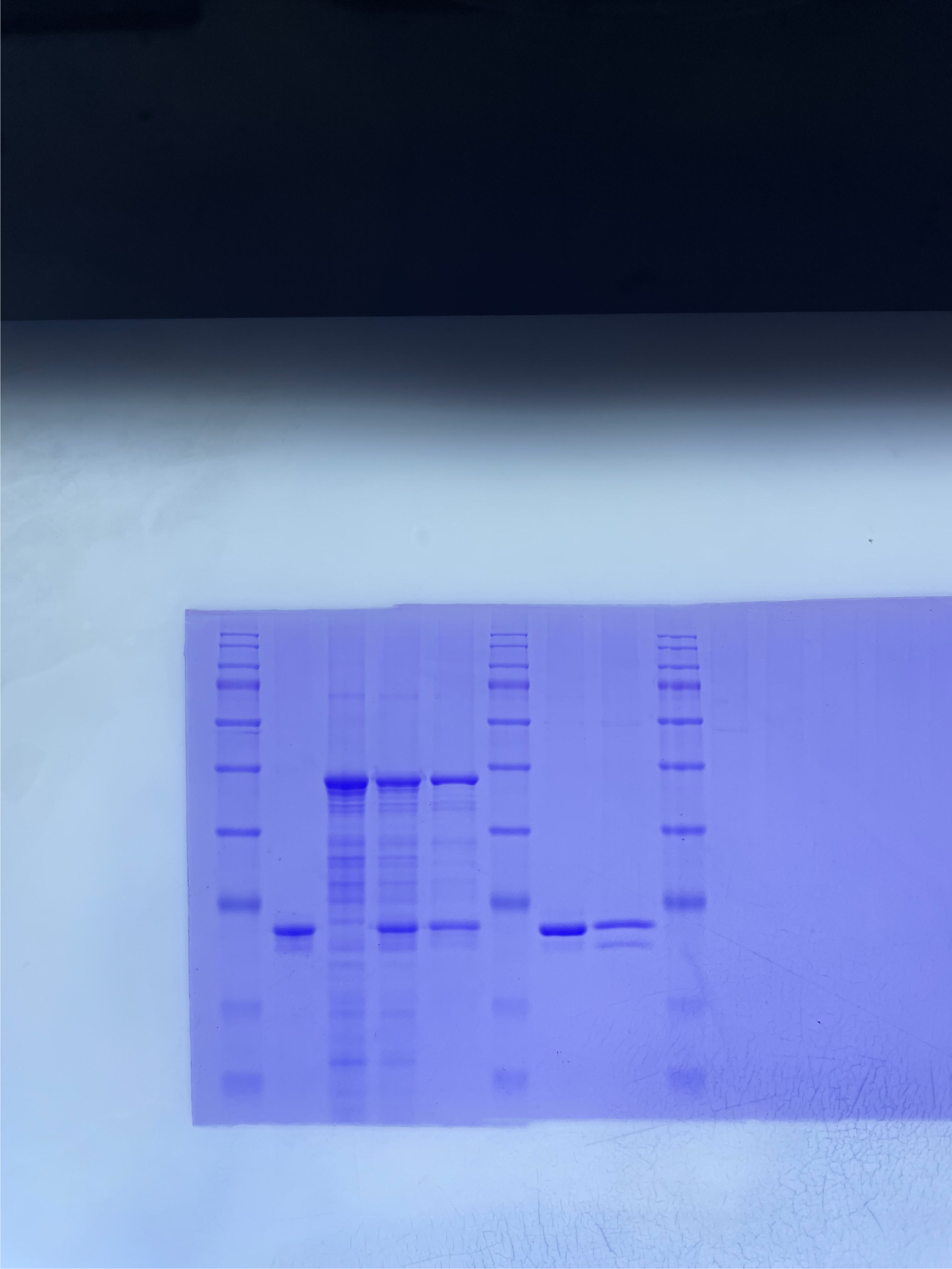

Supplement: S1 File — (ZIP) [file ppat.1013909.s010.zip › Fig 5/Fig 5C-gel.jpg]

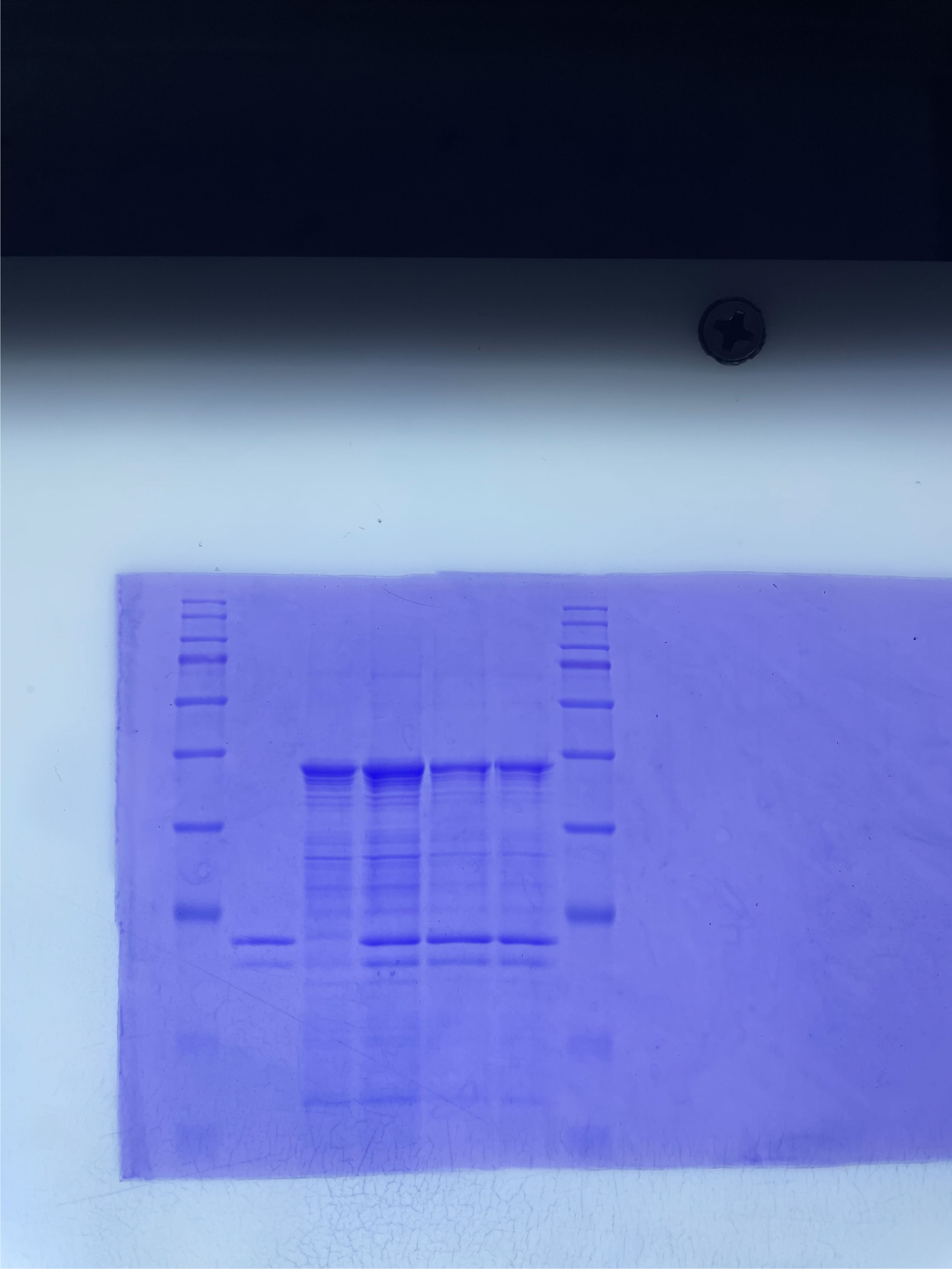

Supplement: S1 File — (ZIP) [file ppat.1013909.s010.zip › Fig 5/Fig 5D-gel.jpg]

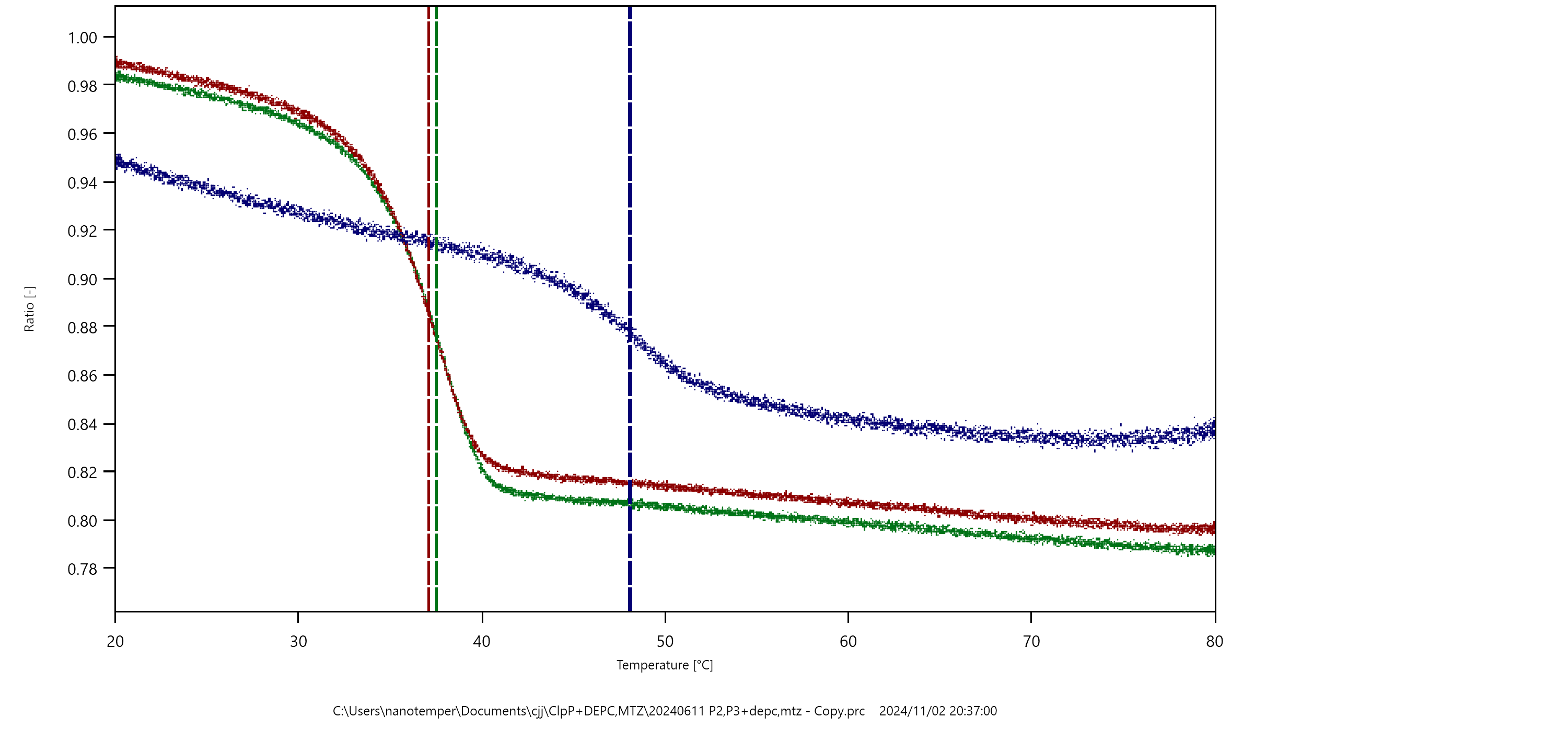

Supplement: S1 File — (ZIP) [file ppat.1013909.s010.zip › Fig 6/Fig 6B.tif]

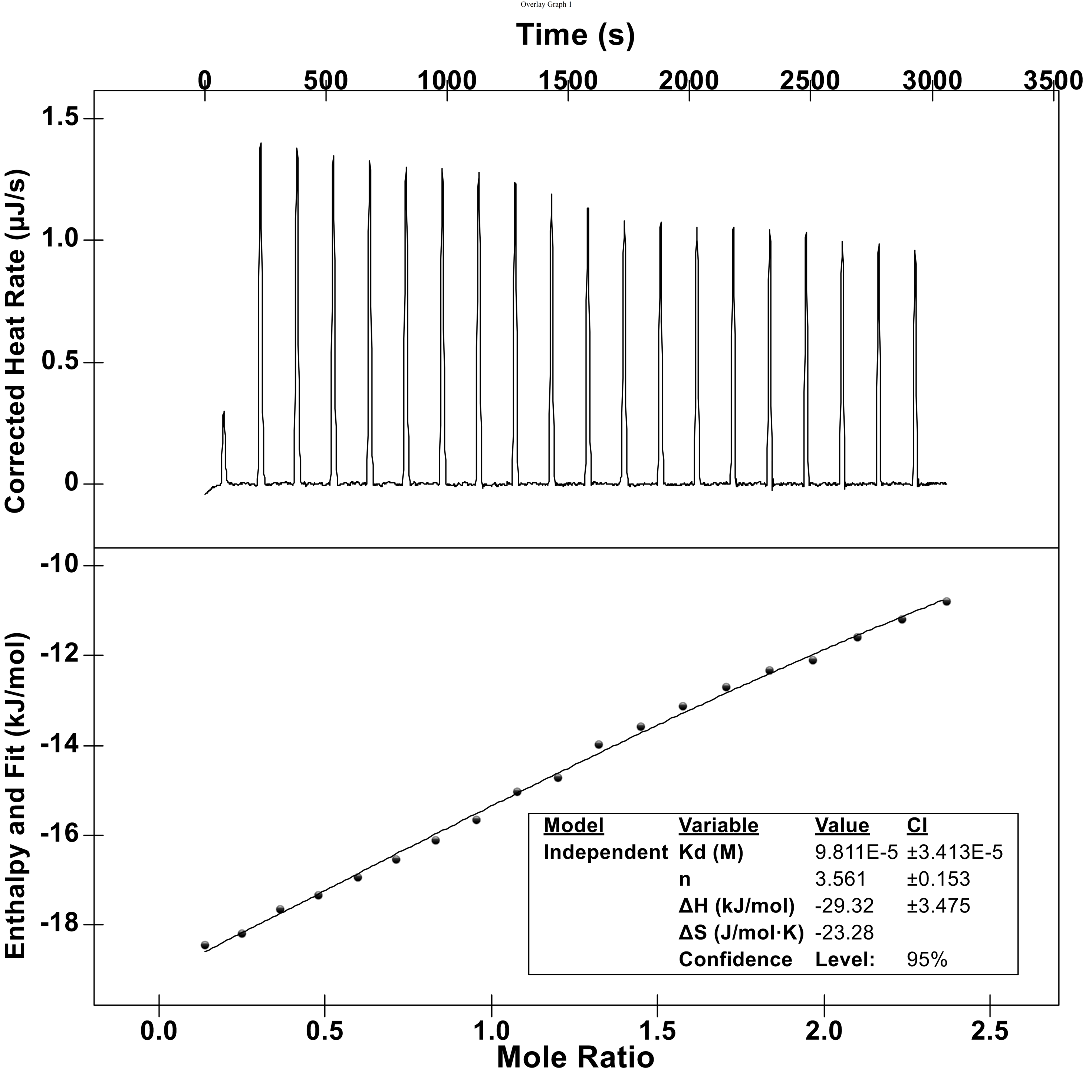

Supplement: S1 File — (ZIP) [file ppat.1013909.s010.zip › Fig 6/Fig 6C.tif]

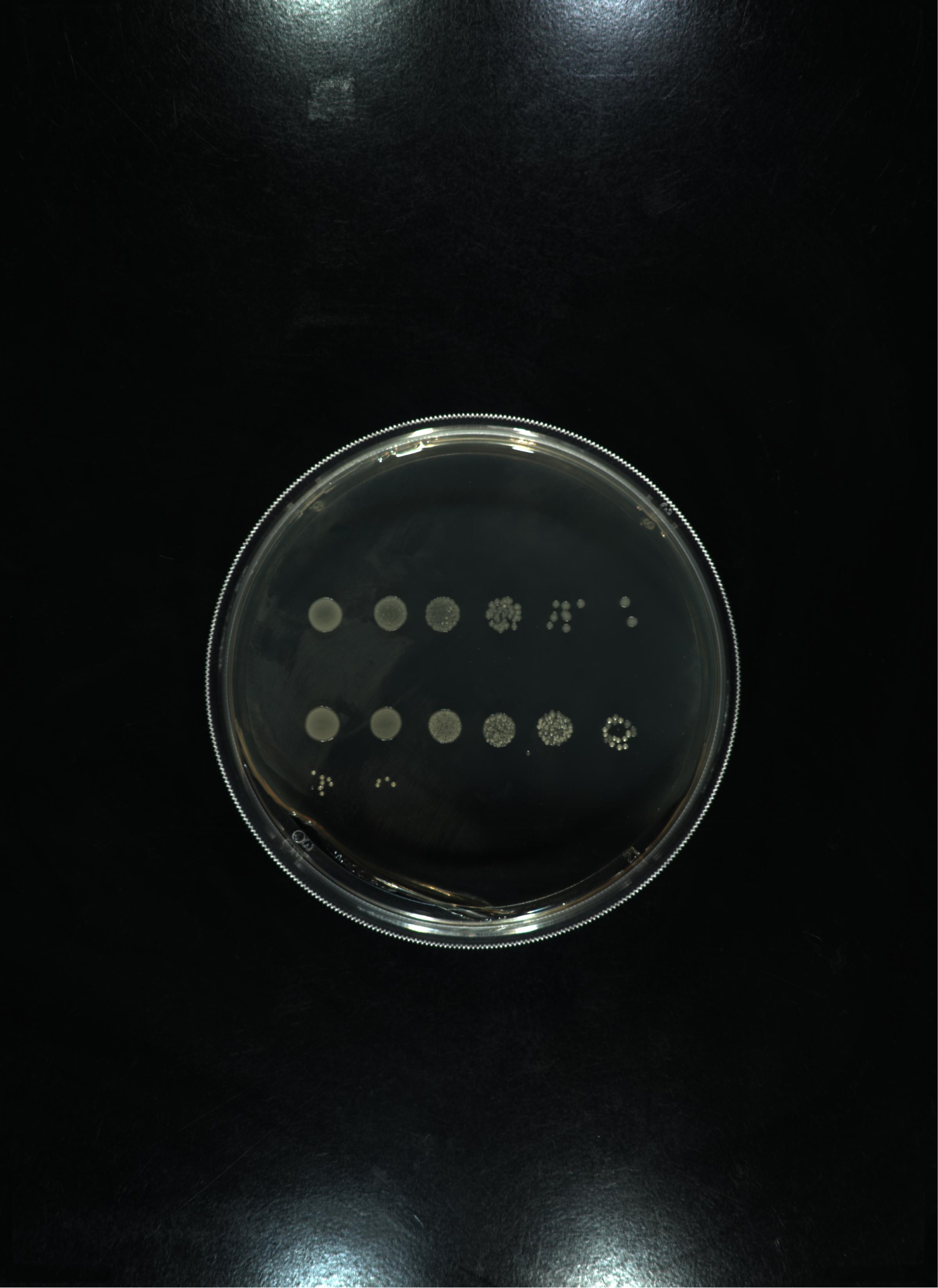

Supplement: S1 File — (ZIP) [file ppat.1013909.s010.zip › Fig 6/Fig 6F-Left.jpg]

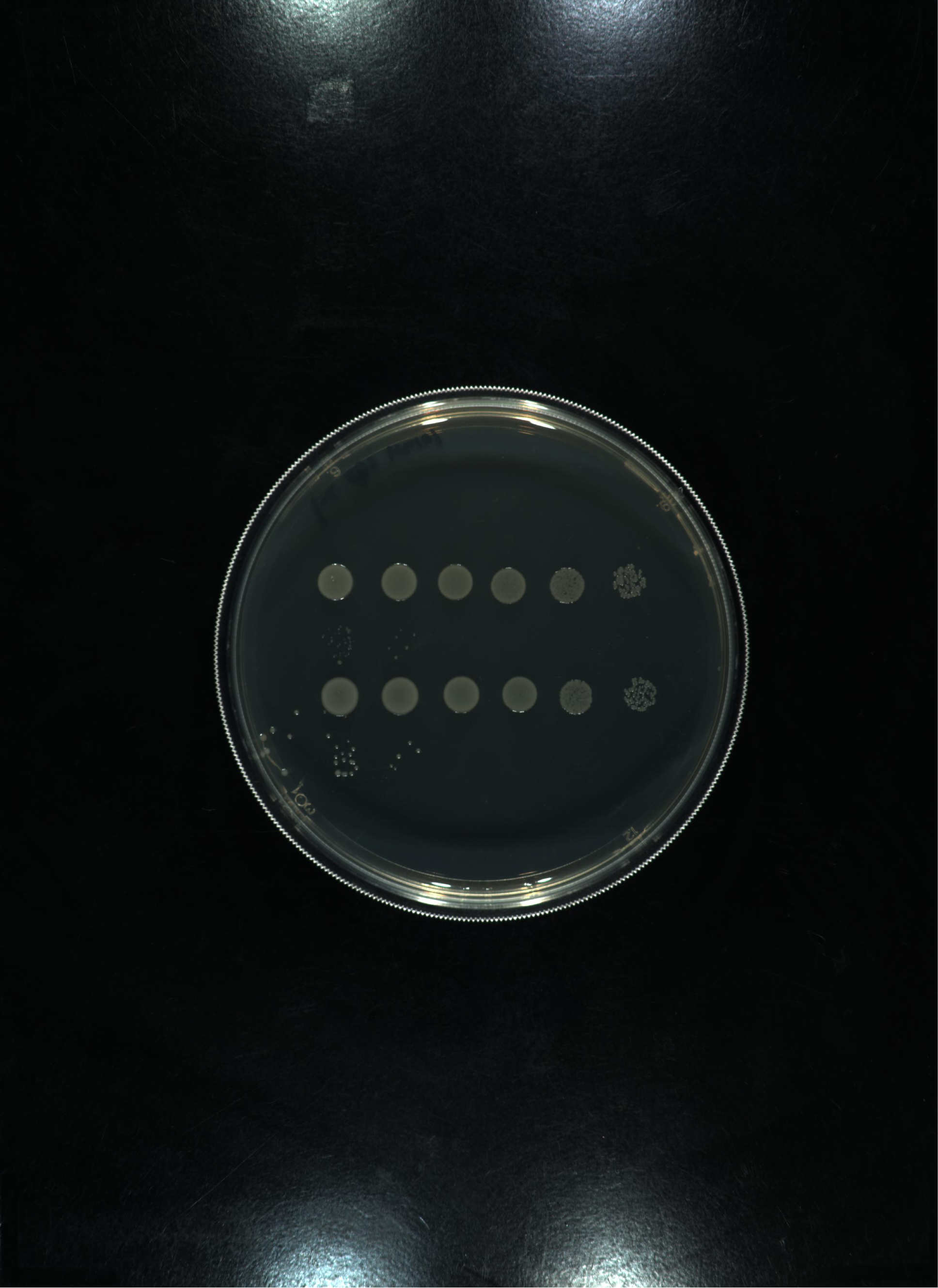

Supplement: S1 File — (ZIP) [file ppat.1013909.s010.zip › Fig 6/Fig 6G-Left.jpg]

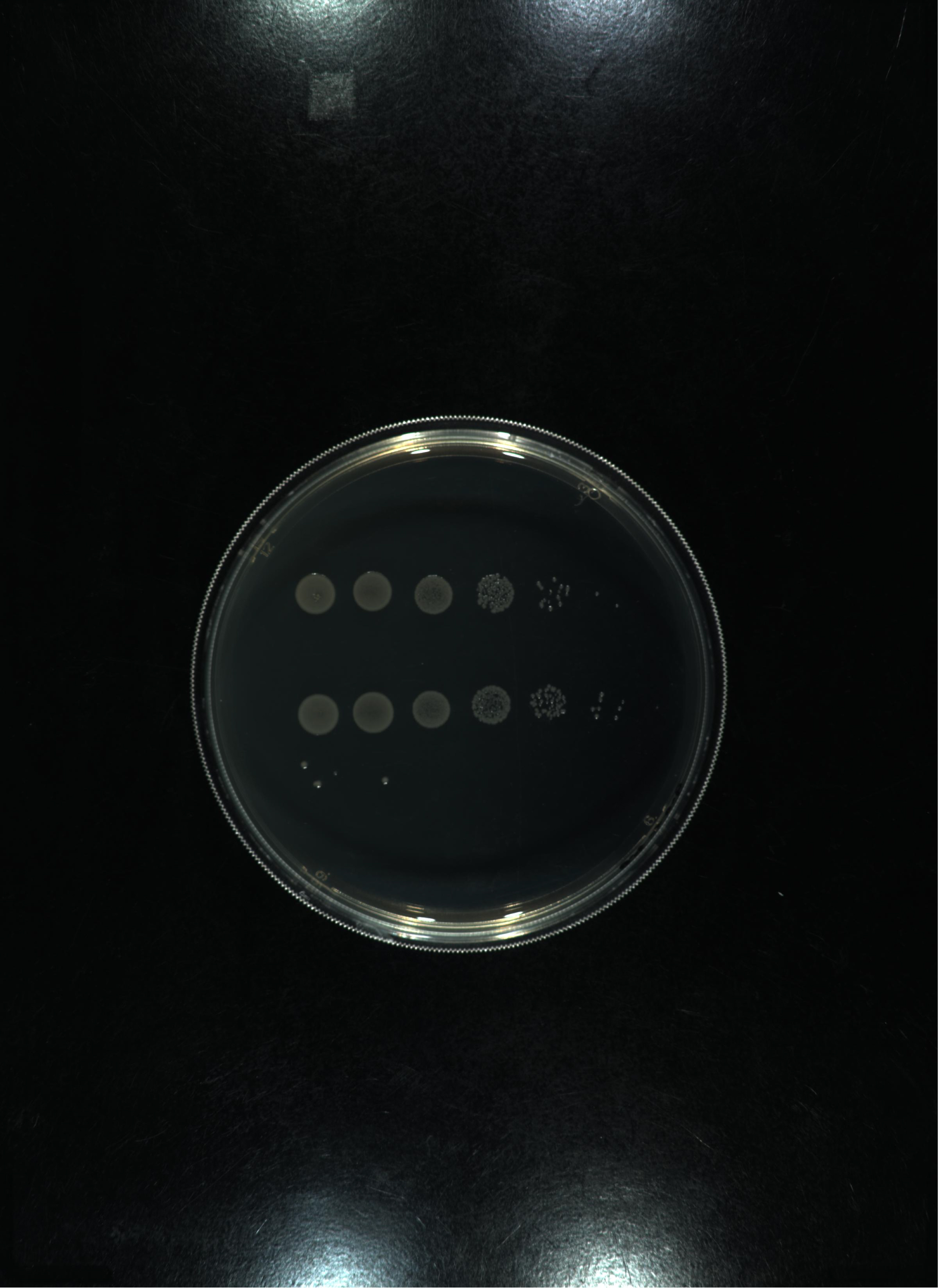

Supplement: S1 File — (ZIP) [file ppat.1013909.s010.zip › Fig 6/Fig 6H-Left.jpg]

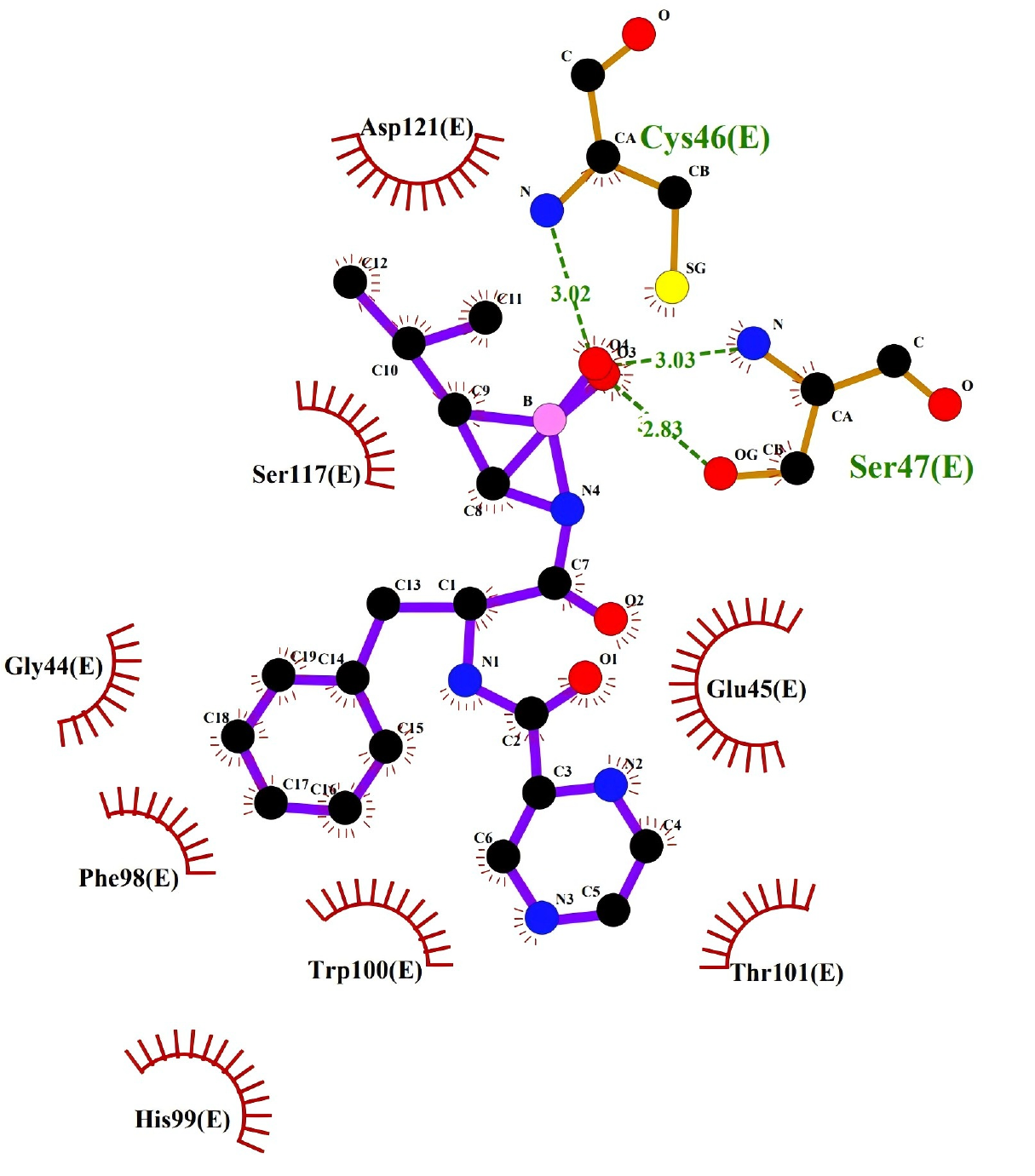

Supplement: S1 File — (ZIP) [file ppat.1013909.s010.zip › Fig 7/Fig 7A-Left.tif]

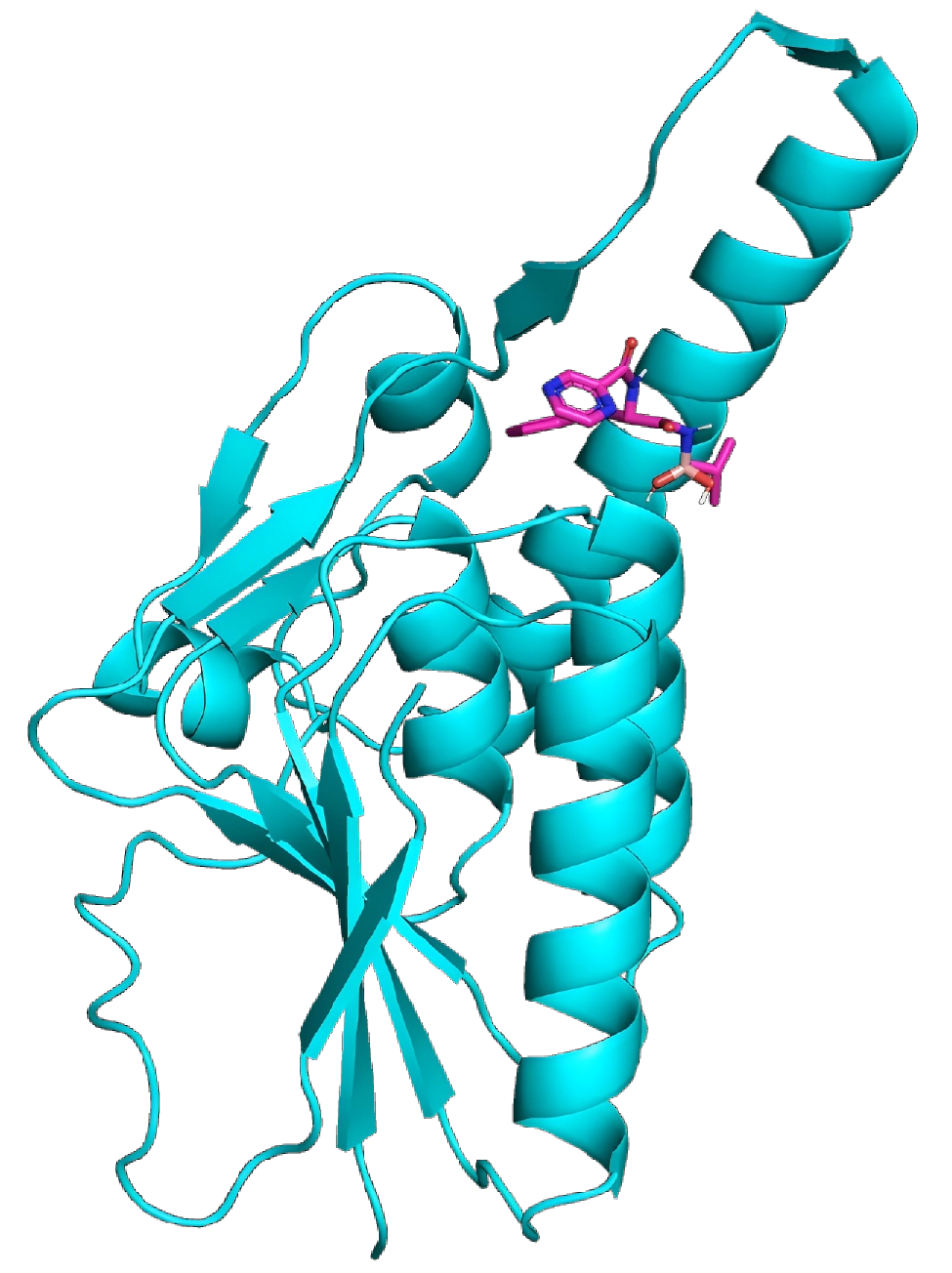

Supplement: S1 File — (ZIP) [file ppat.1013909.s010.zip › Fig 7/Fig 7A-Right.tif]

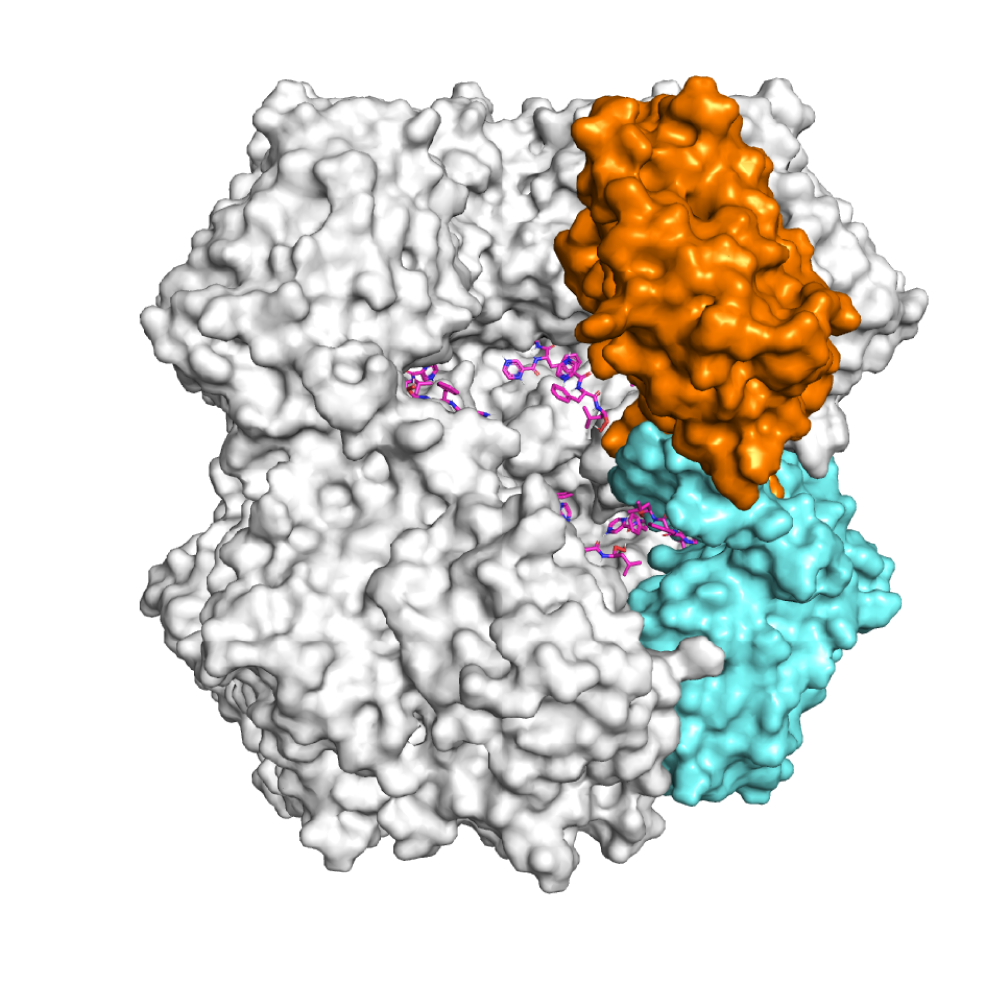

Supplement: S1 File — (ZIP) [file ppat.1013909.s010.zip › Fig 7/Fig 7B-1.tif]

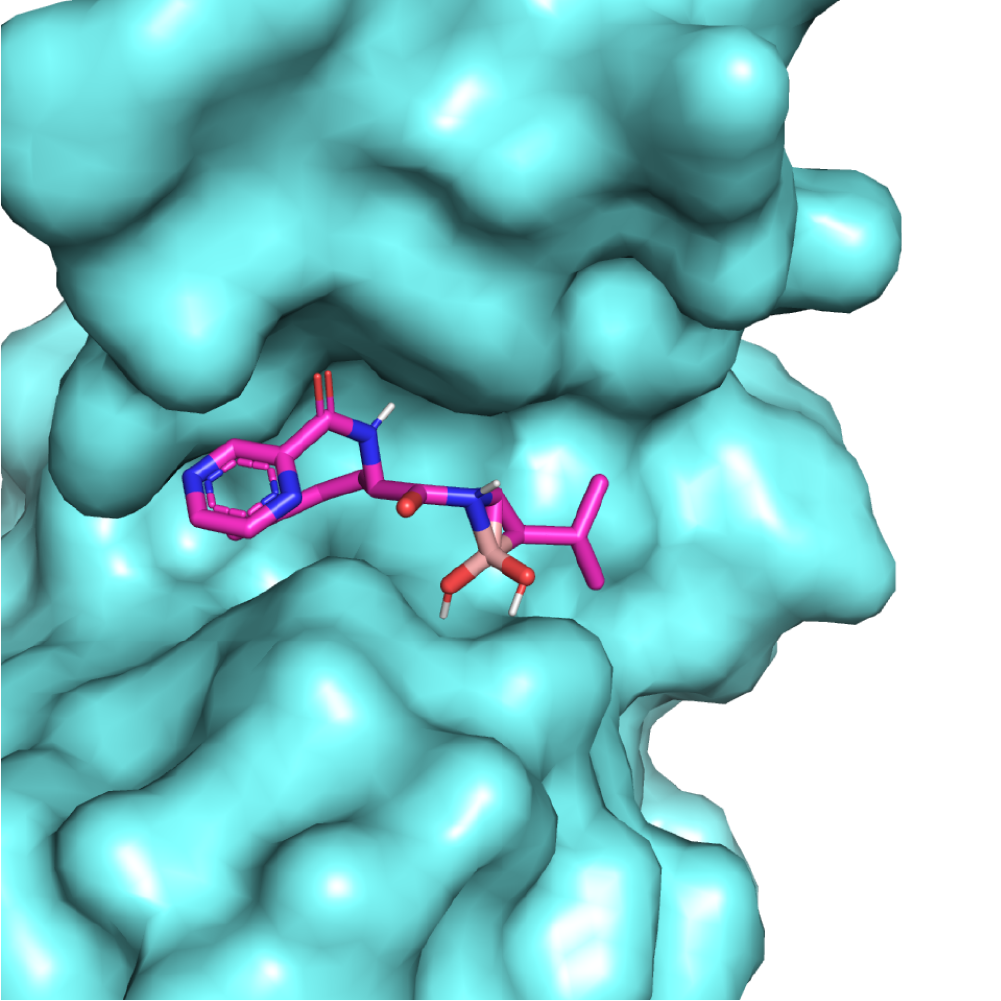

Supplement: S1 File — (ZIP) [file ppat.1013909.s010.zip › Fig 7/Fig 7B-2.tif]

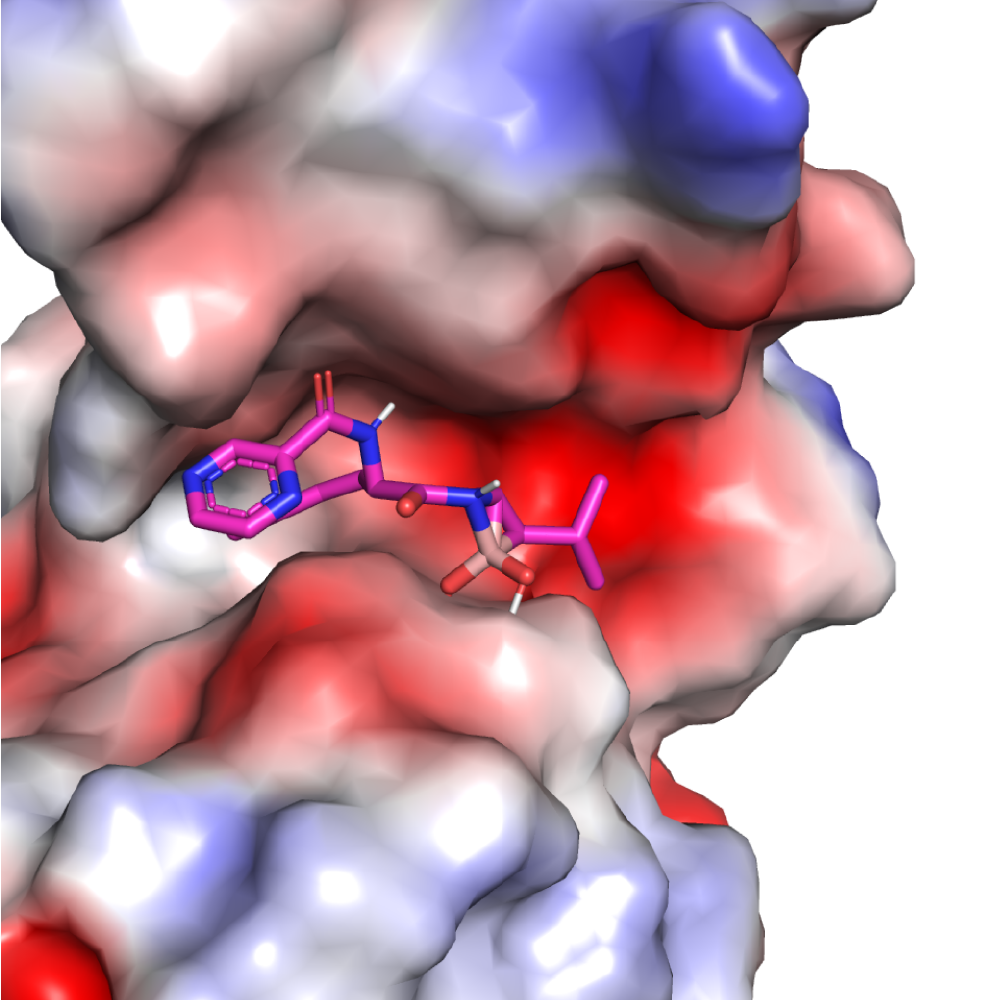

Supplement: S1 File — (ZIP) [file ppat.1013909.s010.zip › Fig 7/Fig 7B-3.tif]

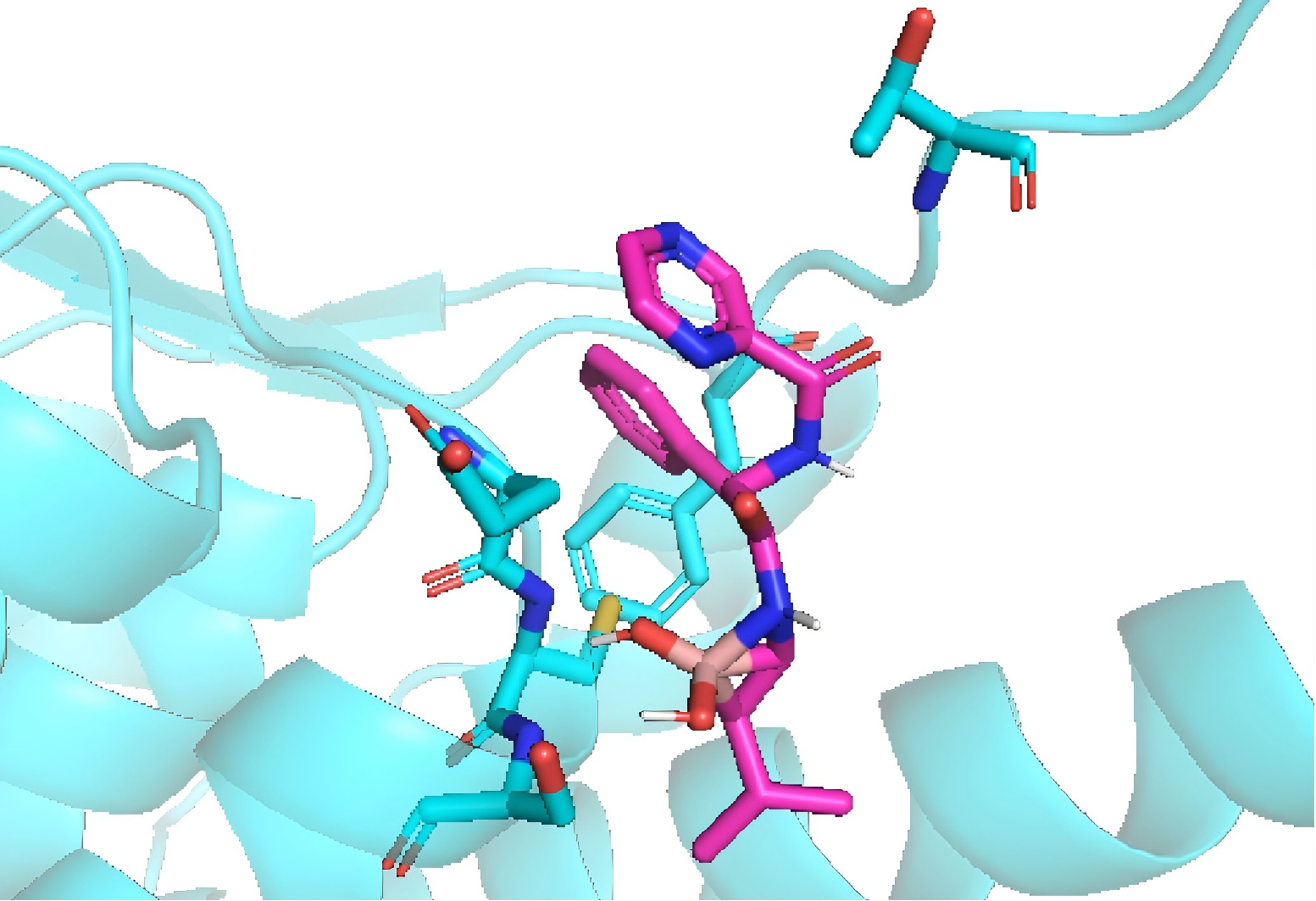

Supplement: S1 File — (ZIP) [file ppat.1013909.s010.zip › Fig 7/Fig 7C.tif]

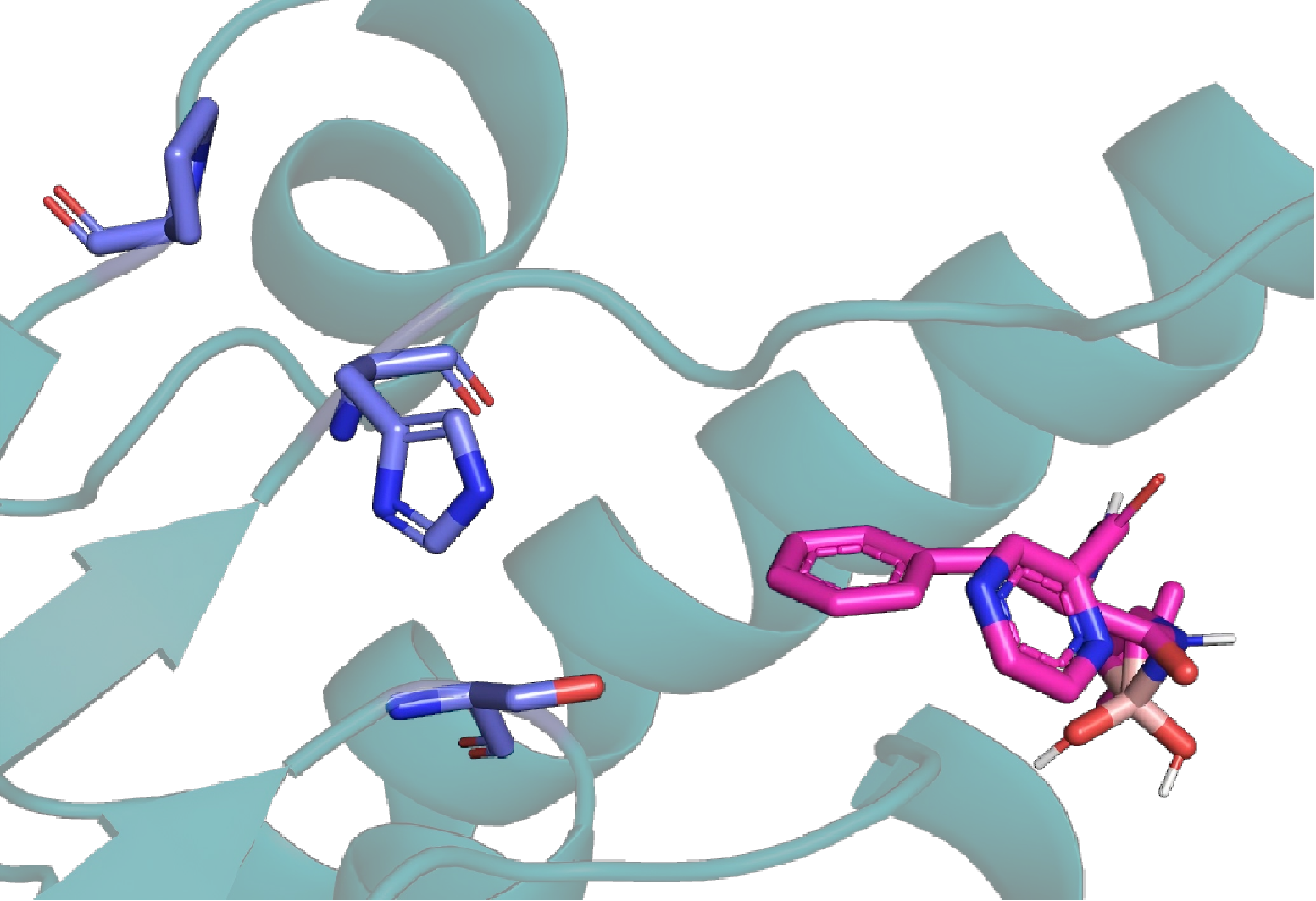

Supplement: S1 File — (ZIP) [file ppat.1013909.s010.zip › Fig 7/Fig 7D.tif]

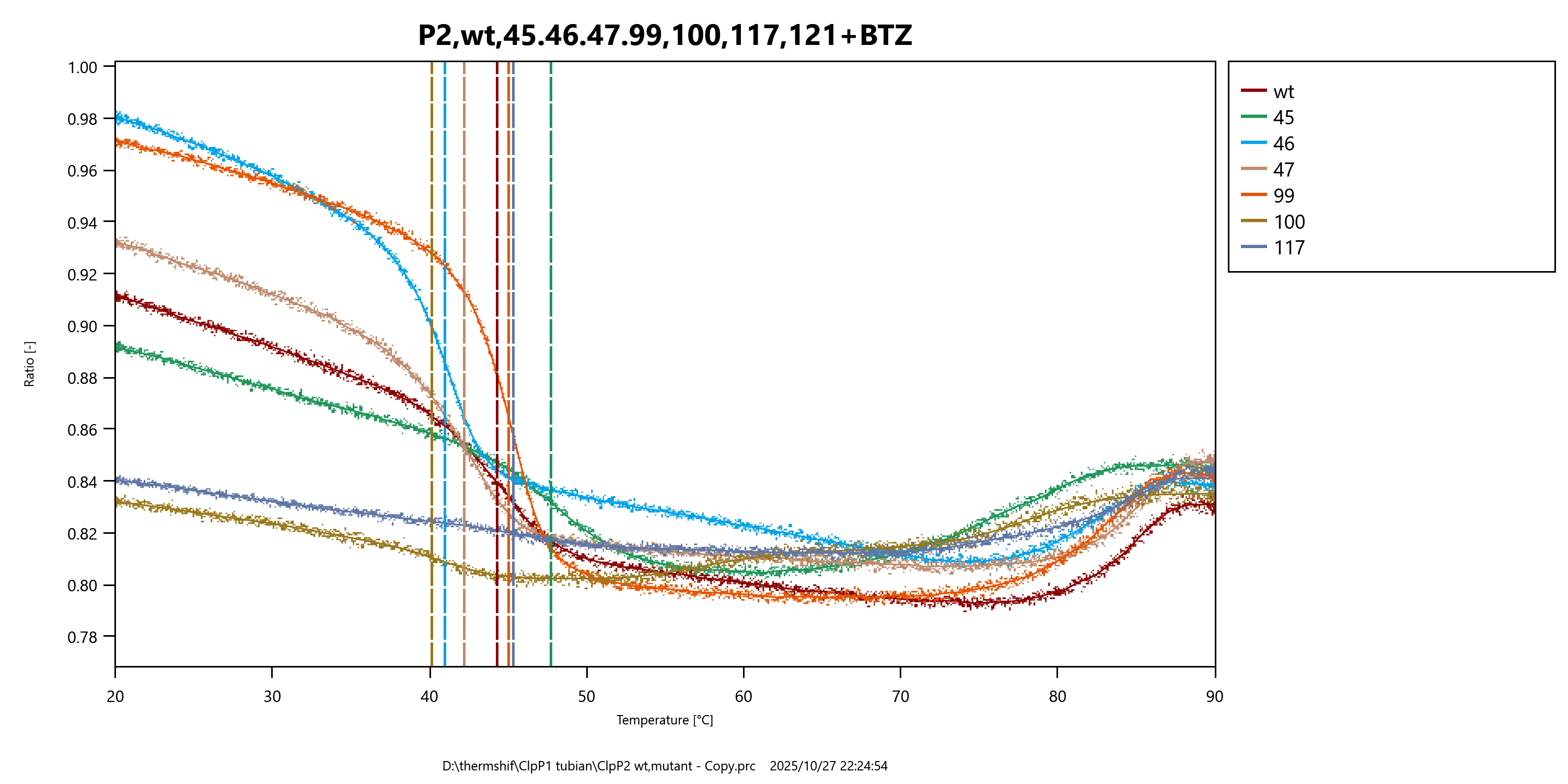

Supplement: S1 File — (ZIP) [file ppat.1013909.s010.zip › Fig 7/Fig 7E.tif]

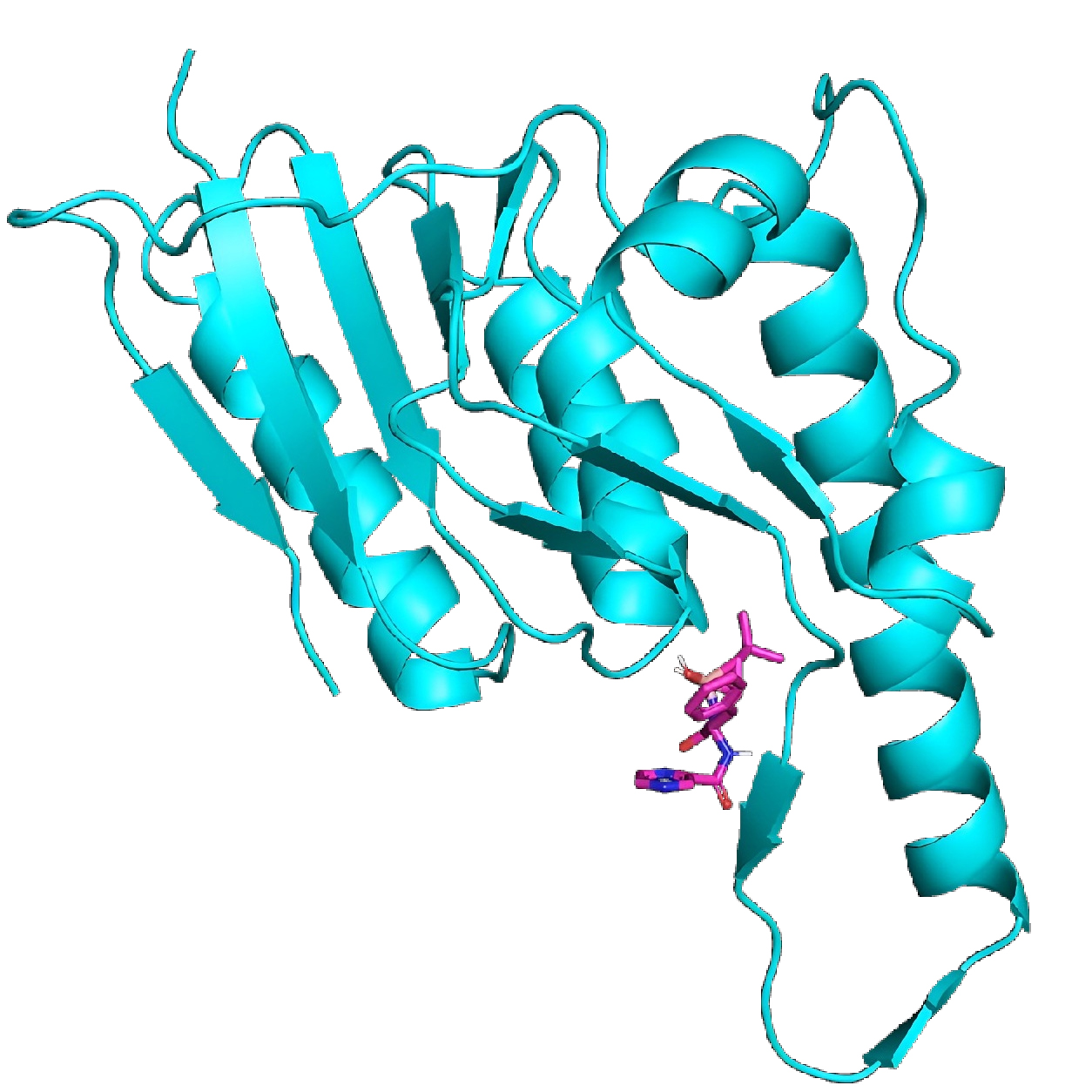

Supplement: S1 File — (ZIP) [file ppat.1013909.s010.zip › Fig 8/Fig 8A.jpg]

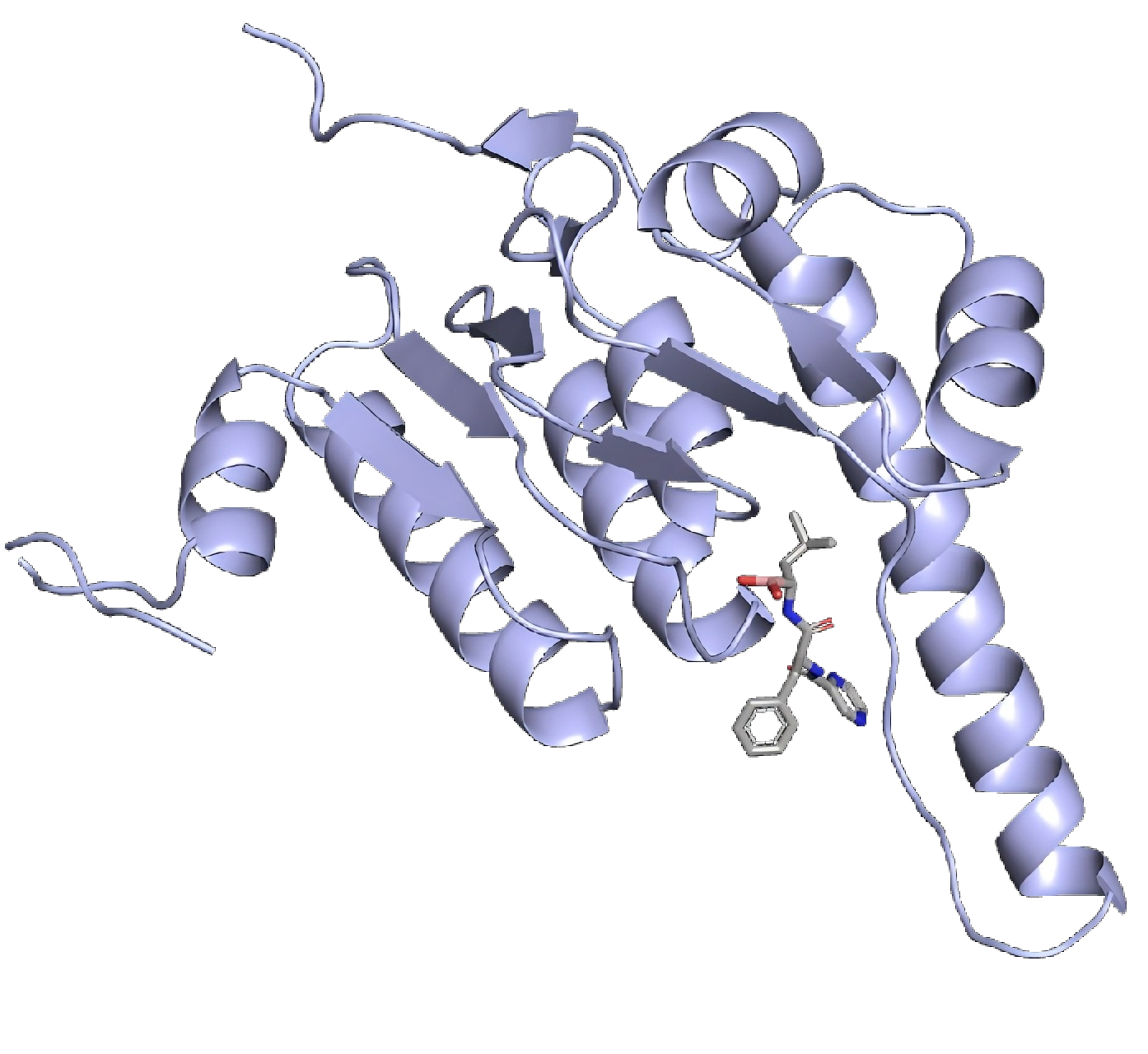

Supplement: S1 File — (ZIP) [file ppat.1013909.s010.zip › Fig 8/Fig 8B.jpg]

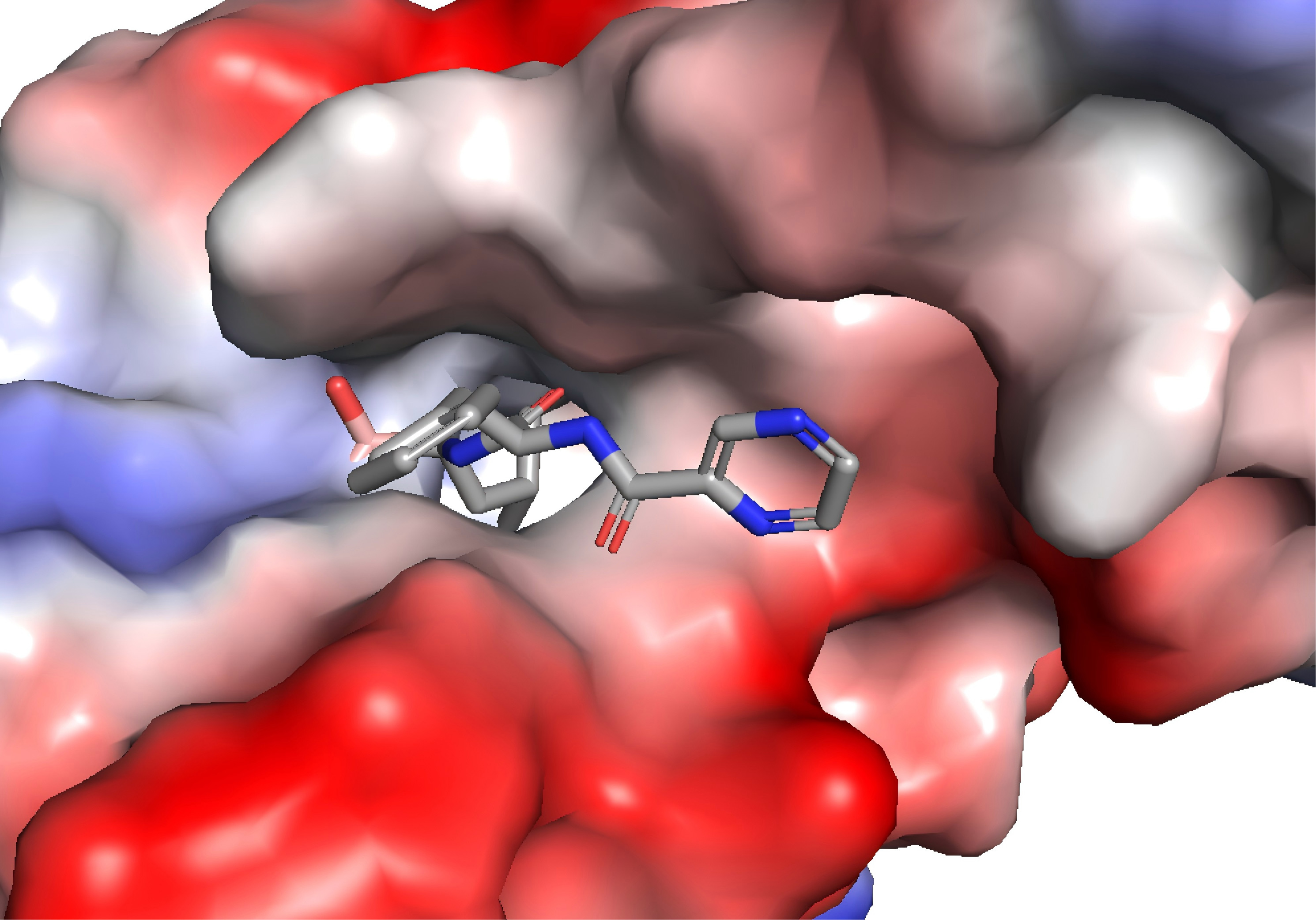

Supplement: S1 File — (ZIP) [file ppat.1013909.s010.zip › Fig 8/Fig 8C-right-1.jpg]

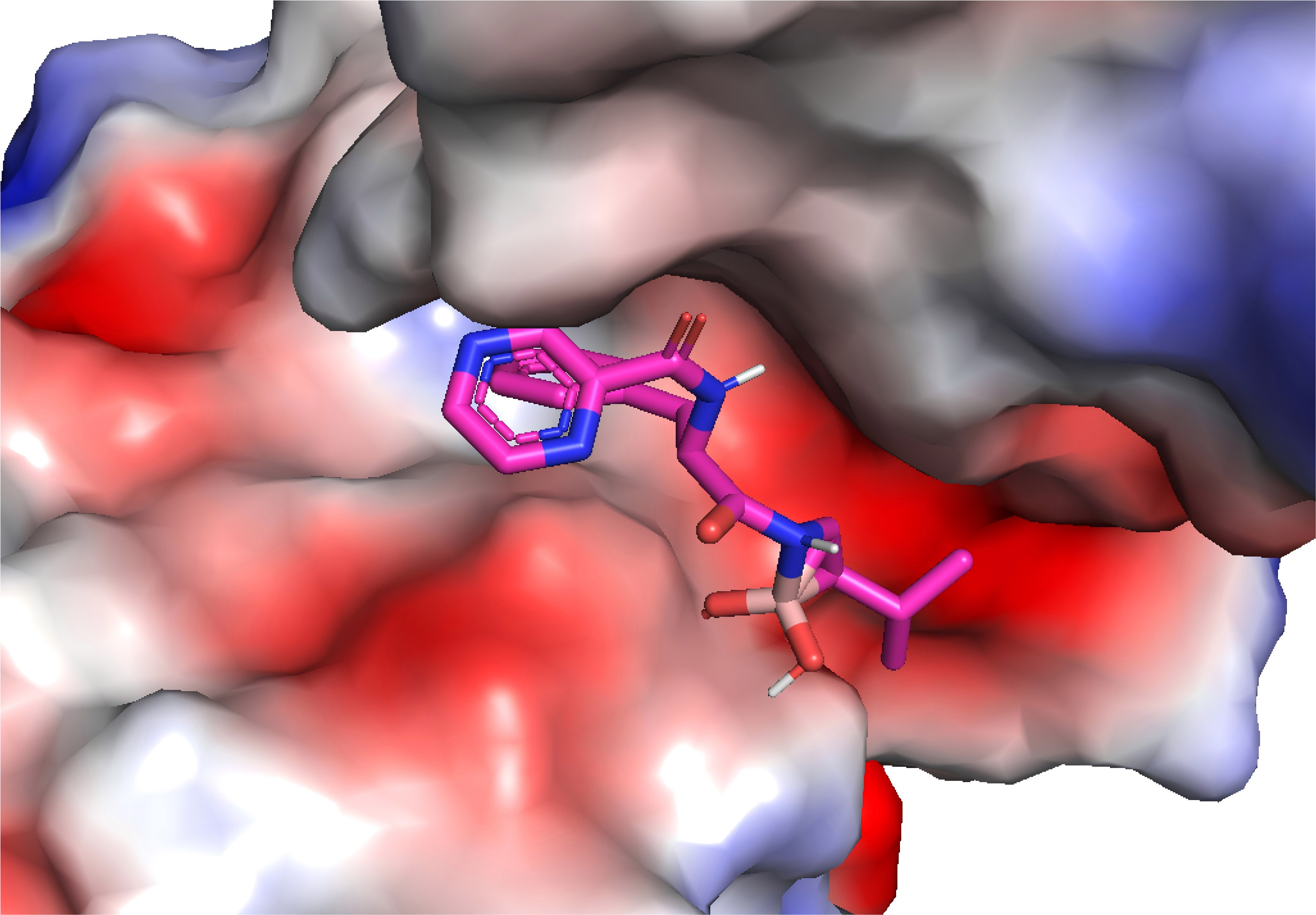

Supplement: S1 File — (ZIP) [file ppat.1013909.s010.zip › Fig 8/Fig 8C-right-2.jpg]

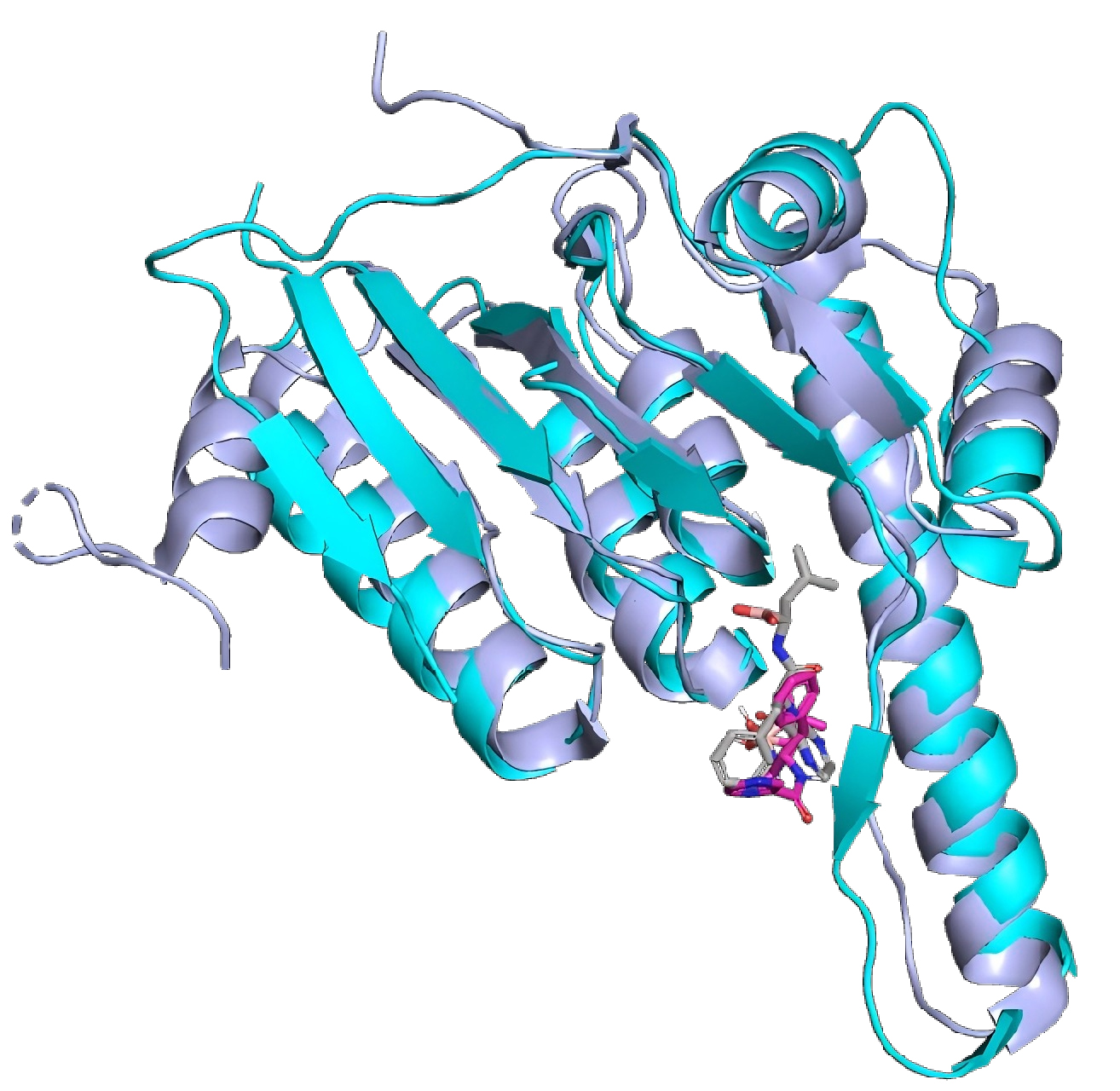

Supplement: S1 File — (ZIP) [file ppat.1013909.s010.zip › Fig 8/Fig 8C.jpg]

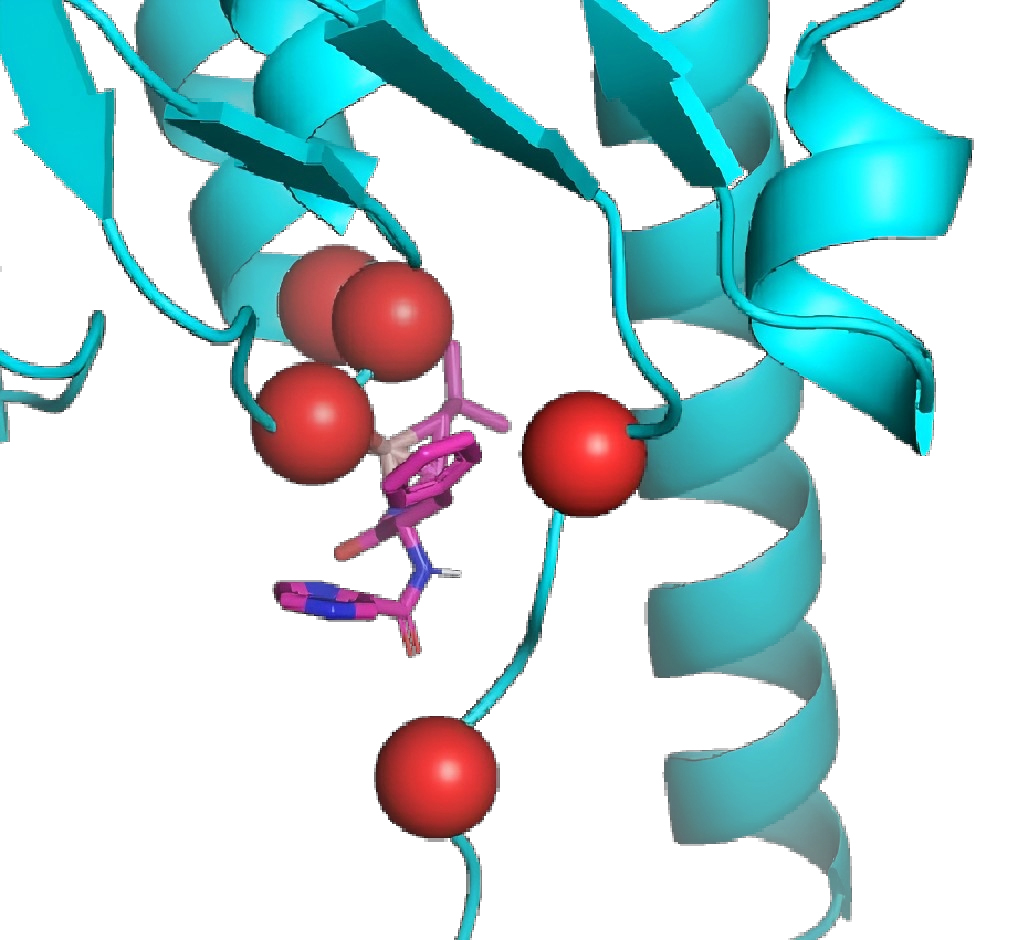

Supplement: S1 File — (ZIP) [file ppat.1013909.s010.zip › Fig 8/Fig 8D.jpg]

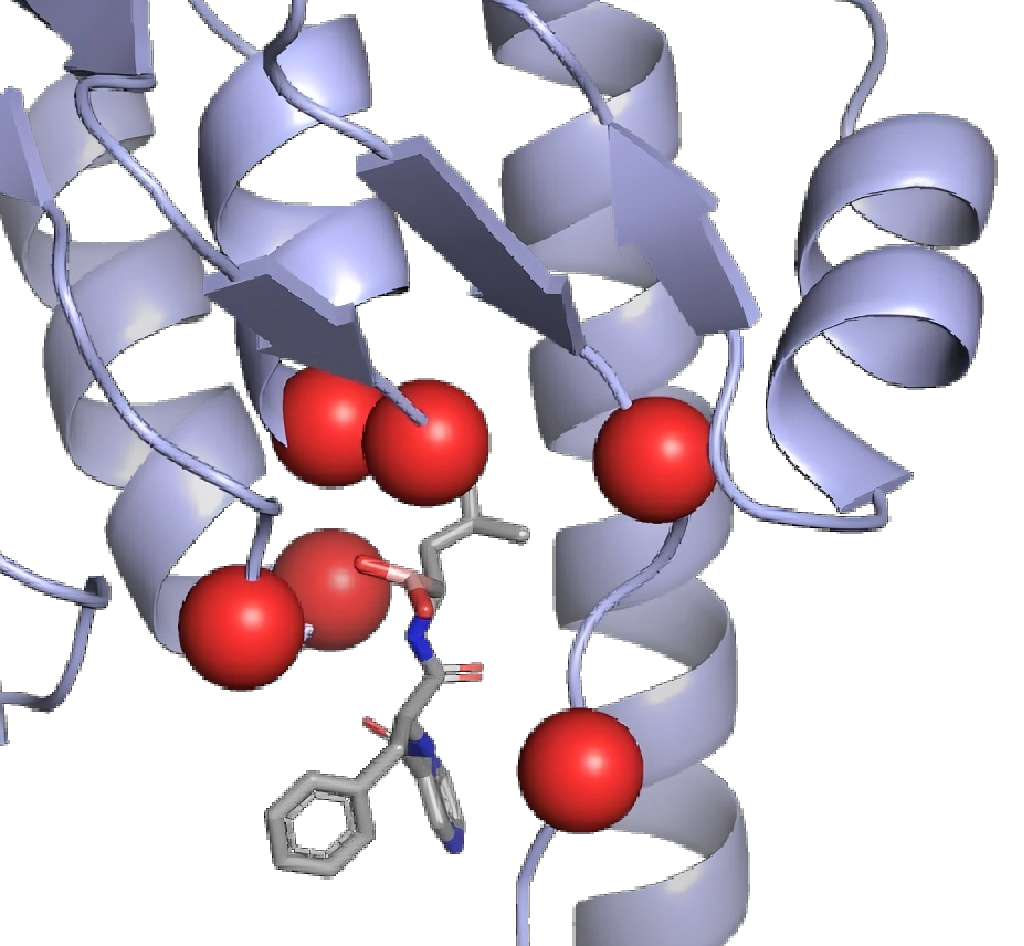

Supplement: S1 File — (ZIP) [file ppat.1013909.s010.zip › Fig 8/Fig 8E.jpg]

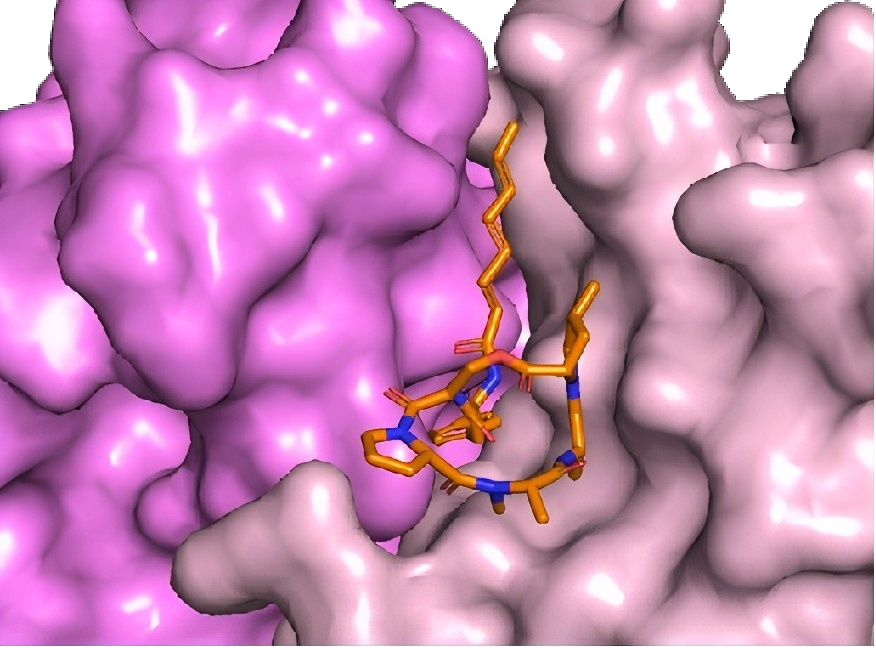

Supplement: S1 File — (ZIP) [file ppat.1013909.s010.zip › Fig 8/Fig 8F-right-1.jpg]

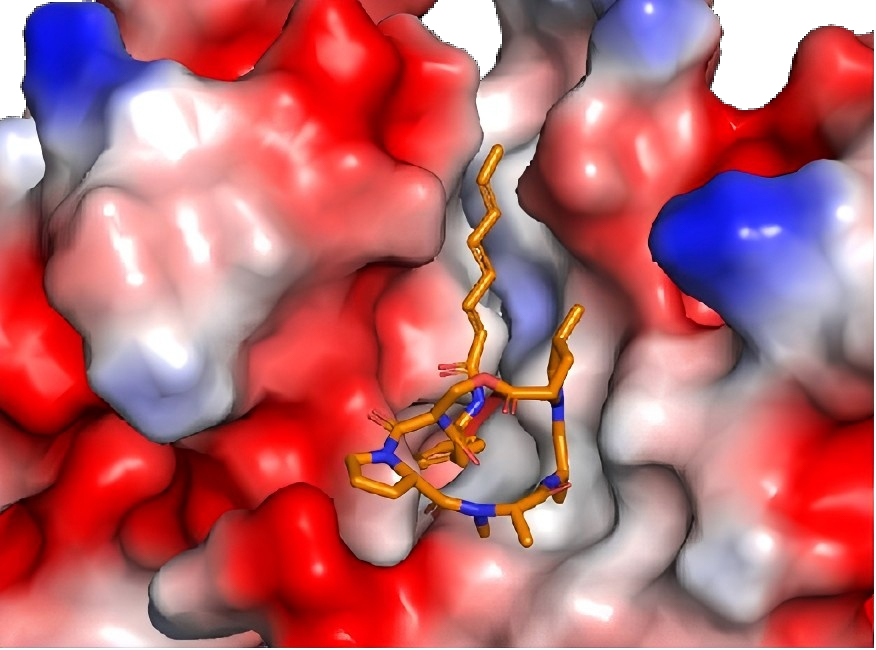

Supplement: S1 File — (ZIP) [file ppat.1013909.s010.zip › Fig 8/Fig 8F-right-2.jpg]

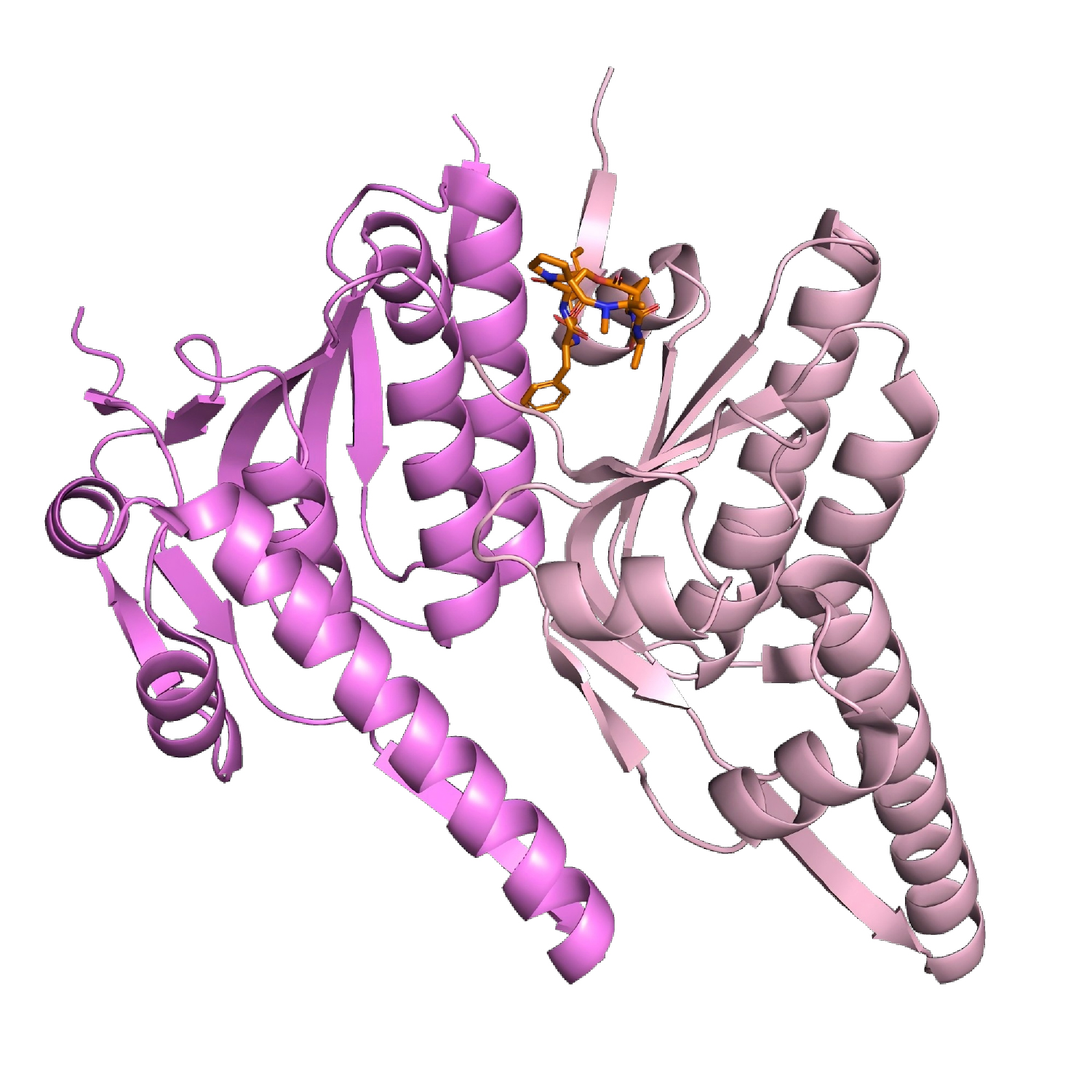

Supplement: S1 File — (ZIP) [file ppat.1013909.s010.zip › Fig 8/Fig 8F.jpg]

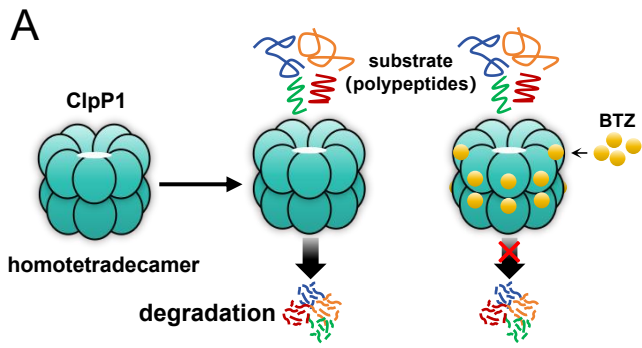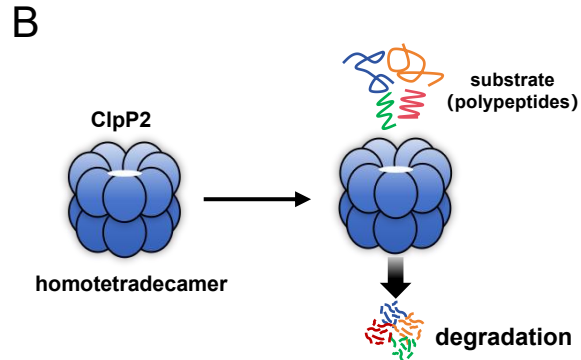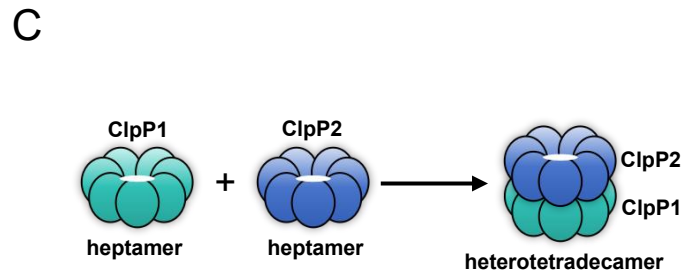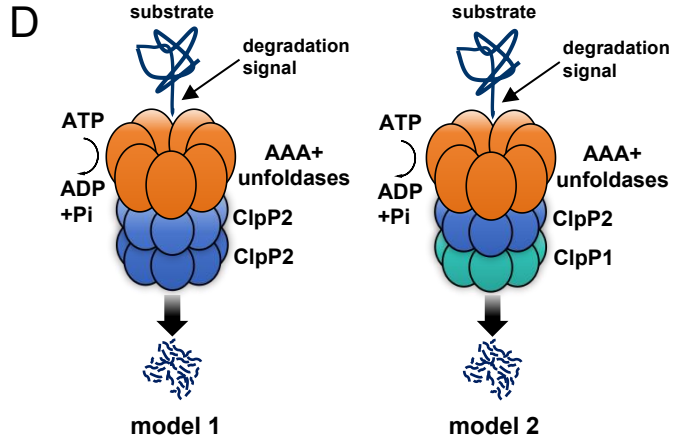

Supplement: S1 File — (ZIP) [file ppat.1013909.s010.zip › Fig 9/Figure 9.pdf]

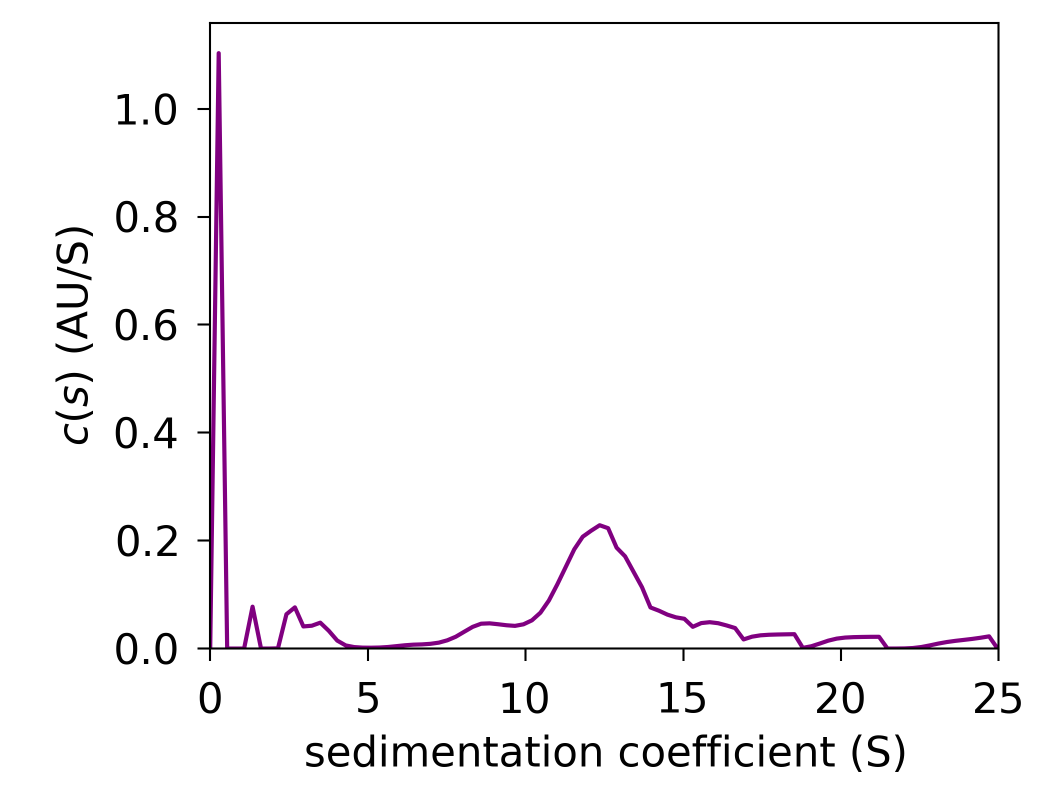

Supplement: S1 File — (ZIP) [file ppat.1013909.s010.zip › S1 Fig/S1C Fig.tif]

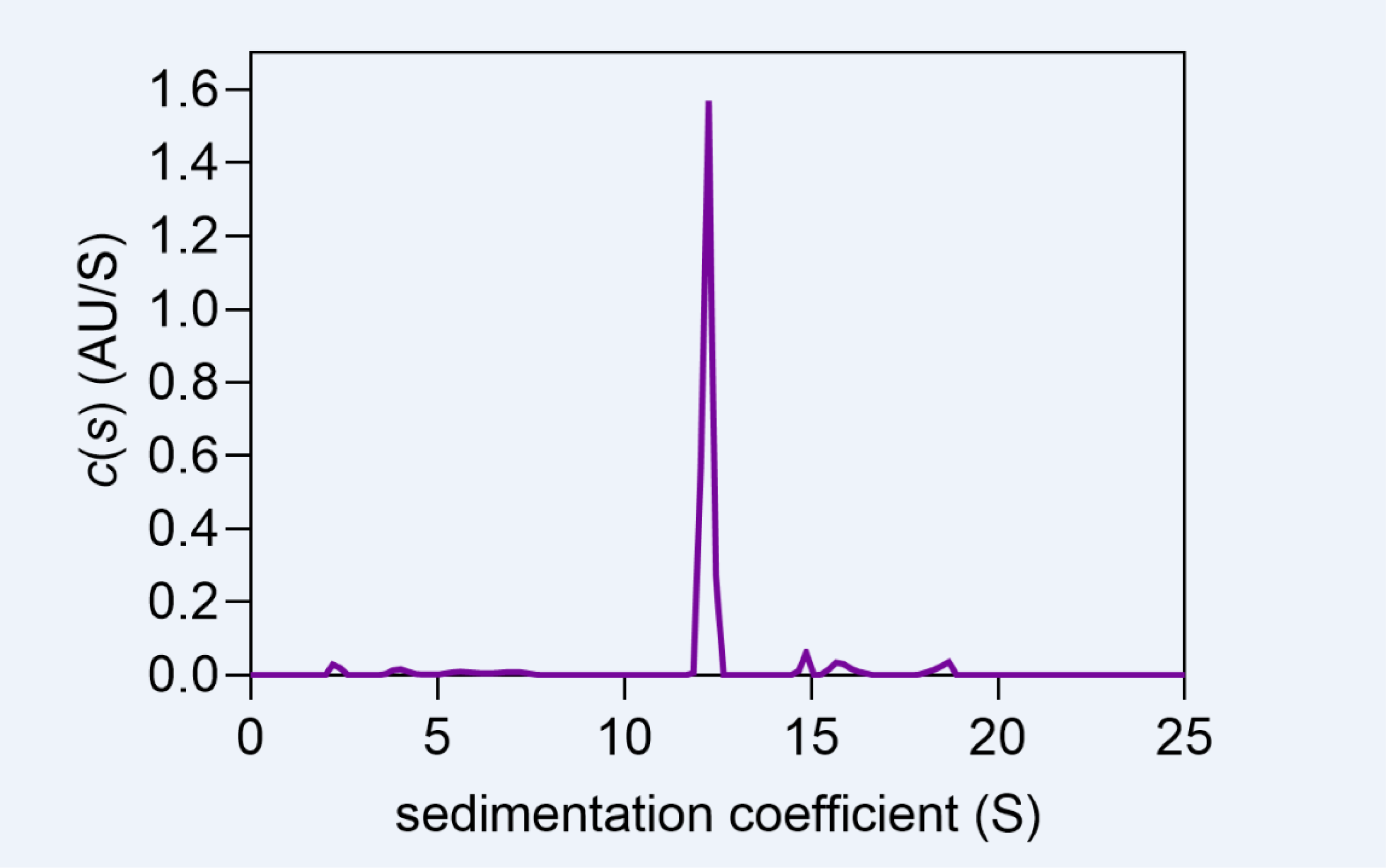

Supplement: S1 File — (ZIP) [file ppat.1013909.s010.zip › S1 Fig/S1D Fig.tif]

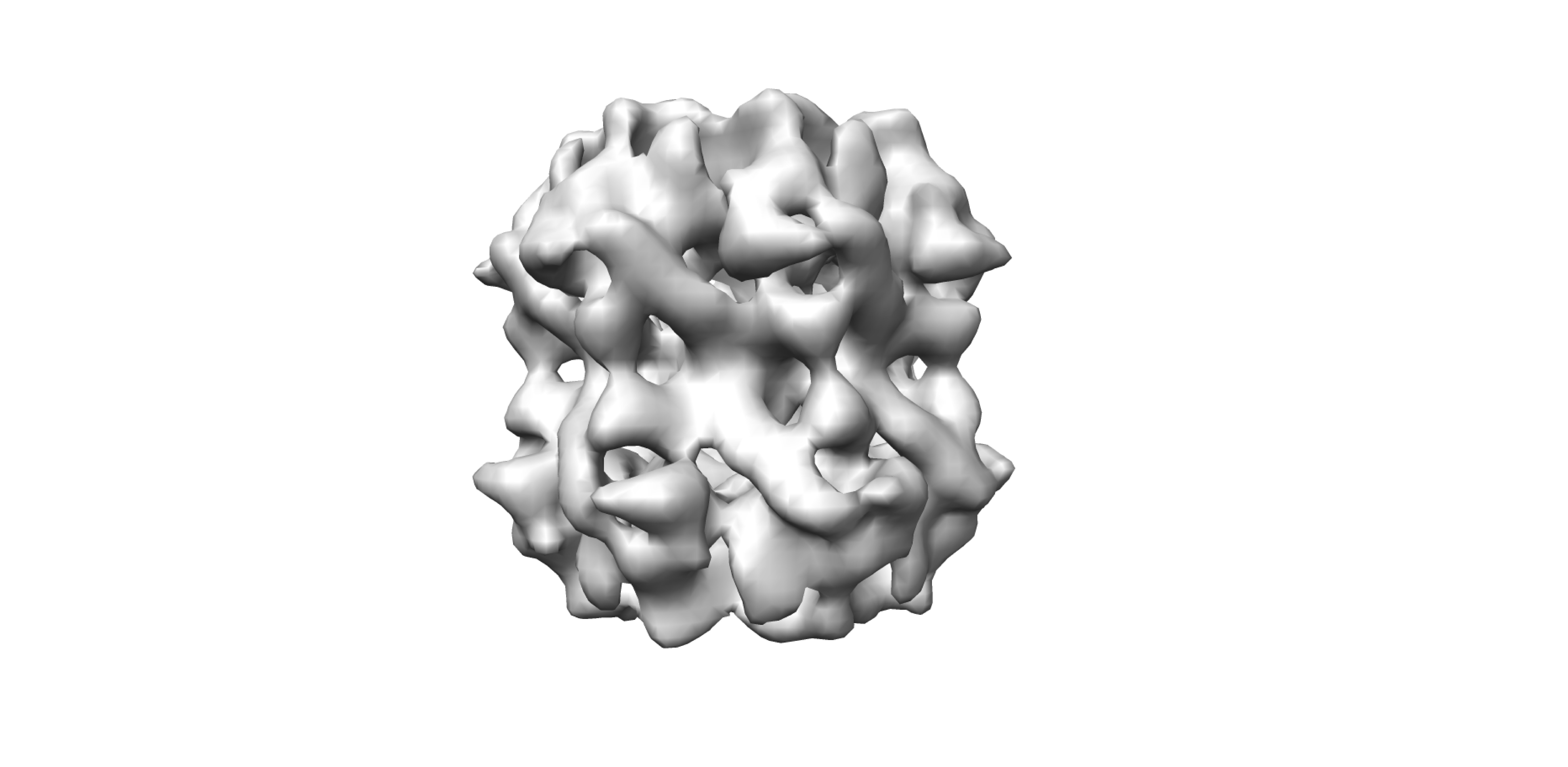

Supplement: S1 File — (ZIP) [file ppat.1013909.s010.zip › S2 Fig/S2A-1 Fig.jpg]

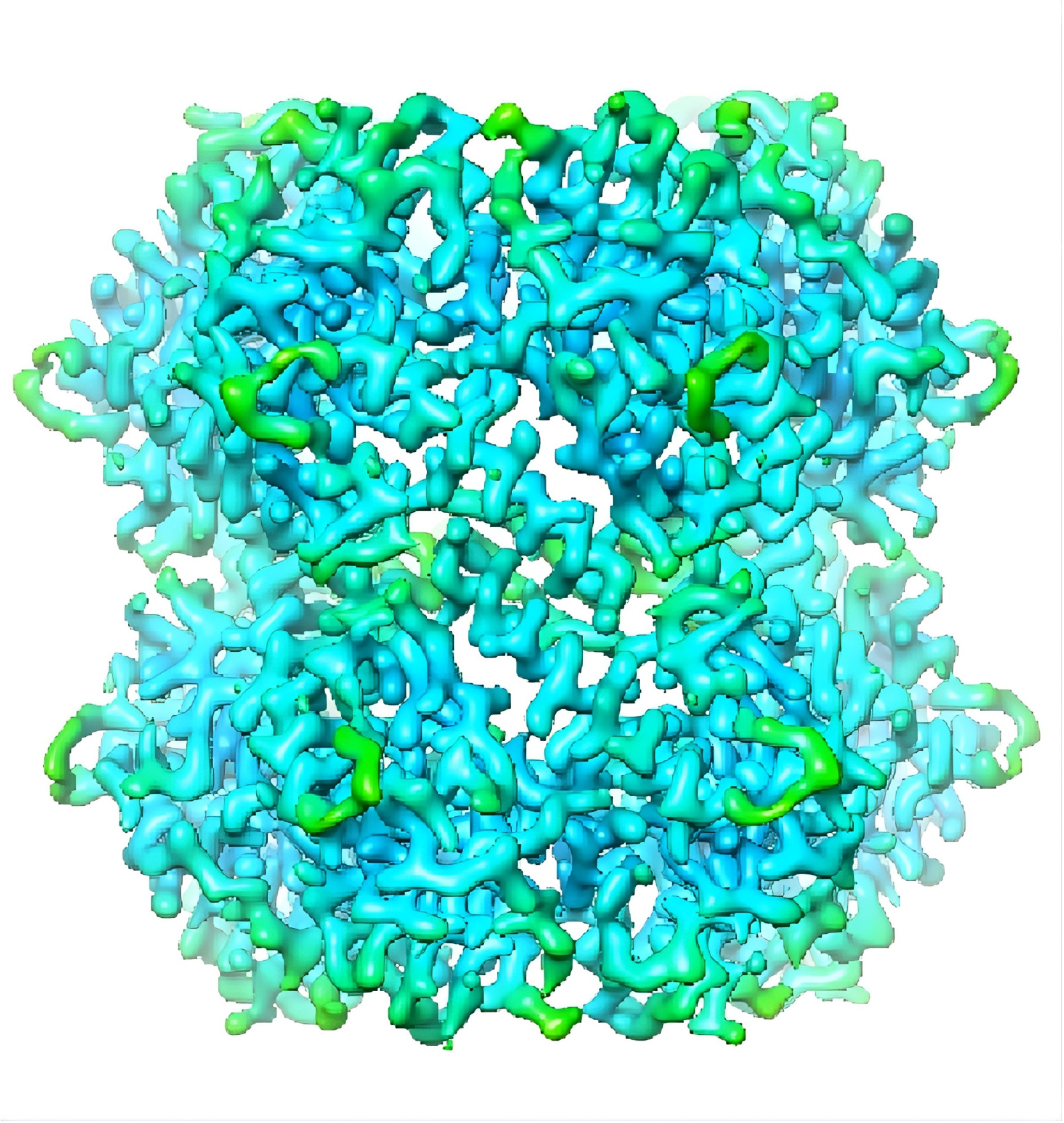

Supplement: S1 File — (ZIP) [file ppat.1013909.s010.zip › S2 Fig/S2A-2 Fig.jpg]

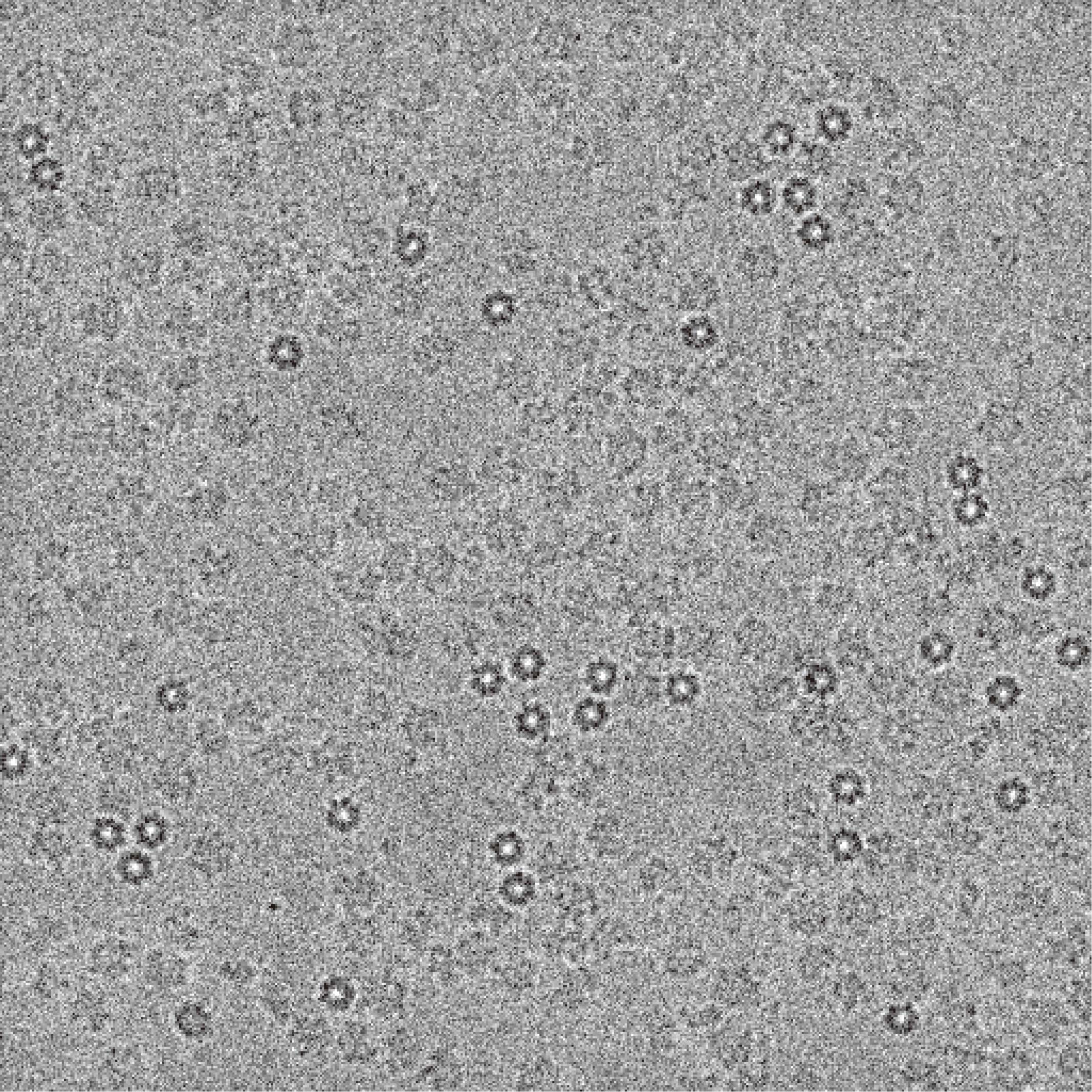

Supplement: S1 File — (ZIP) [file ppat.1013909.s010.zip › S2 Fig/S2B Fig.jpg]

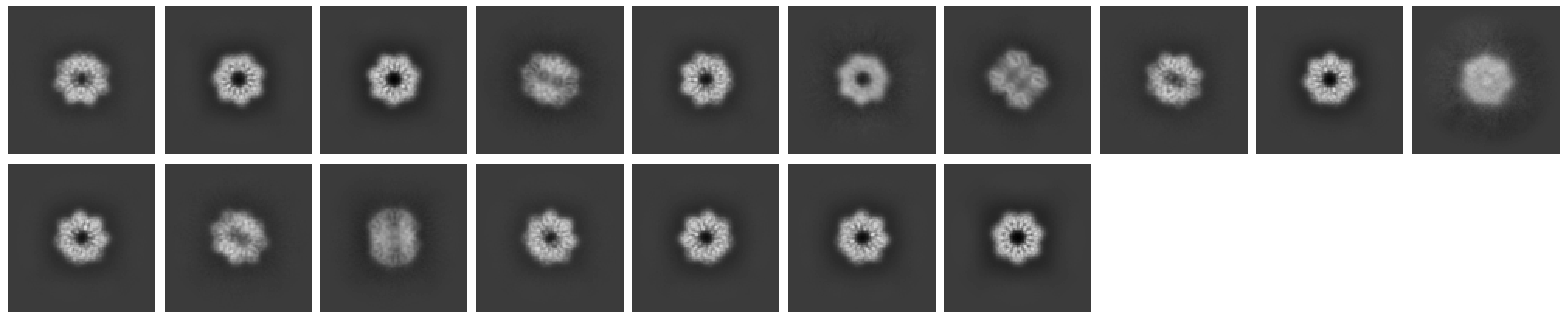

Supplement: S1 File — (ZIP) [file ppat.1013909.s010.zip › S2 Fig/S2C Fig.tif]

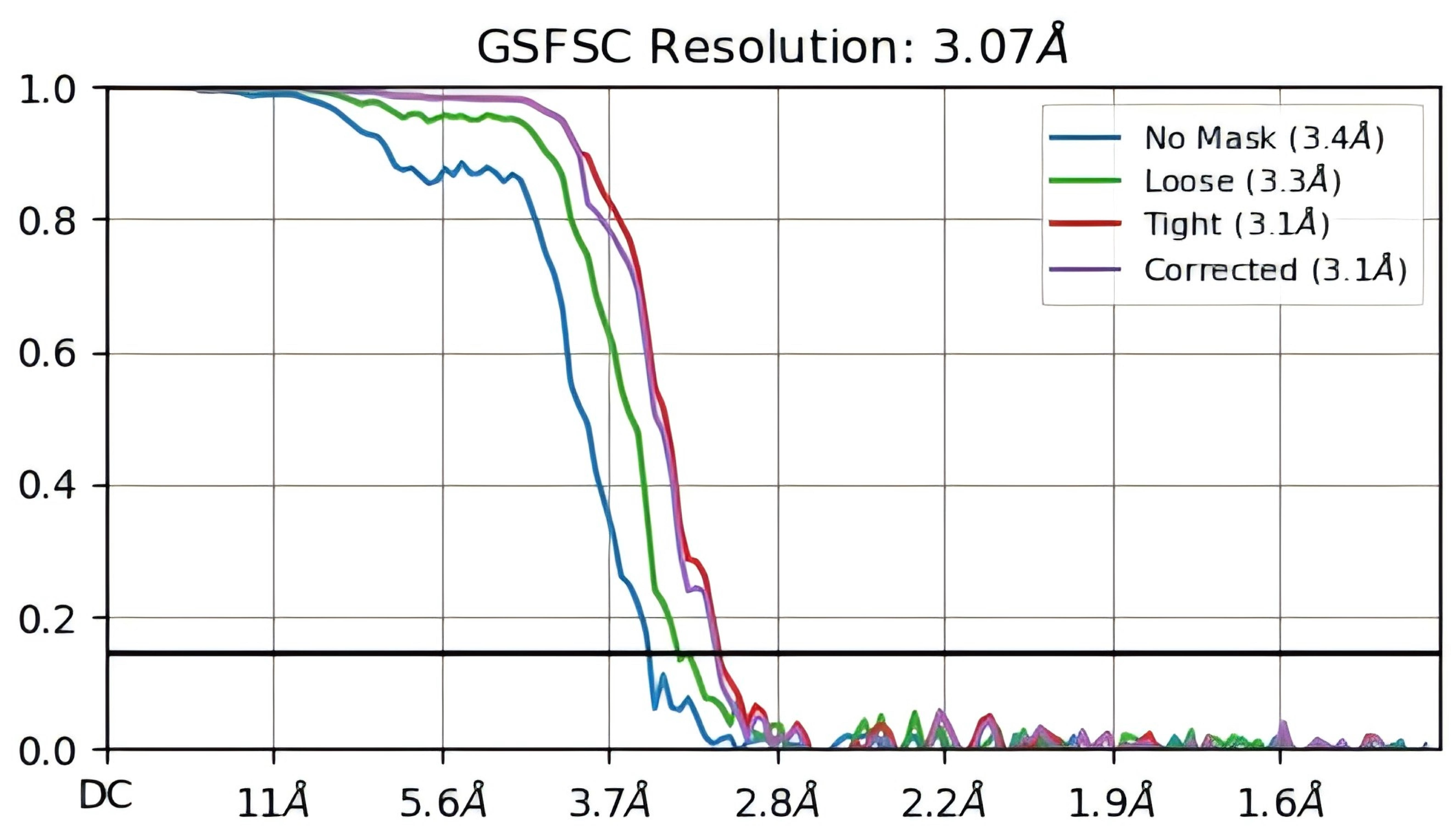

Supplement: S1 File — (ZIP) [file ppat.1013909.s010.zip › S2 Fig/S2D Fig.jpg]

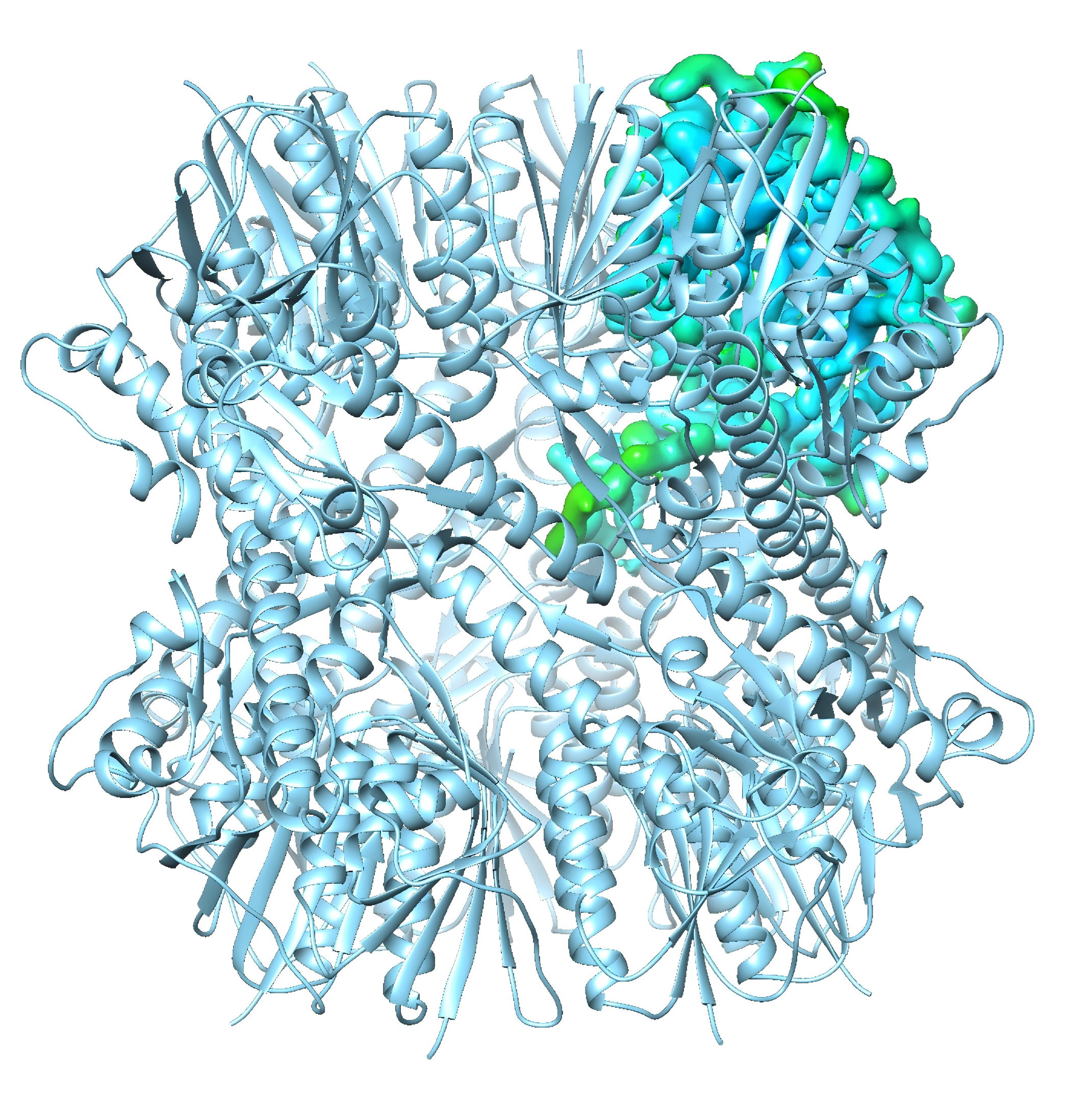

Supplement: S1 File — (ZIP) [file ppat.1013909.s010.zip › S2 Fig/S2E Fig.jpg]

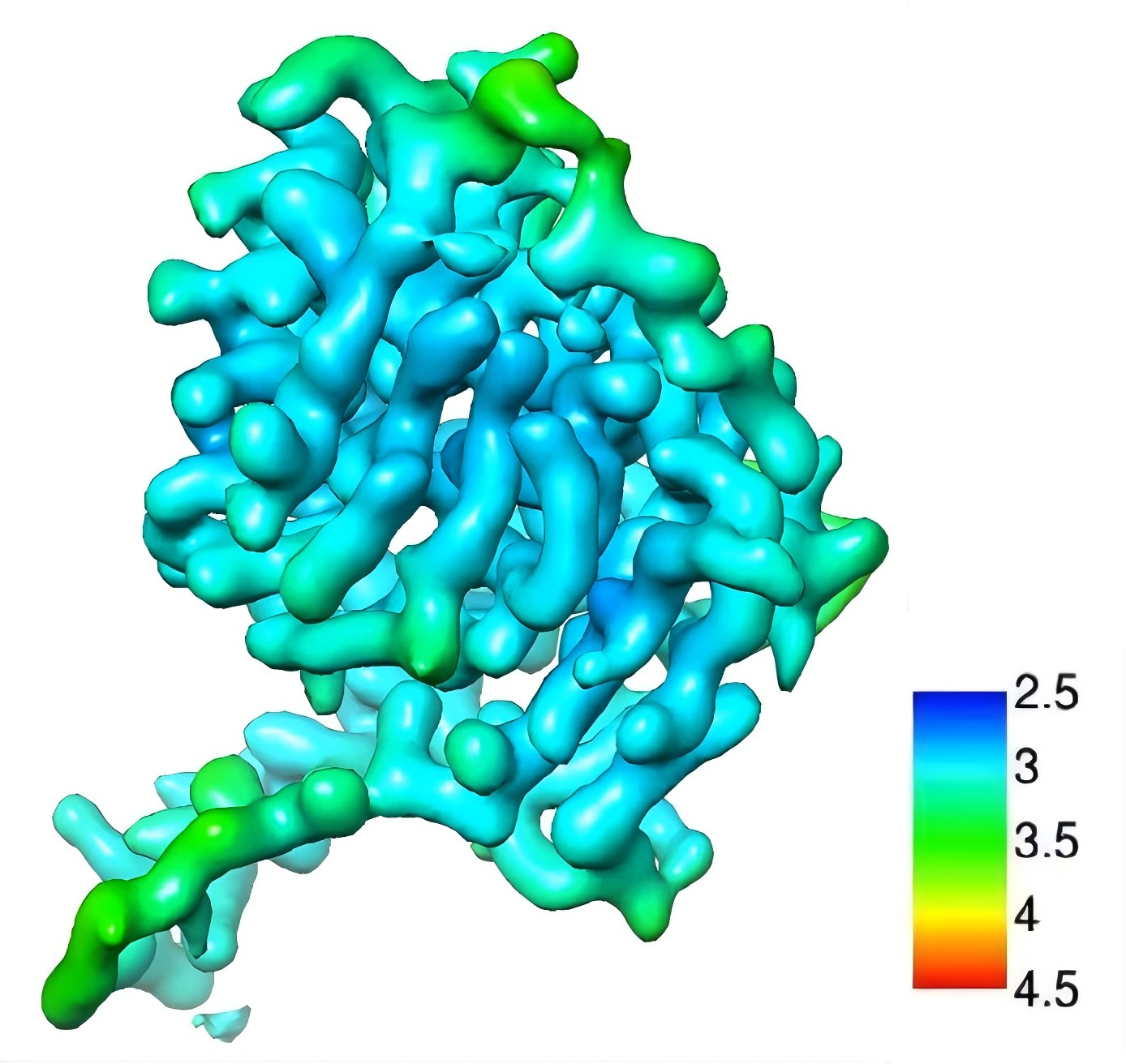

Supplement: S1 File — (ZIP) [file ppat.1013909.s010.zip › S2 Fig/S2F Fig.jpg]

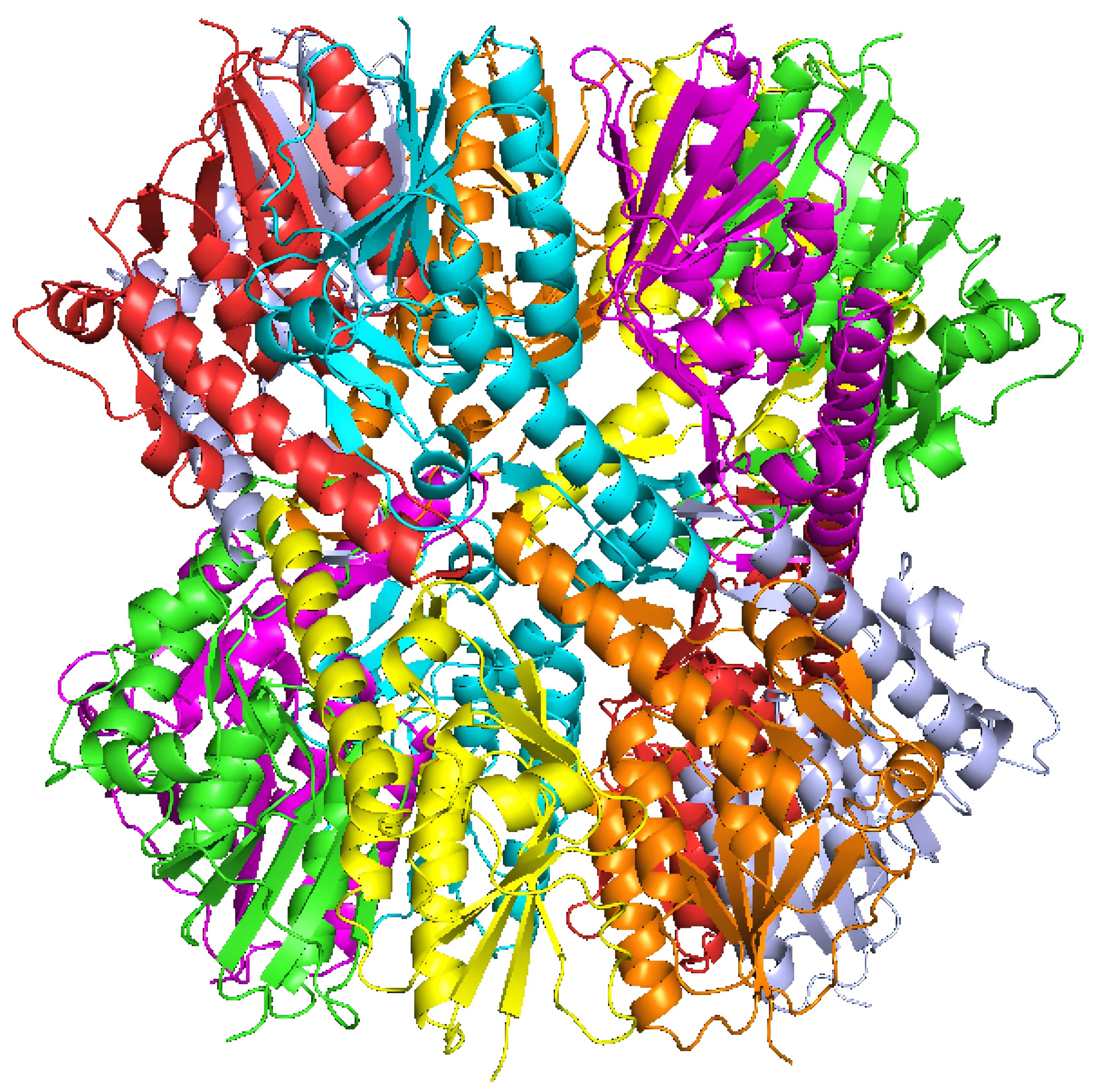

Supplement: S1 File — (ZIP) [file ppat.1013909.s010.zip › S3 Fig/S3A Fig.jpg]

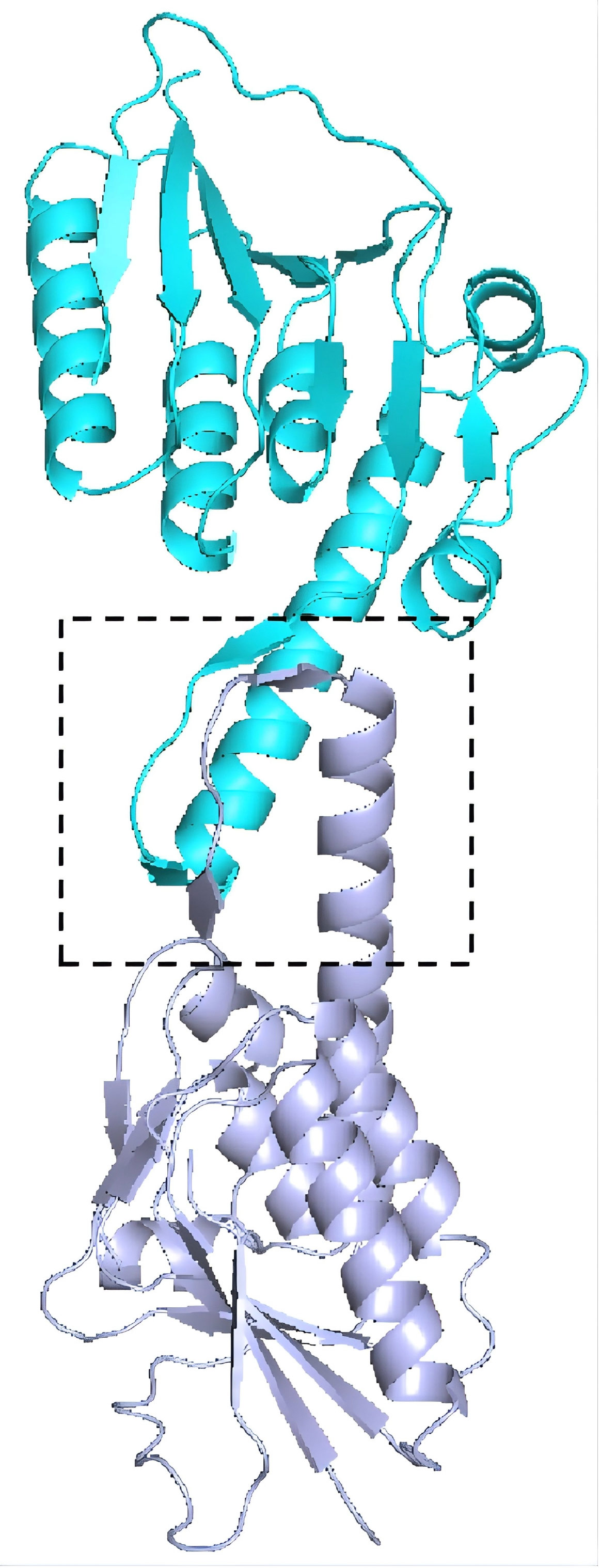

Supplement: S1 File — (ZIP) [file ppat.1013909.s010.zip › S3 Fig/S3B-a Fig.jpg]

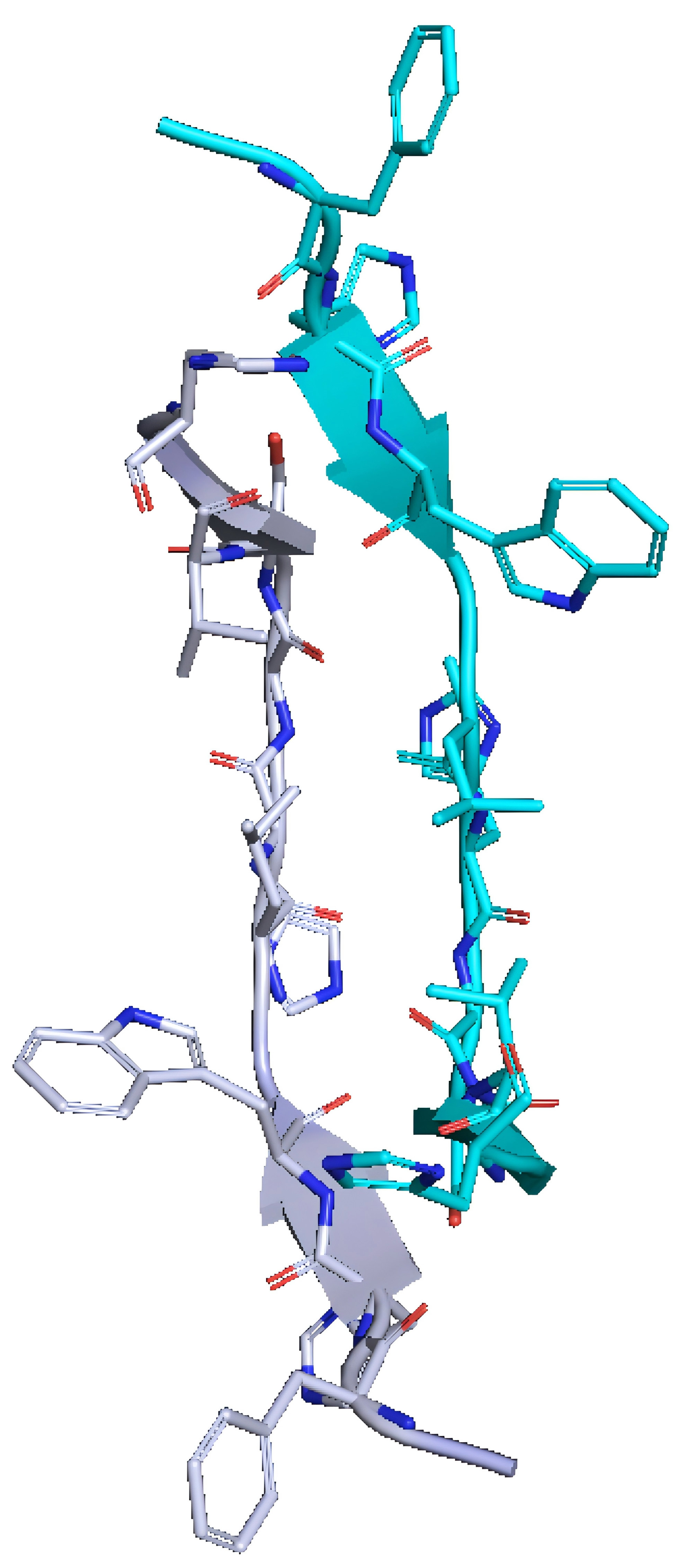

Supplement: S1 File — (ZIP) [file ppat.1013909.s010.zip › S3 Fig/S3B-b Fig.jpg]

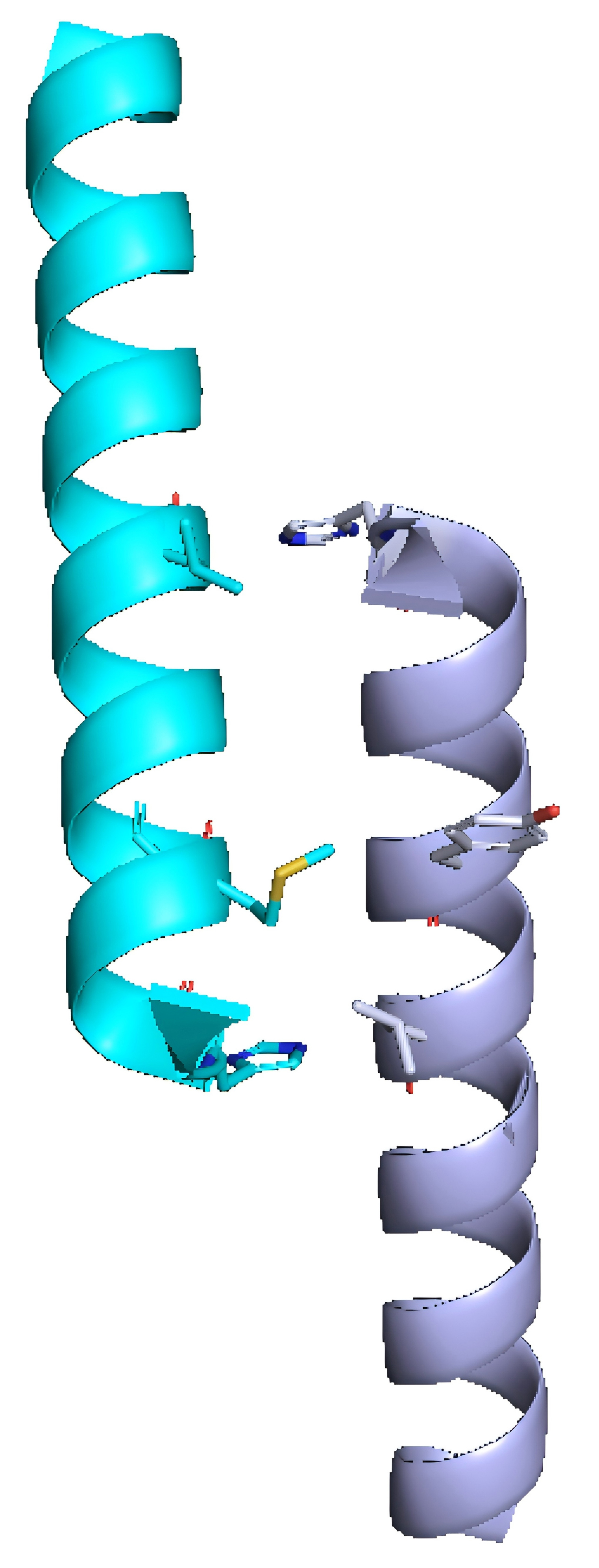

Supplement: S1 File — (ZIP) [file ppat.1013909.s010.zip › S3 Fig/S3B-c Fig.jpg]

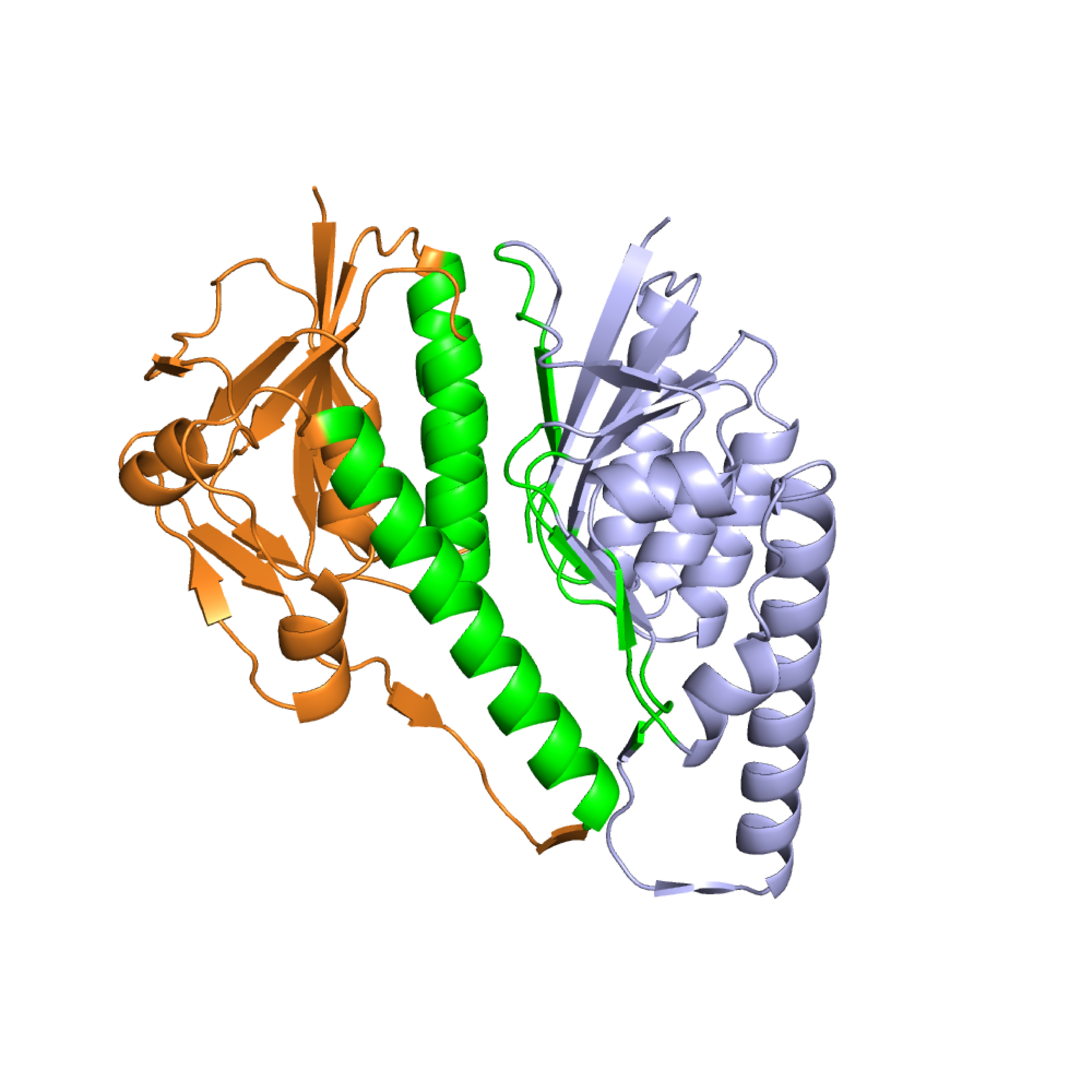

Supplement: S1 File — (ZIP) [file ppat.1013909.s010.zip › S3 Fig/S3C-a Fig.jpg]

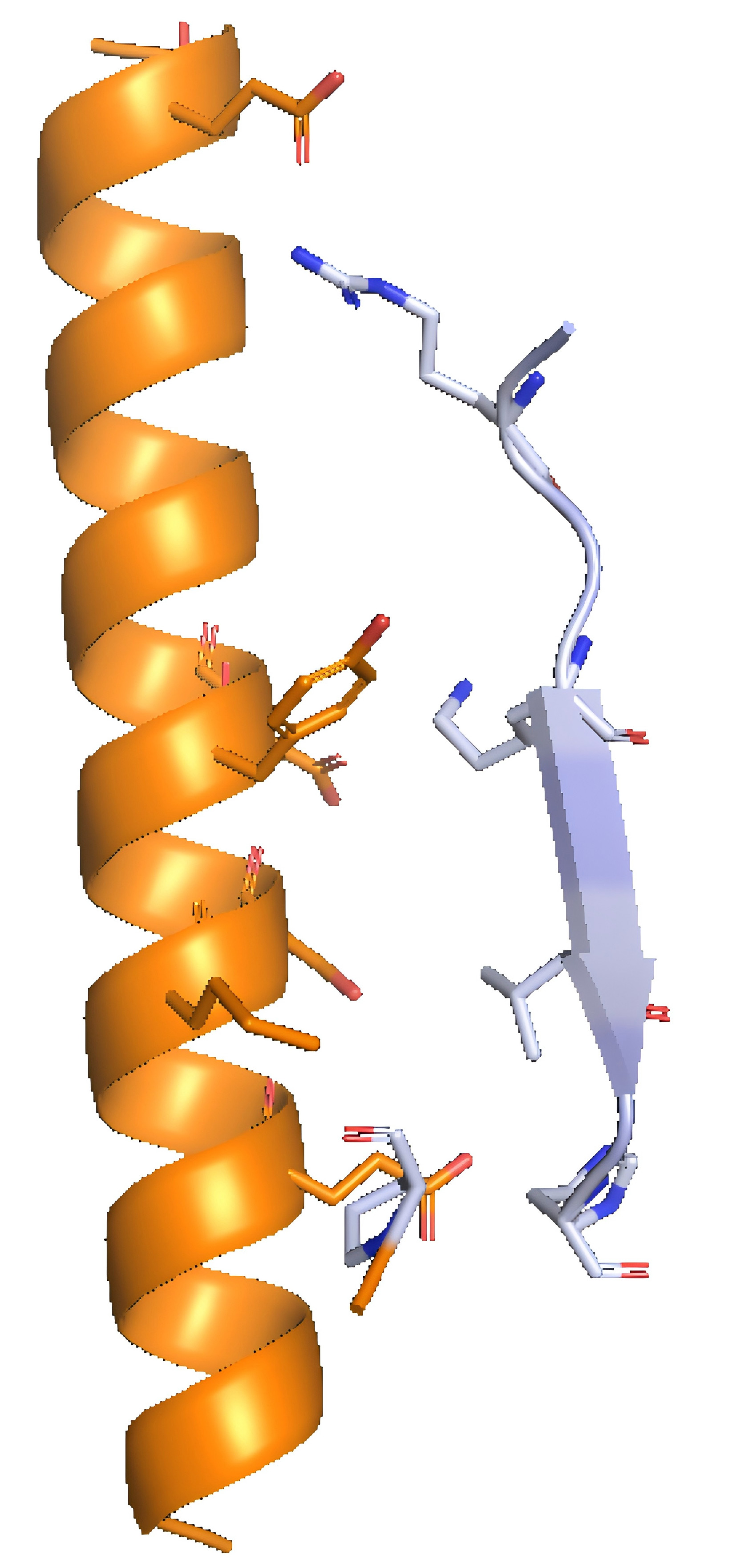

Supplement: S1 File — (ZIP) [file ppat.1013909.s010.zip › S3 Fig/S3C-b Fig.jpg]

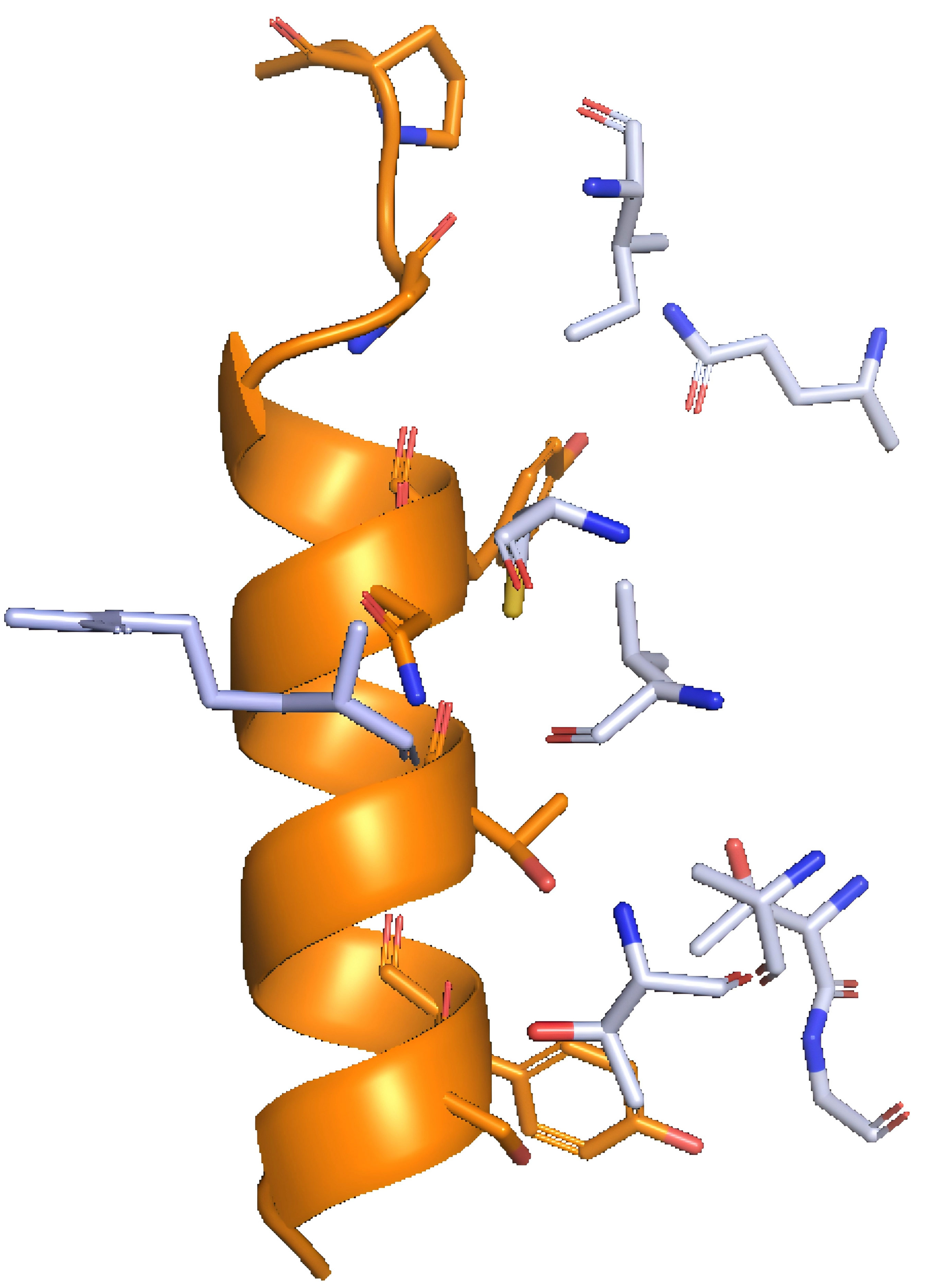

Supplement: S1 File — (ZIP) [file ppat.1013909.s010.zip › S3 Fig/S3C-c Fig.jpg]

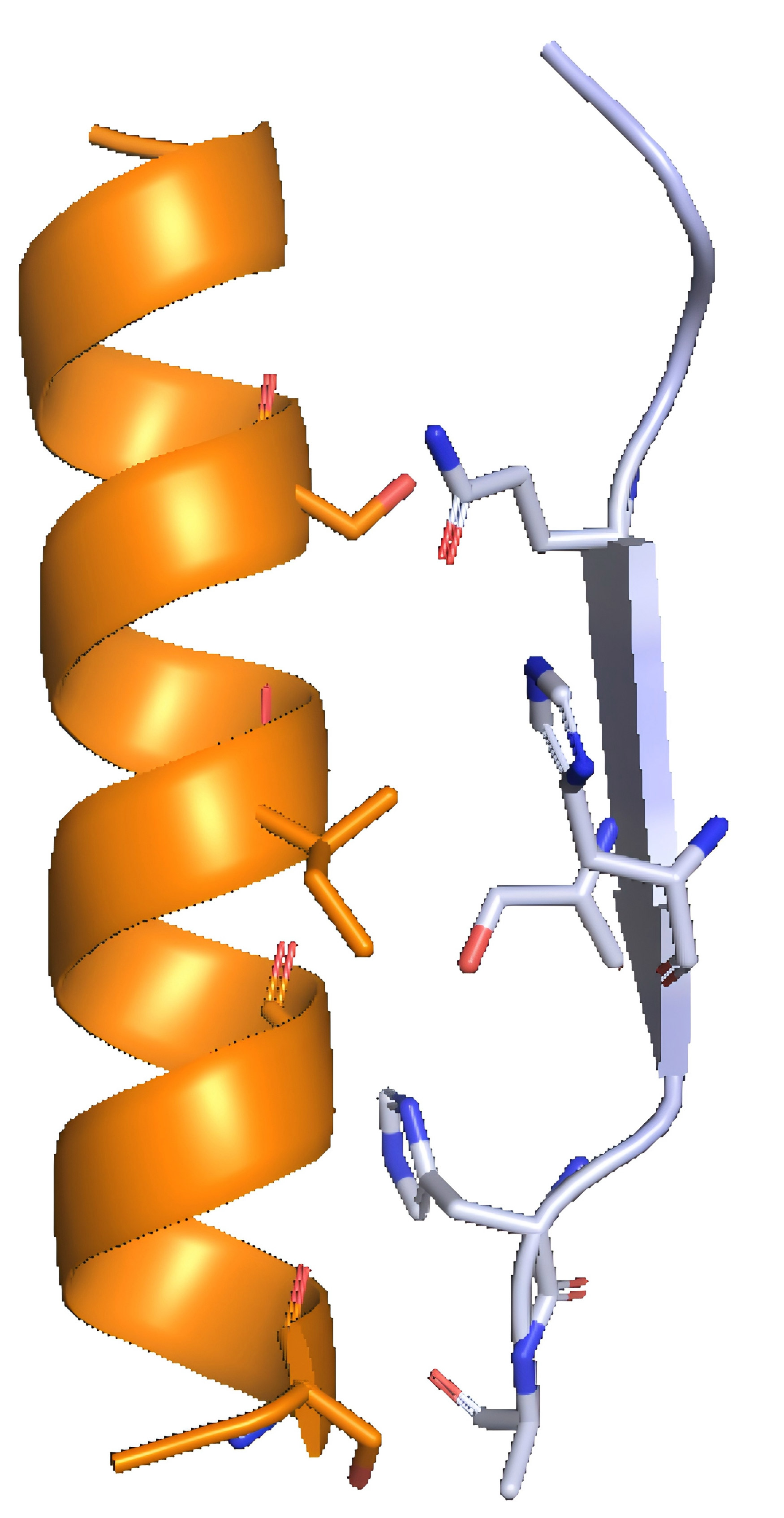

Supplement: S1 File — (ZIP) [file ppat.1013909.s010.zip › S3 Fig/S3C-d Fig.jpg]

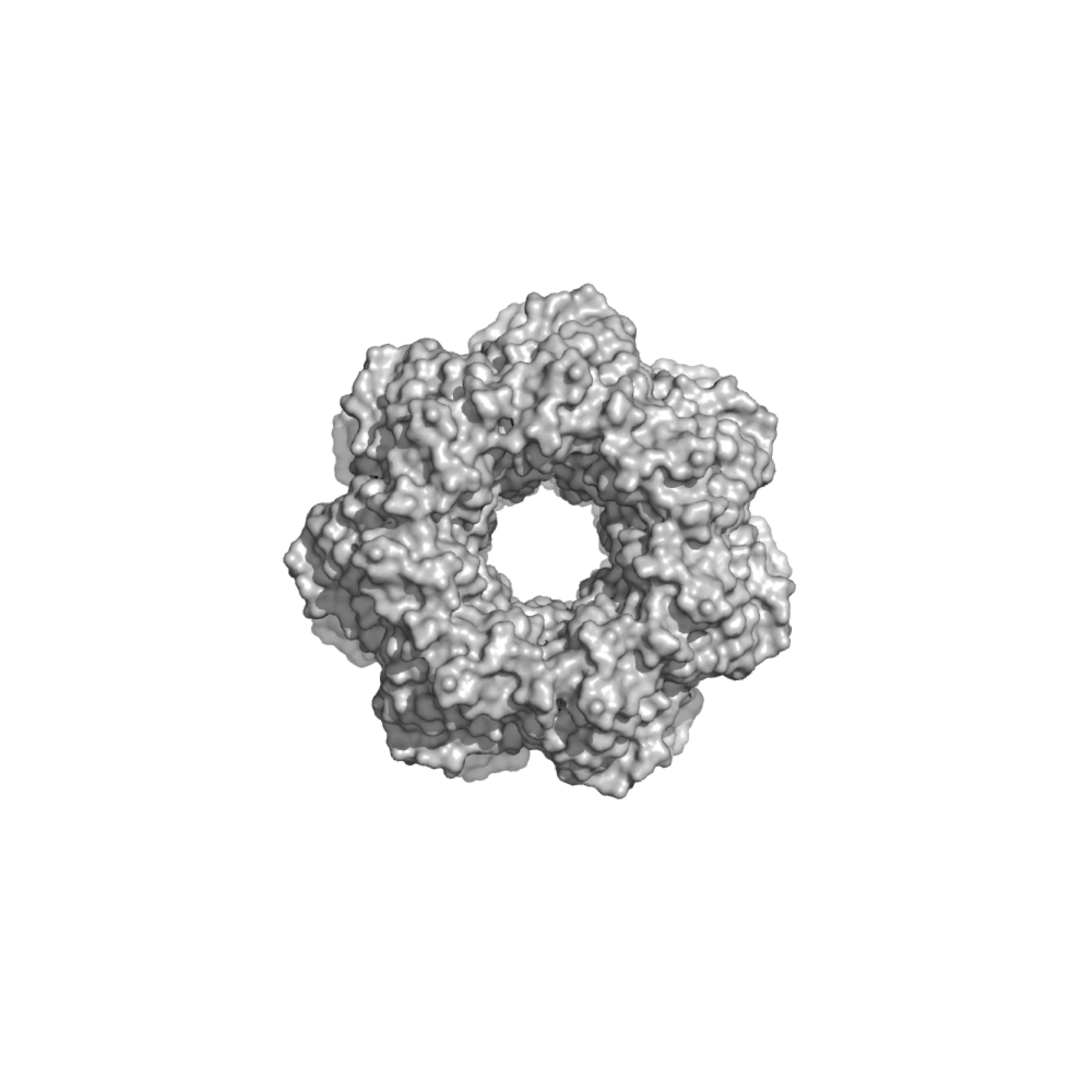

Supplement: S1 File — (ZIP) [file ppat.1013909.s010.zip › S4 Fig/S4A-a Fig.jpg]

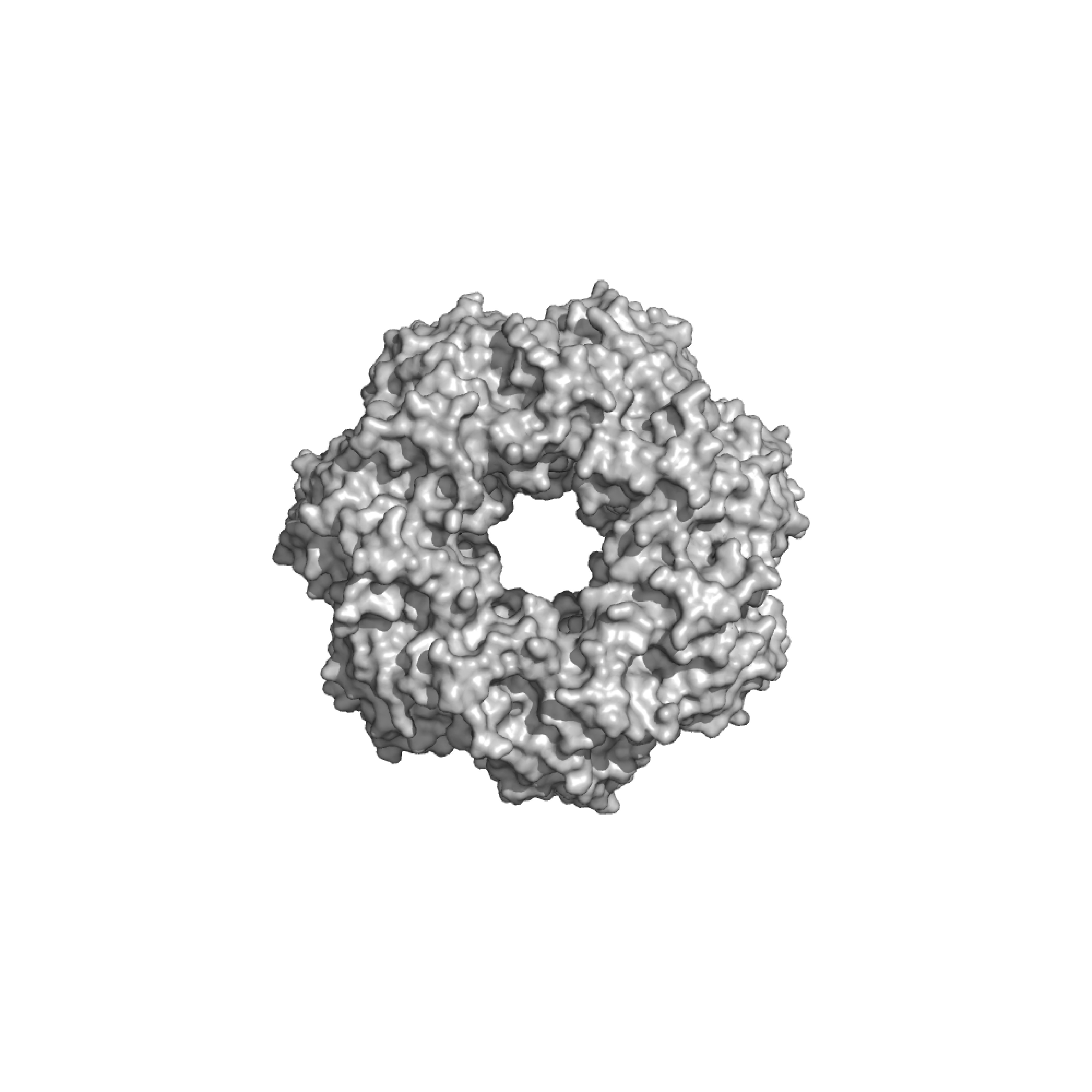

Supplement: S1 File — (ZIP) [file ppat.1013909.s010.zip › S4 Fig/S4A-b Fig.jpg]

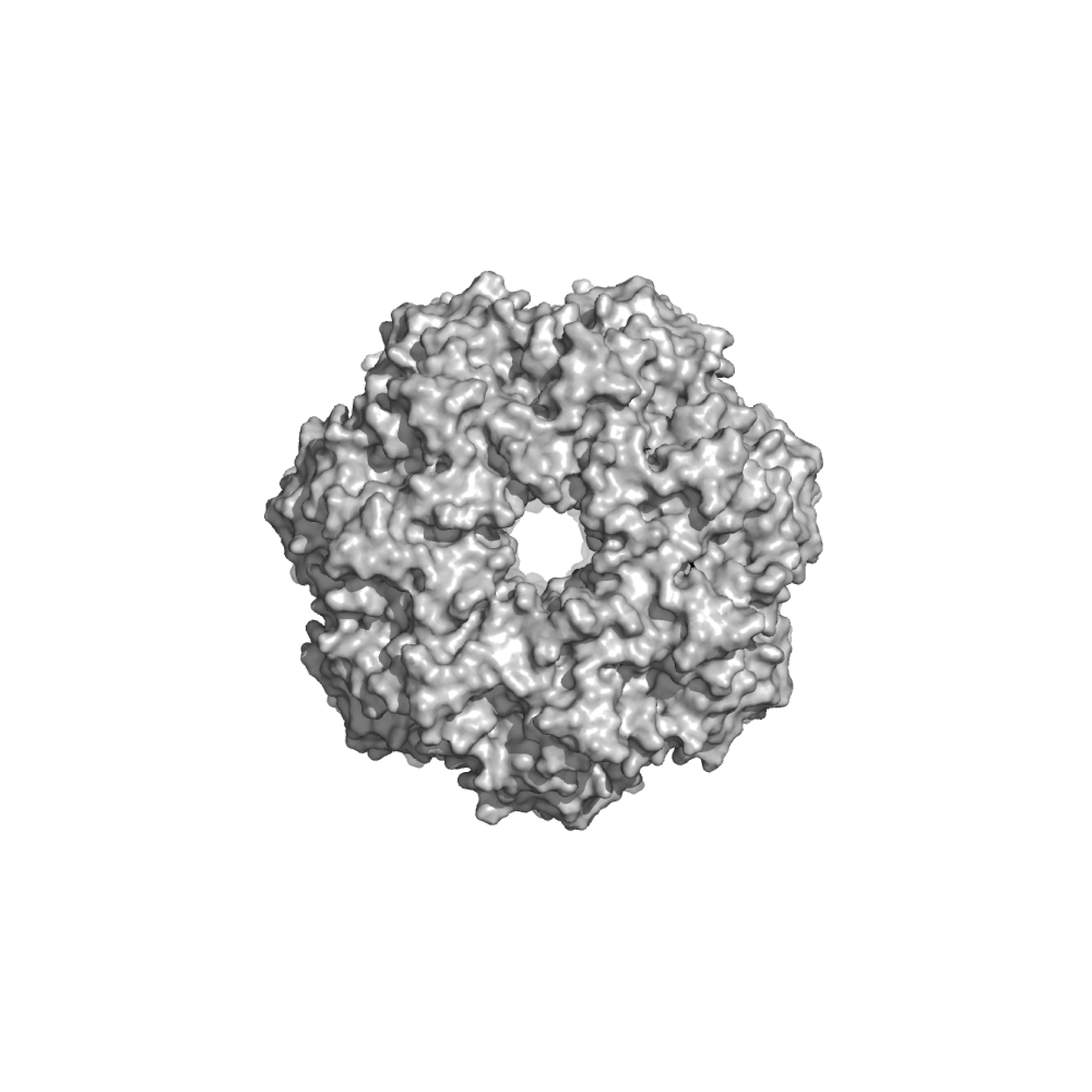

Supplement: S1 File — (ZIP) [file ppat.1013909.s010.zip › S4 Fig/S4A-c Fig.jpg]

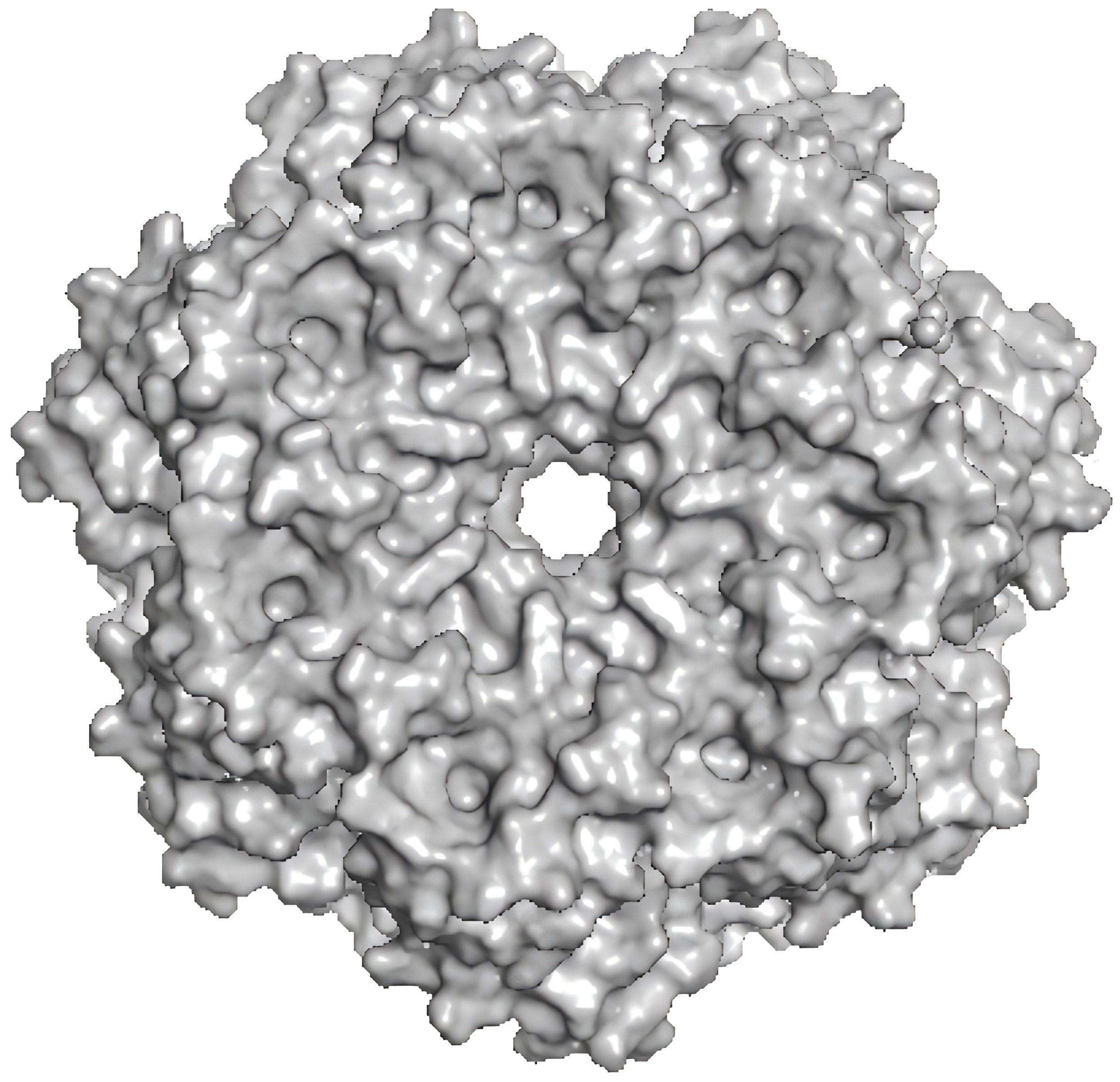

Supplement: S1 File — (ZIP) [file ppat.1013909.s010.zip › S4 Fig/S4A-d Fig.jpg]

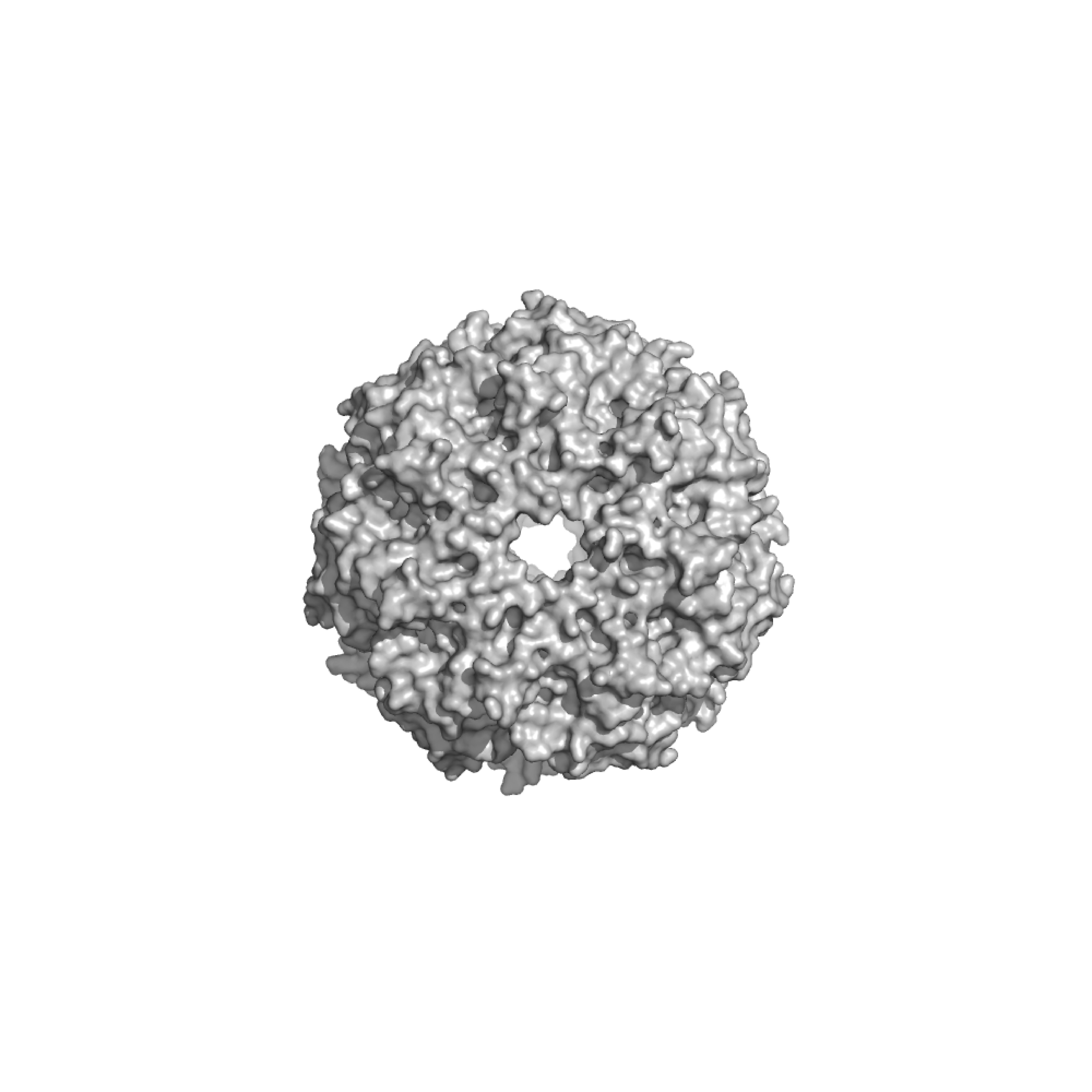

Supplement: S1 File — (ZIP) [file ppat.1013909.s010.zip › S4 Fig/S4A-e Fig.jpg]

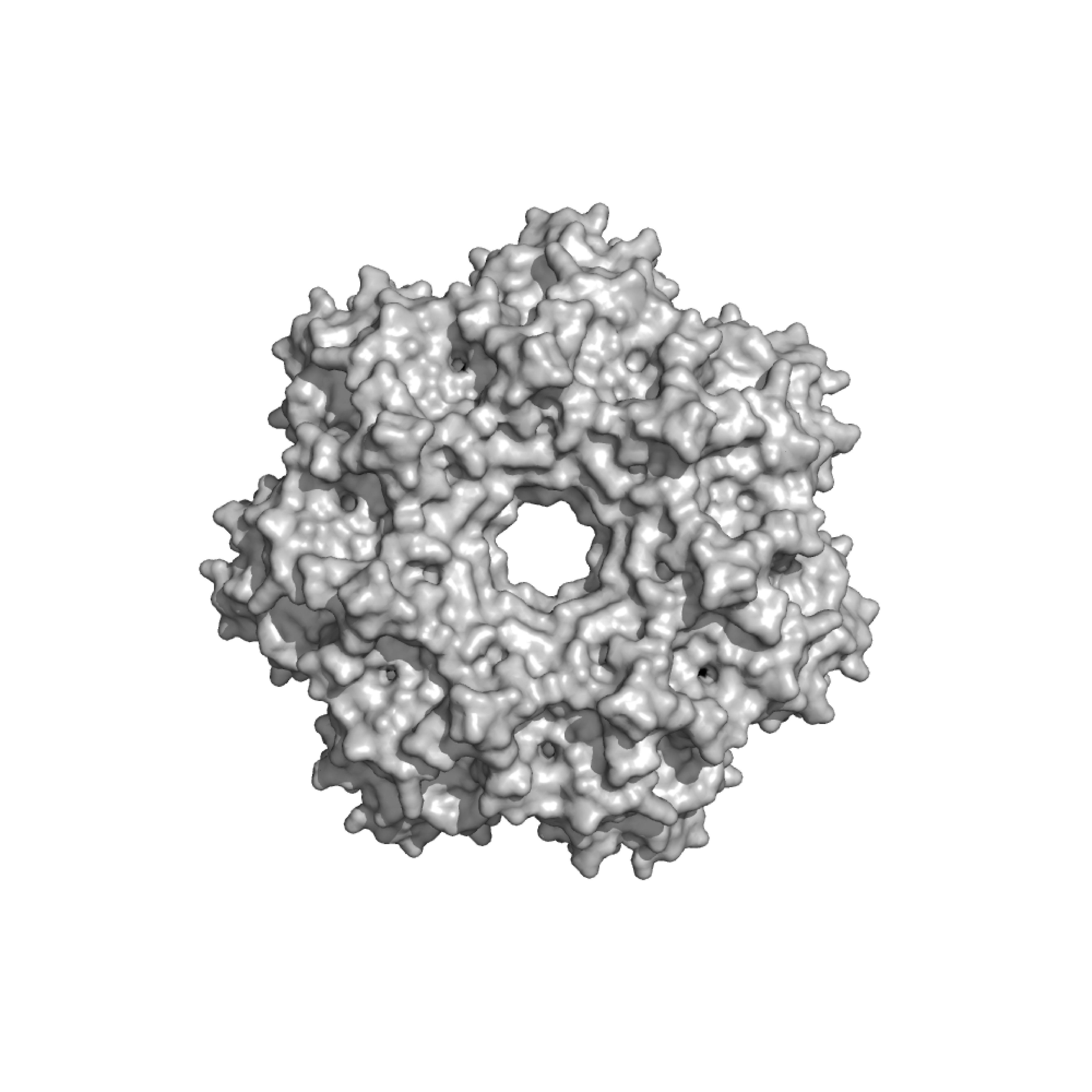

Supplement: S1 File — (ZIP) [file ppat.1013909.s010.zip › S4 Fig/S4A-f Fig.jpg]

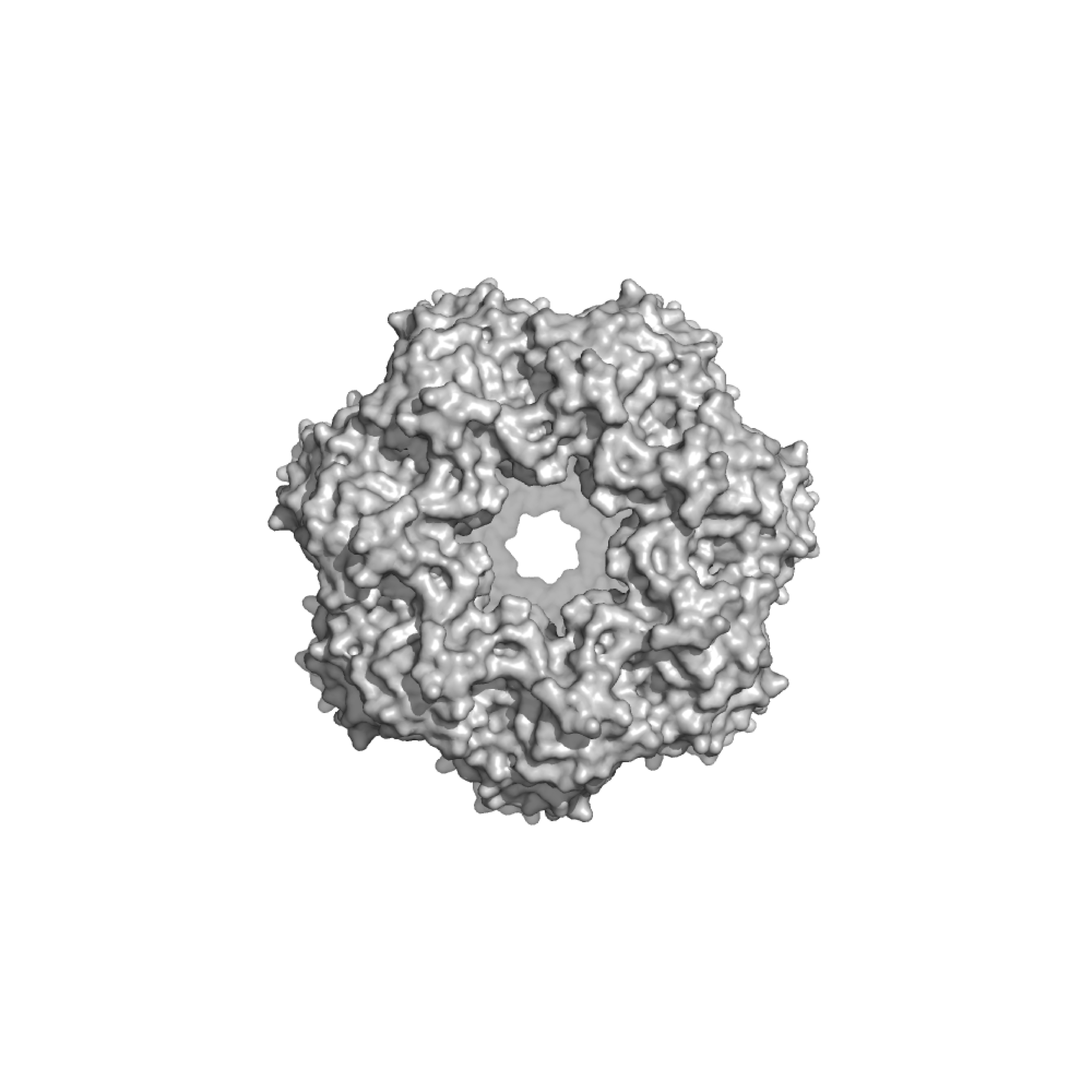

Supplement: S1 File — (ZIP) [file ppat.1013909.s010.zip › S4 Fig/S4A-g Fig.jpg]

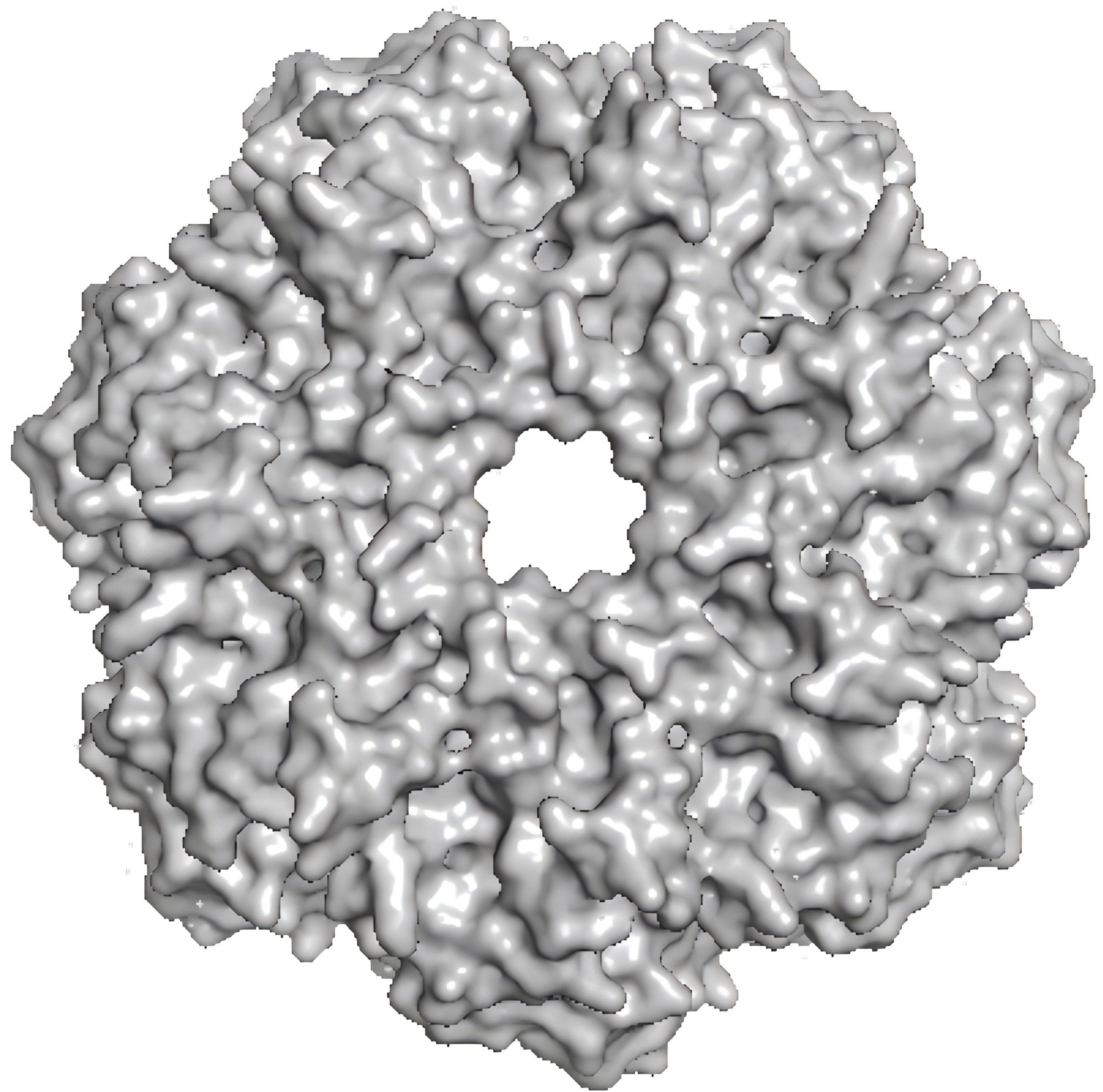

Supplement: S1 File — (ZIP) [file ppat.1013909.s010.zip › S4 Fig/S4A-h Fig.jpg]

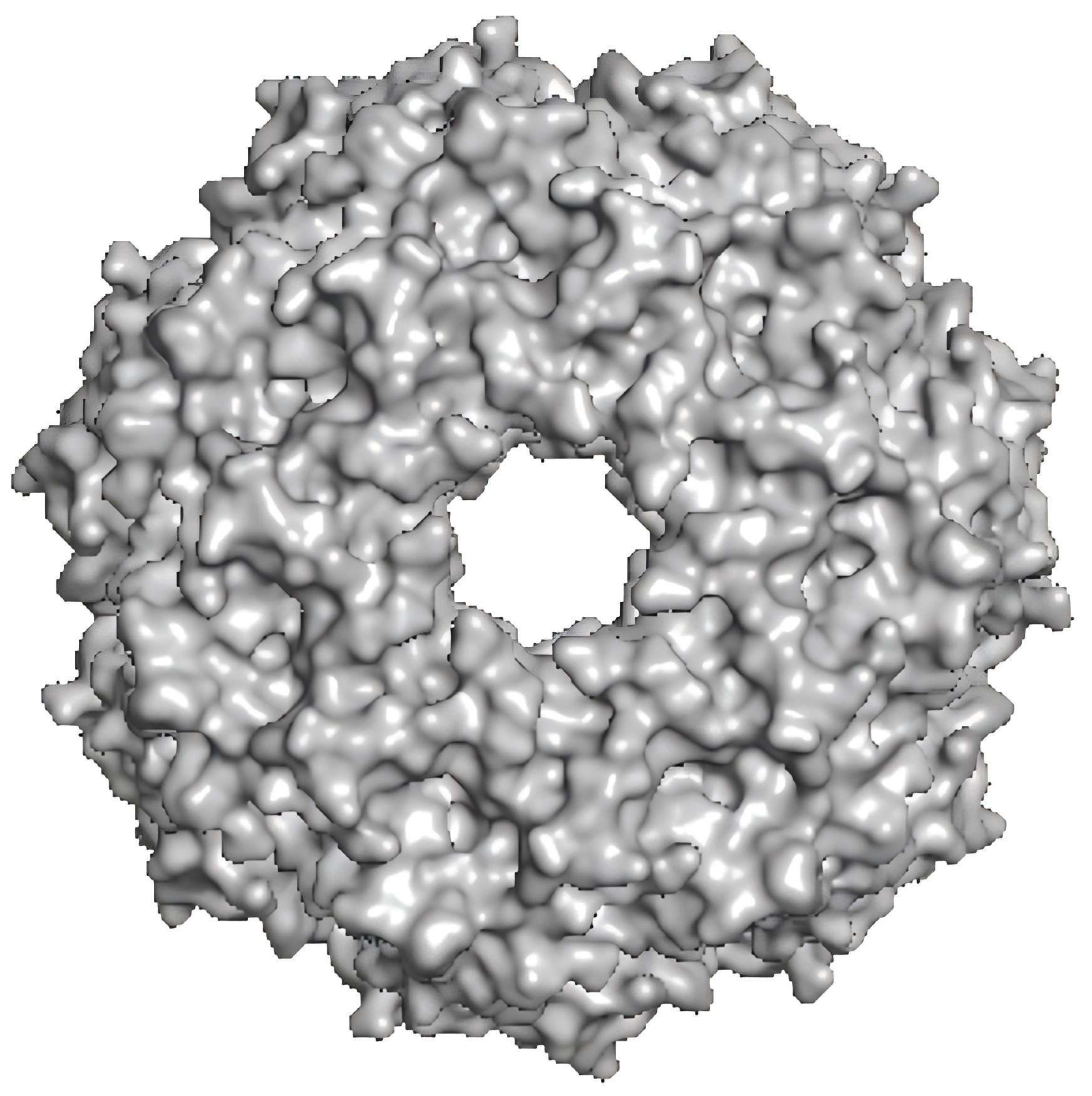

Supplement: S1 File — (ZIP) [file ppat.1013909.s010.zip › S4 Fig/S4A-i Fig.jpg]

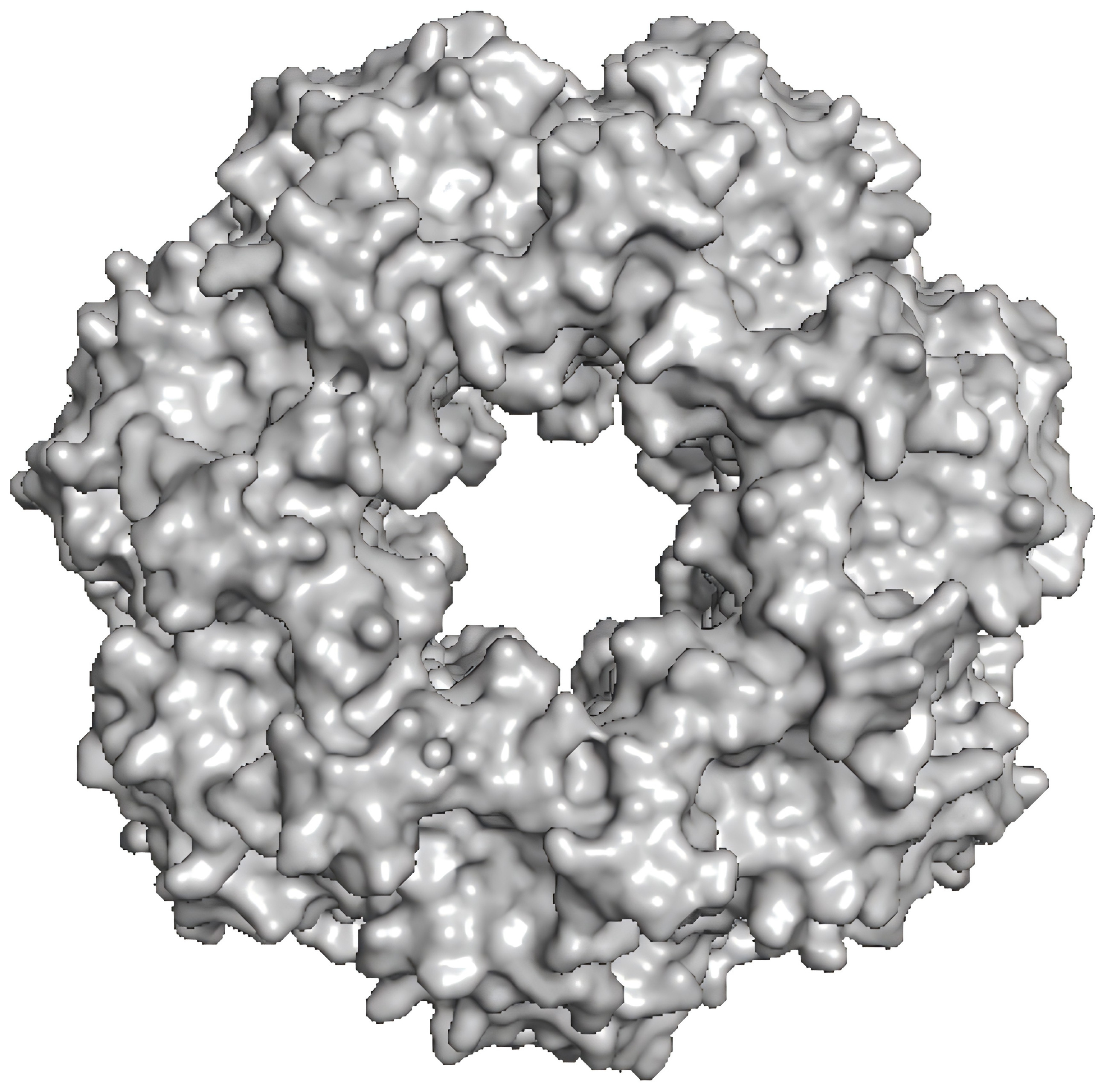

Supplement: S1 File — (ZIP) [file ppat.1013909.s010.zip › S4 Fig/S4A-j Fig.jpg]

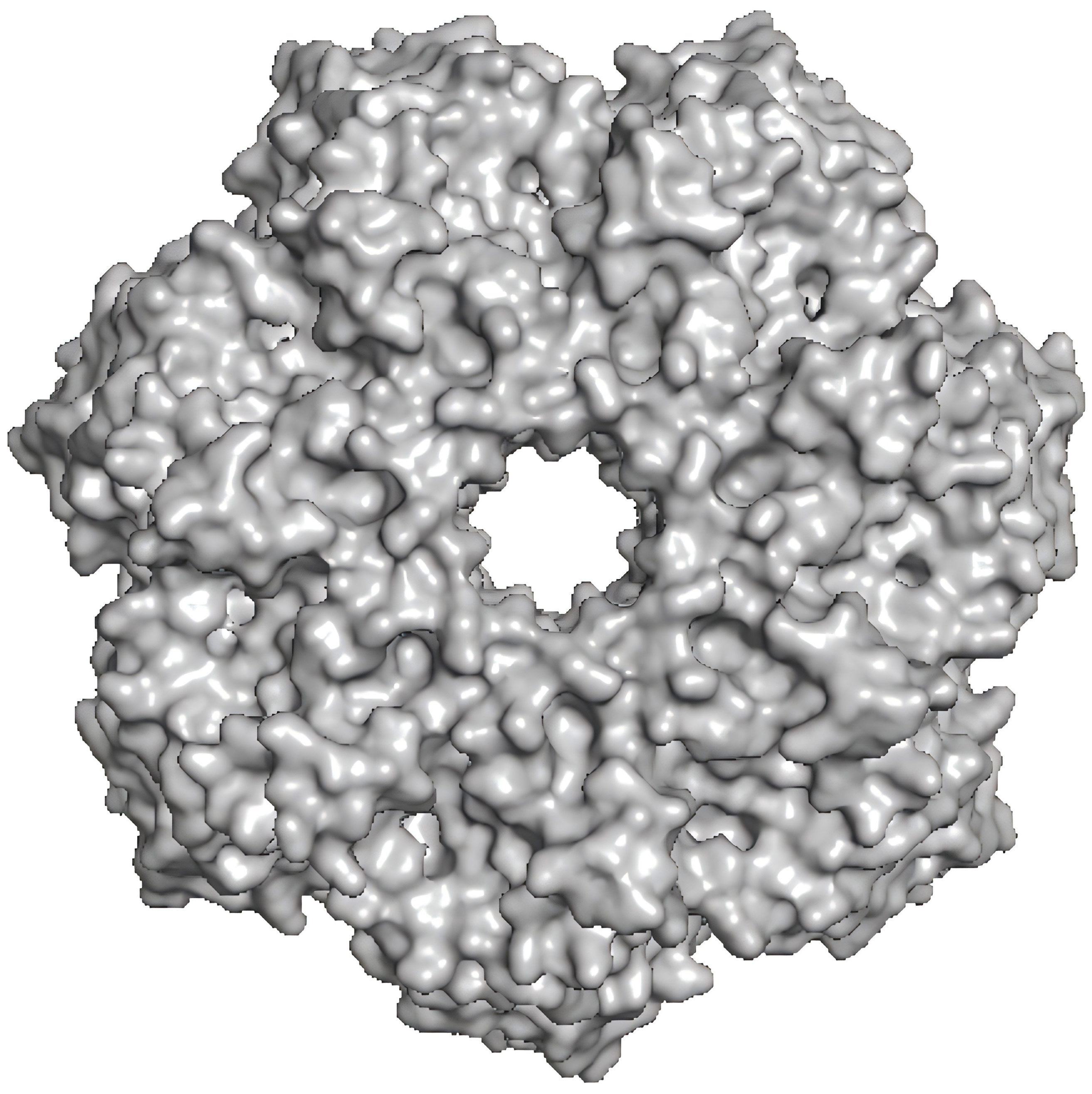

Supplement: S1 File — (ZIP) [file ppat.1013909.s010.zip › S4 Fig/S4A-k Fig.jpg]

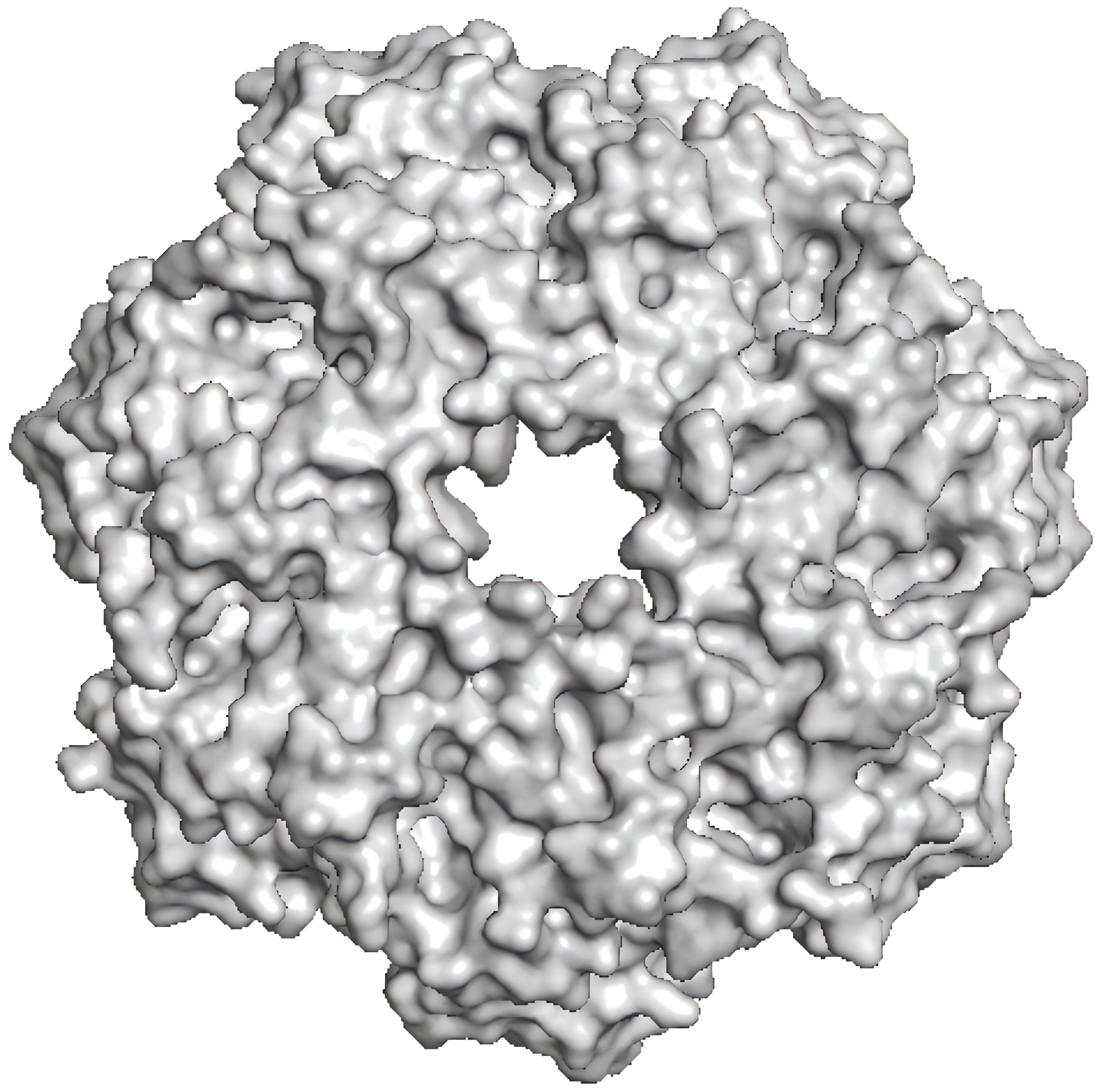

Supplement: S1 File — (ZIP) [file ppat.1013909.s010.zip › S4 Fig/S4A-l Fig.jpg]

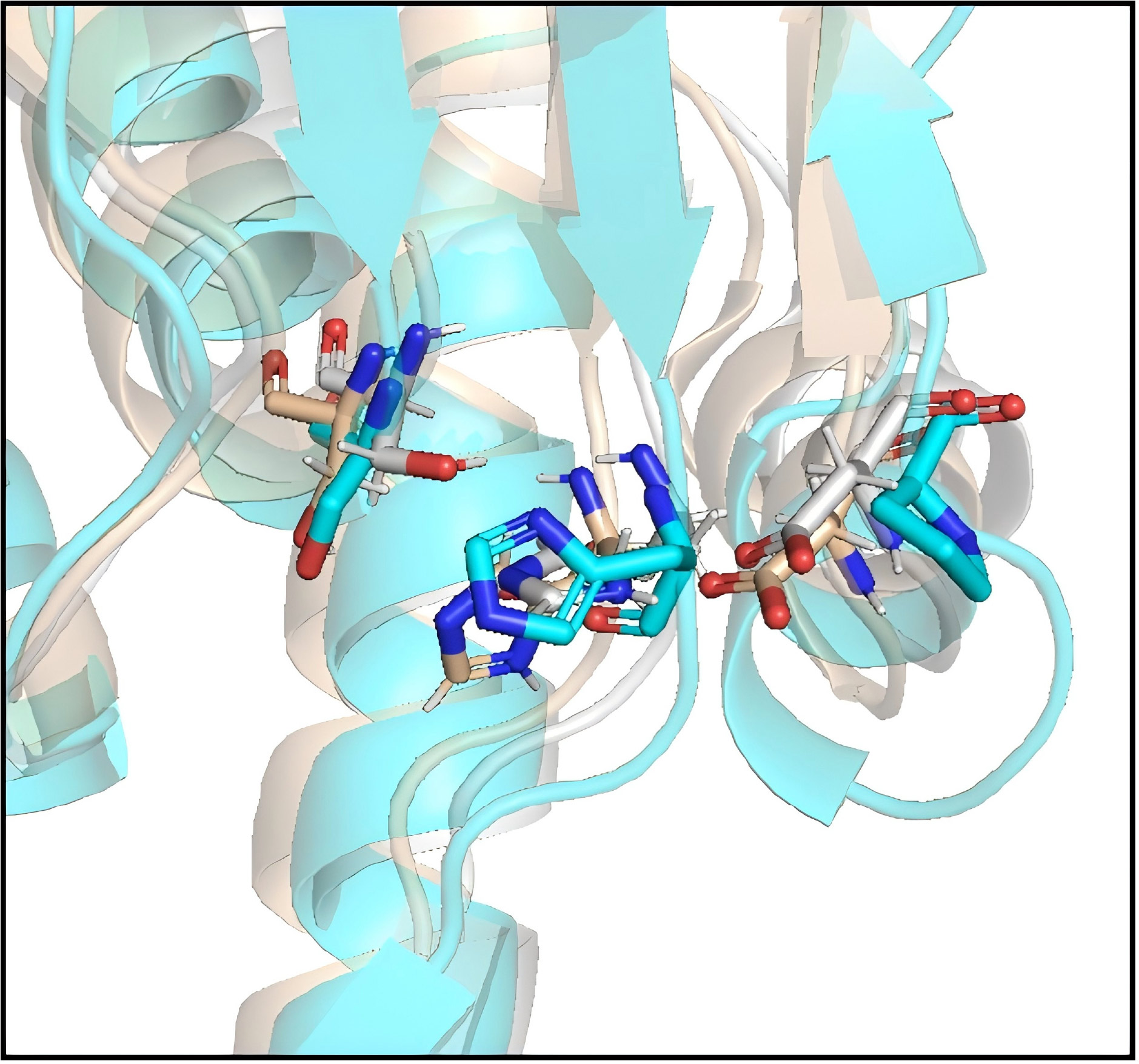

Supplement: S1 File — (ZIP) [file ppat.1013909.s010.zip › S4 Fig/S4B Fig.jpg]

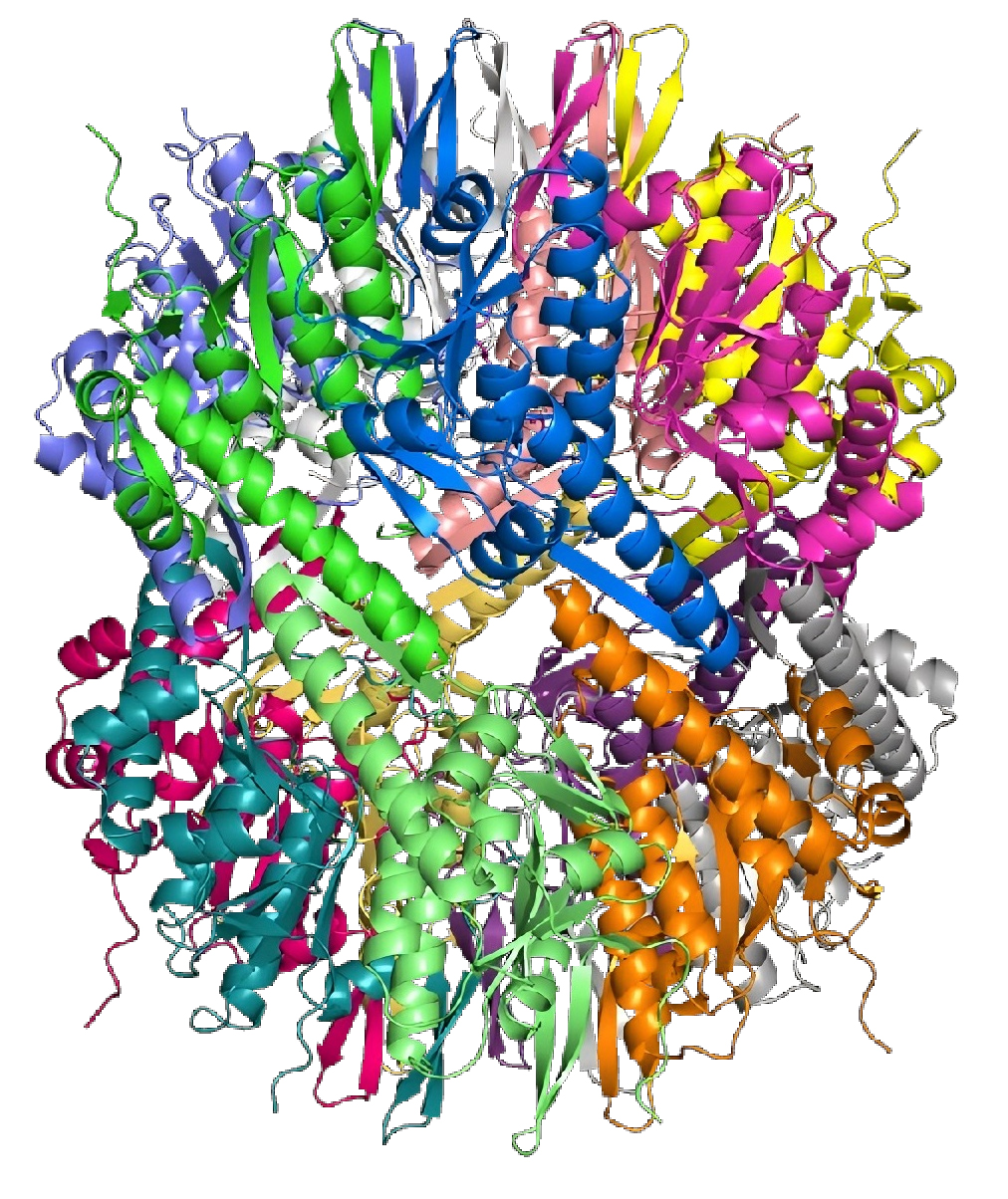

Supplement: S1 File — (ZIP) [file ppat.1013909.s010.zip › S5 Fig/S5A Fig-Side view.jpg]

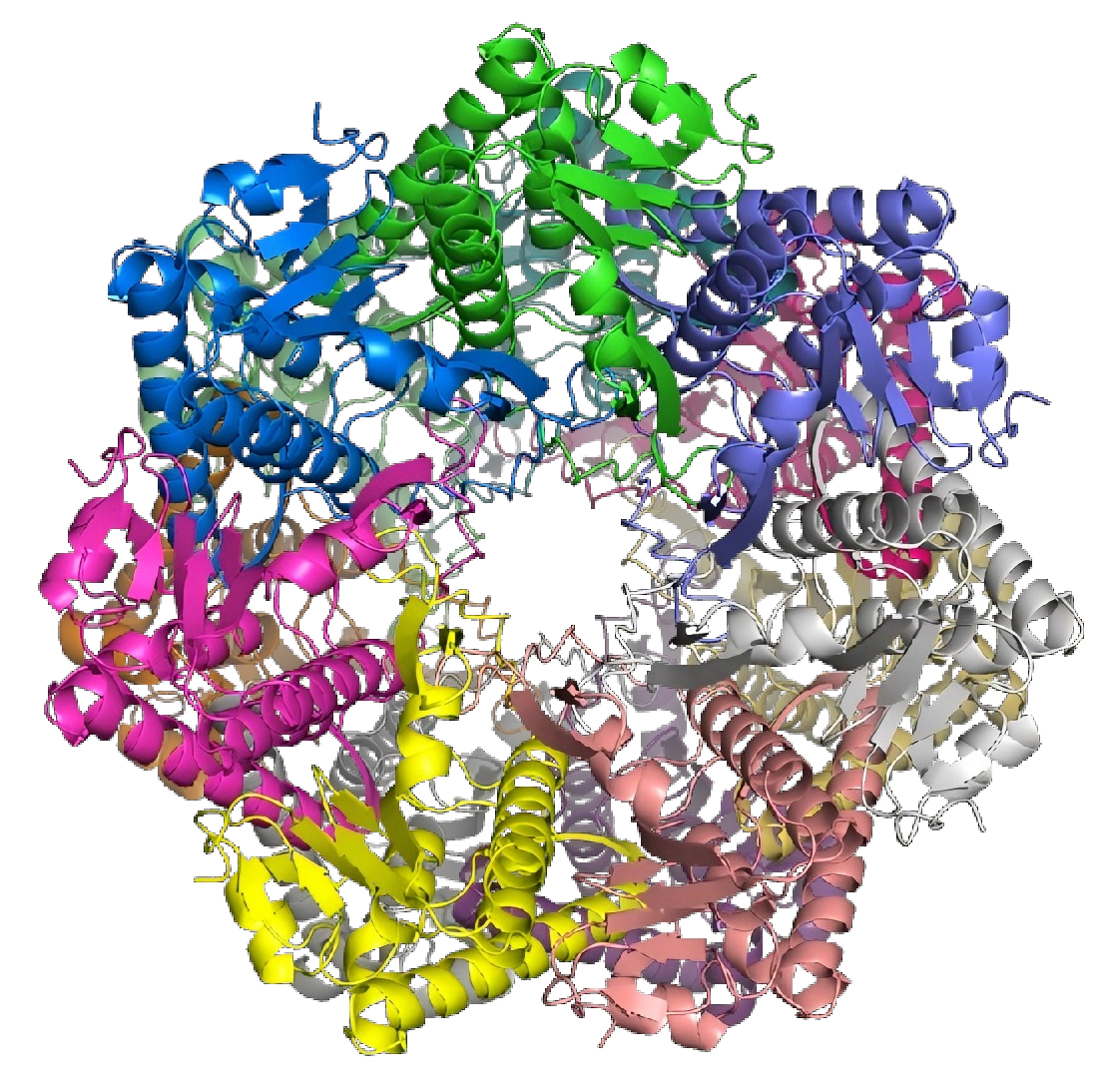

Supplement: S1 File — (ZIP) [file ppat.1013909.s010.zip › S5 Fig/S5A Fig-Top view.jpg]

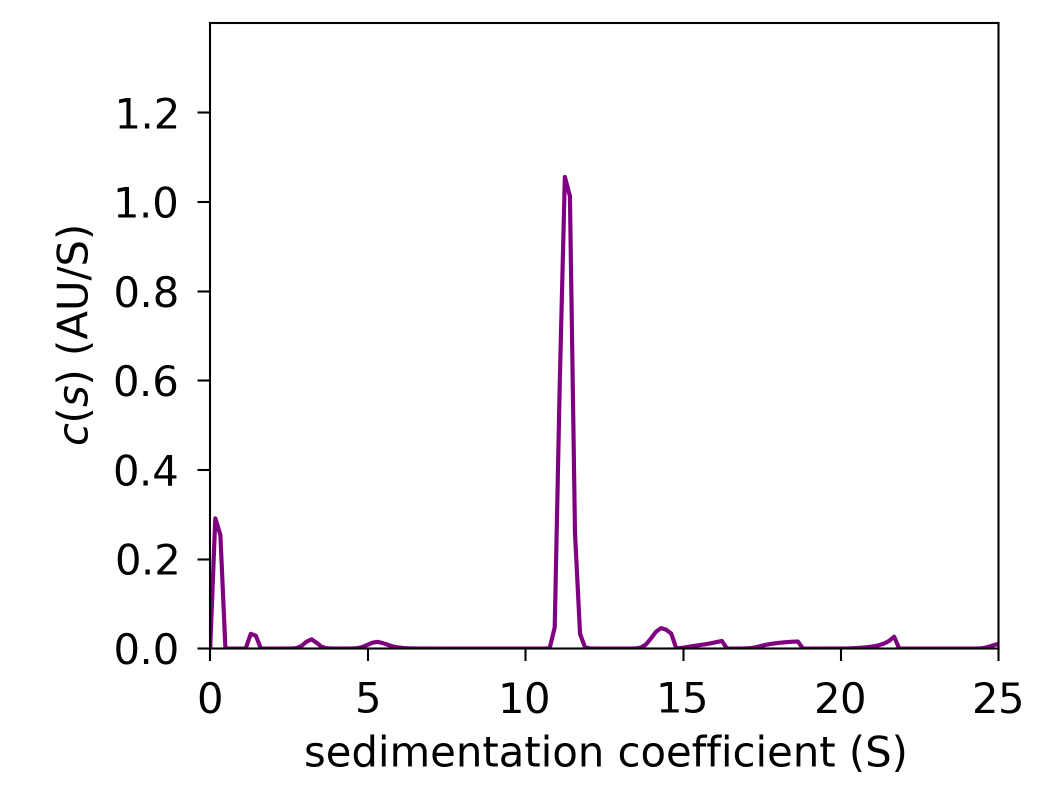

Supplement: S1 File — (ZIP) [file ppat.1013909.s010.zip › S5 Fig/S5B Fig.jpg]

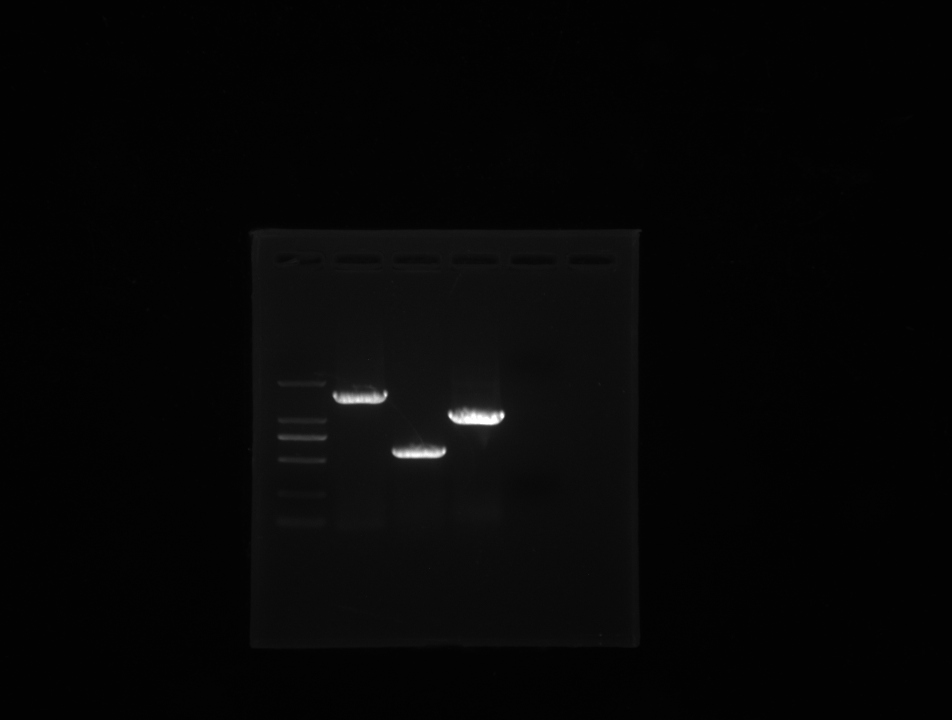

Supplement: S1 File — (ZIP) [file ppat.1013909.s010.zip › S5 Fig/S5C Fig.Tif]

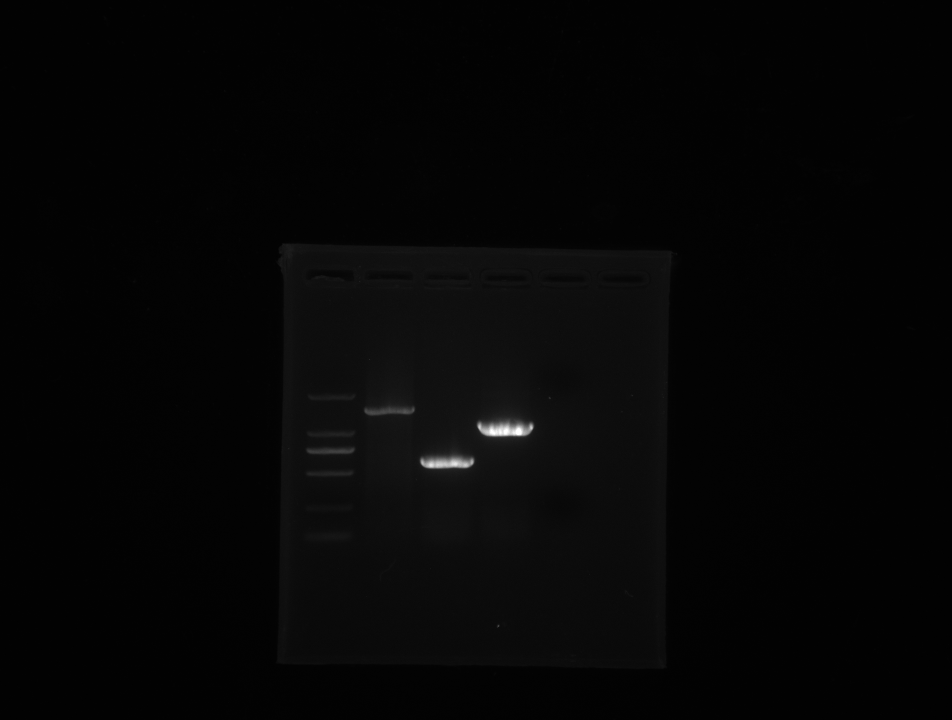

Supplement: S1 File — (ZIP) [file ppat.1013909.s010.zip › S5 Fig/S5D Fig.Tif]

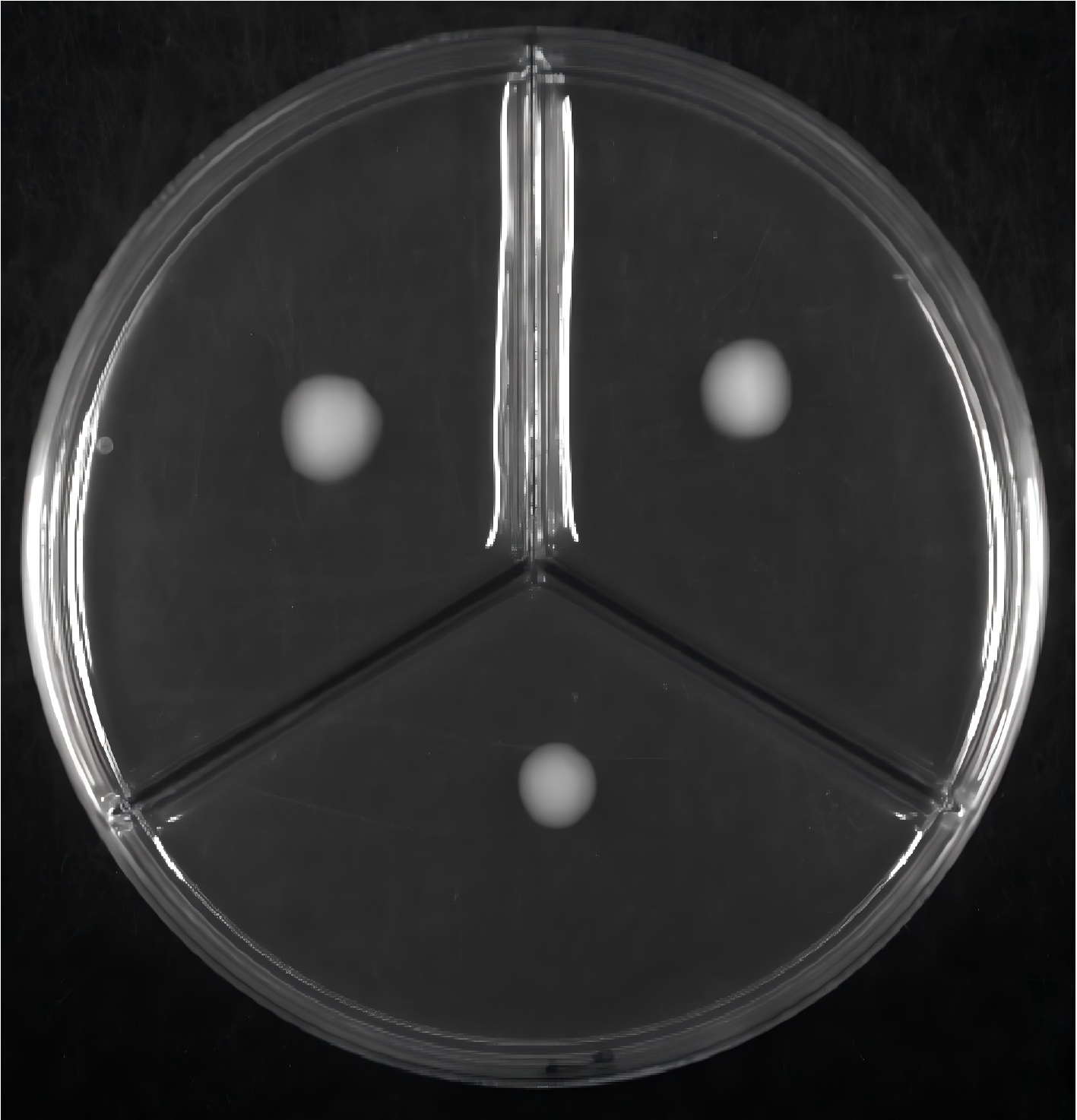

Supplement: S1 File — (ZIP) [file ppat.1013909.s010.zip › S5 Fig/S5E Fig-PpClpP1-swarming.jpg]

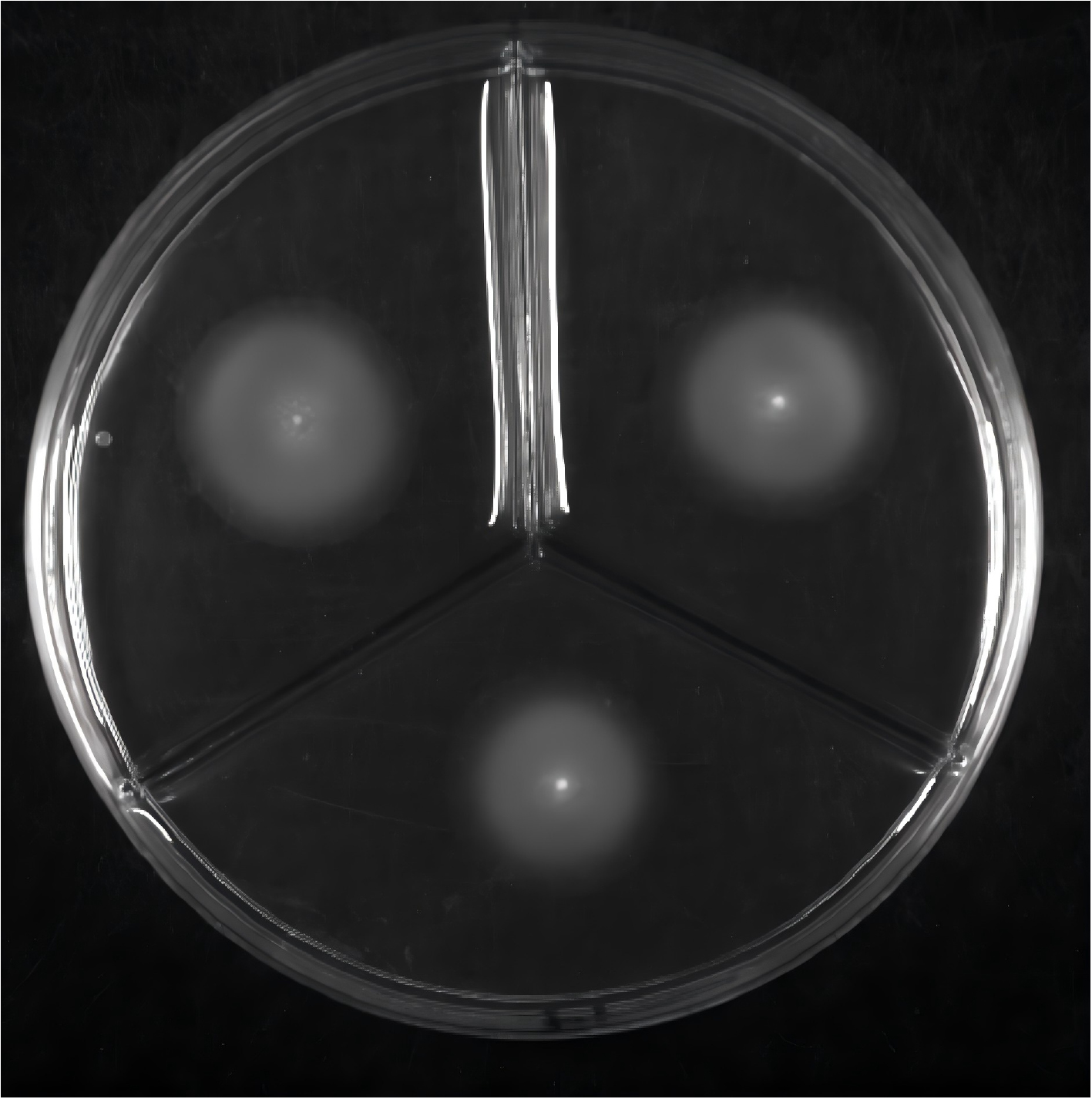

Supplement: S1 File — (ZIP) [file ppat.1013909.s010.zip › S5 Fig/S5E Fig-PpClpP1-swimming.jpg]

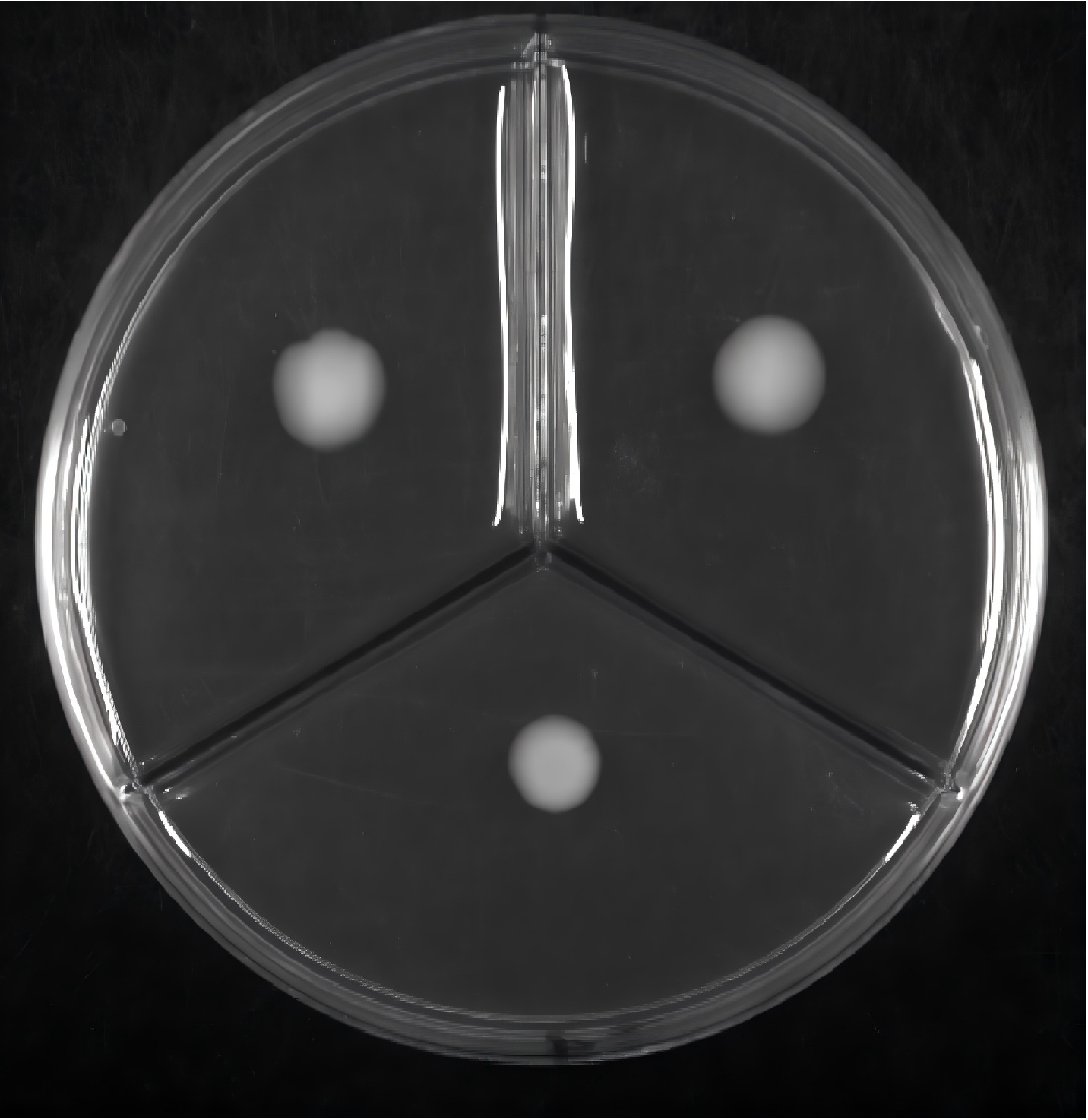

Supplement: S1 File — (ZIP) [file ppat.1013909.s010.zip › S5 Fig/S5F Fig-PpClpP2-swarming.jpg]

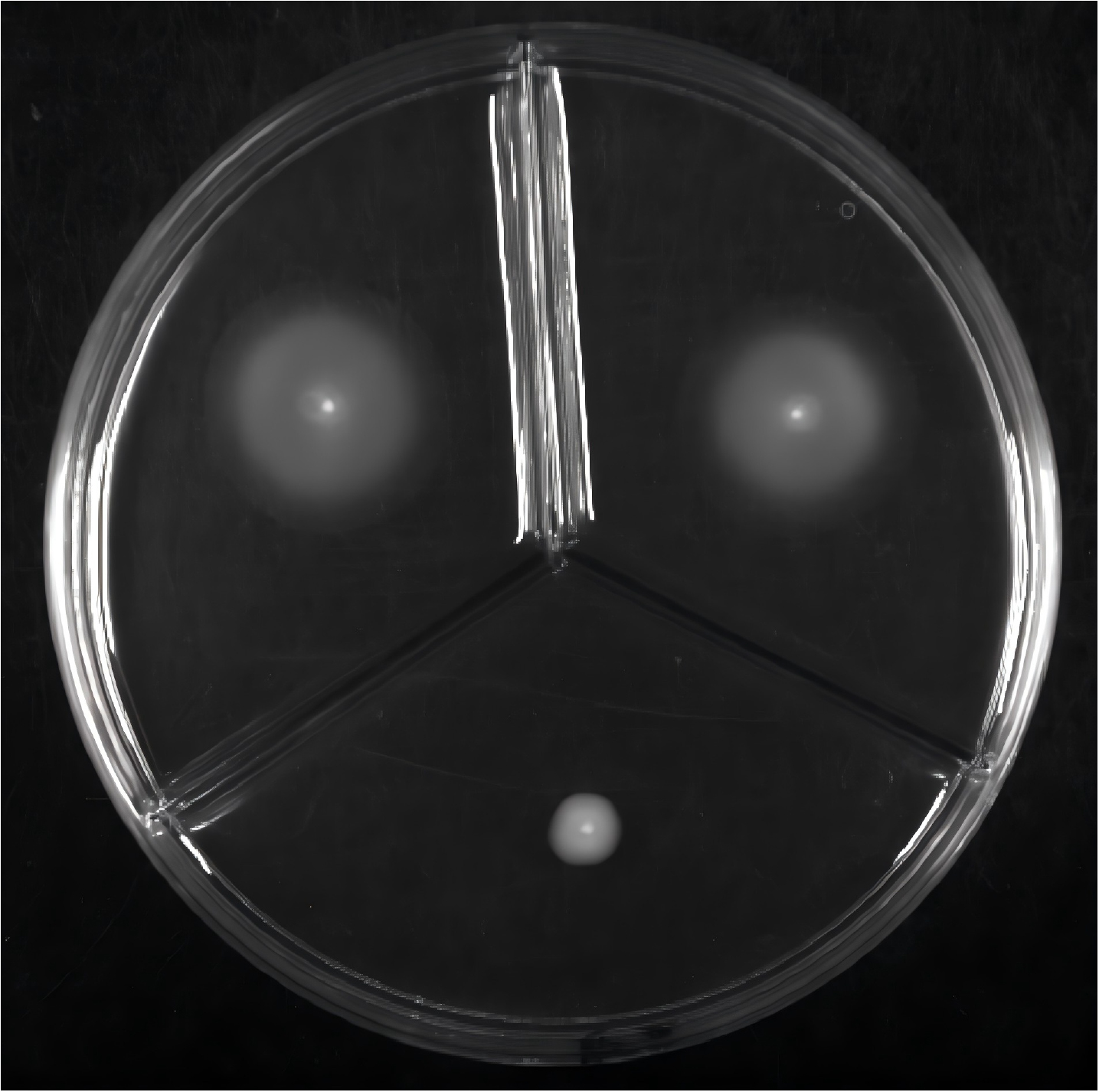

Supplement: S1 File — (ZIP) [file ppat.1013909.s010.zip › S5 Fig/S5F Fig-PpClpP2-swimming.jpg]

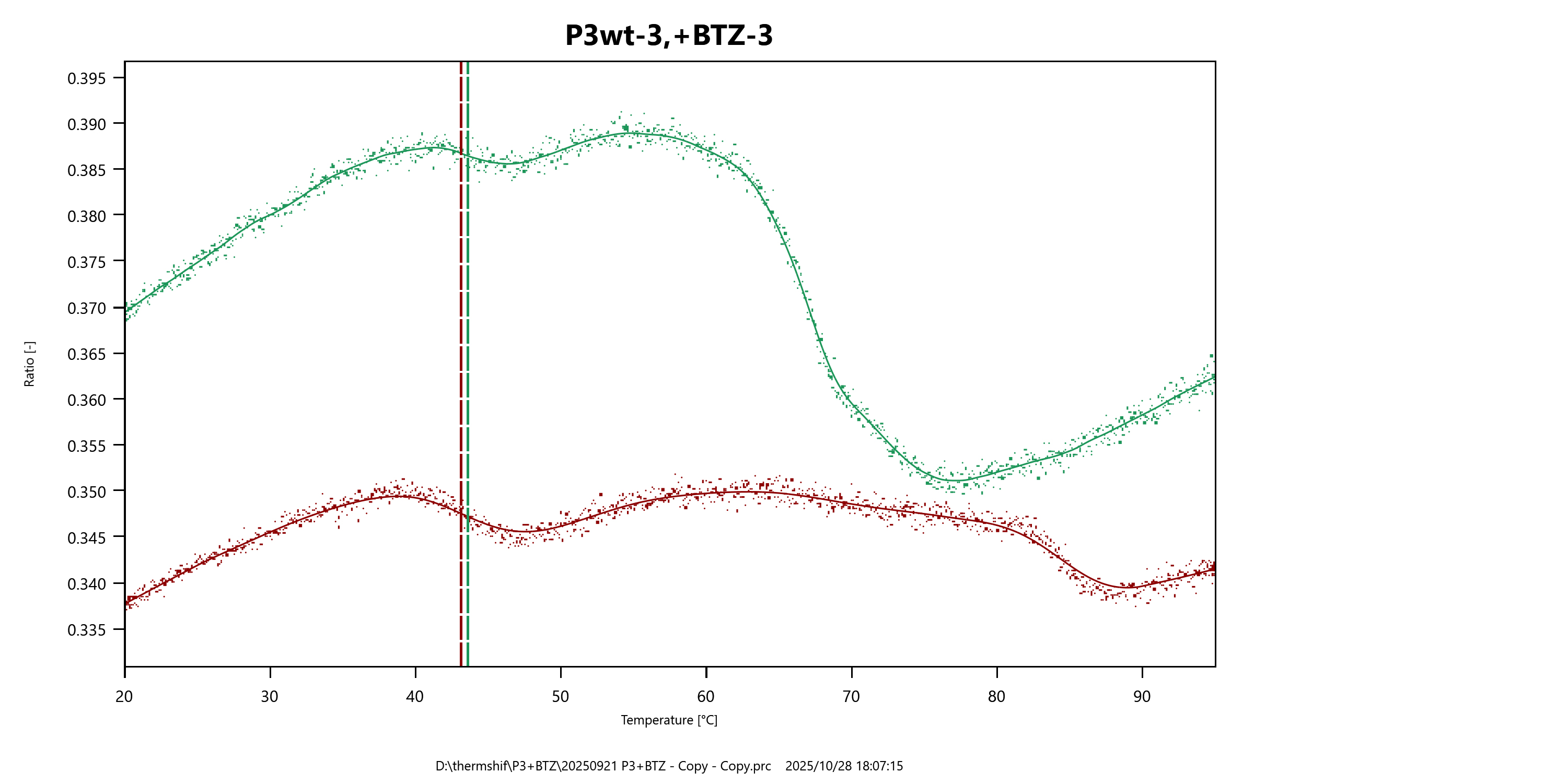

Supplement: S1 File — (ZIP) [file ppat.1013909.s010.zip › S5 Fig/S5G Fig.jpg]

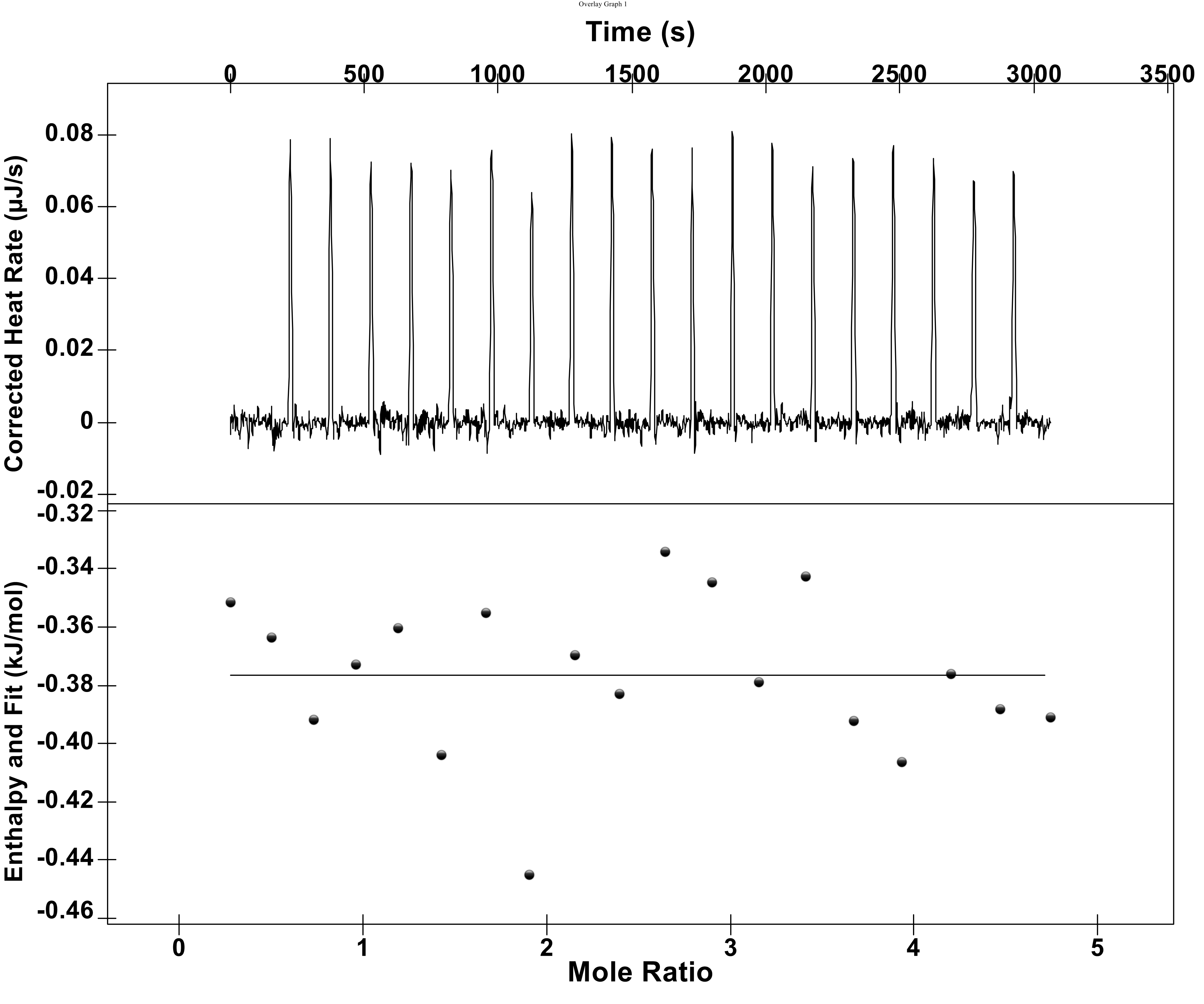

Supplement: S1 File — (ZIP) [file ppat.1013909.s010.zip › S5 Fig/S5H Fig.jpg]

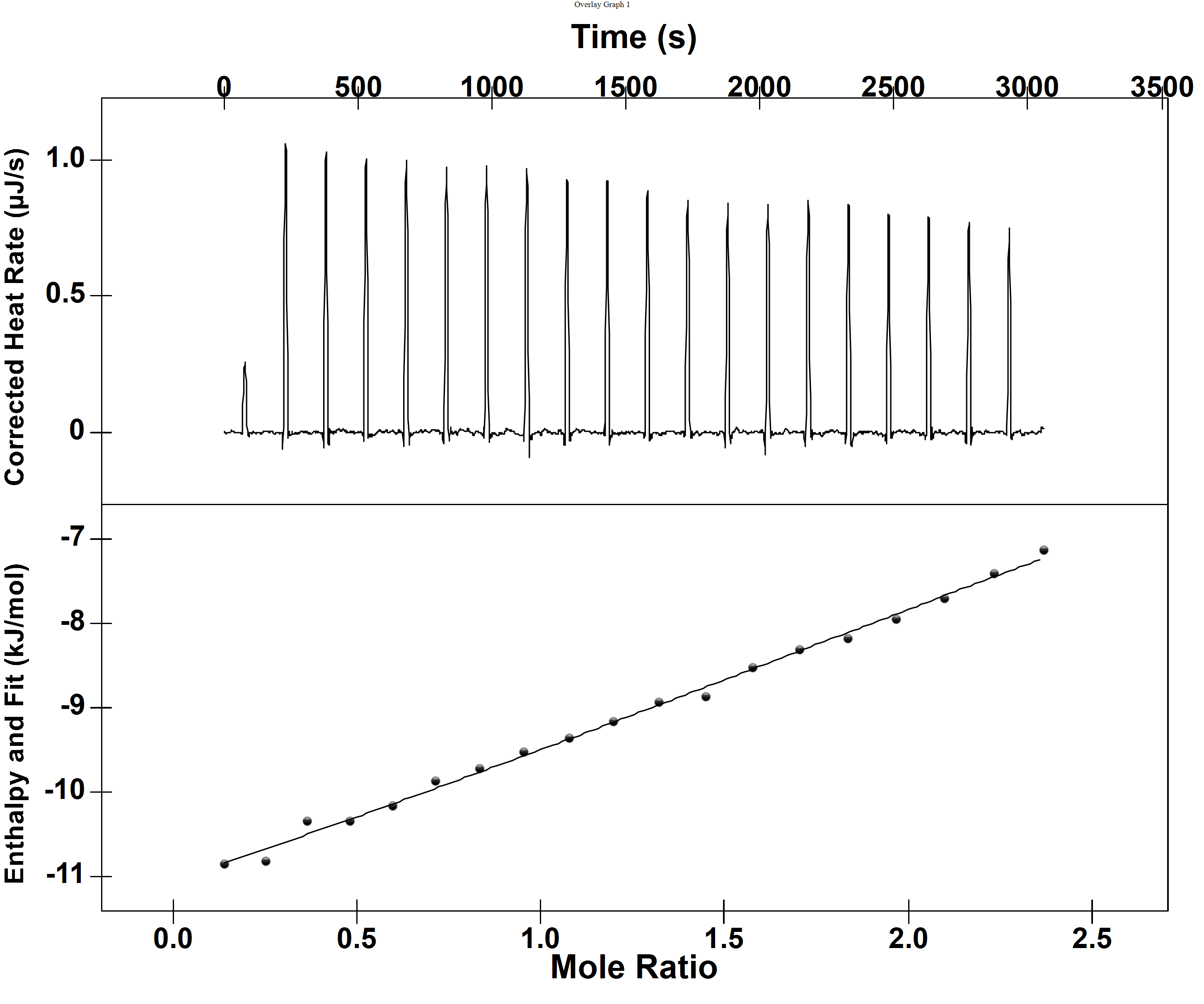

Supplement: S1 File — (ZIP) [file ppat.1013909.s010.zip › S6 Fig/S6A Fig.tif]

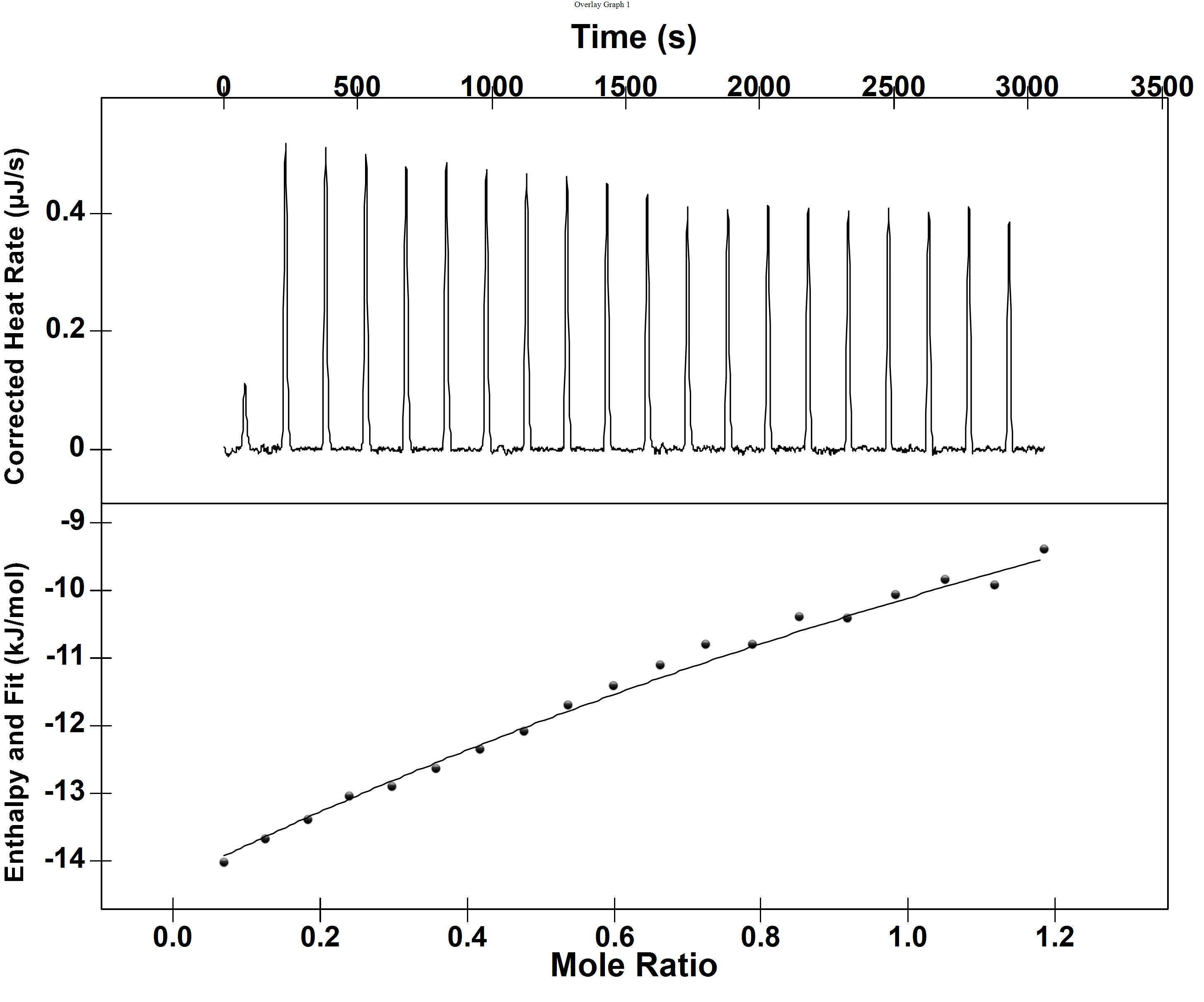

Supplement: S1 File — (ZIP) [file ppat.1013909.s010.zip › S6 Fig/S6B Fig.tif]

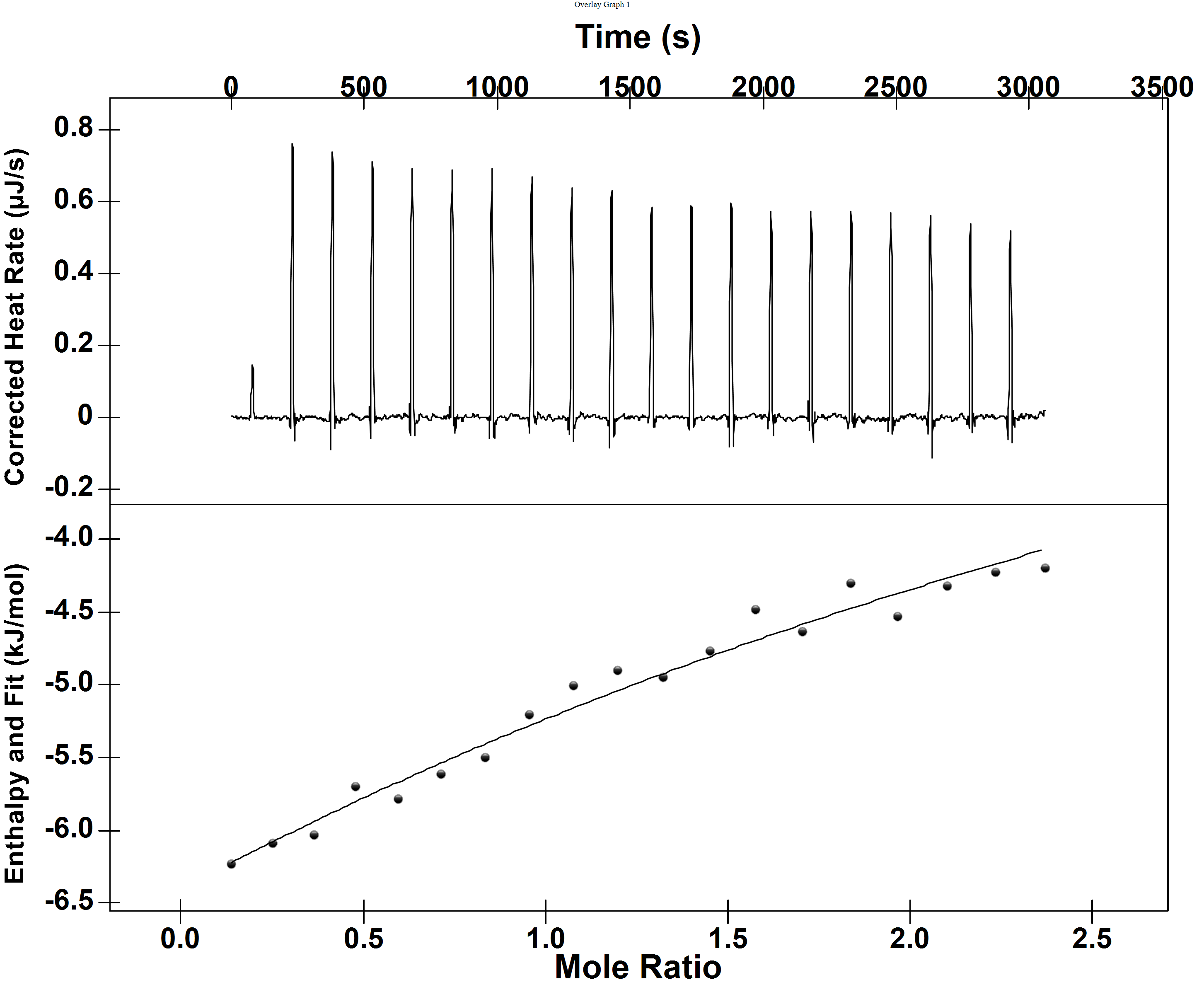

Supplement: S1 File — (ZIP) [file ppat.1013909.s010.zip › S6 Fig/S6C Fig.tif]

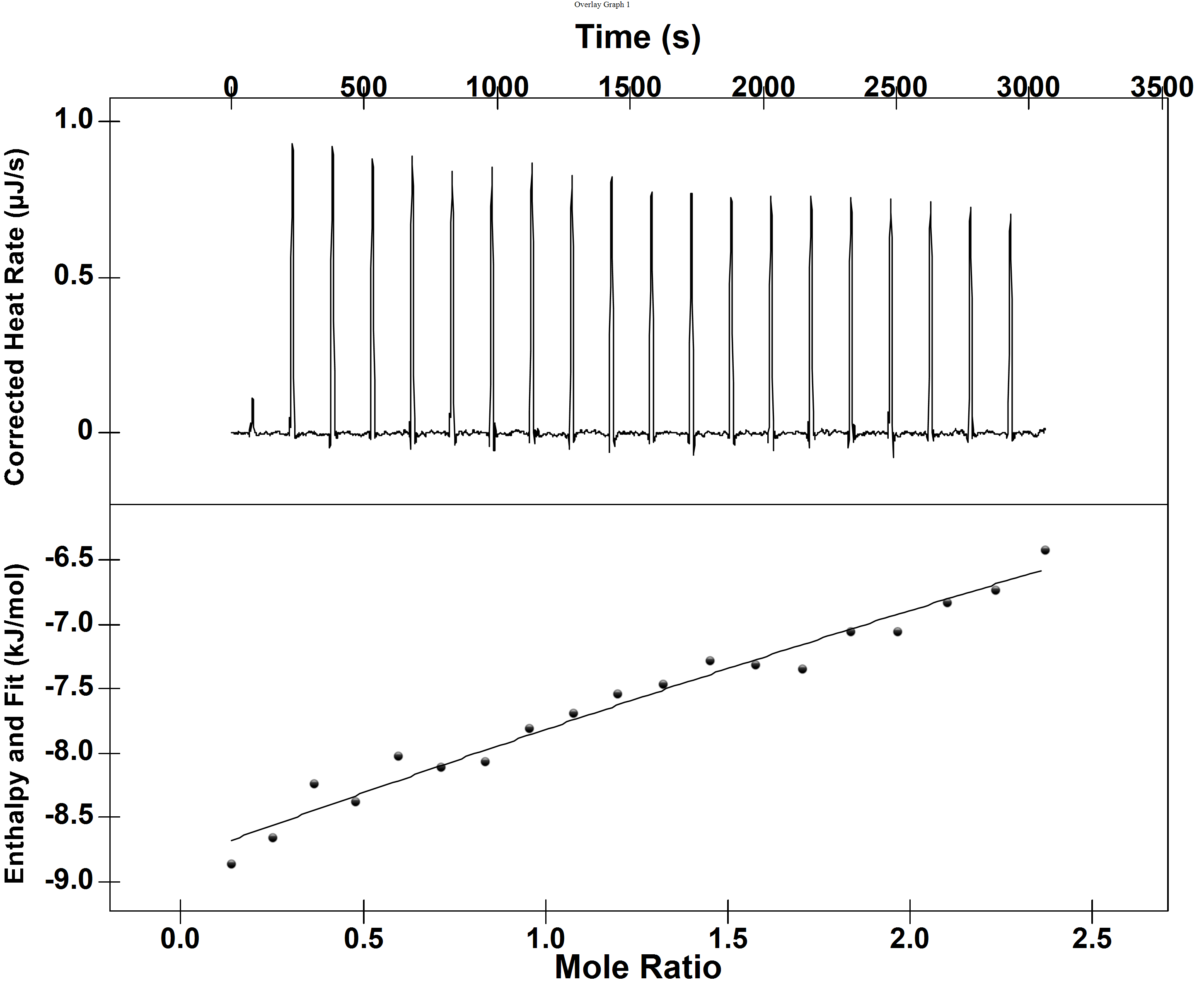

Supplement: S1 File — (ZIP) [file ppat.1013909.s010.zip › S6 Fig/S6D Fig.tif]

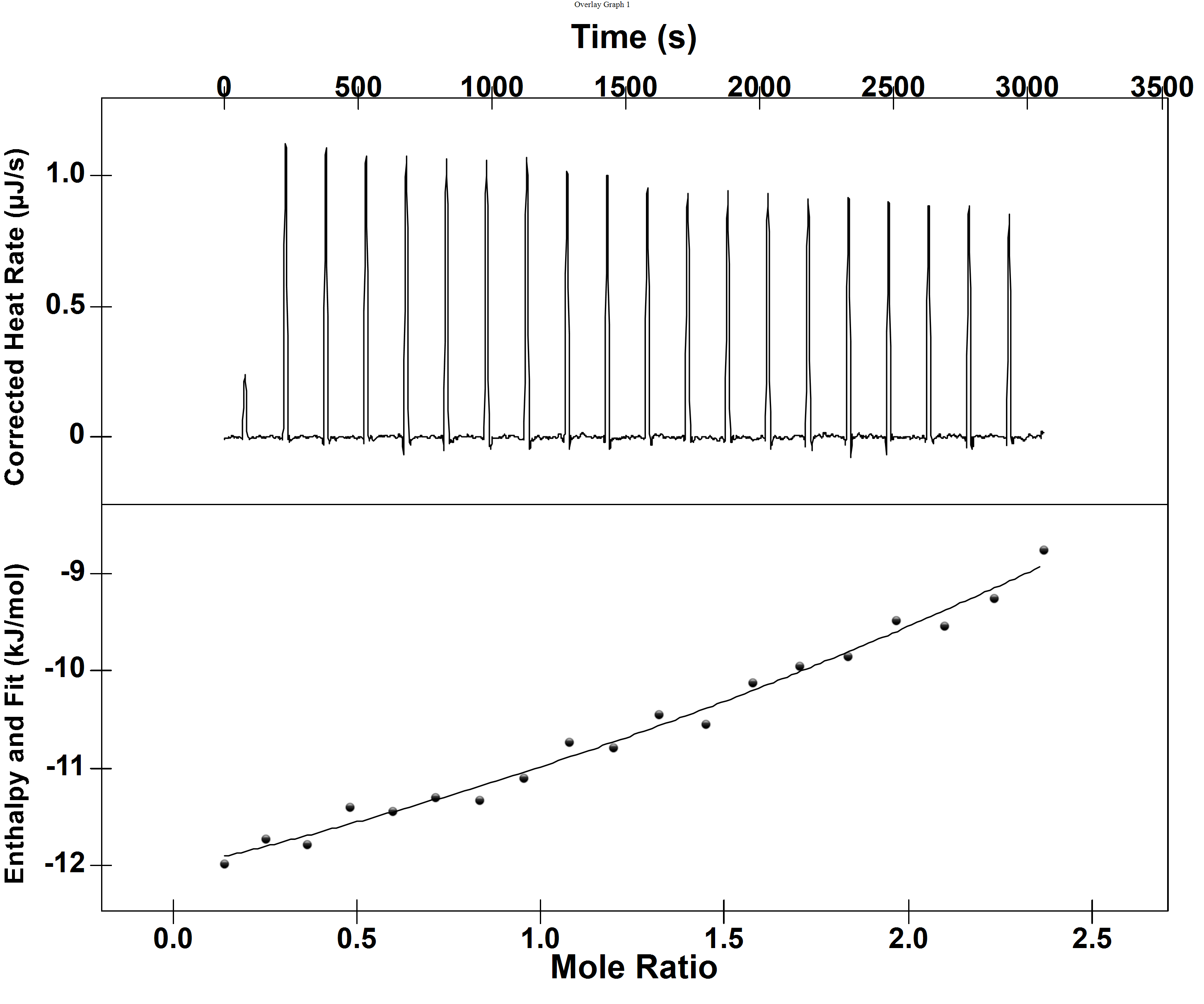

Supplement: S1 File — (ZIP) [file ppat.1013909.s010.zip › S6 Fig/S6E Fig.tif]

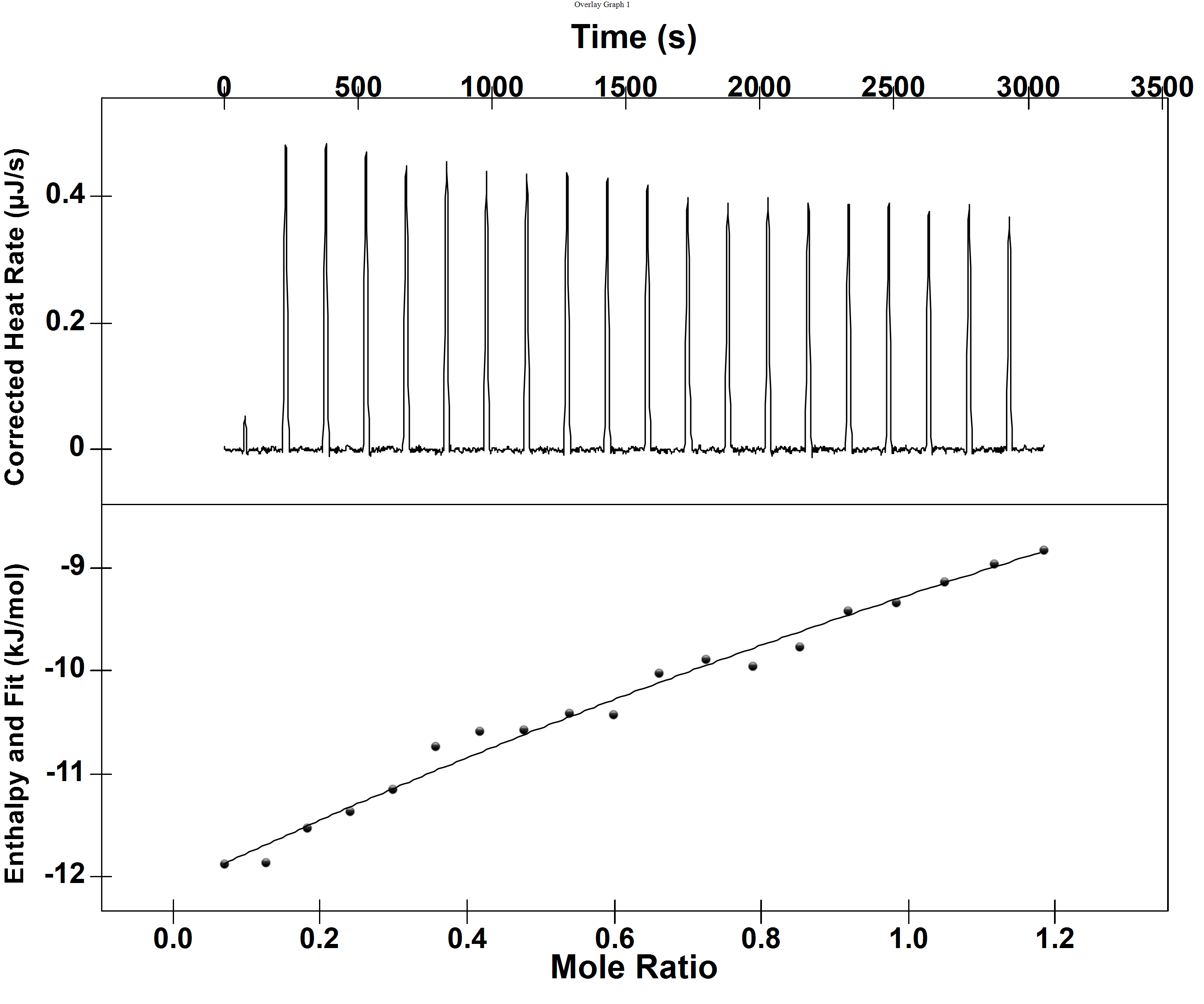

Supplement: S1 File — (ZIP) [file ppat.1013909.s010.zip › S6 Fig/S6F Fig.tif]

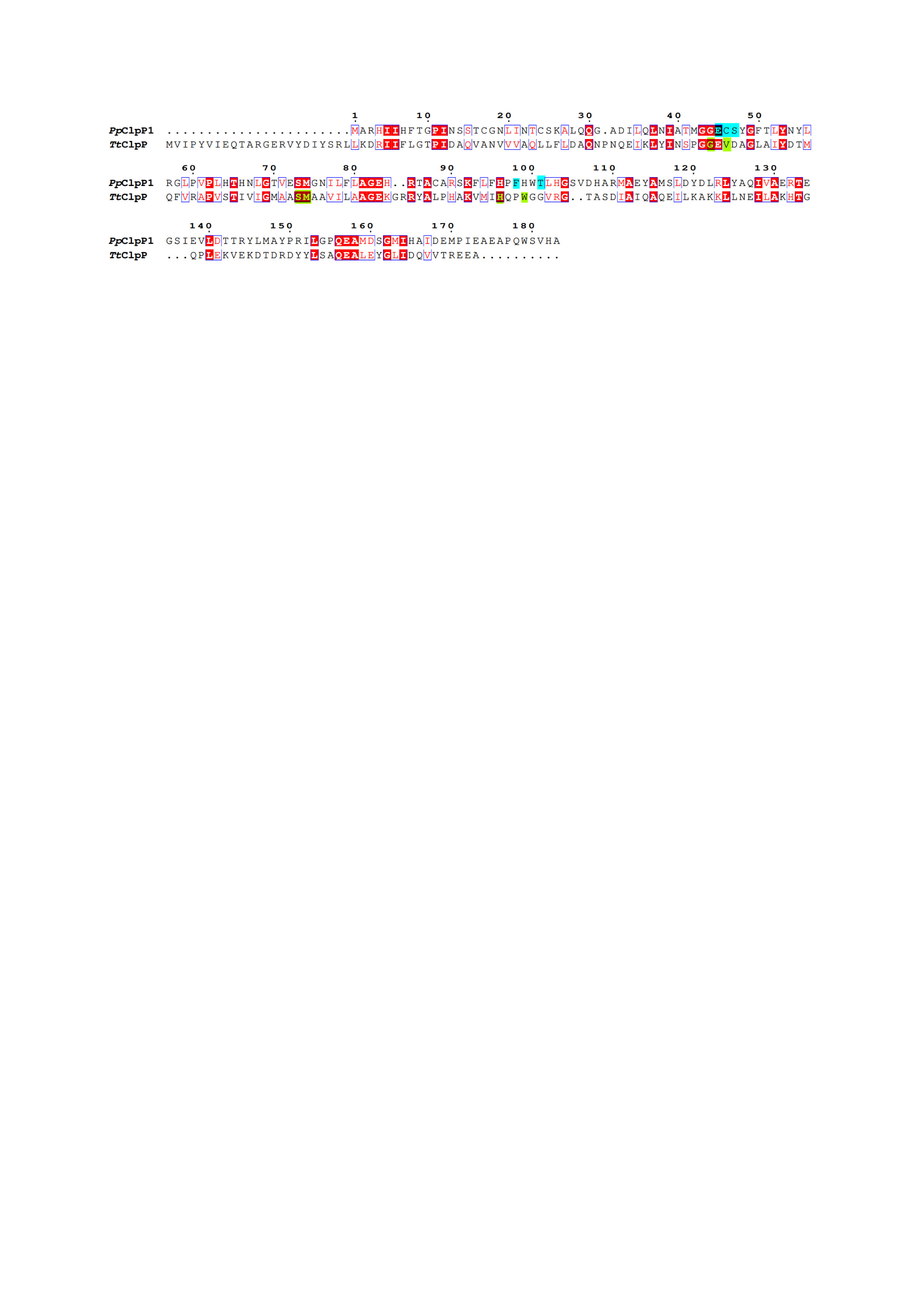

Supplement: S1 File — (ZIP) [file ppat.1013909.s010.zip › S7 Fig/S7A Fig.jpg]

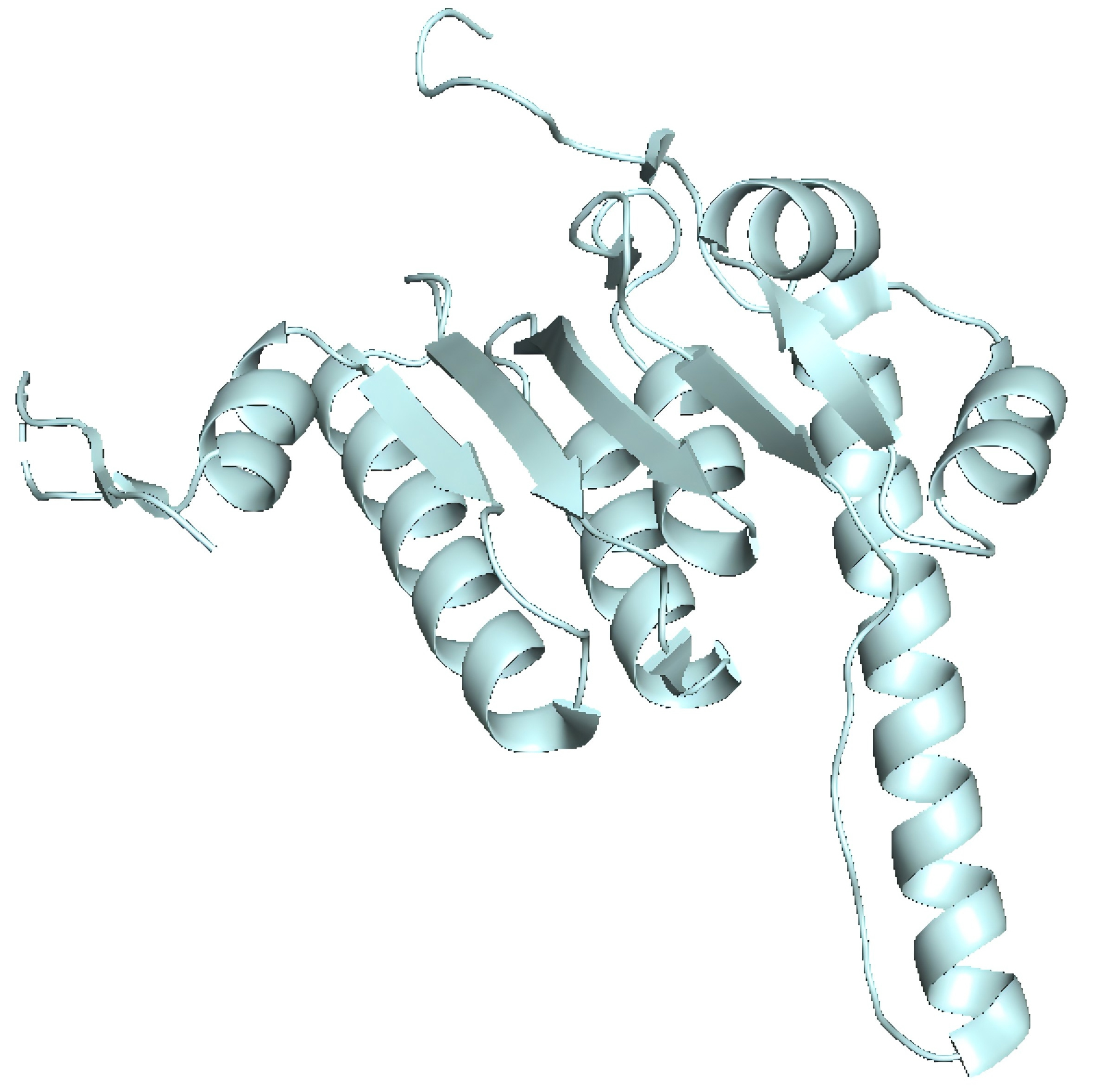

Supplement: S1 File — (ZIP) [file ppat.1013909.s010.zip › S7 Fig/S7B-Left Fig.jpg]

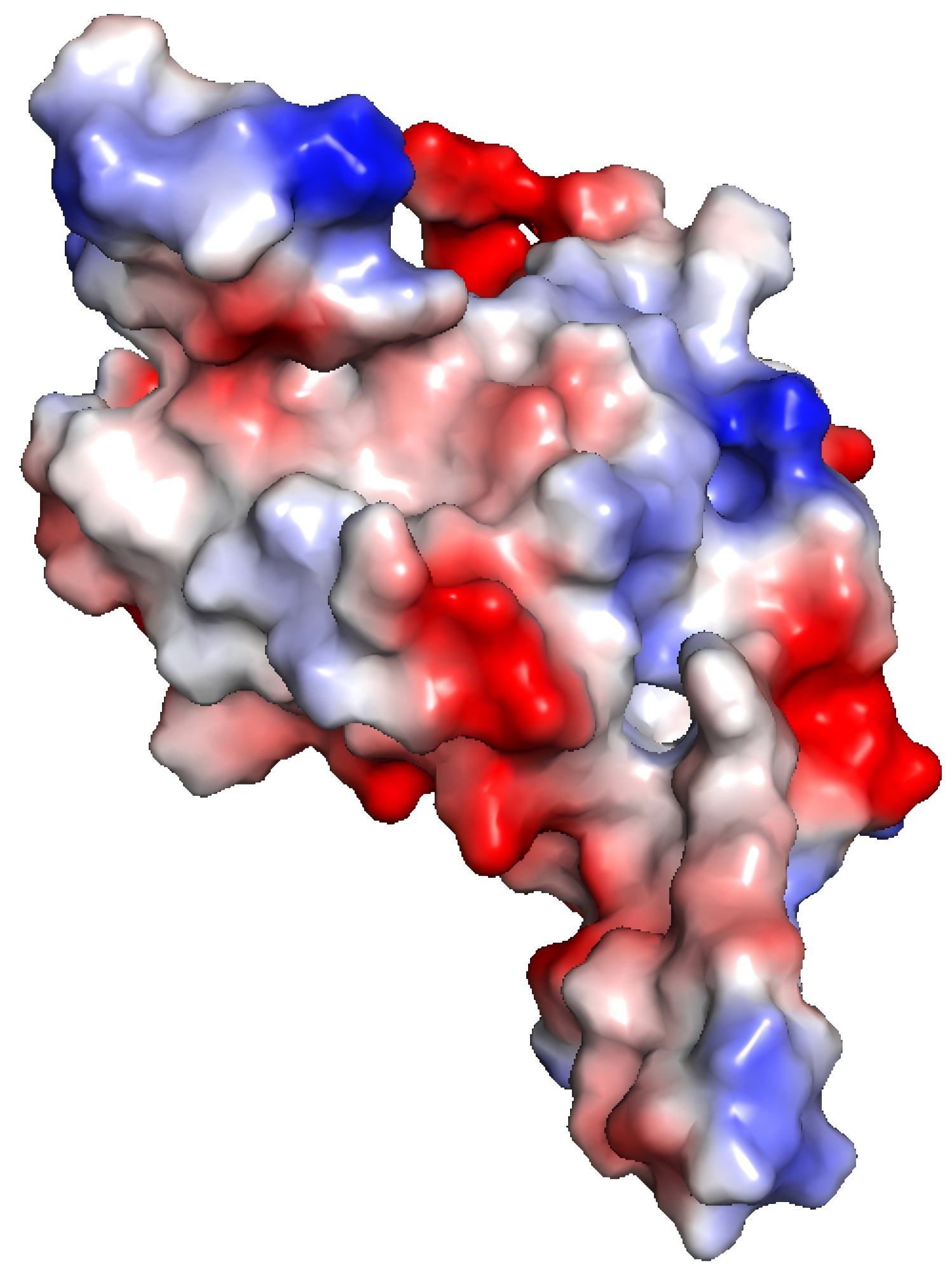

Supplement: S1 File — (ZIP) [file ppat.1013909.s010.zip › S7 Fig/S7B-Right Fig.jpg]

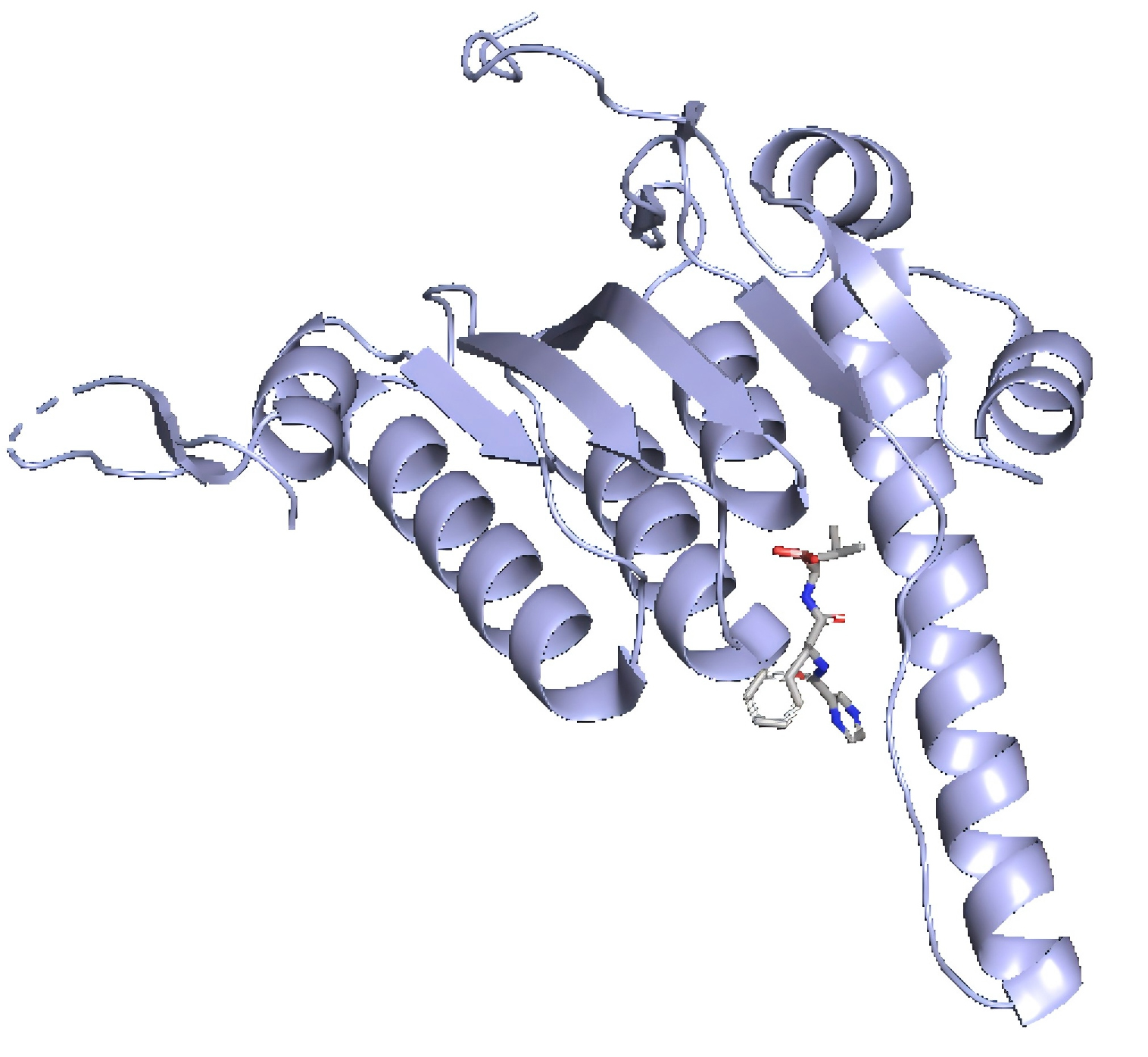

Supplement: S1 File — (ZIP) [file ppat.1013909.s010.zip › S7 Fig/S7C-Left Fig.jpg]

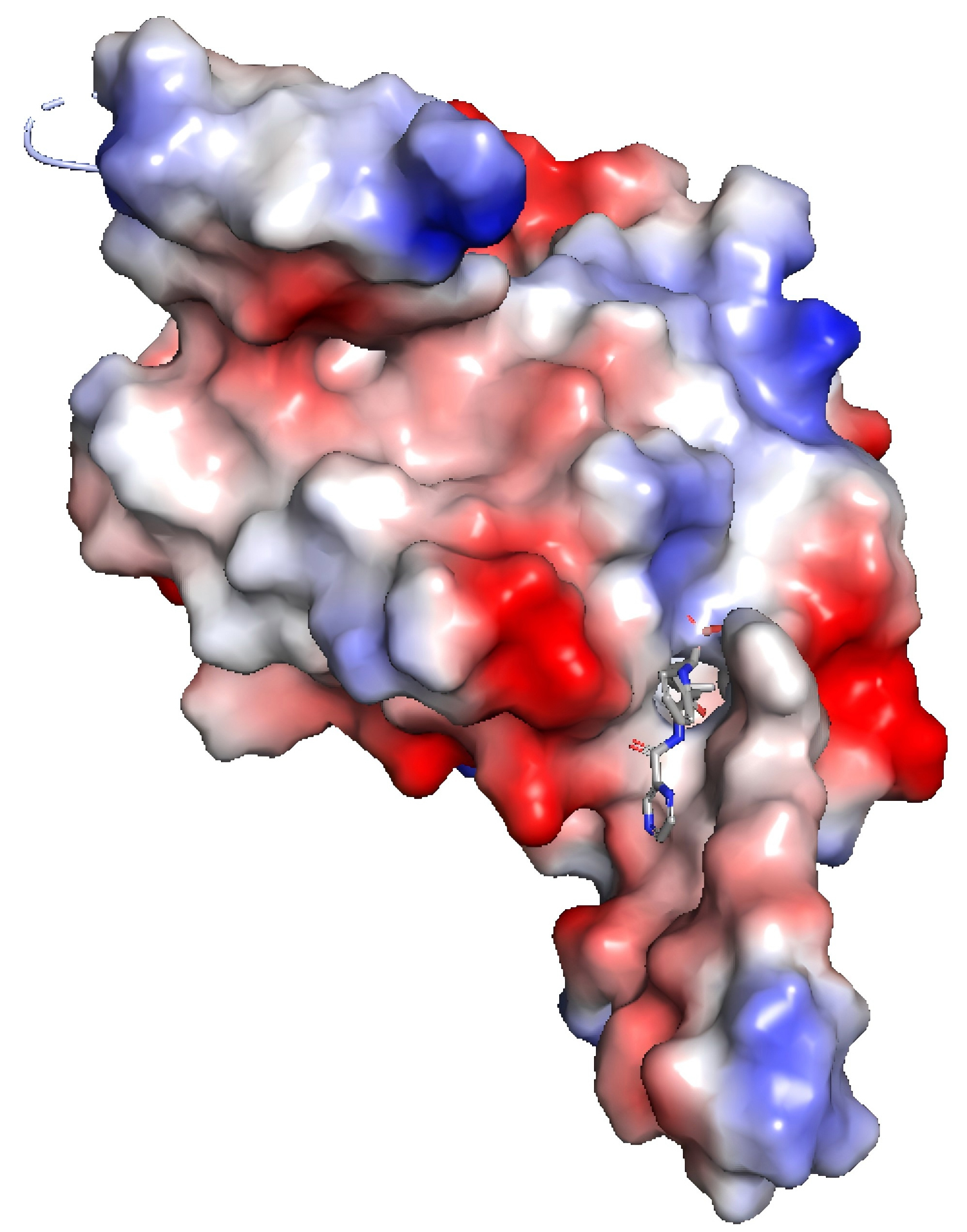

Supplement: S1 File — (ZIP) [file ppat.1013909.s010.zip › S7 Fig/S7C-Right Fig.jpg]

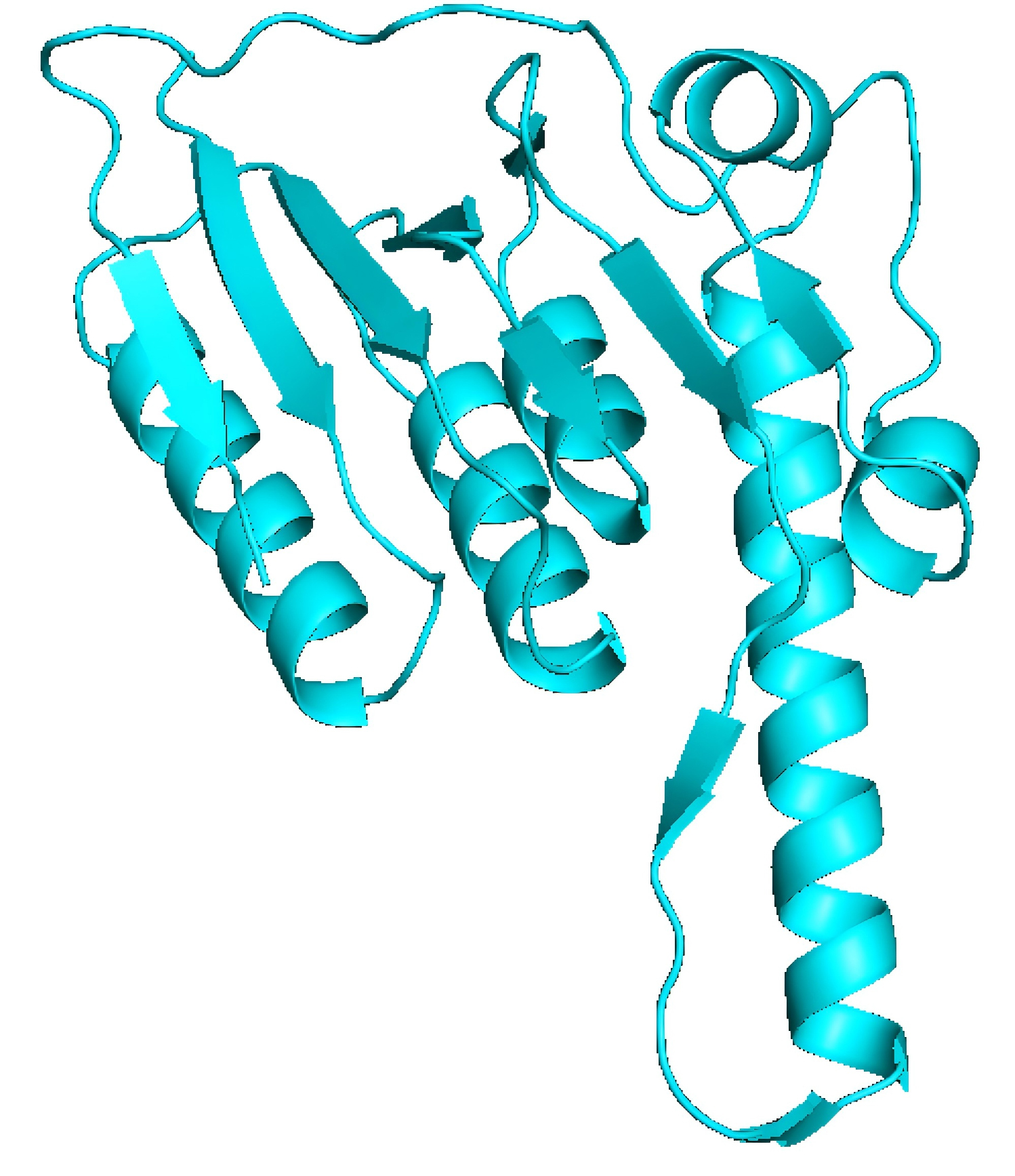

Supplement: S1 File — (ZIP) [file ppat.1013909.s010.zip › S7 Fig/S7D-Left Fig.jpg]

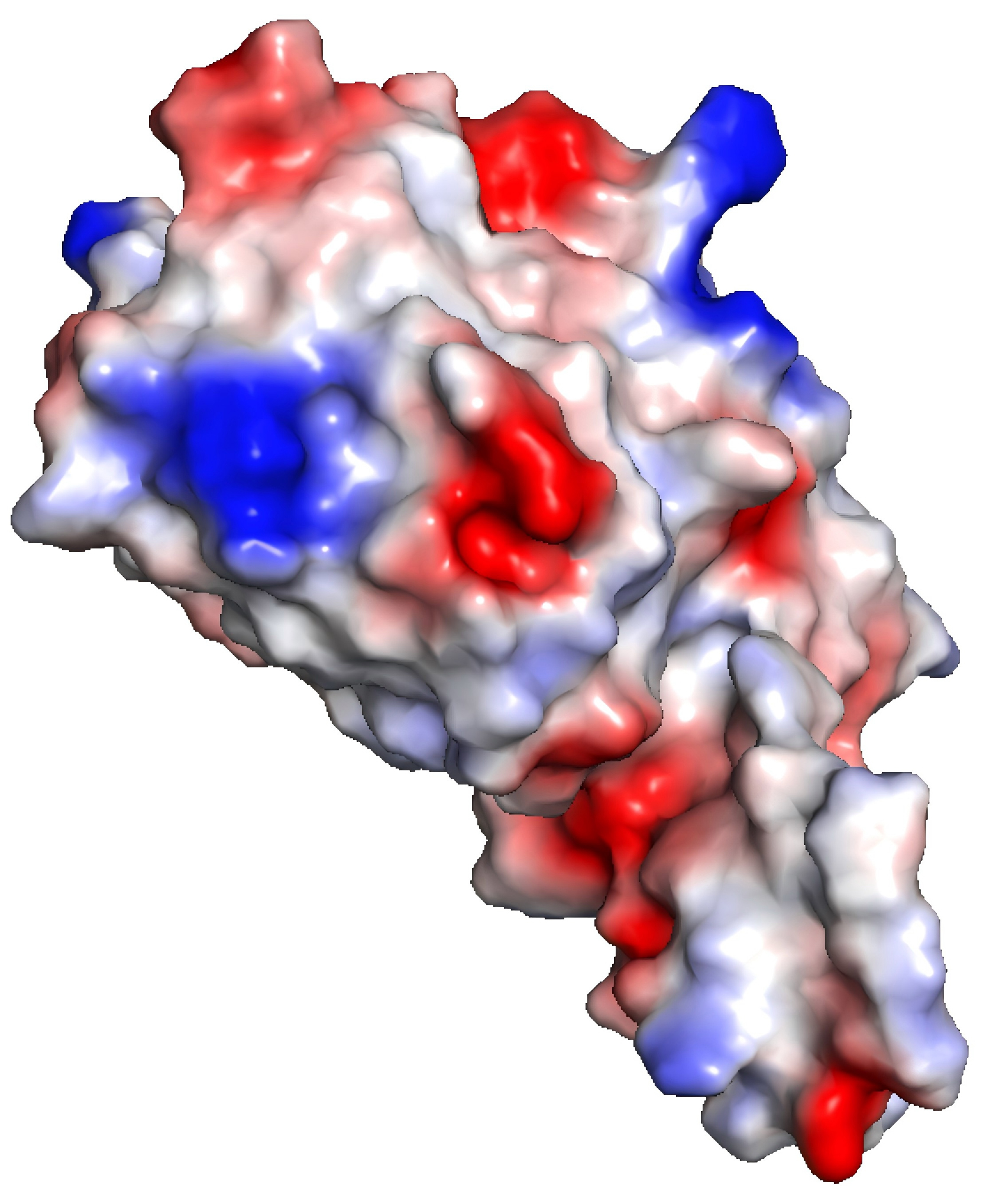

Supplement: S1 File — (ZIP) [file ppat.1013909.s010.zip › S7 Fig/S7D-Right Fig.jpg]

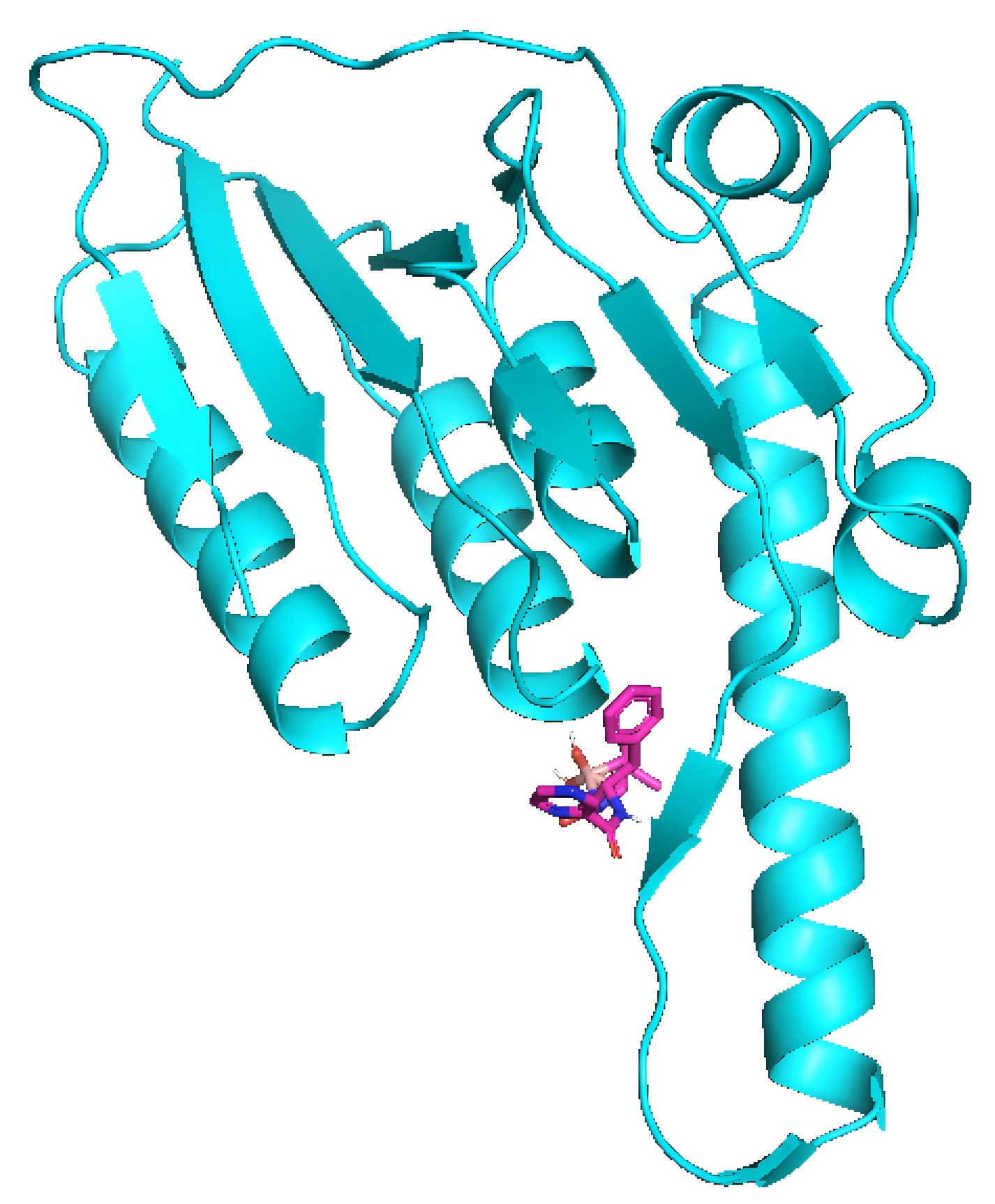

Supplement: S1 File — (ZIP) [file ppat.1013909.s010.zip › S7 Fig/S7E-Left Fig.jpg]

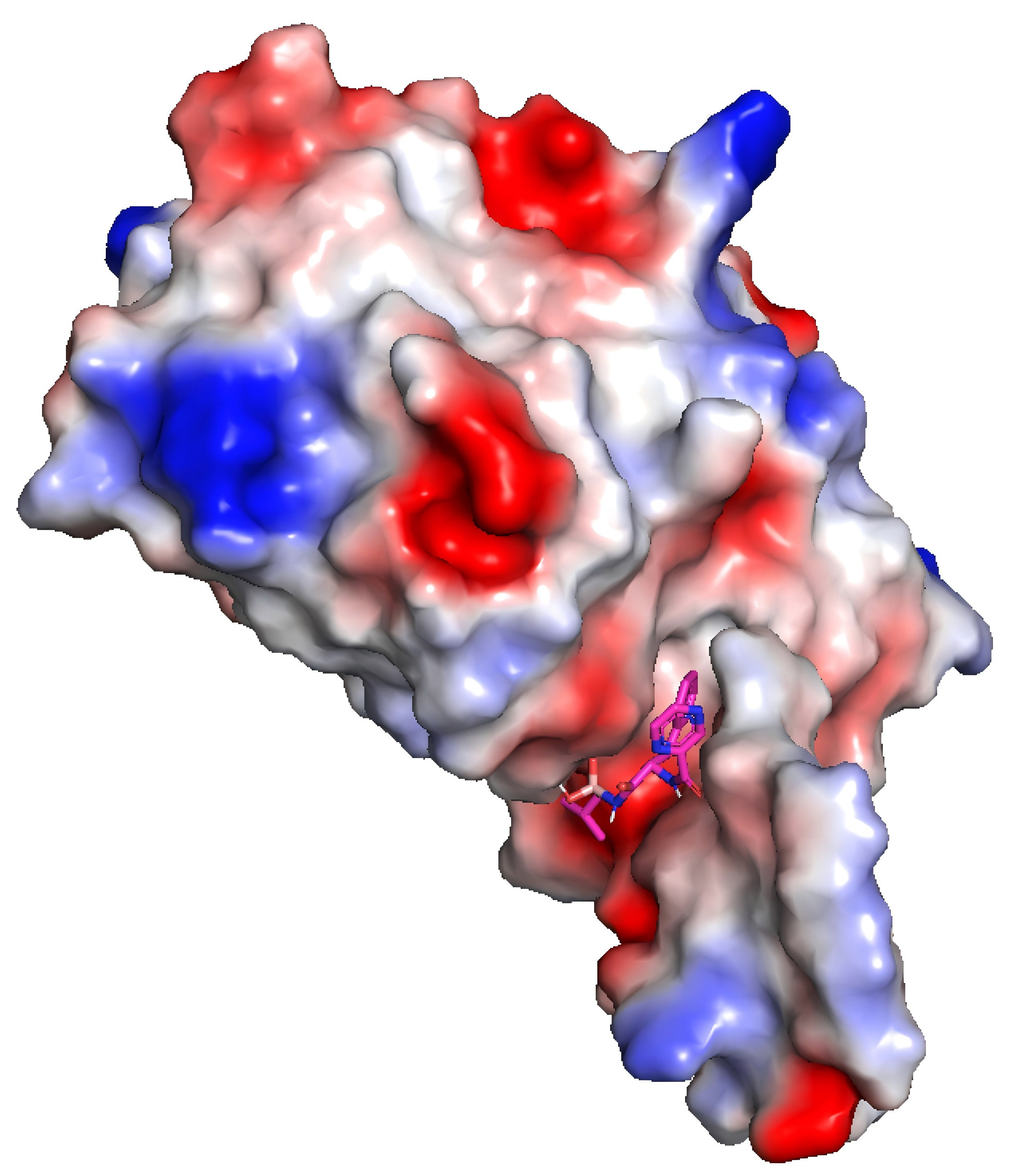

Supplement: S1 File — (ZIP) [file ppat.1013909.s010.zip › S7 Fig/S7E-Right Fig.jpg]

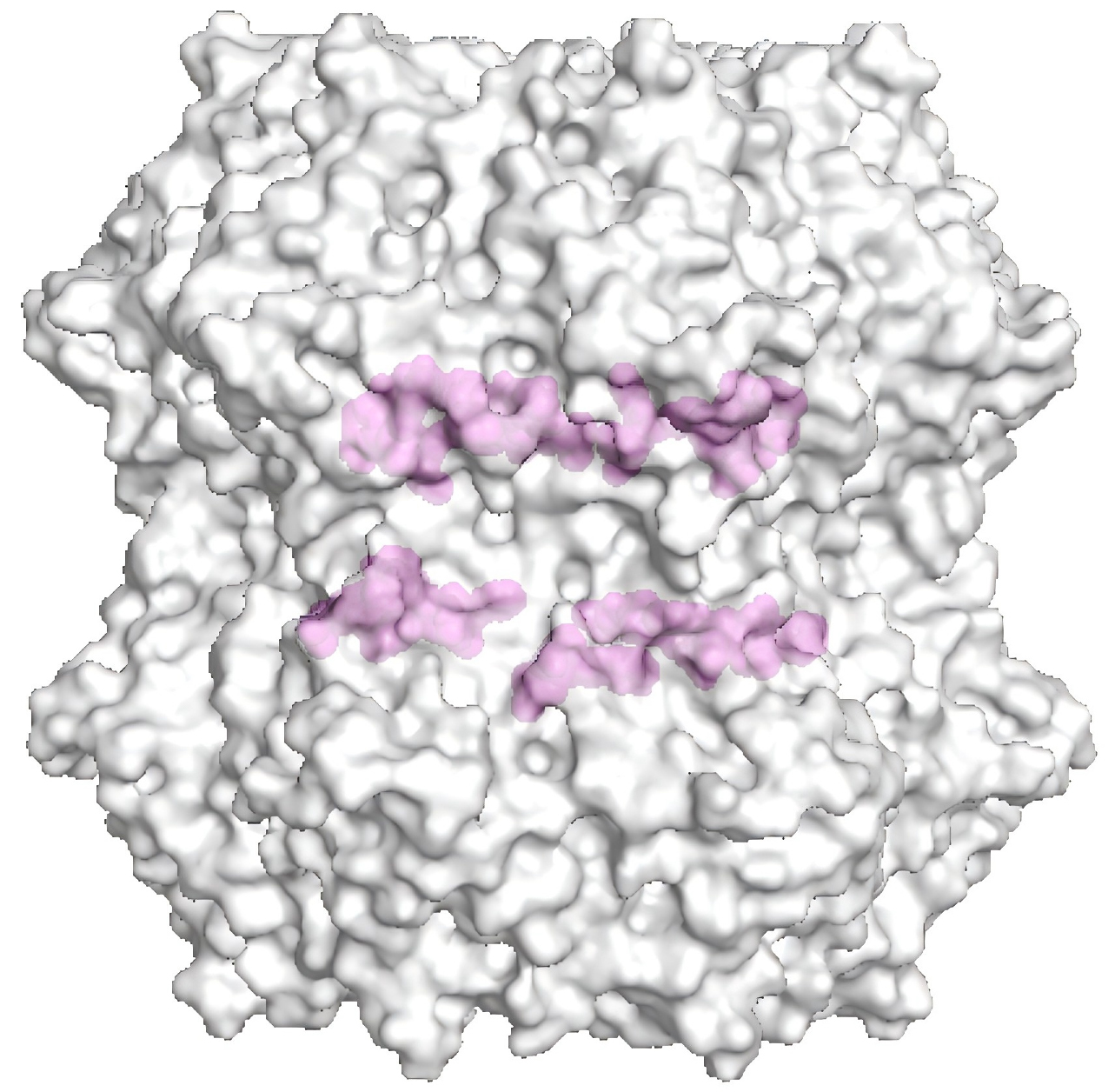

Supplement: S1 File — (ZIP) [file ppat.1013909.s010.zip › S7 Fig/S7F-Side view Fig.jpg]

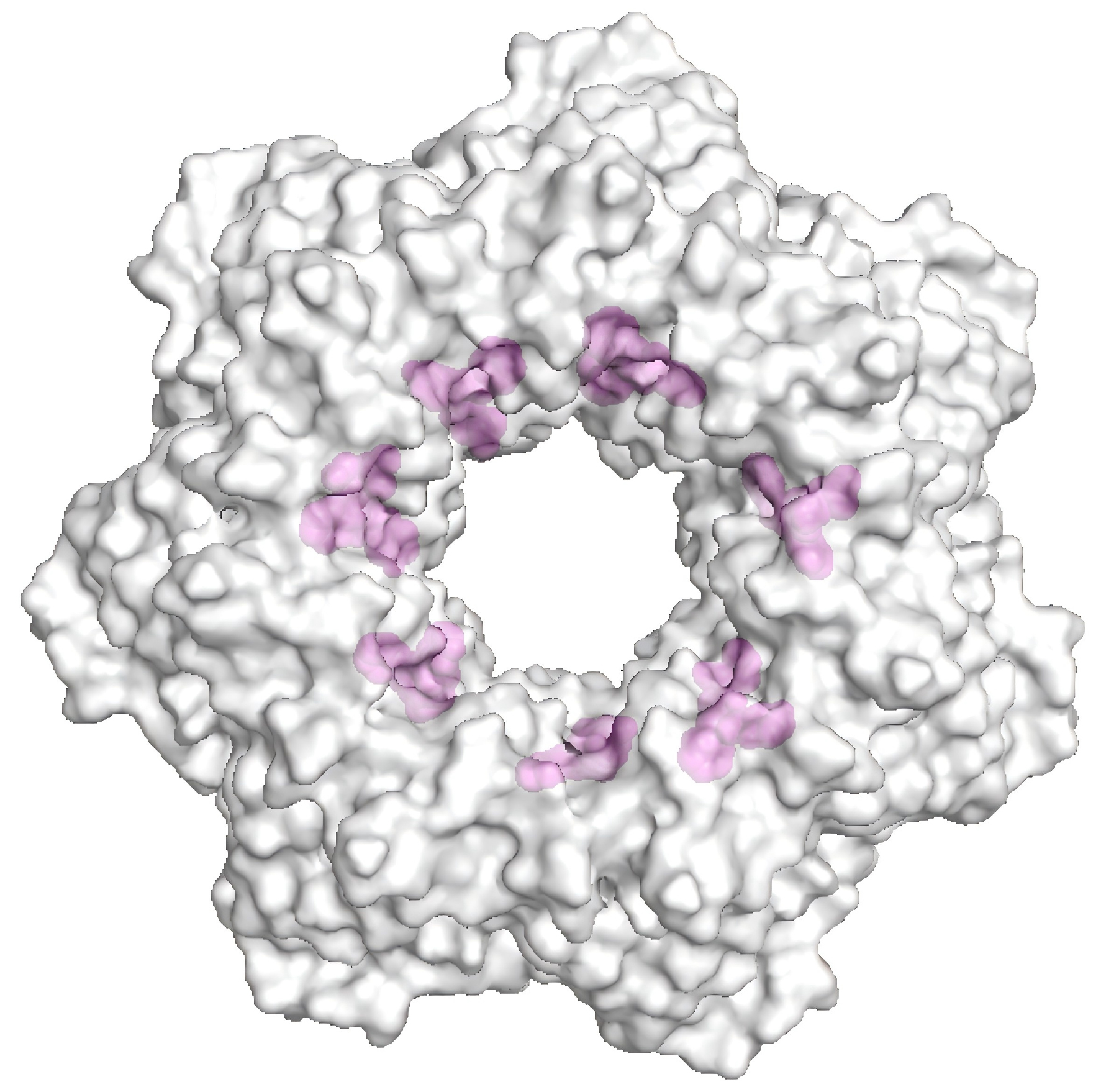

Supplement: S1 File — (ZIP) [file ppat.1013909.s010.zip › S7 Fig/S7F-Top view Fig.jpg]

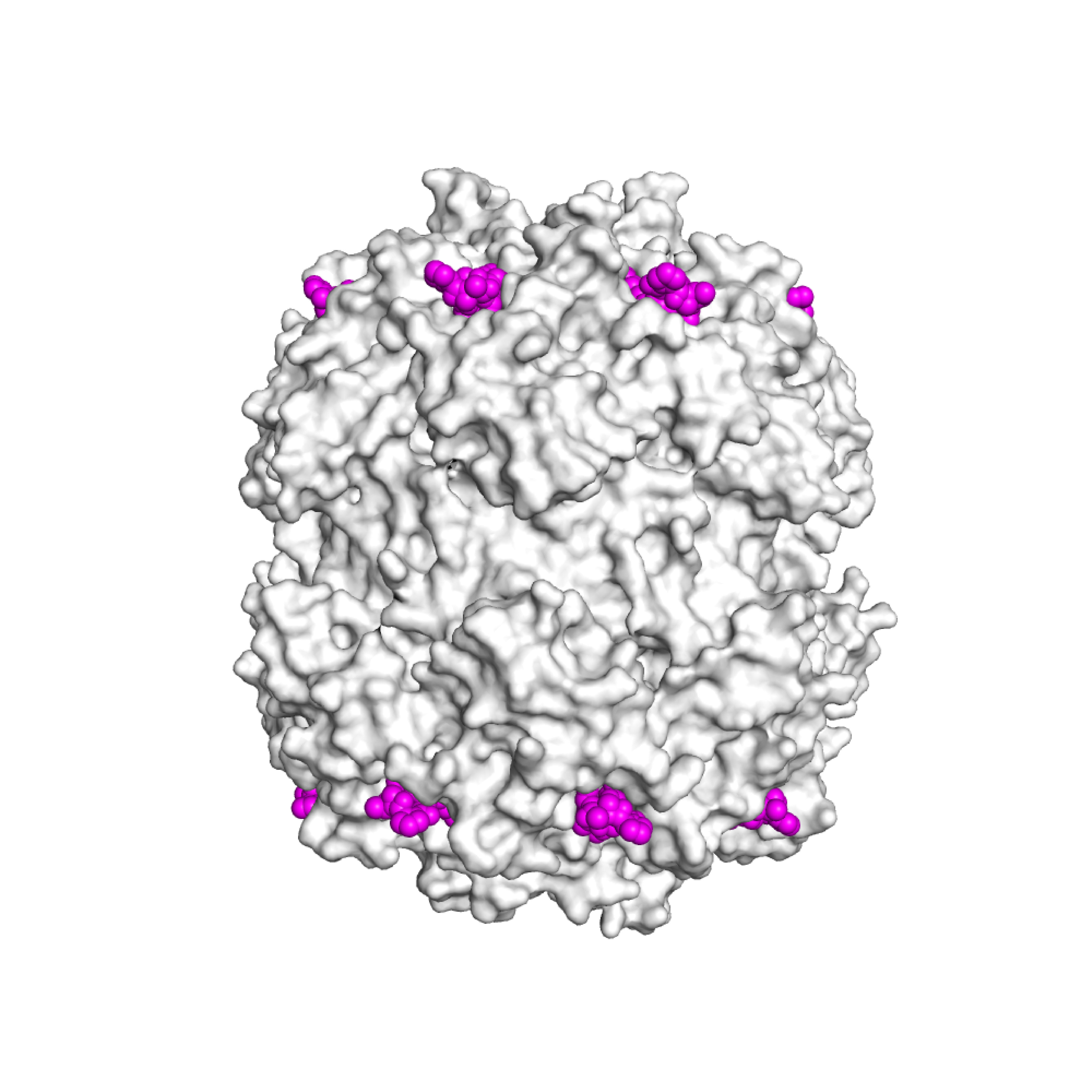

Supplement: S1 File — (ZIP) [file ppat.1013909.s010.zip › S7 Fig/S7G-Side view Fig.jpg]

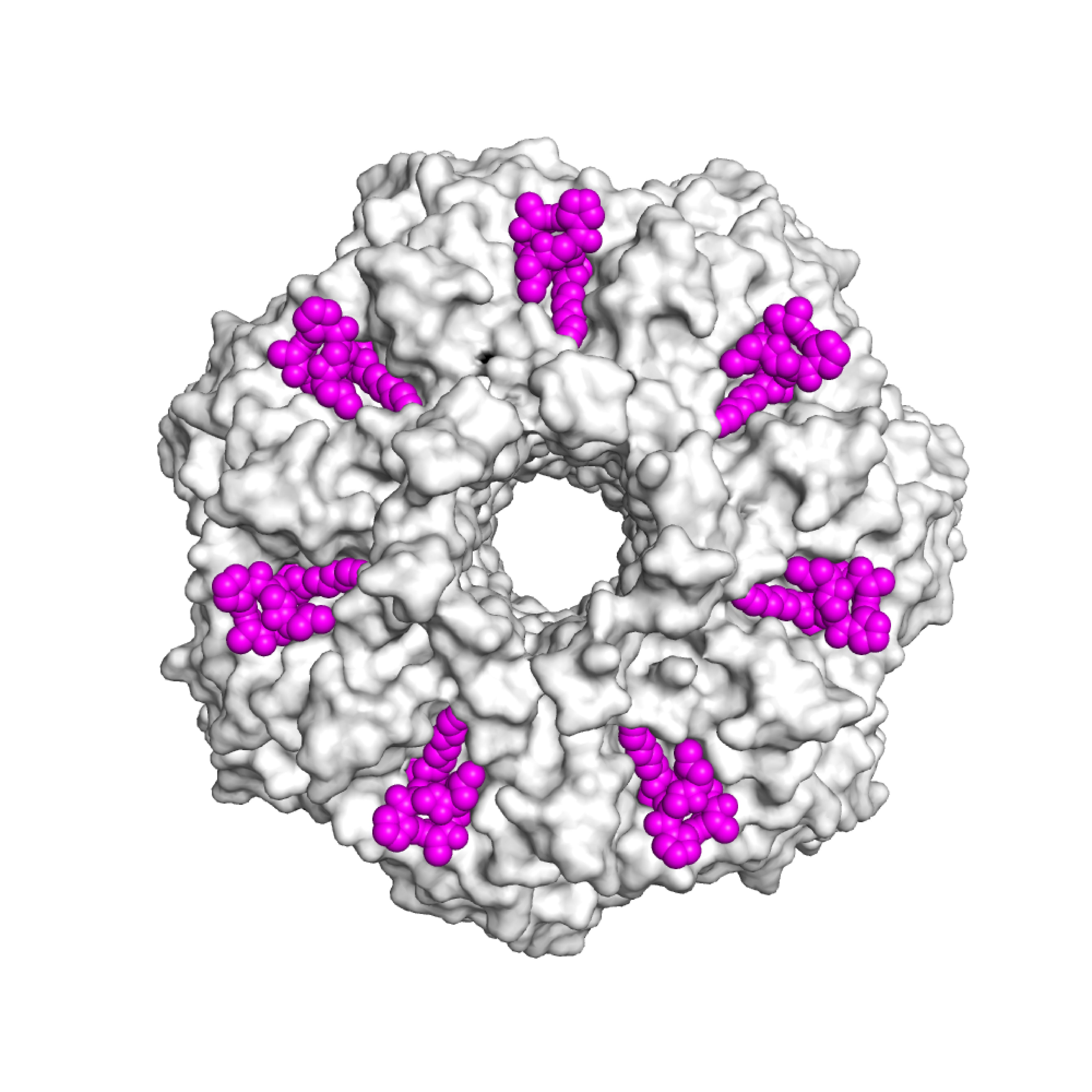

Supplement: S1 File — (ZIP) [file ppat.1013909.s010.zip › S7 Fig/S7G-Top view Fig.jpg]

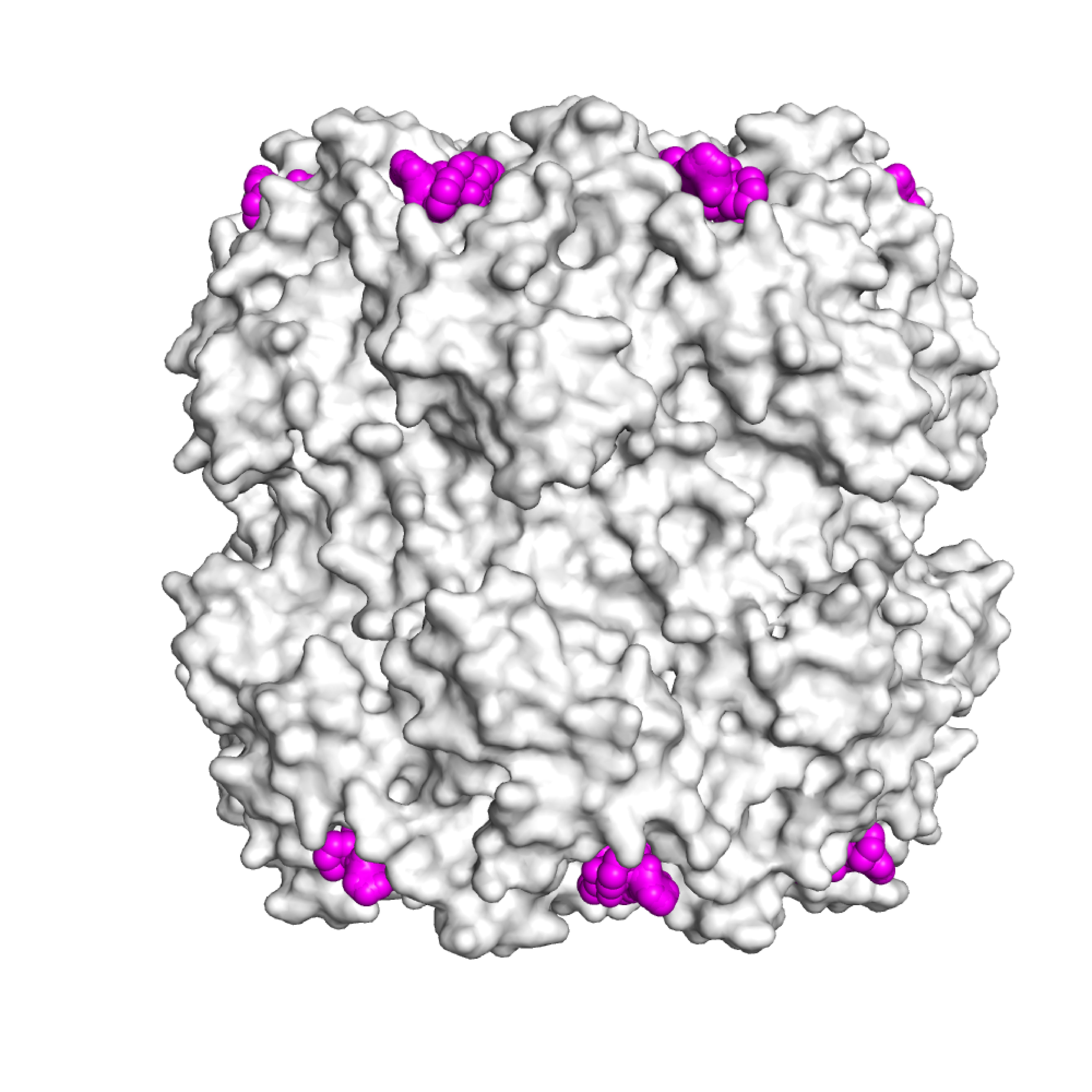

Supplement: S1 File — (ZIP) [file ppat.1013909.s010.zip › S7 Fig/S7H-Side view Fig.jpg]

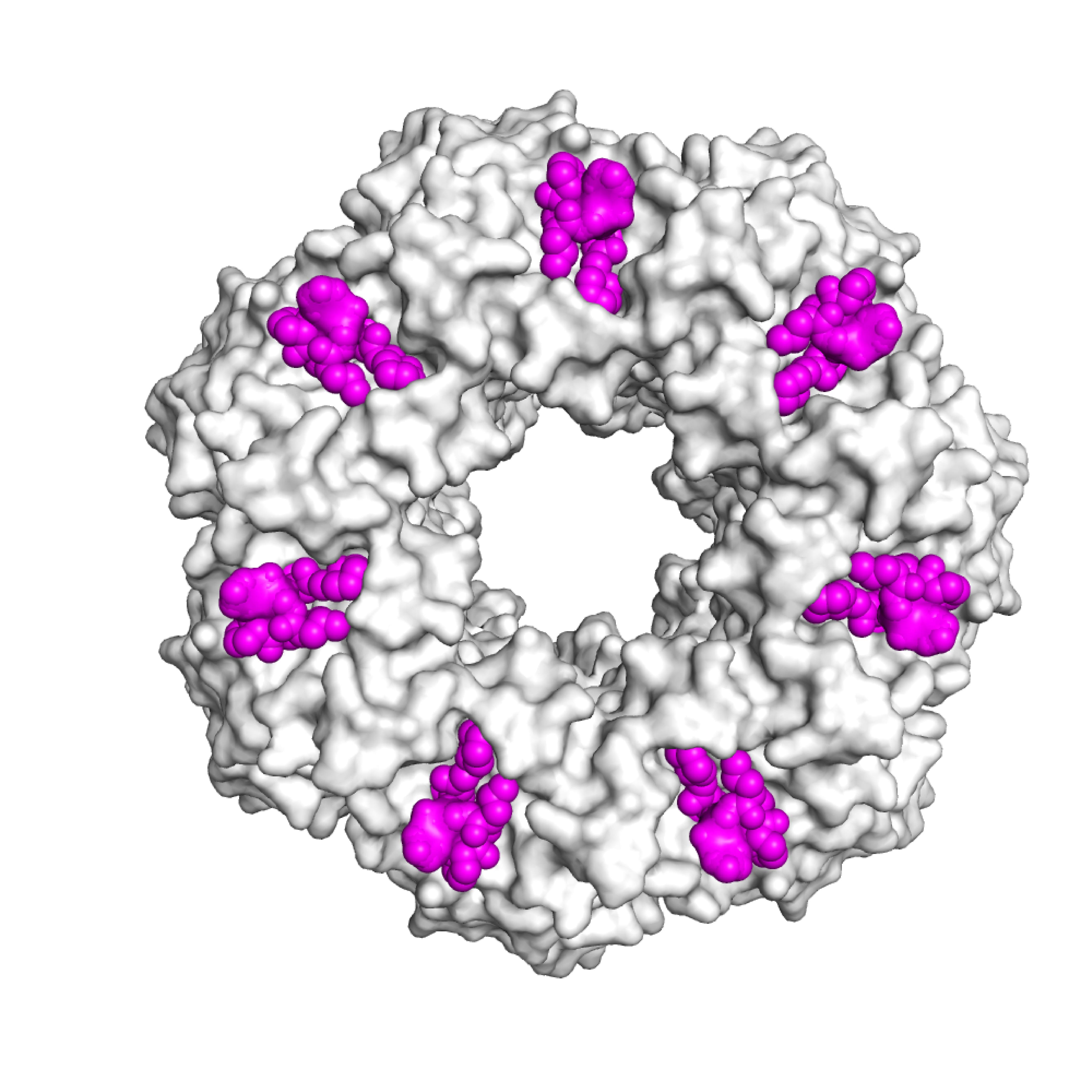

Supplement: S1 File — (ZIP) [file ppat.1013909.s010.zip › S7 Fig/S7H-Top view Fig.jpg]

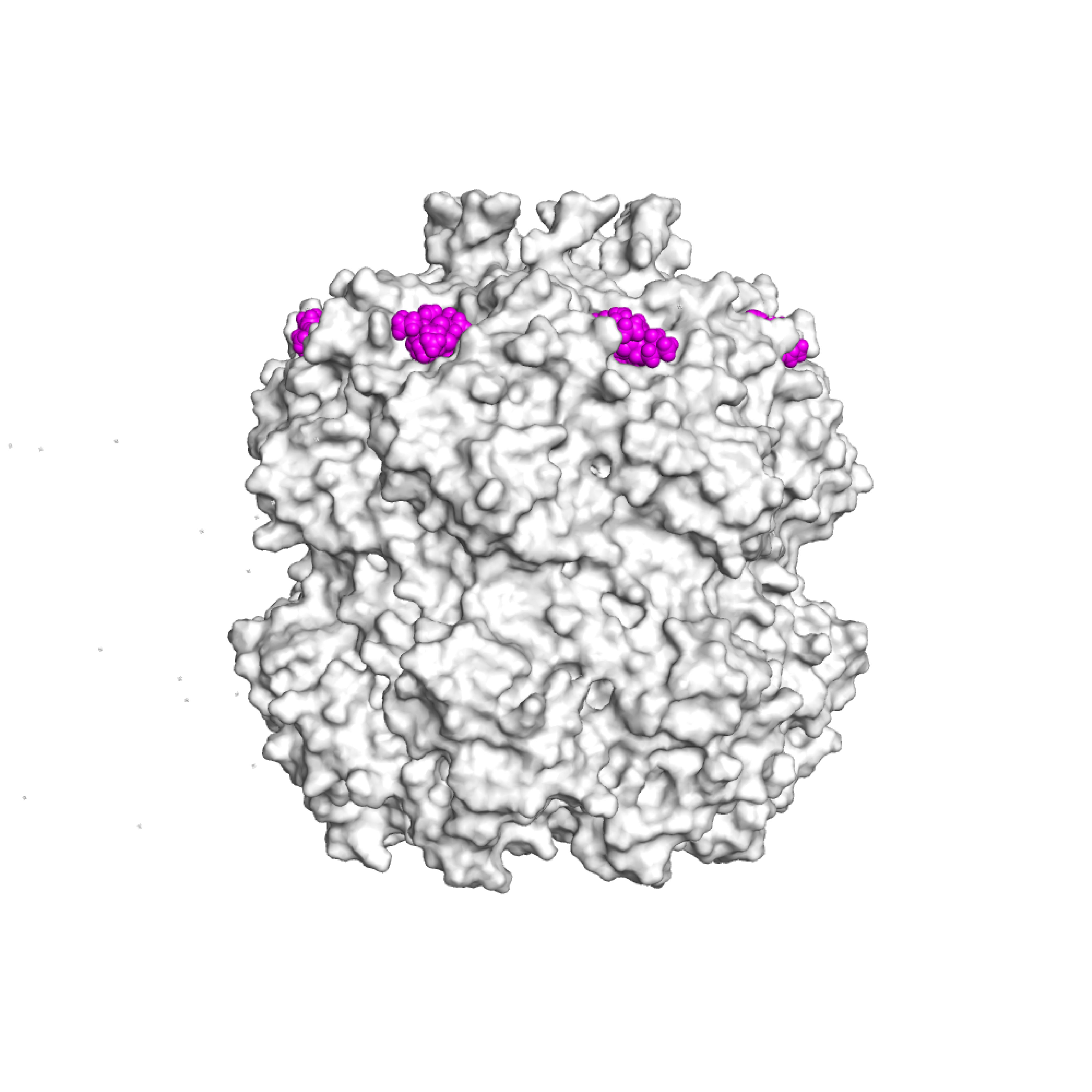

Supplement: S1 File — (ZIP) [file ppat.1013909.s010.zip › S7 Fig/S7I-Side view Fig.jpg]

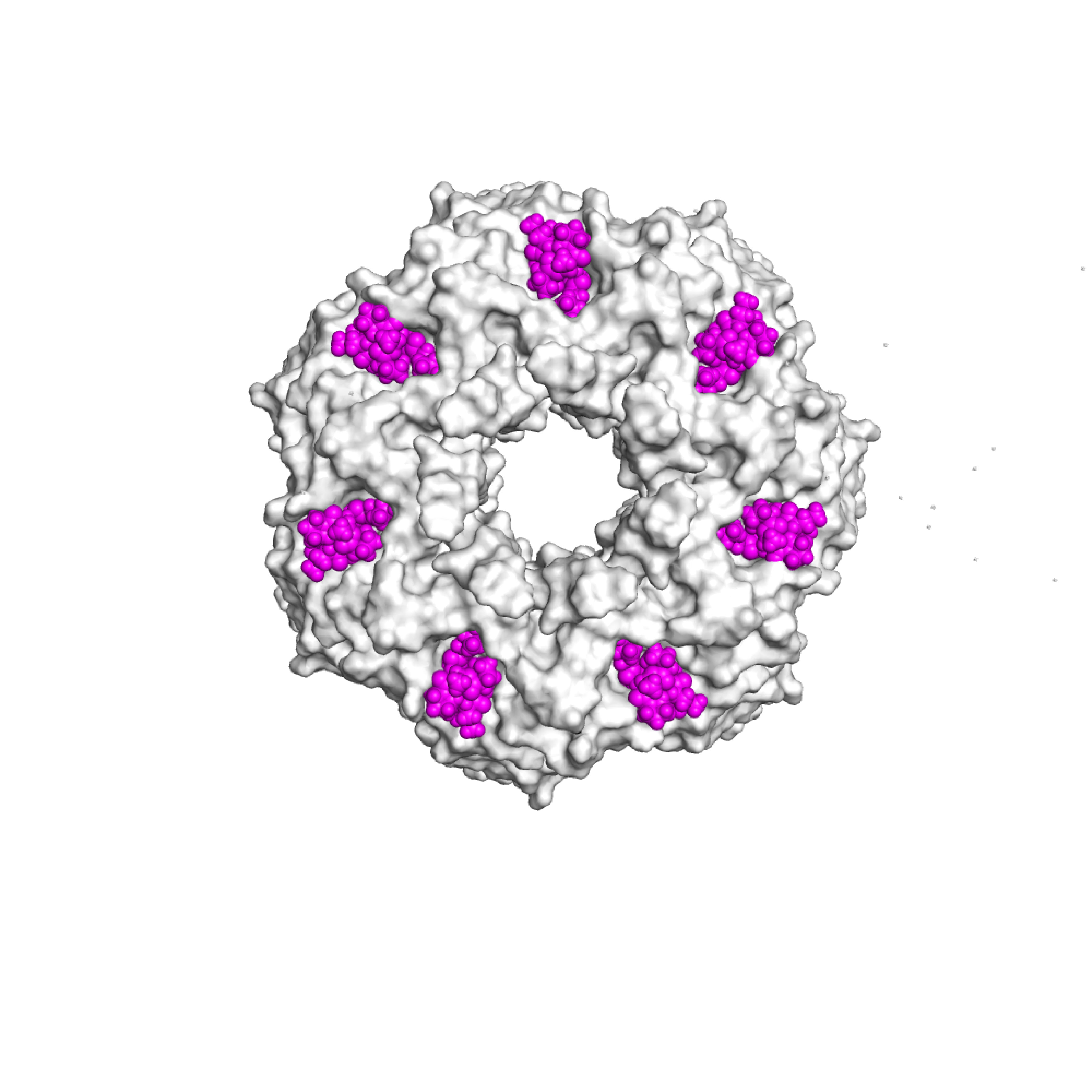

Supplement: S1 File — (ZIP) [file ppat.1013909.s010.zip › S7 Fig/S7I-Top view Fig.jpg]
