# Supplementary material for: Structural and mechanistic insights into caseinolytic protease inhibition for antimicrobial development against Pseudomonas plecoglossicida
Source: PLoS Pathog. 2026 Feb 12;22(2):e1013909. doi: 10.1371/journal.ppat.1013909 (PMC12900304; doi:10.1371/journal.ppat.1013909)
Supplement: S1 Table — (DOCX) [file ppat.1013909.s008.docx]

**S1 Table. Oligonucleotide primers used in this study**

| **Primer** | **primer sequence(5’→3’)** | **application** |
| --- | --- | --- |
| *Pp*ClpP1-pF | GGCGGATCCATGGCCAGACATATCATCCACT | Vector construction |
| *Pp*ClpP1-pR | GCGCTCGAGTCACGCGTGCACGCTCCACTG | Vector construction |
| *Pp*ClpP2-pF | GCGGGATCCATGTCCCGCAATTCTTATATT | Vector construction |
| *Pp*ClpP2-pR | GCGCTCGAGTCATCAGGAGGCCAGTTGCCGCTTGTC | Vector construction |
| *Pp*ClpX(65-414)-pF | GGCGGATCCATGTTGCCTTCGCCGAAAGAAAT | Vector construction |
| *Pp*ClpX(65-414)-pR | GCGCTCGAGTCAGTAGATCATCAGCGGCTGCG | Vector construction |
| *Pp*ClpX (full-length)-pF | GGCGGATCCATGACTGACACCCGTAACGG | Vector construction |
| *Pp*ClpX (full-length)-pR | GCGCTCGAGTCAGGCGTCGGGGGCGGCCT | Vector construction |
| *Pp*ClpP1-qF | CTGTACAACTACCTGCGGGG | Real-time PCR |
| *Pp*ClpP1-qR | TCGATGCTTCCTTCGGTACG | Real-time PCR |
| *Pp*ClpP2-qF | ACATGGCCAACCTGGTAGTG | Real-time PCR |
| *Pp*ClpP2-qR | TCGTAGATCGACATGCCAGC | Real-time PCR |
| *Pp*GyrB*-*qF | TGCTGAAGGACGAGCGTTCG | Real-time PCR |
| *Pp*GyrB*-*qR | ATCATCTTGCCGACAACAGC | Real-time PCR |
| 18Tc-Δ*Pp*ClpP1-U F | acgaattcgagctcggtacctcggcaaagttcccgcgc | Gene knockout |
| 18Tc-Δ*Pp*ClpP1-U R | gccaaactctggaggcaagcatggccagacatatcatcca | Gene knockout |
| 18Tc-Δ*Pp*ClpP1-D F | Tgggcattgcgaatgctcccggatgatatgtctggcca | Gene knockout |
| 18Tc-Δ*Pp*ClpP1-D R | aacgacggccagtgccaaggcatcactccatcaaatgt | Gene knockout |
| 18Tc-Δ*Pp*ClpP2-U F | ACGAATTCGAGCTCGGTATCAAGCCTGAGCAACTGCCGGCCGAGCTGTTCGAA | Gene knockout |
| 18Tc-Δ*Pp*ClpP2-U R | GCTCTGGCTCCTGGGTGTCCTGCGCTCACTCCCAAATAGTCATGG | Gene knockout |
| 18Tc-Δ*Pp*ClpP2-D F | GAGTGAGCGCAGGACACCCAGGAGCCAGAGCAGGTAGGTGTCGAA | Gene knockout |
| 18Tc-Δ*Pp*ClpP2-D R | AACGACGGCCAGTGCCAAGCGAGCAGGGTCTTGCCCGAGCCGGTCGGCCCGAT | Gene knockout |
| *Pp*ClpP1-F | atggccagacatatcatcca | Gene knockout |
| *Pp*ClpP1-R | cgcgtgcacgctccactggg | Gene knockout |
| *Pp*ClpP2-F | ATGTCCCGCAATTCTTATATT | Gene knockout |
| *Pp*ClpP2-R | TCAGGAGGCCAGTTGCCGCTTGTC | Gene knockout |
