# Supplementary material for: Structural and mechanistic insights into caseinolytic protease inhibition for antimicrobial development against Pseudomonas plecoglossicida
Source: PLoS Pathog. 2026 Feb 12;22(2):e1013909. doi: 10.1371/journal.ppat.1013909 (PMC12900304; doi:10.1371/journal.ppat.1013909)
Supplement: S2 Table — (DOCX) [file ppat.1013909.s009.docx]

**S2 Table. Cryo-EM statistics and model refinement of *Pp*ClpP1 tetradecamer**

| **Data collection and processing** | *Pp*ClpP1 |
| --- | --- |
| Magnification | 165,000 |
| Voltage (kV) | 200 |
| Electron exposure (e−/Å2) | 50 |
| Defocus range (μm) | -0.4 to -2.6 |
| Pixel size (Å) | 0.698 |
| Symmetry imposed | D7 |
| Initial particle images (no.) | 1,218,774 |
| Final particle images (no.) | 39,438 |
| Map resolution (Å) | 3.07 |
| FSC threshold | 0.143 |
| **Refinement** |  |
| Initial model used (PDB code) | 9UXT |
| Model resolution (Å) |  |
| FSC threshold | 0.5 |
| Map sharpening *B* factor (Å^2^) | -60 |
| **Model composition** |  |
| Nonhydrogen atoms | 19,992 |
| Protein residues | 2,562 |
| Ligands | 0 |
| *B* factors (Å^2^) |  |
| Protein | 54.43/82.01/62.88 |
| Ligands | 0 |
| R.m.s. deviation |  |
| Bond lengths (Å) | 0.003 |
| Bond angles (°) | 0.553 |
| **Validation** |  |
| MolProbity score | 1.56 |
| Clash score | 10.48 |
| rotamer outliers (%) | 0.00 |
| Ramachandran plot |  |
| Favored (%) | 97.91 |
| Allowed (%) | 2.09 |
| Outliers (%) | 0.00 |
