# Supplementary material for: Structural and mechanistic insights into caseinolytic protease inhibition for antimicrobial development against Pseudomonas plecoglossicida
Source: PLoS Pathog. 2026 Feb 12;22(2):e1013909. doi: 10.1371/journal.ppat.1013909 (PMC12900304; doi:10.1371/journal.ppat.1013909)
Supplement: S1 File — (ZIP) [file ppat.1013909.s010.zip › Fig 9/Figure 9.pptx]

## Slide 1
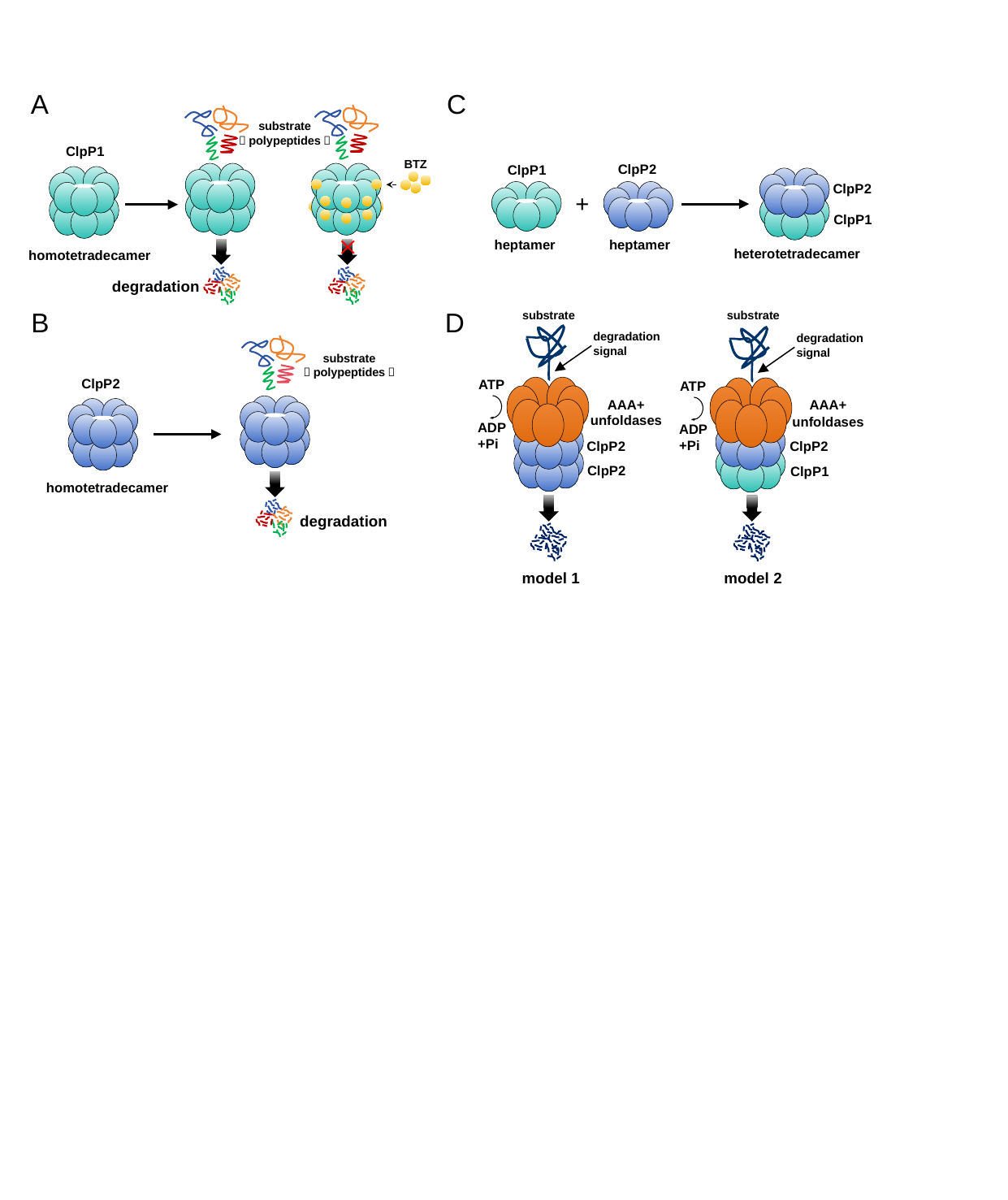

A
C
BTZ
×
substrate
（polypeptides）
ClpP1
homotetradecamer
degradation
ClpP2
ClpP1
ClpP2
ClpP1
+
 heptamer
 heptamer
heterotetradecamer
B
D
substrate
degradation
signal
ATP
ADP
+Pi
AAA+ unfoldases
ClpP2
ClpP2
substrate
degradation
signal
ATP
ADP
+Pi
AAA+ unfoldases
ClpP2
ClpP1
substrate
（polypeptides）
degradation
ClpP2
homotetradecamer
model 1
model 2
