# Supplementary figures and images for: Structural and mechanistic insights into caseinolytic protease inhibition for antimicrobial development against Pseudomonas plecoglossicida (part 2 of 2)
Source: PLoS Pathog. 2026 Feb 12;22(2):e1013909. doi: 10.1371/journal.ppat.1013909 (PMC12900304; doi:10.1371/journal.ppat.1013909)

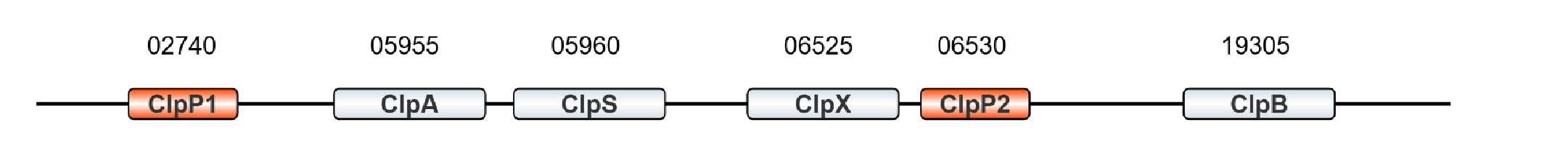

Supplement: S1 File — (ZIP) [file ppat.1013909.s010.zip › Fig 1/Fig 1A.tif]

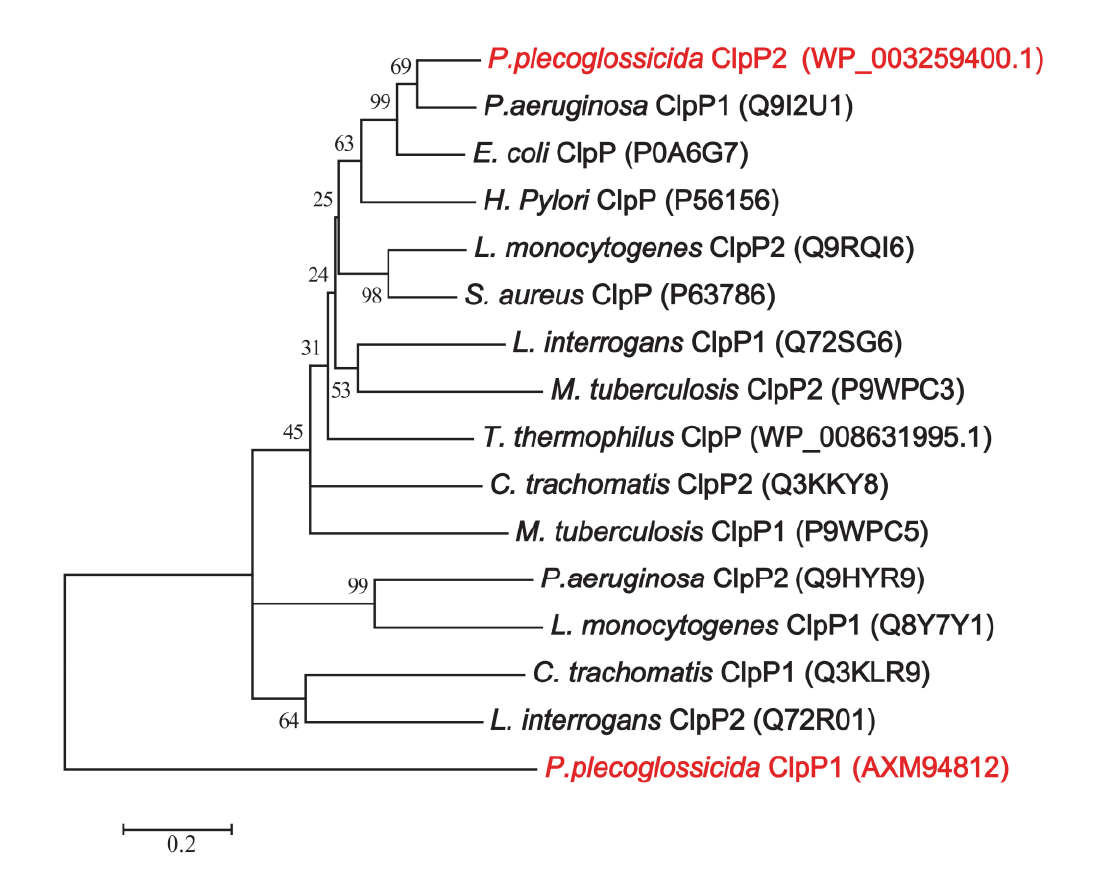

Supplement: S1 File — (ZIP) [file ppat.1013909.s010.zip › Fig 1/Fig 1B.tif]

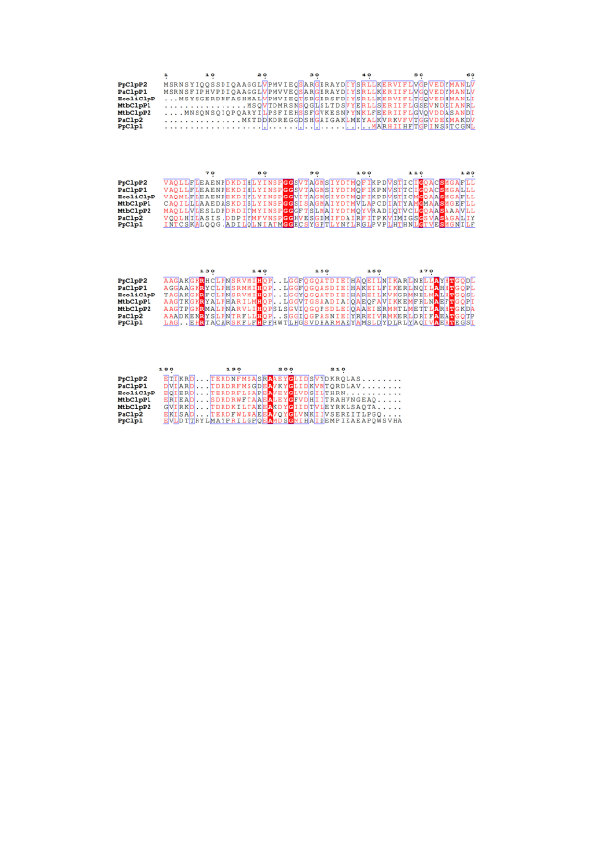

Supplement: S1 File — (ZIP) [file ppat.1013909.s010.zip › Fig 1/Fig 1C.tif]

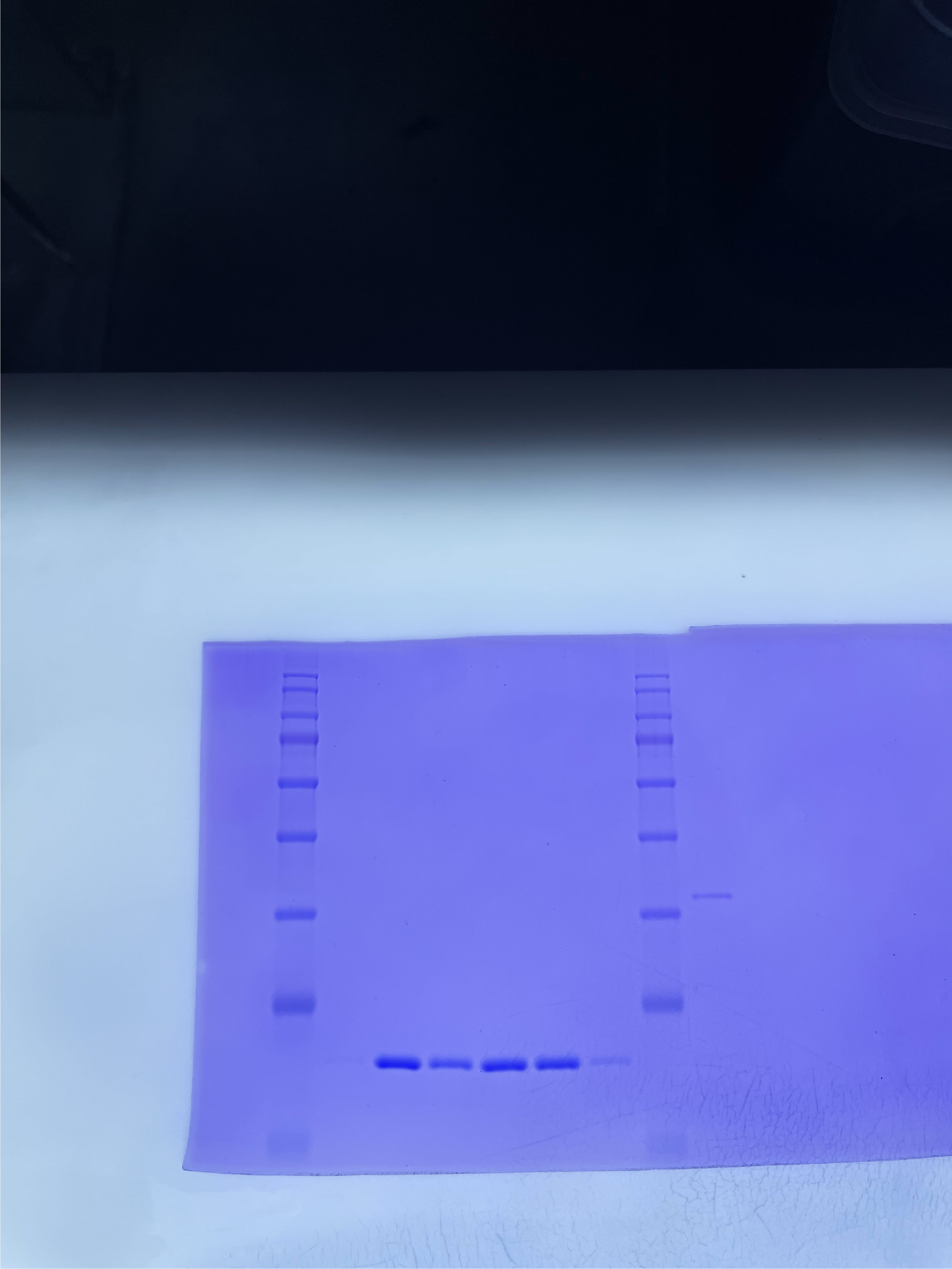

Supplement: S1 File — (ZIP) [file ppat.1013909.s010.zip › Fig 1/Fig 1D-gel.jpg]

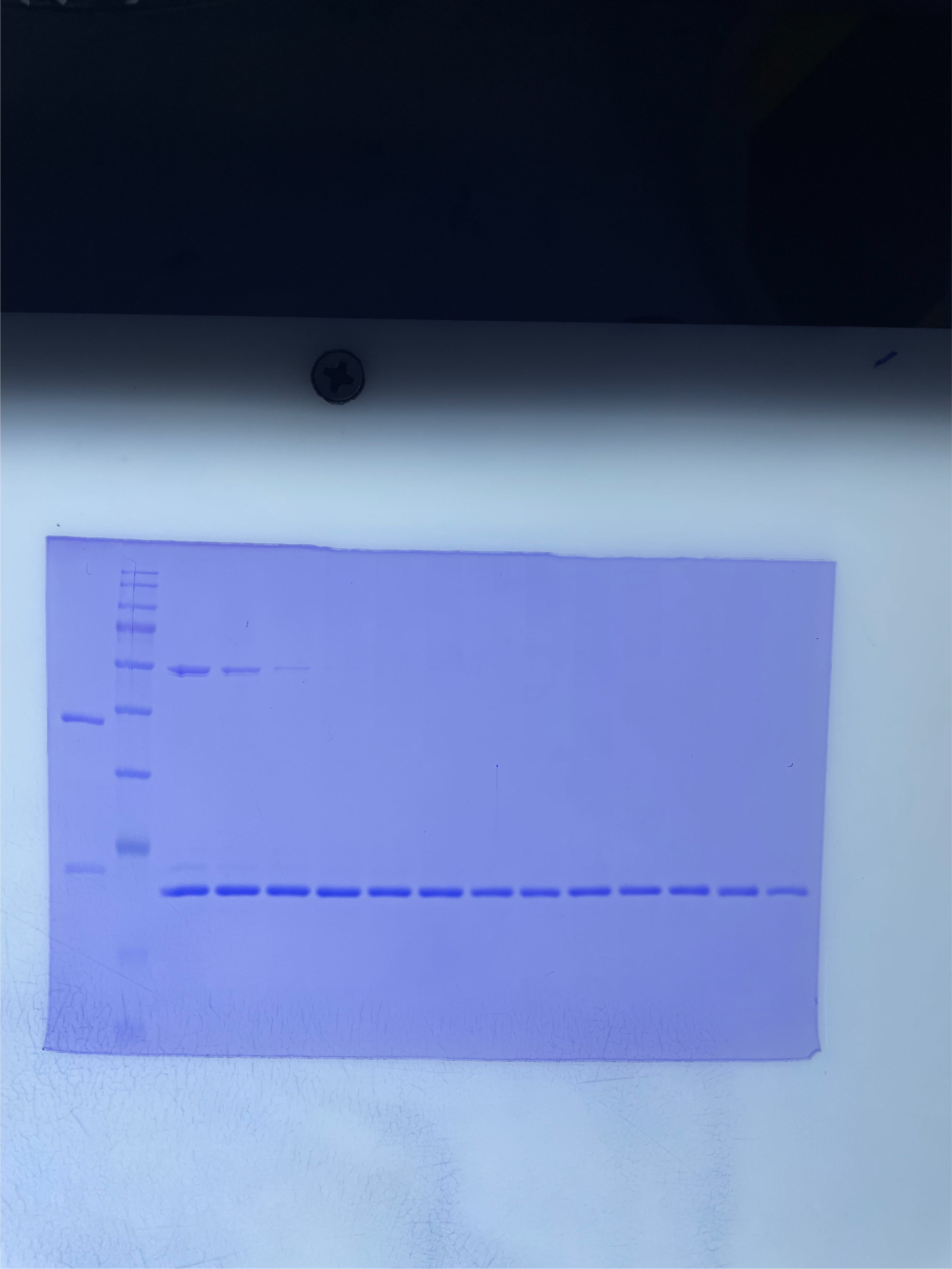

Supplement: S1 File — (ZIP) [file ppat.1013909.s010.zip › Fig 1/Fig 1E-gel.jpg]

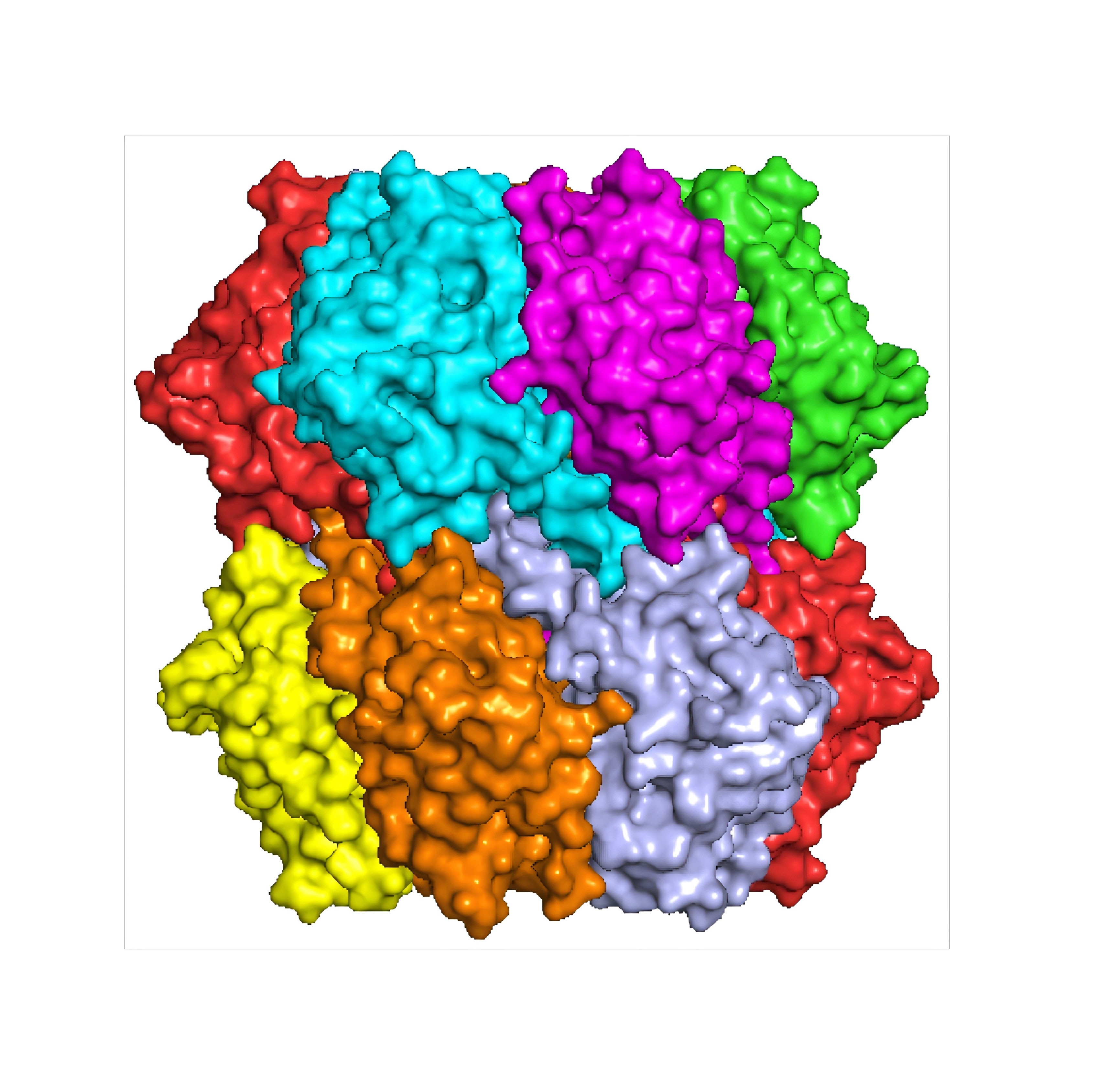

Supplement: S1 File — (ZIP) [file ppat.1013909.s010.zip › Fig 2/Fig 2A-1.jpg]

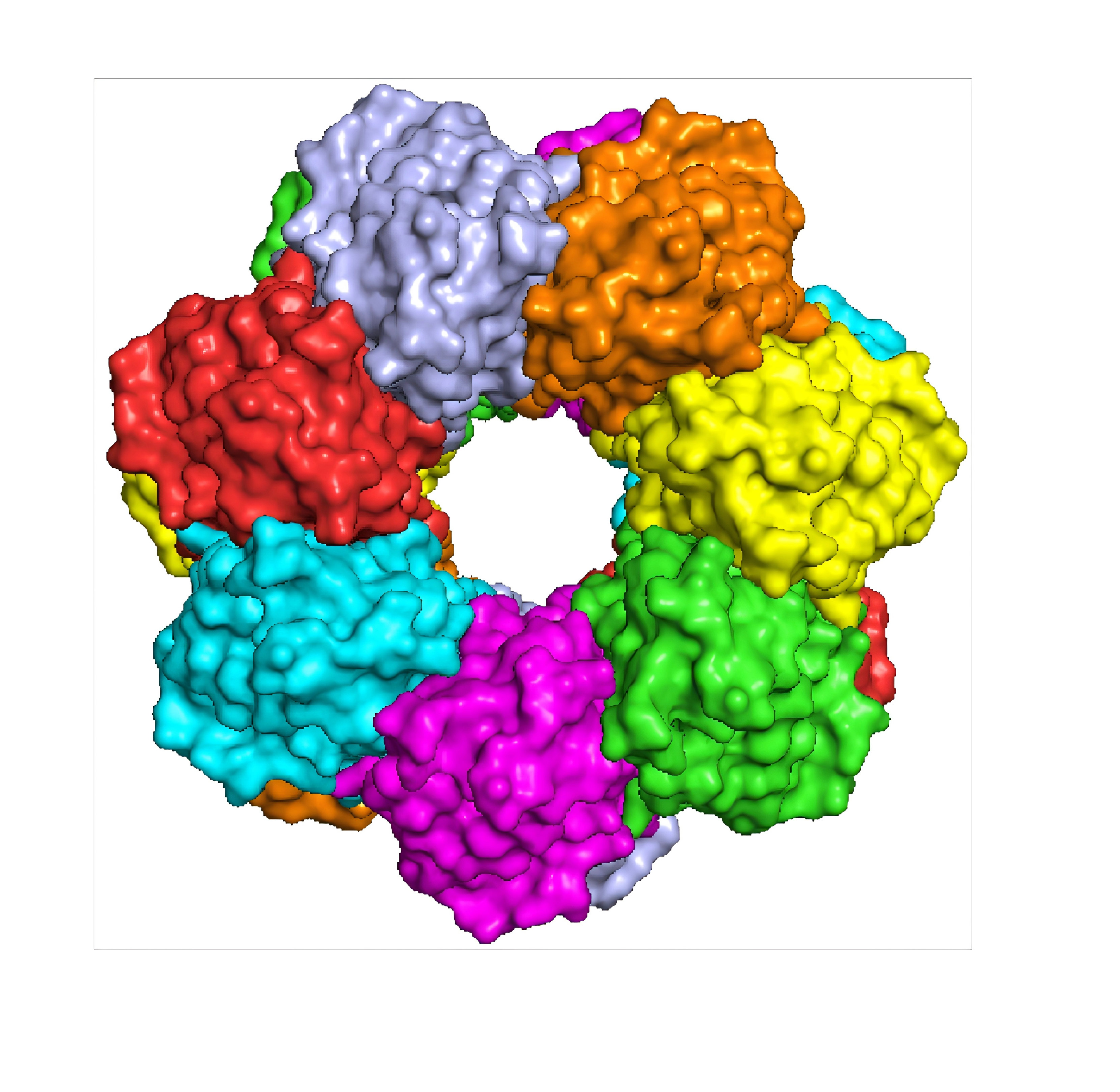

Supplement: S1 File — (ZIP) [file ppat.1013909.s010.zip › Fig 2/Fig 2A-2.jpg]

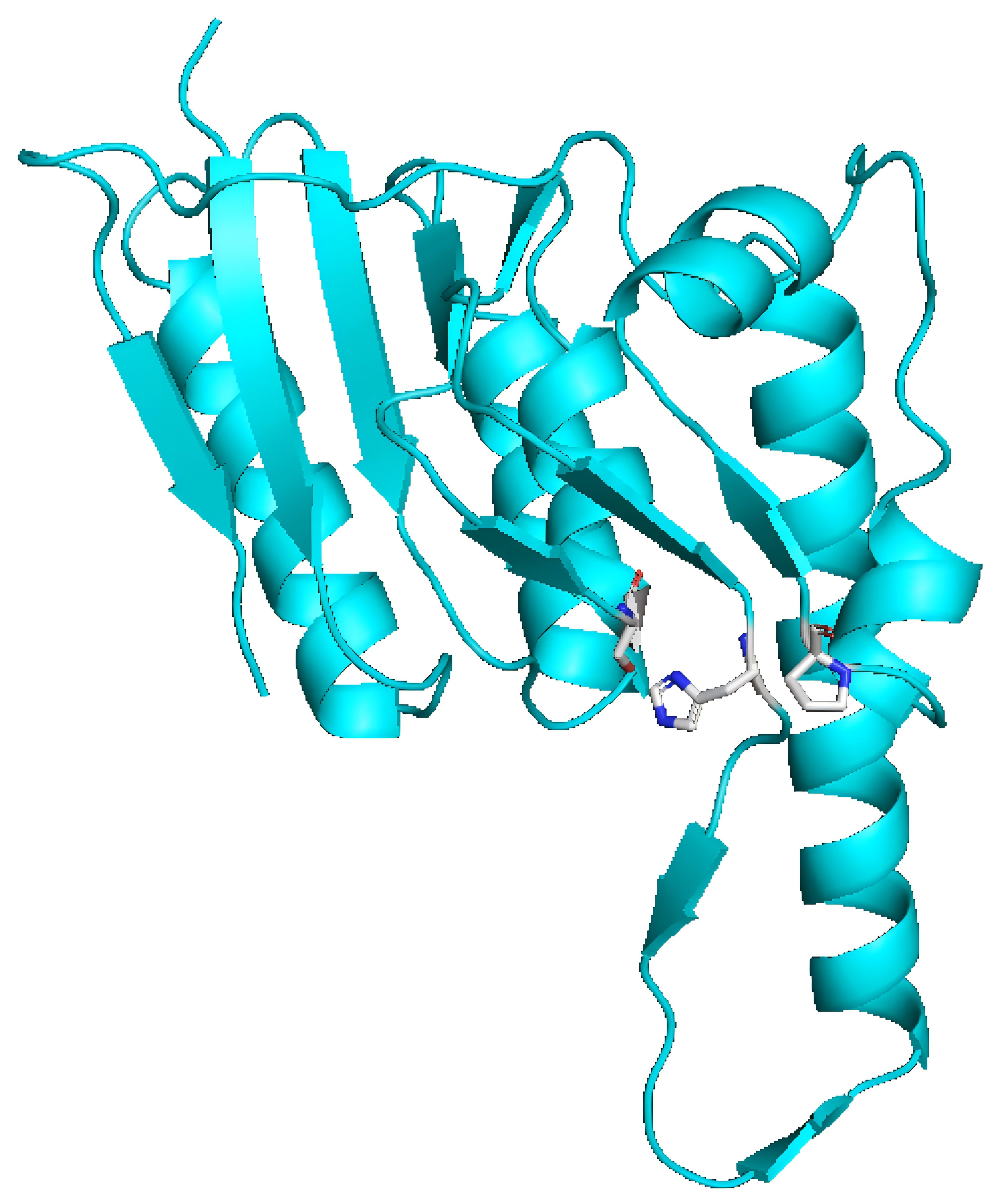

Supplement: S1 File — (ZIP) [file ppat.1013909.s010.zip › Fig 2/Fig 2B.jpg]

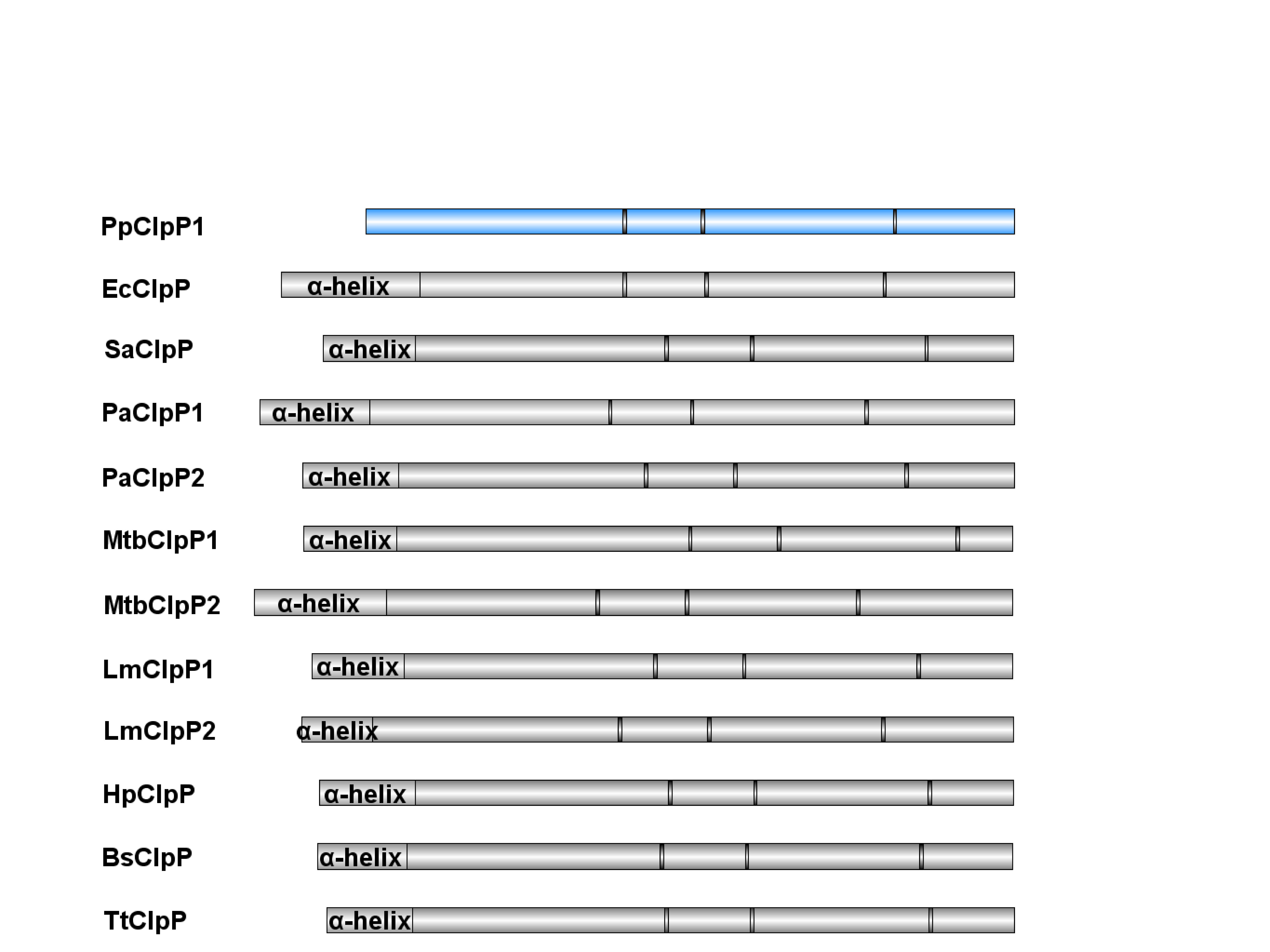

Supplement: S1 File — (ZIP) [file ppat.1013909.s010.zip › Fig 3/Fig 3A.jpg]

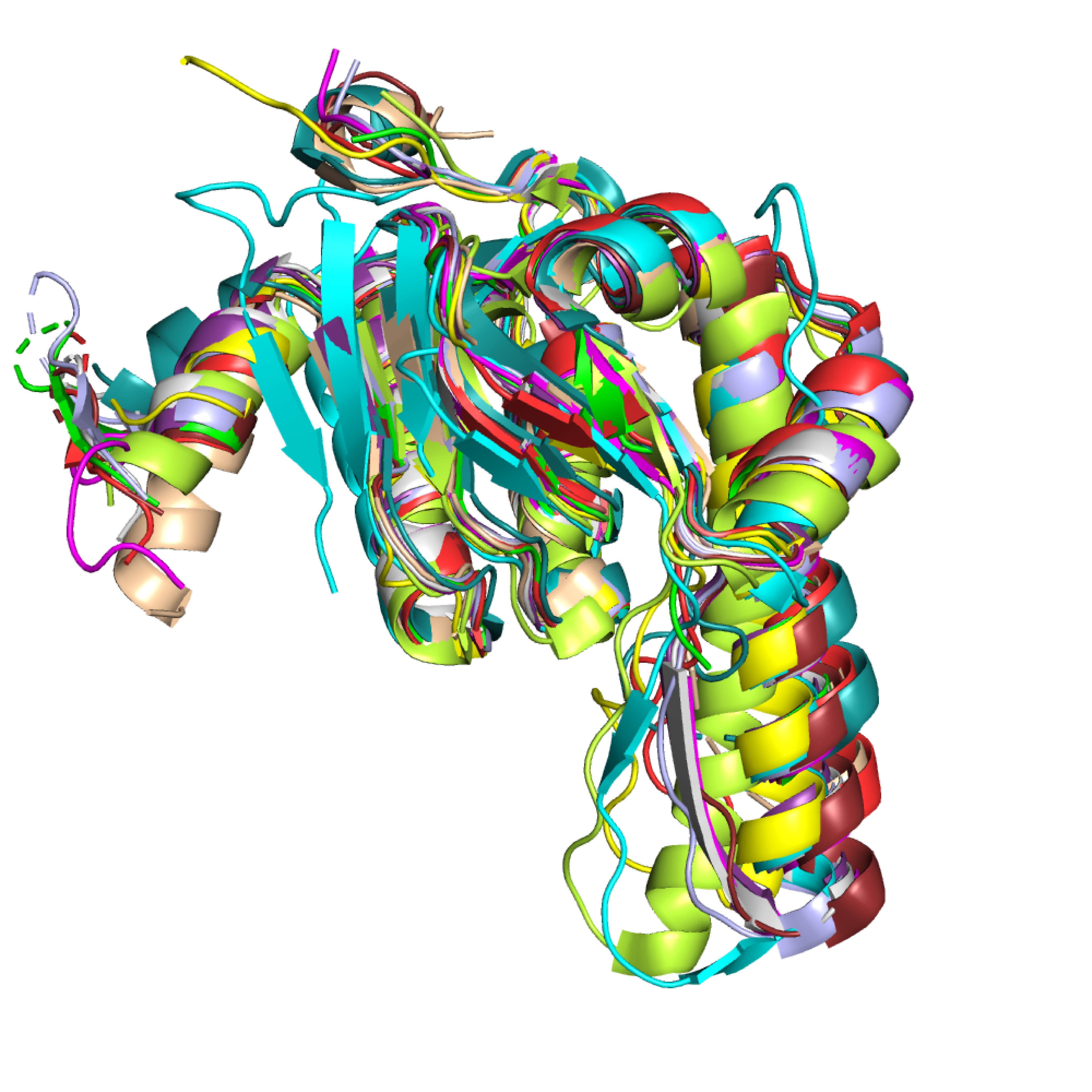

Supplement: S1 File — (ZIP) [file ppat.1013909.s010.zip › Fig 3/Fig 3B-a.jpg]

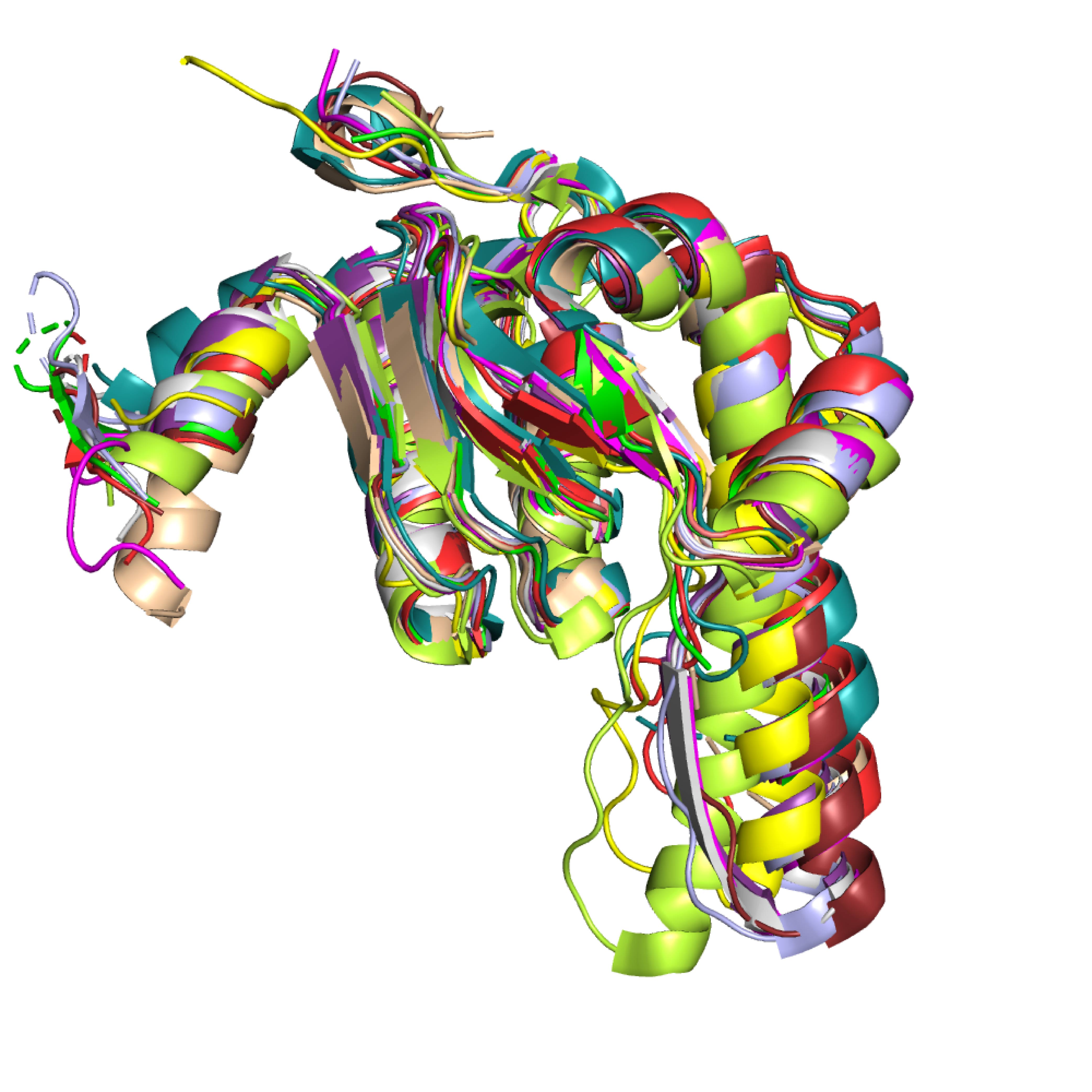

Supplement: S1 File — (ZIP) [file ppat.1013909.s010.zip › Fig 3/Fig 3B-b.jpg]

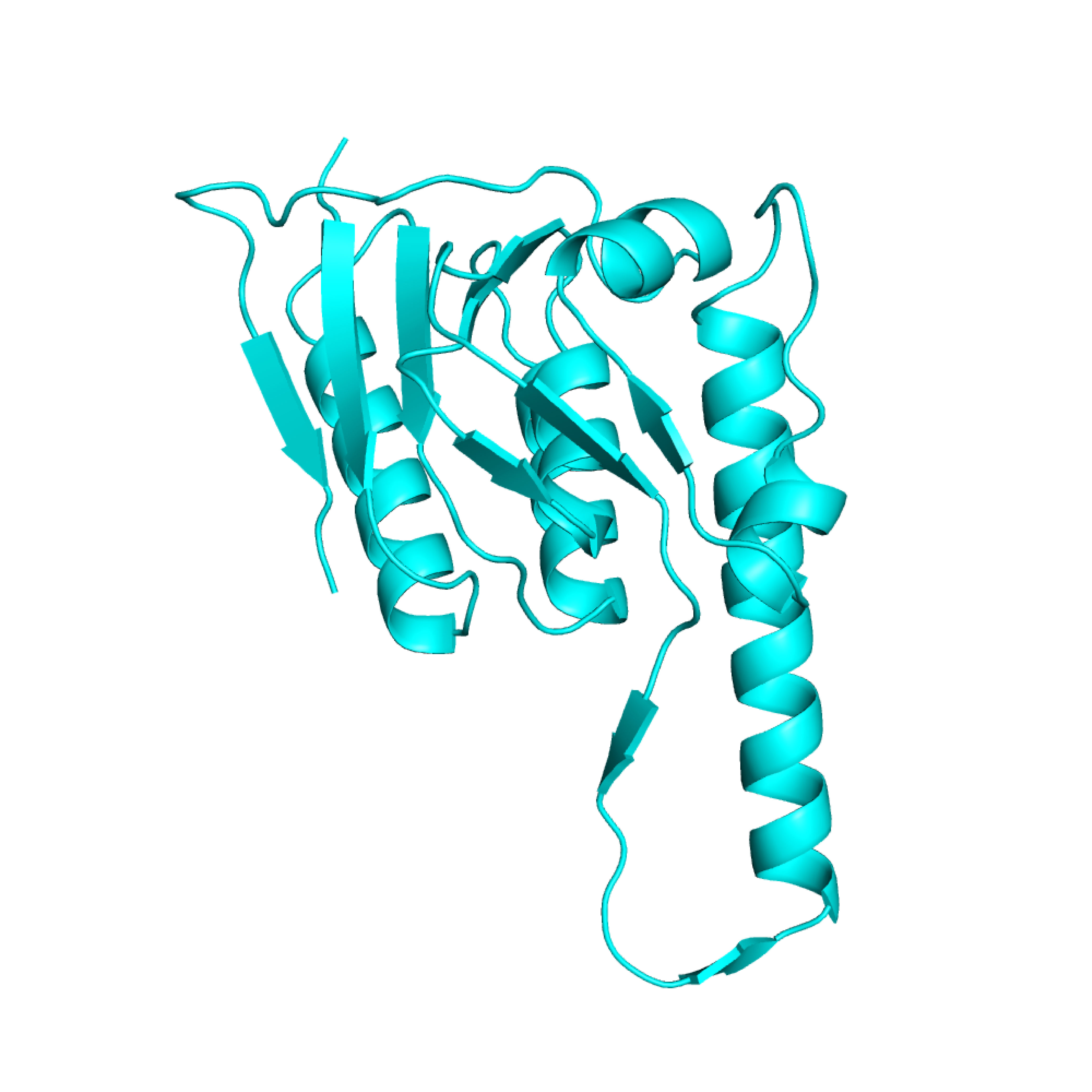

Supplement: S1 File — (ZIP) [file ppat.1013909.s010.zip › Fig 3/Fig 3B-c.jpg]

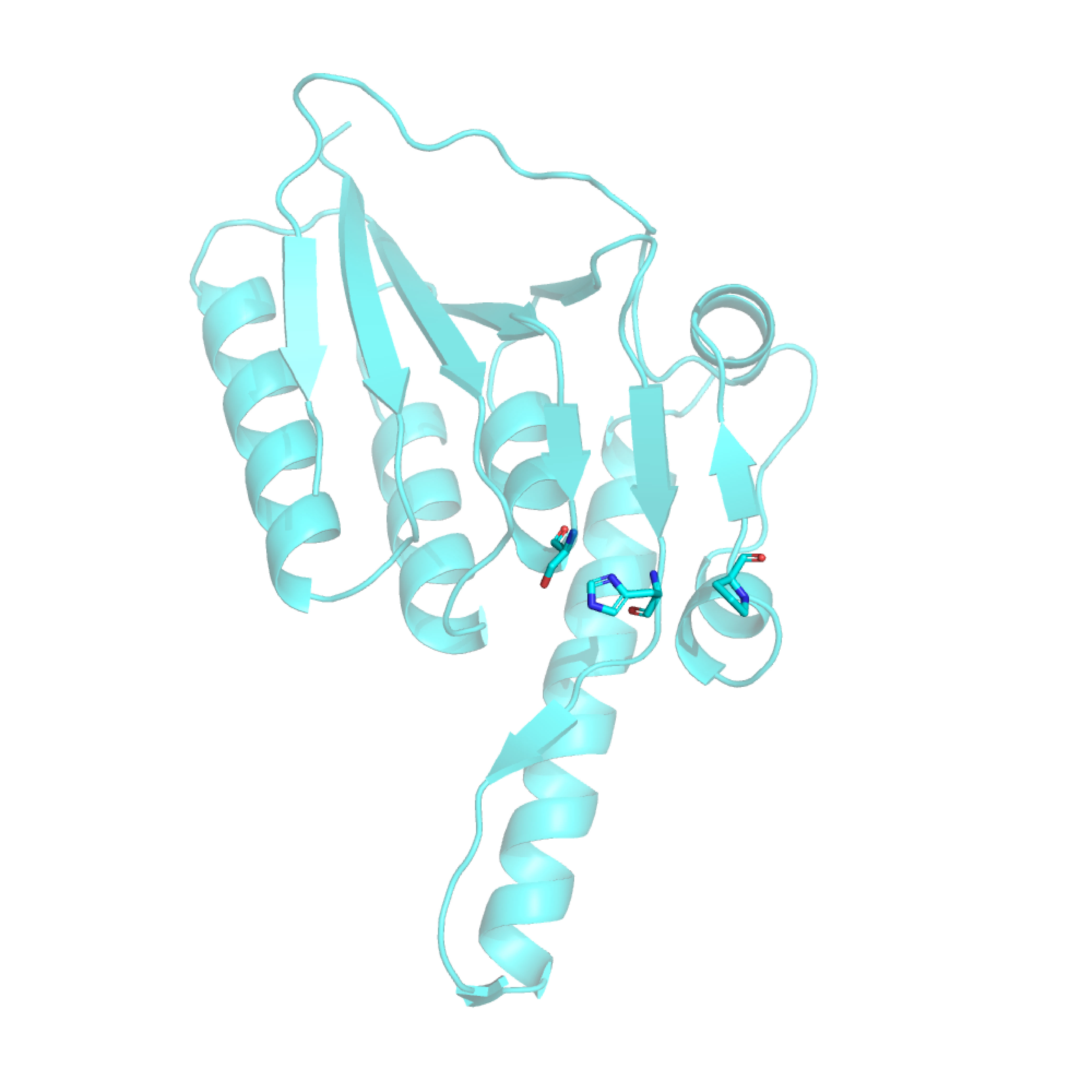

Supplement: S1 File — (ZIP) [file ppat.1013909.s010.zip › Fig 3/Fig 3C-a.jpg]

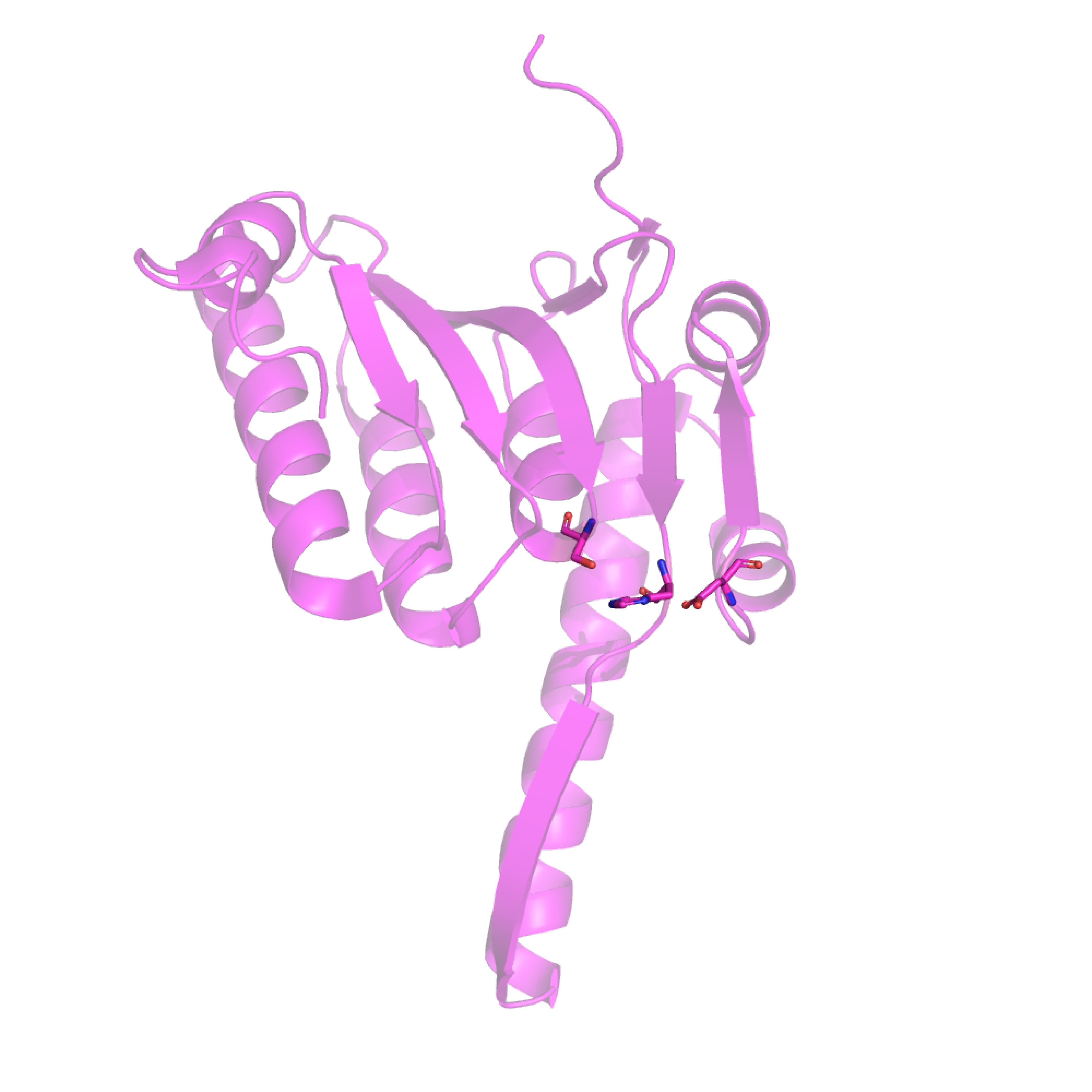

Supplement: S1 File — (ZIP) [file ppat.1013909.s010.zip › Fig 3/Fig 3C-b.jpg]

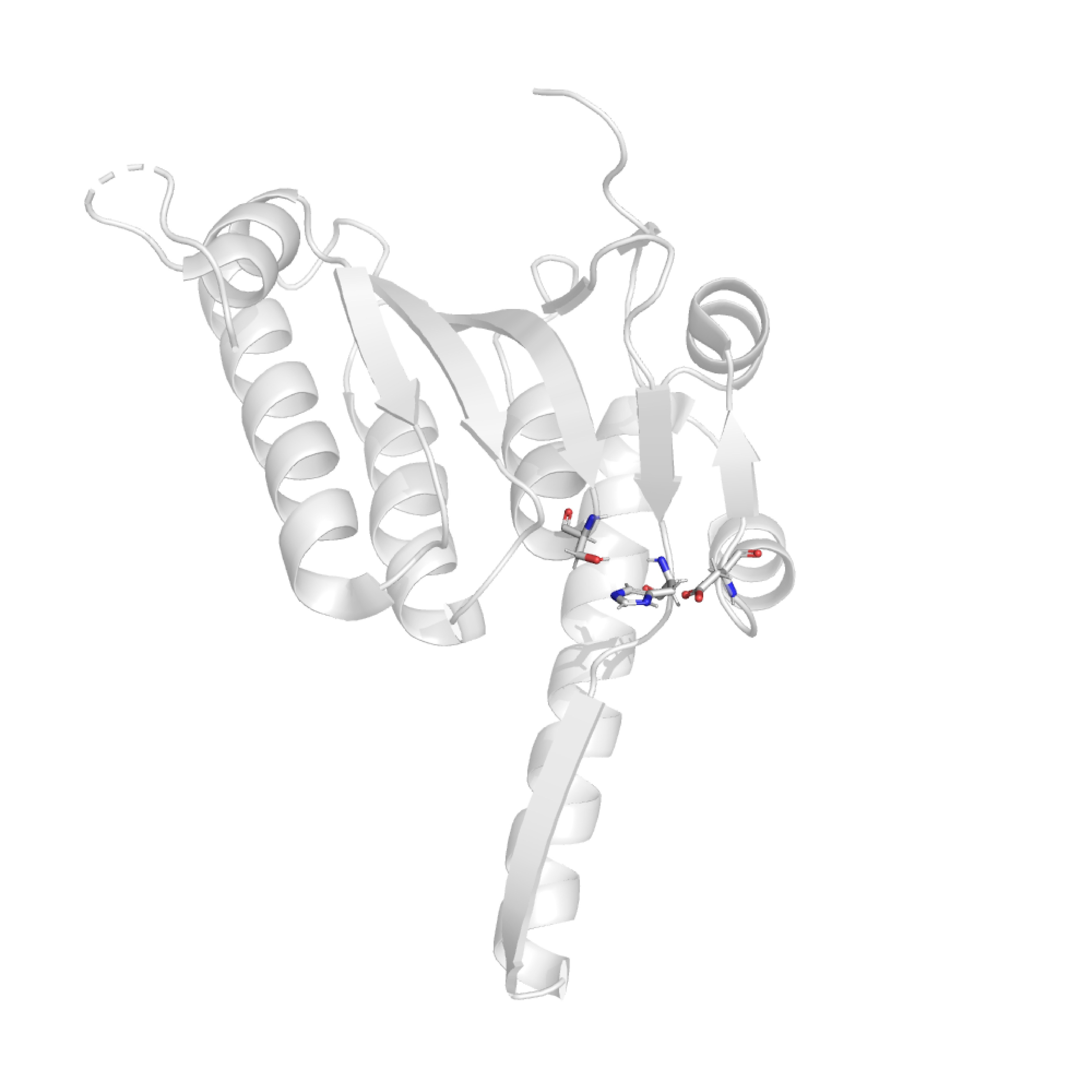

Supplement: S1 File — (ZIP) [file ppat.1013909.s010.zip › Fig 3/Fig 3C-c.jpg]

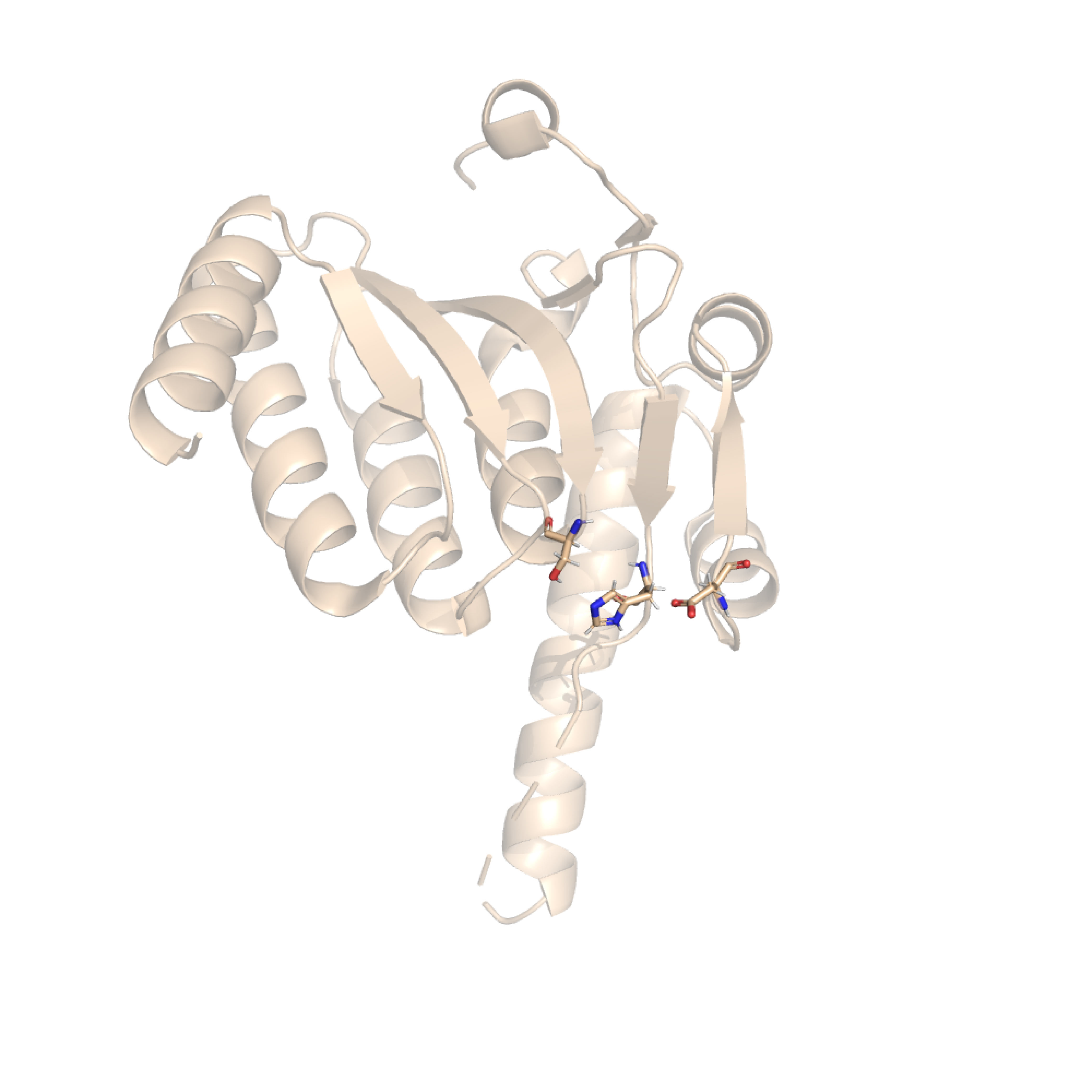

Supplement: S1 File — (ZIP) [file ppat.1013909.s010.zip › Fig 3/Fig 3C-d.jpg]

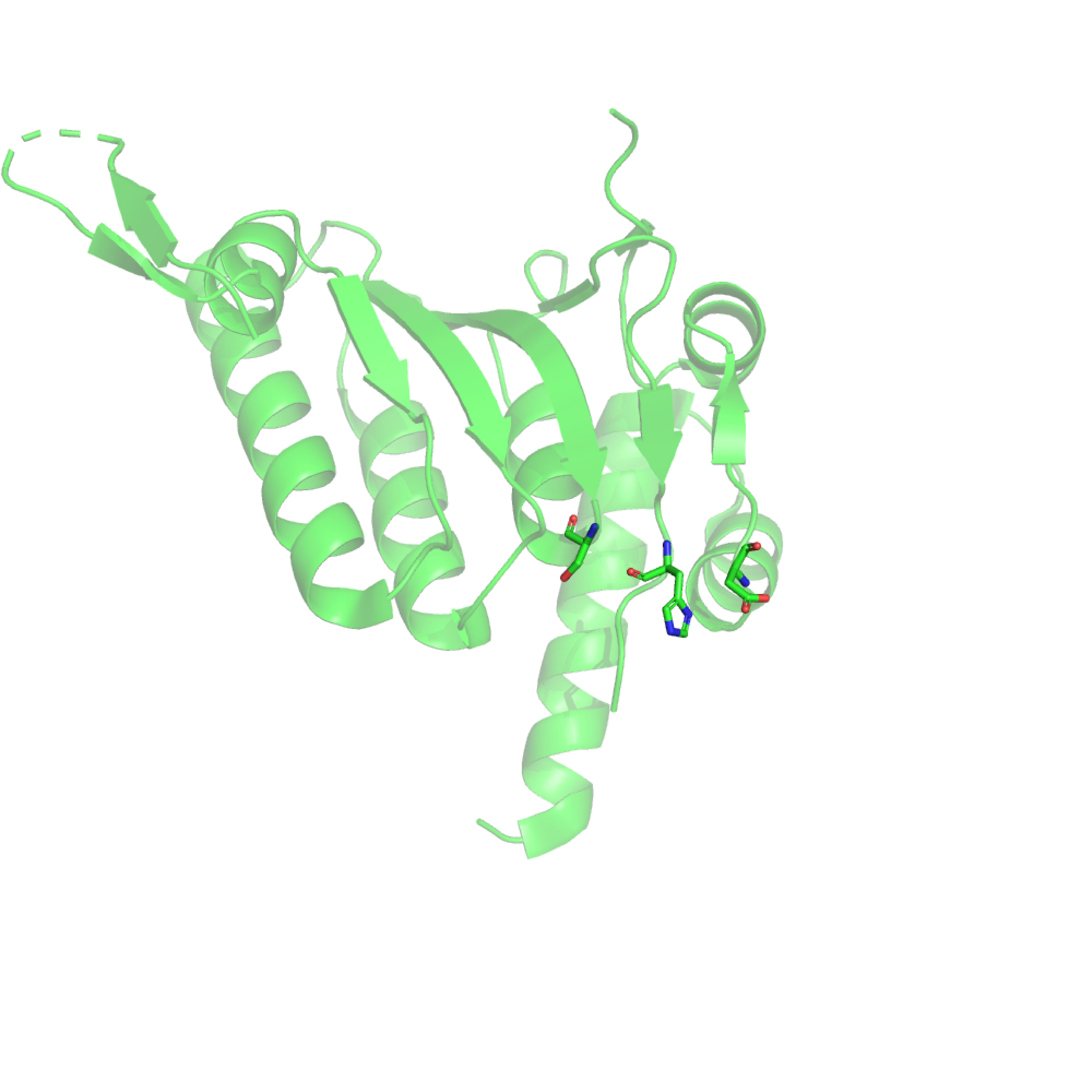

Supplement: S1 File — (ZIP) [file ppat.1013909.s010.zip › Fig 3/Fig 3C-e.jpg]

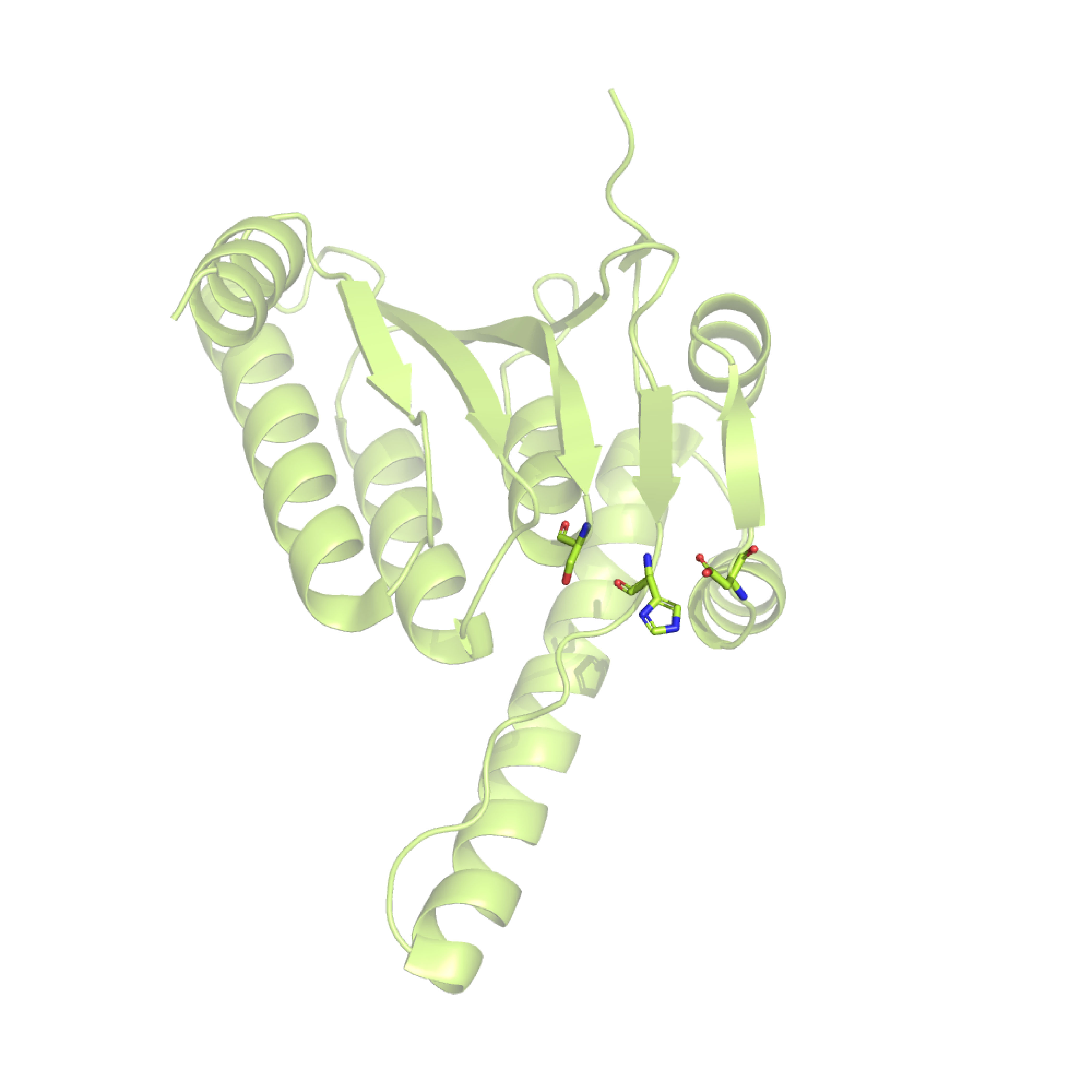

Supplement: S1 File — (ZIP) [file ppat.1013909.s010.zip › Fig 3/Fig 3C-f.jpg]

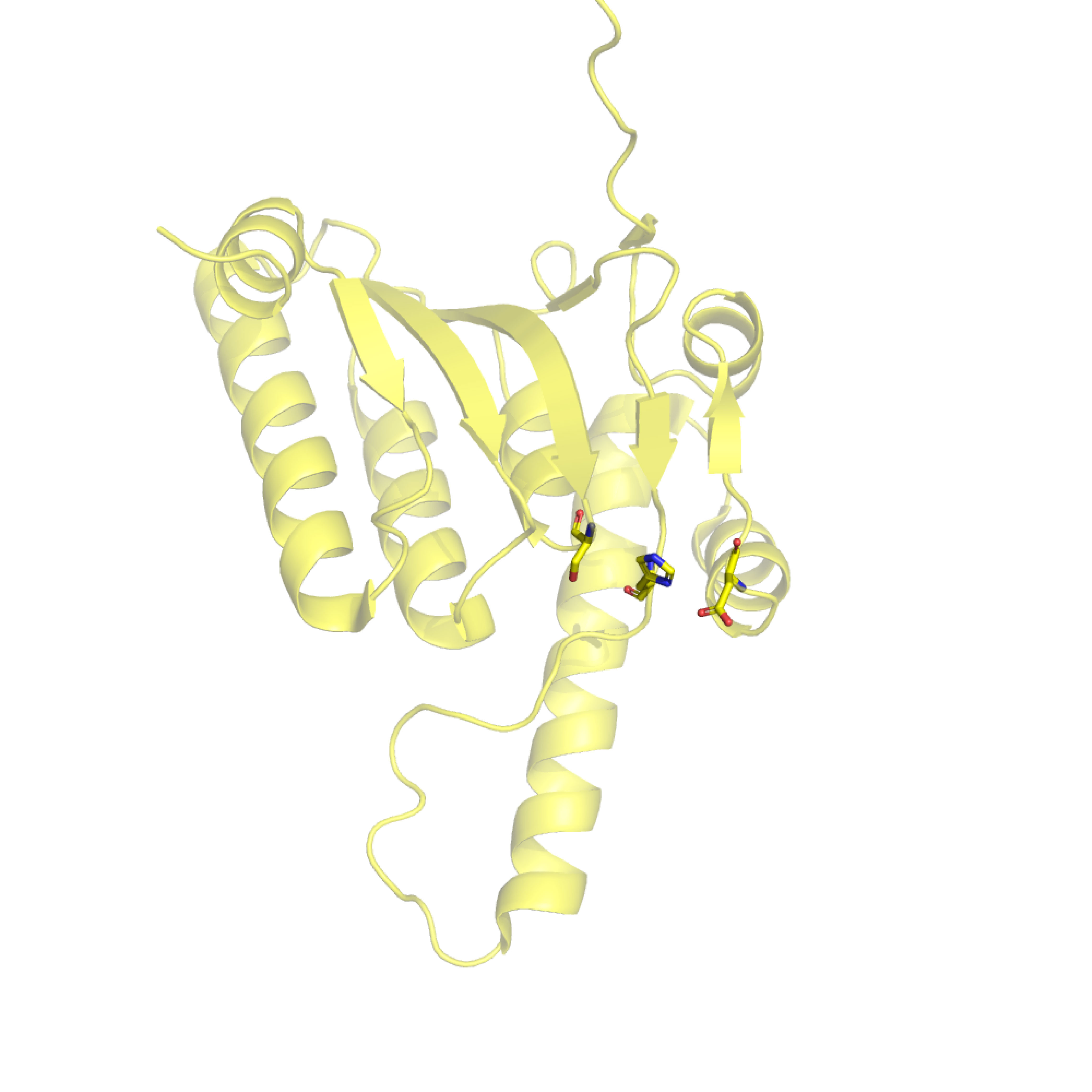

Supplement: S1 File — (ZIP) [file ppat.1013909.s010.zip › Fig 3/Fig 3C-g.jpg]

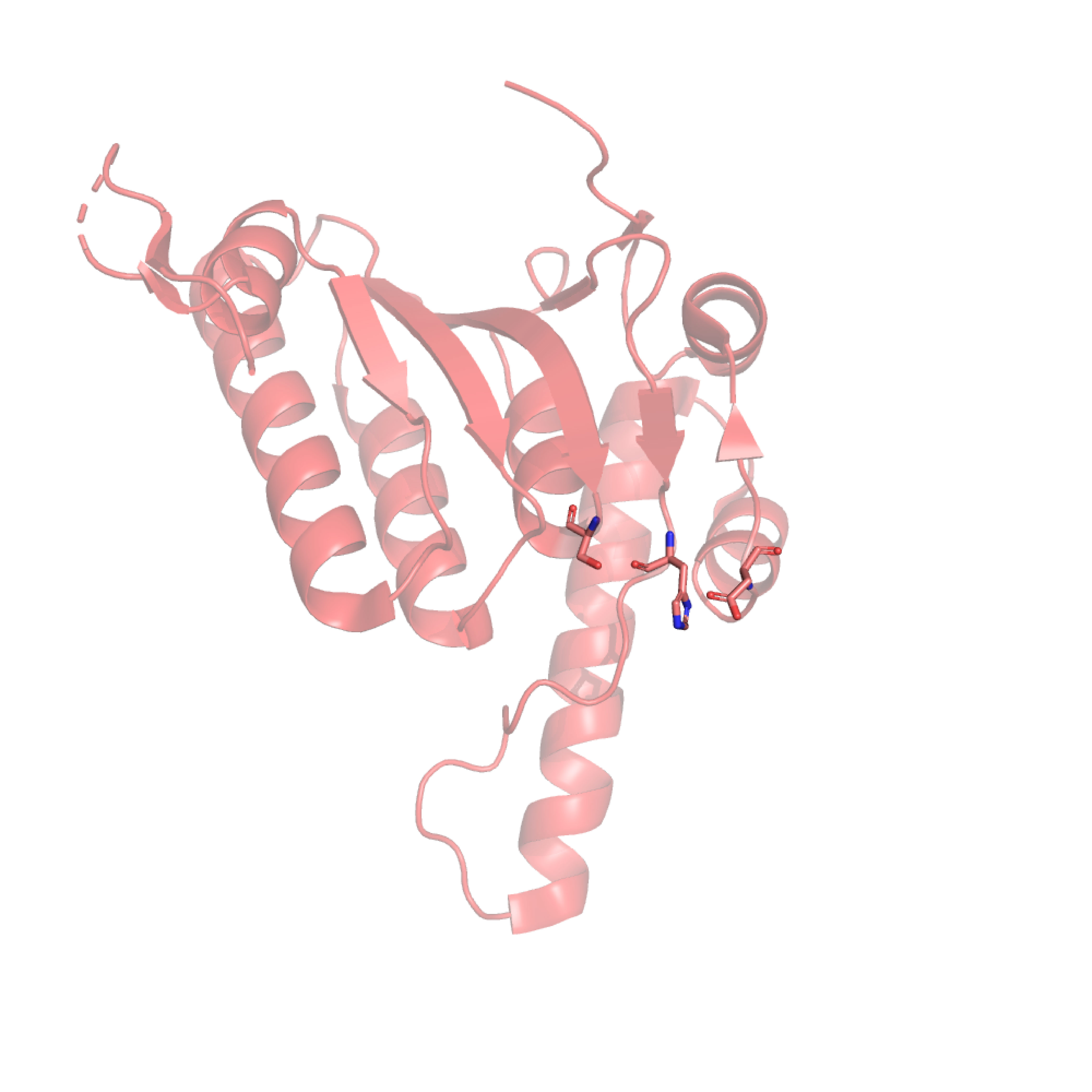

Supplement: S1 File — (ZIP) [file ppat.1013909.s010.zip › Fig 3/Fig 3C-h.jpg]

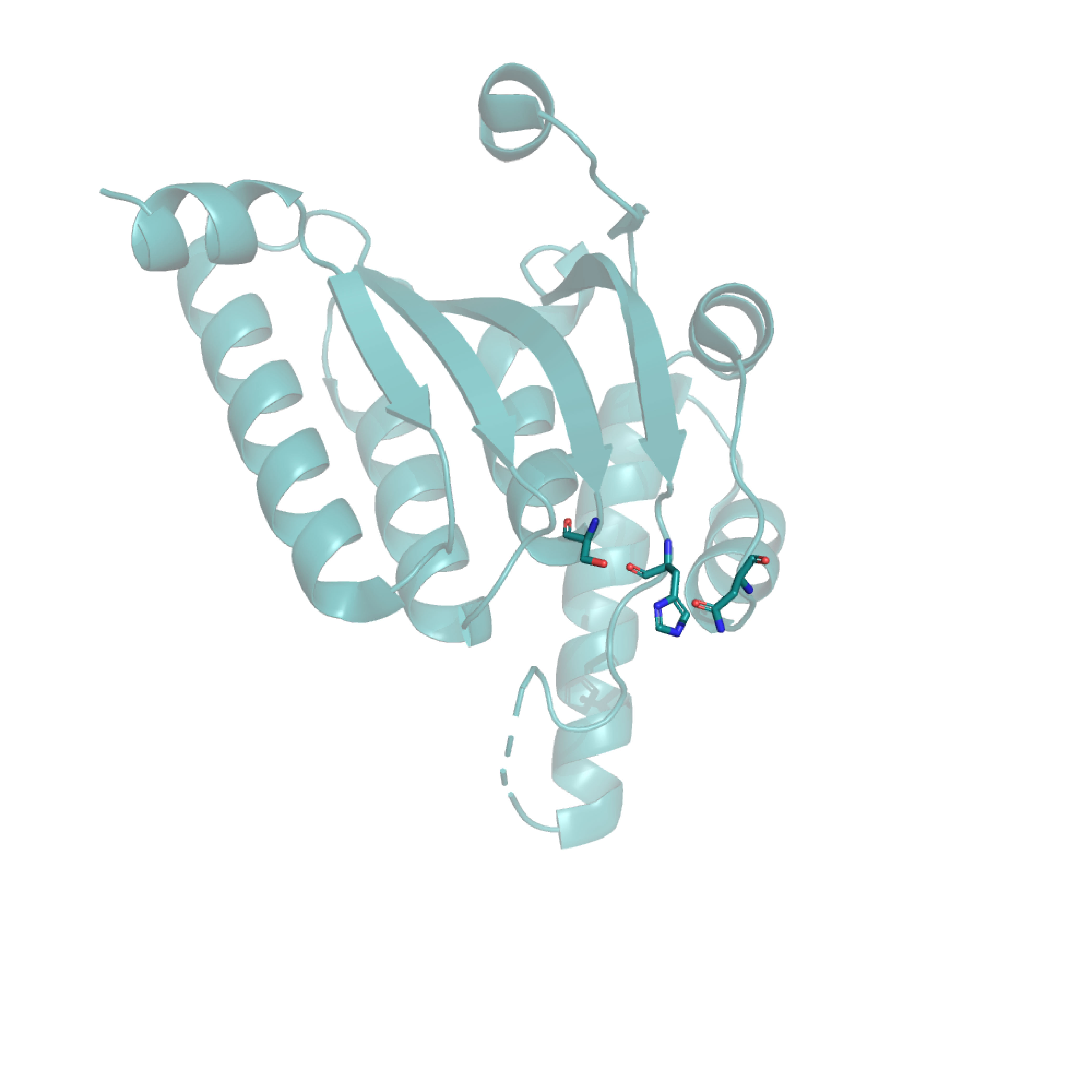

Supplement: S1 File — (ZIP) [file ppat.1013909.s010.zip › Fig 3/Fig 3C-i.jpg]

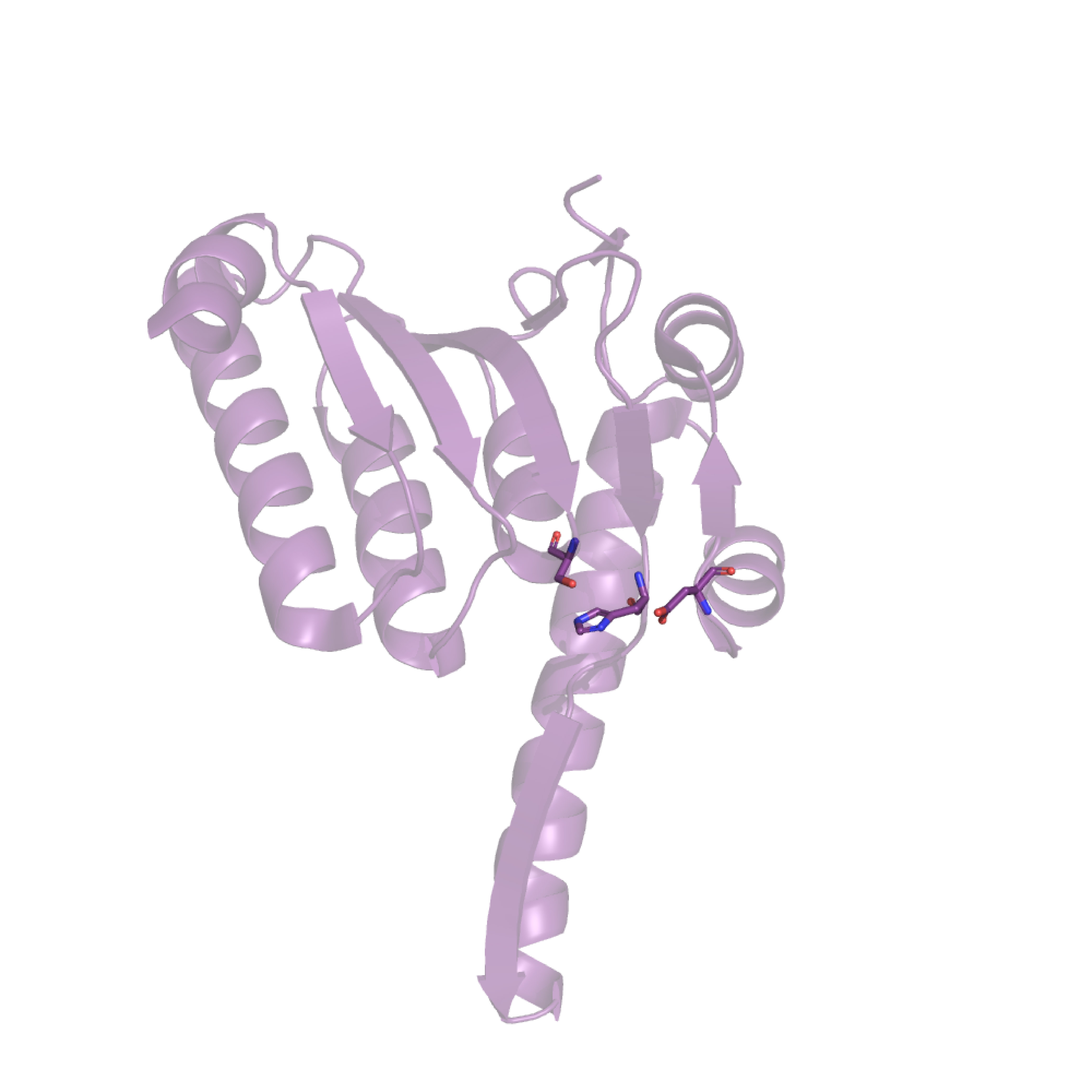

Supplement: S1 File — (ZIP) [file ppat.1013909.s010.zip › Fig 3/Fig 3C-j.jpg]

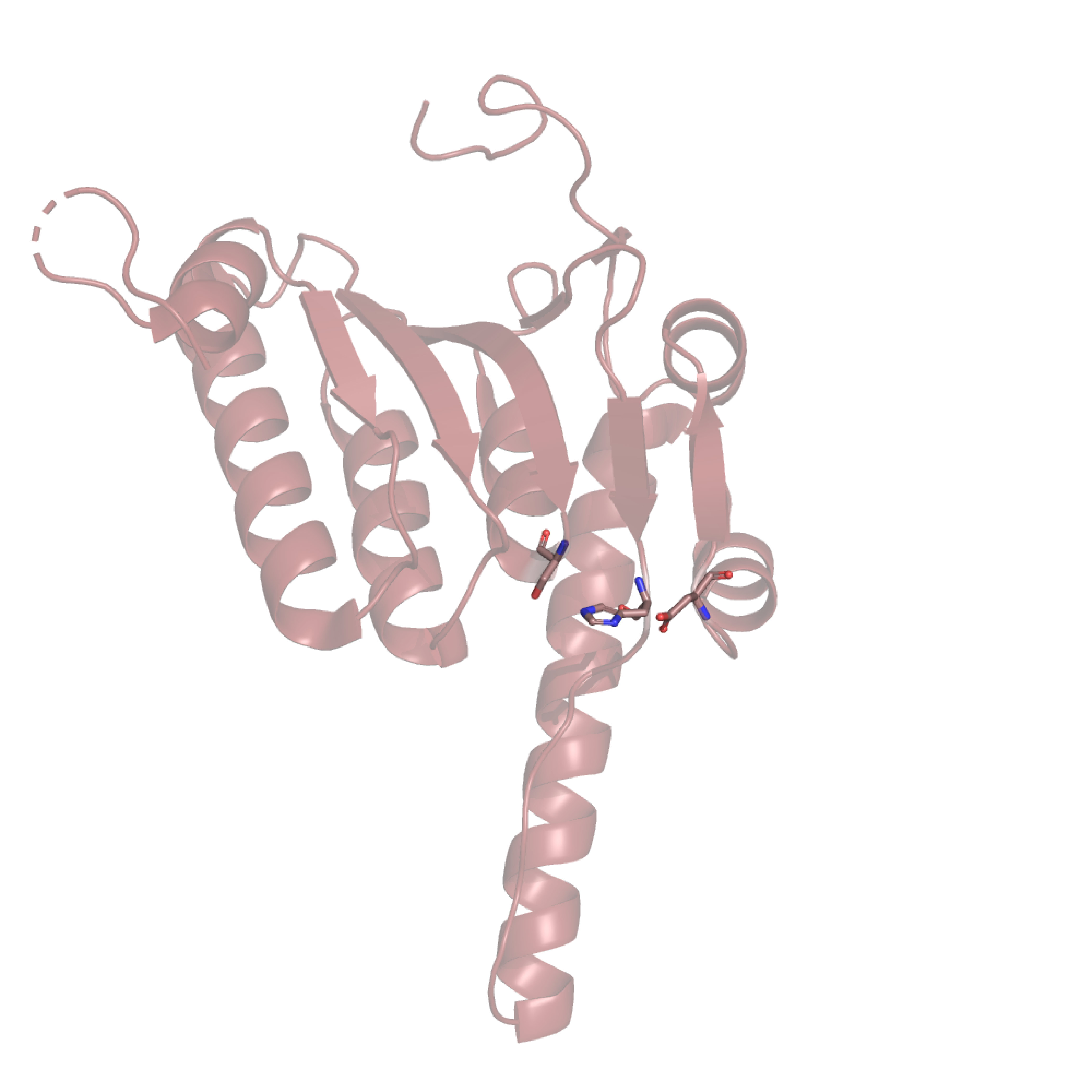

Supplement: S1 File — (ZIP) [file ppat.1013909.s010.zip › Fig 3/Fig 3C-k.jpg]

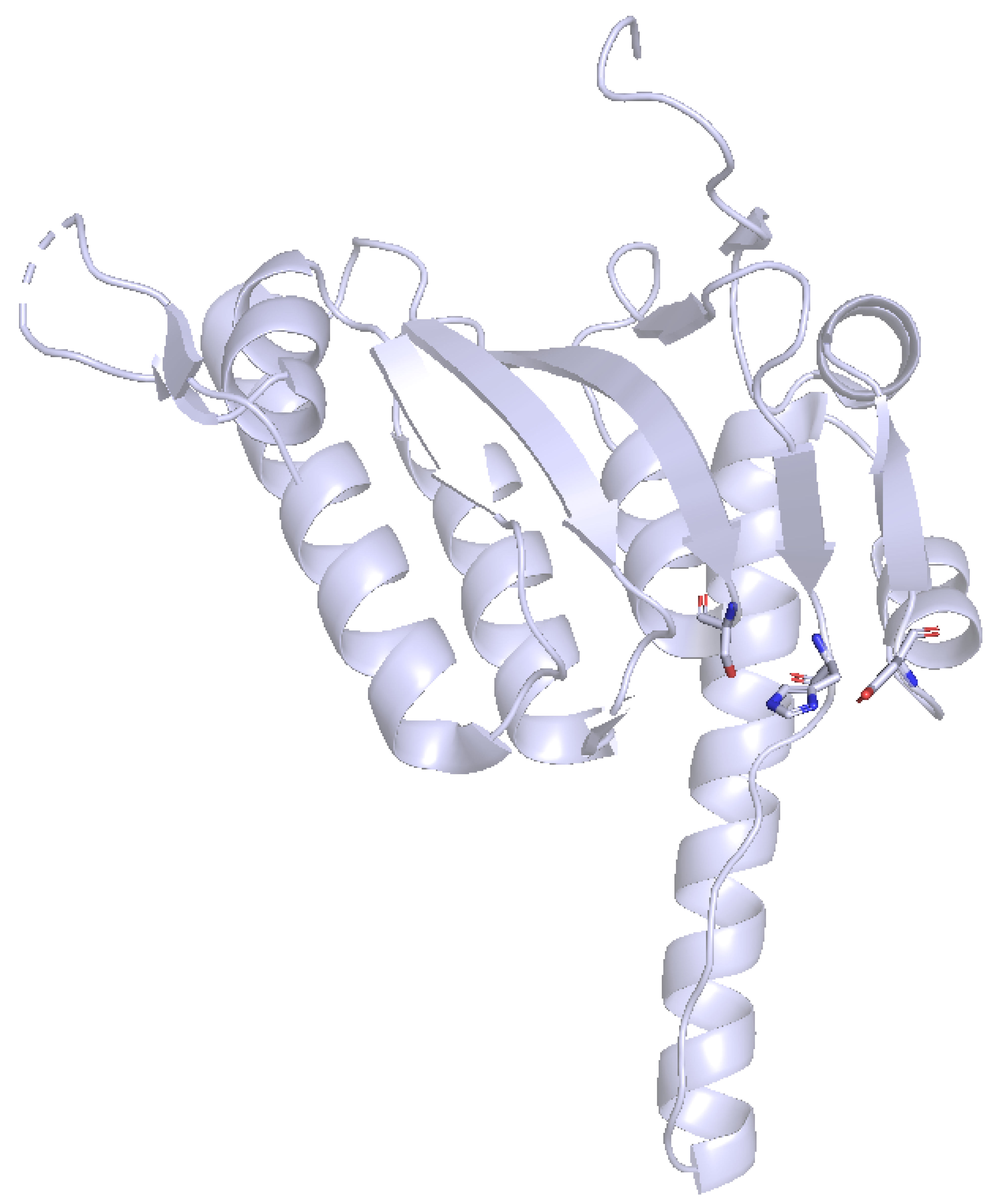

Supplement: S1 File — (ZIP) [file ppat.1013909.s010.zip › Fig 3/Fig 3C-l.jpg]

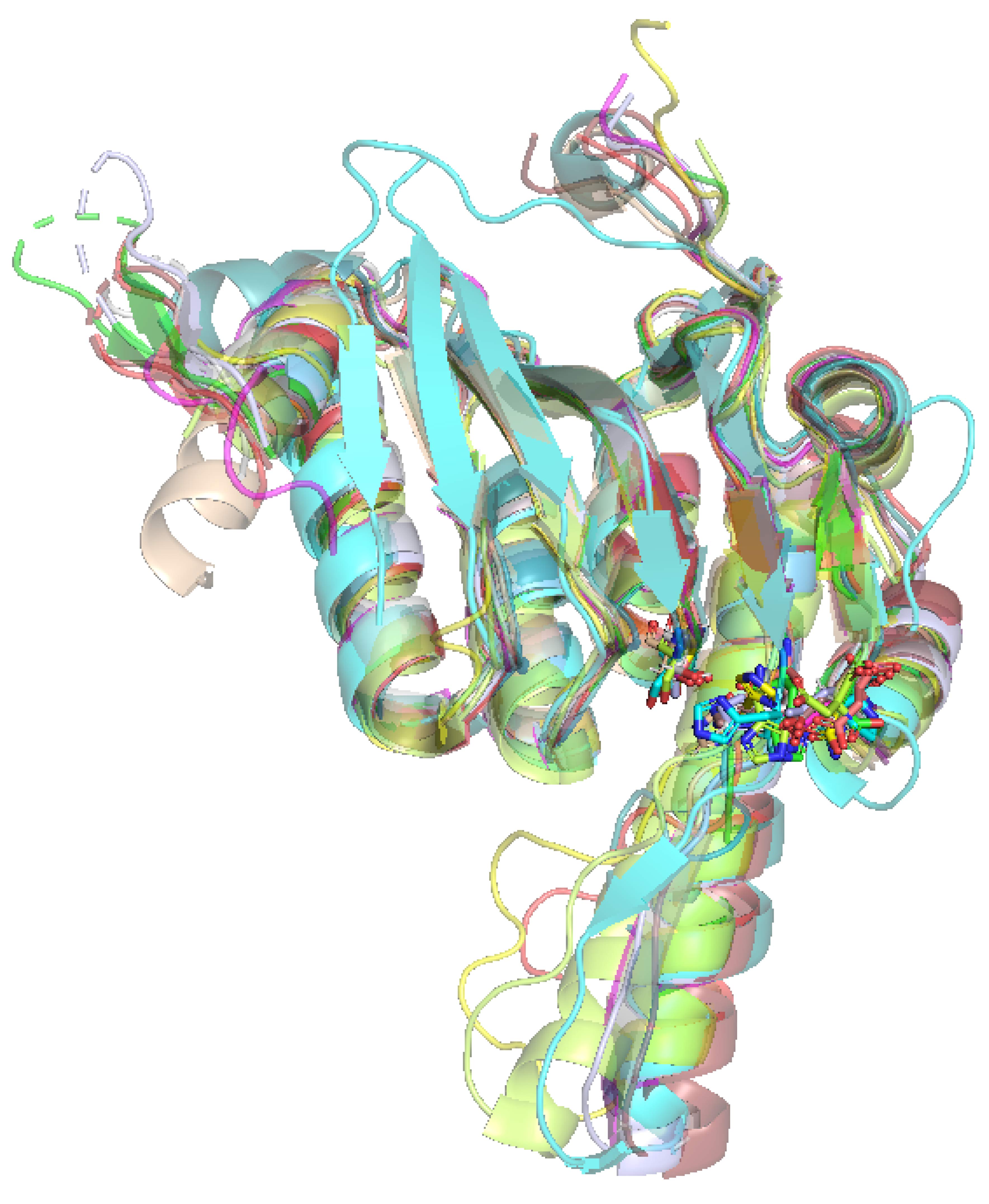

Supplement: S1 File — (ZIP) [file ppat.1013909.s010.zip › Fig 3/Fig 3C-m.jpg]

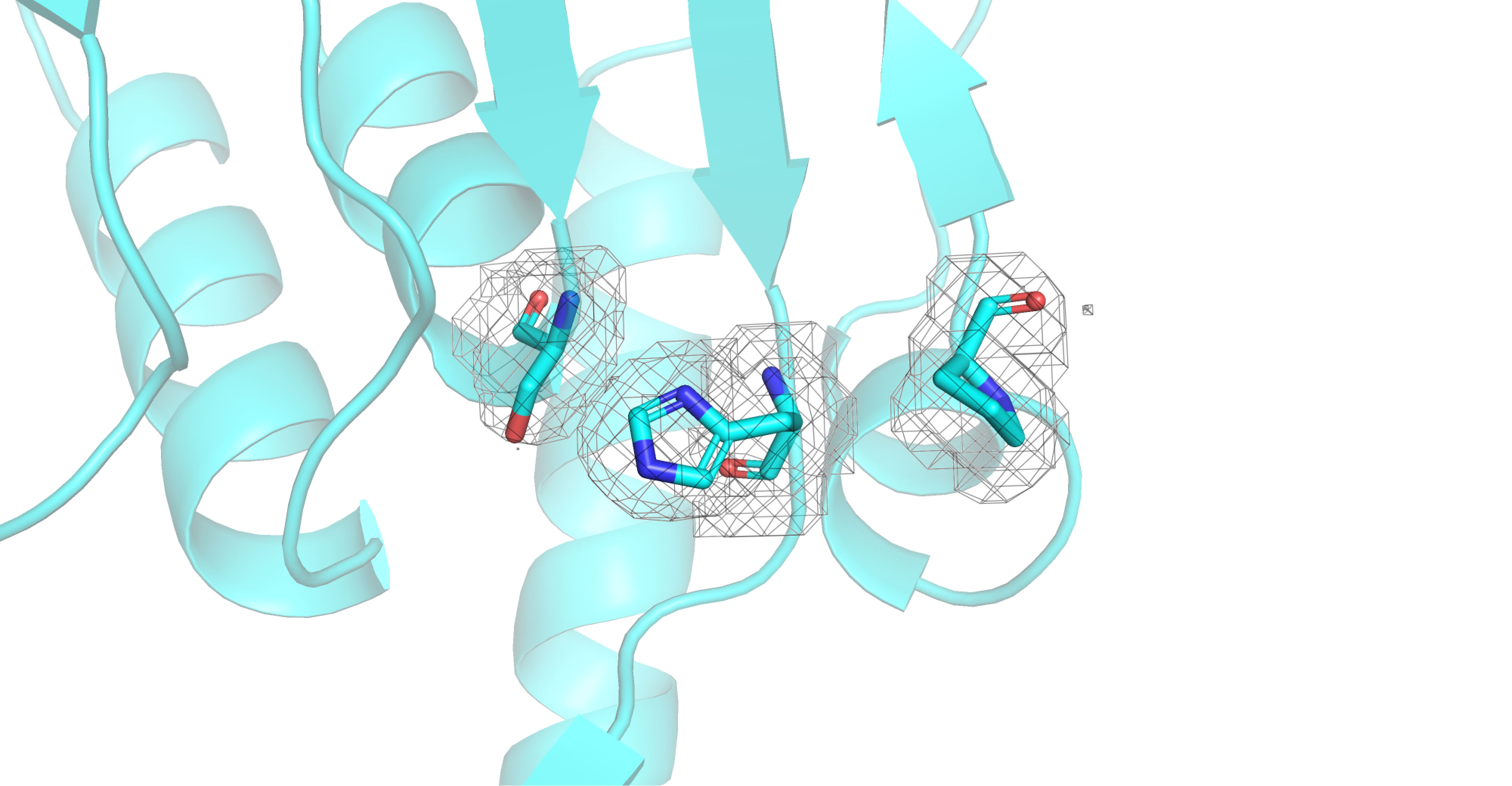

Supplement: S1 File — (ZIP) [file ppat.1013909.s010.zip › Fig 3/Fig 3C-n.jpg]

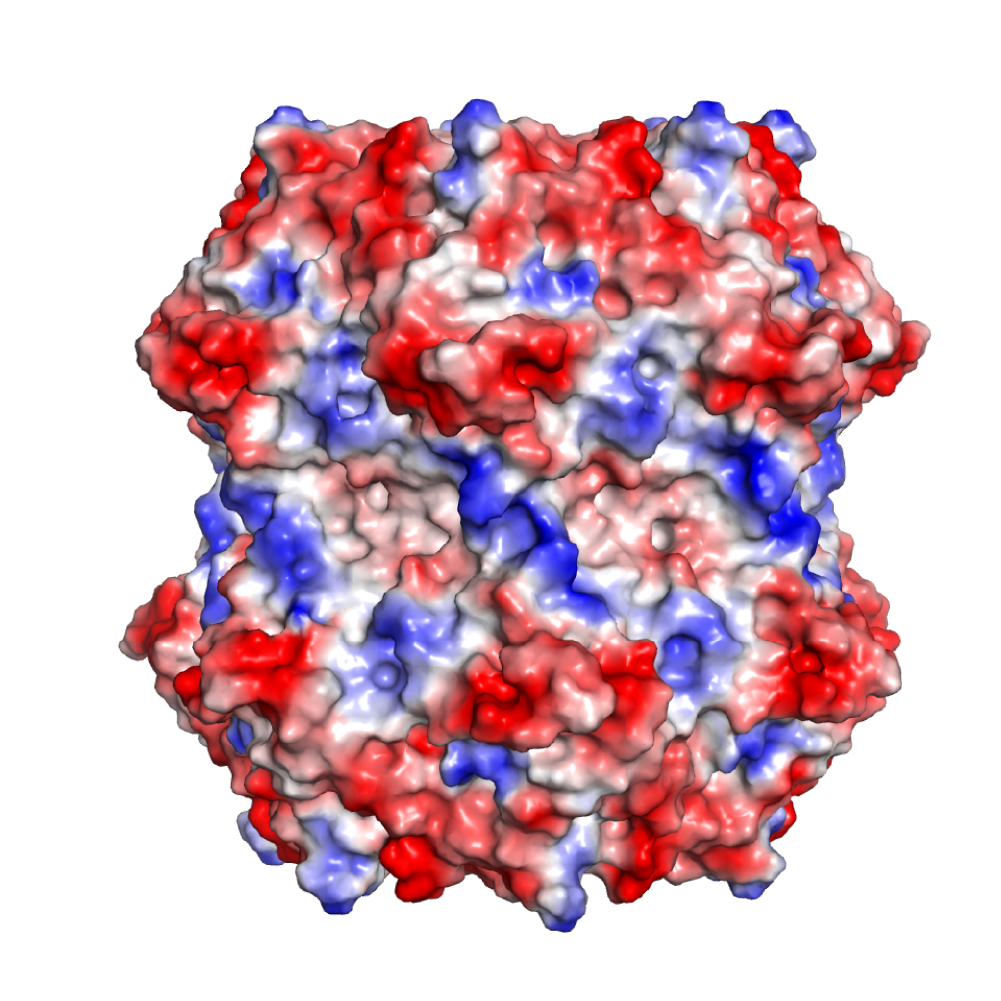

Supplement: S1 File — (ZIP) [file ppat.1013909.s010.zip › Fig 4/Fig 4A-1.jpg]

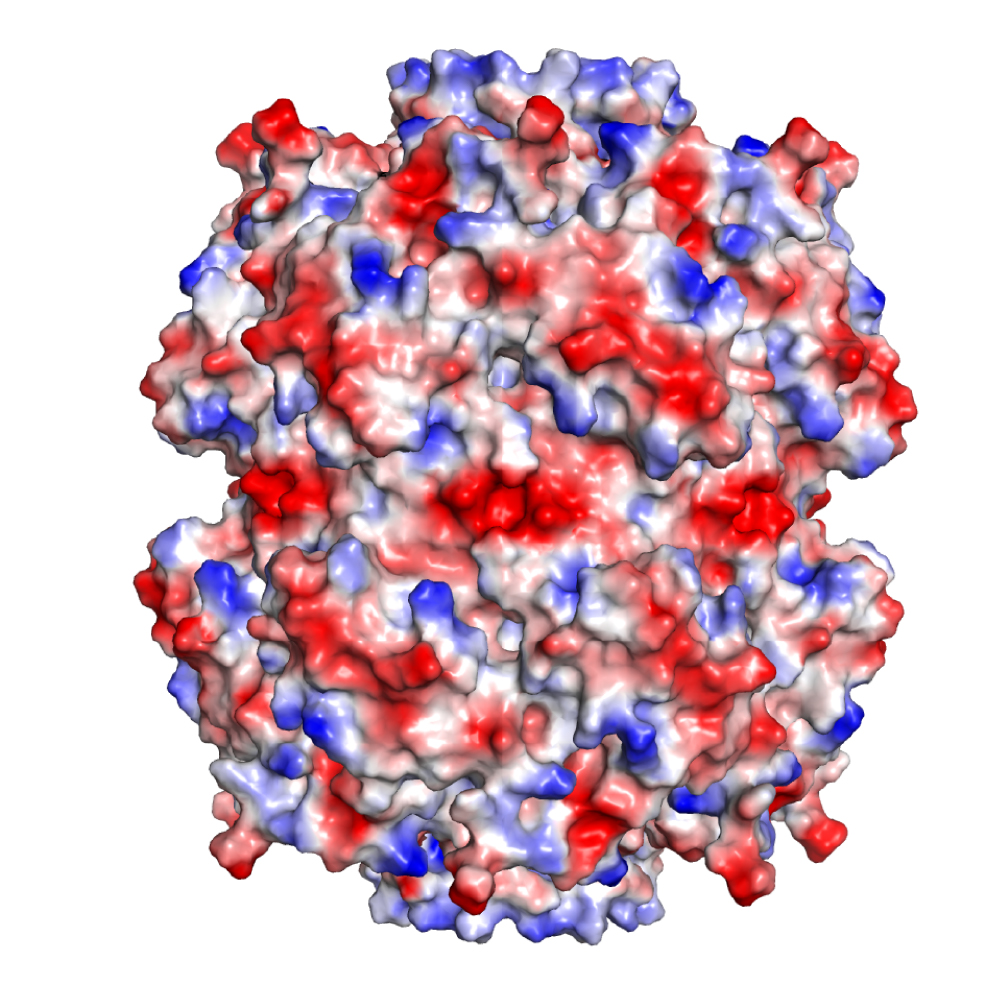

Supplement: S1 File — (ZIP) [file ppat.1013909.s010.zip › Fig 4/Fig 4A-2.jpg]

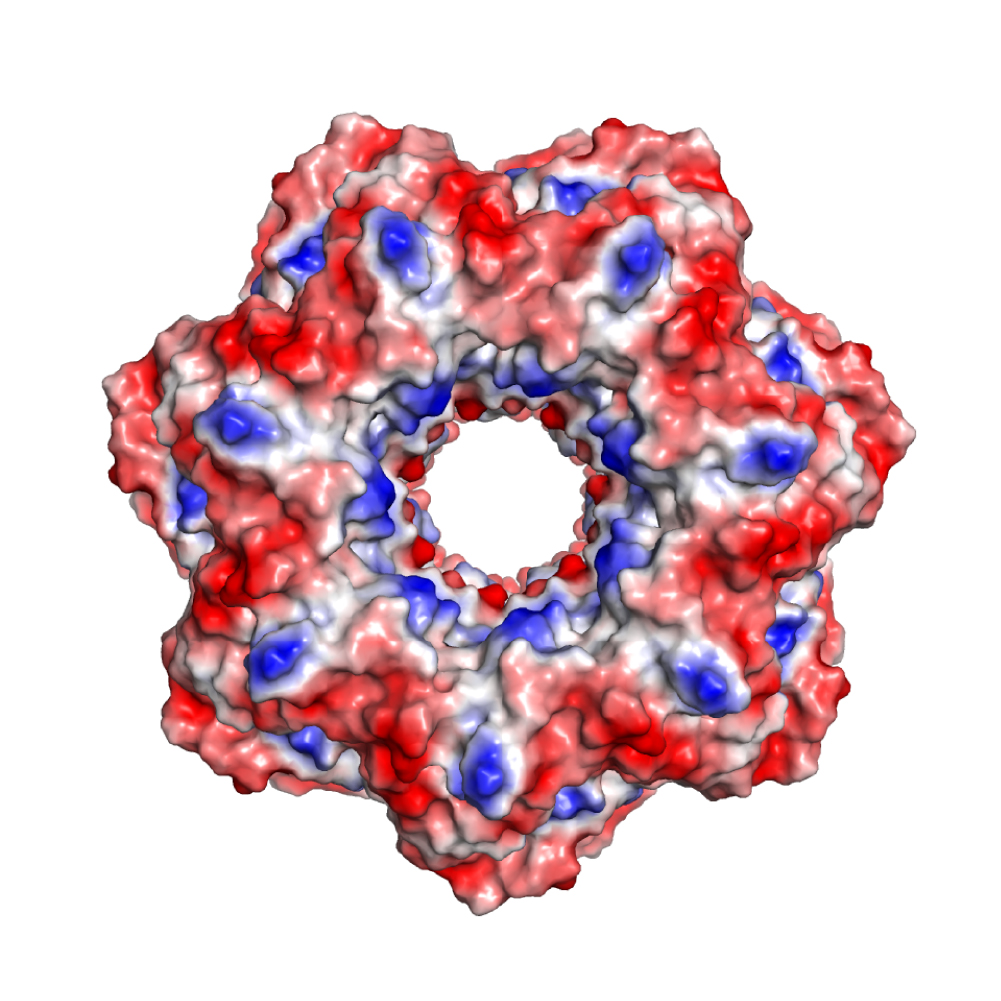

Supplement: S1 File — (ZIP) [file ppat.1013909.s010.zip › Fig 4/Fig 4B-1.jpg]

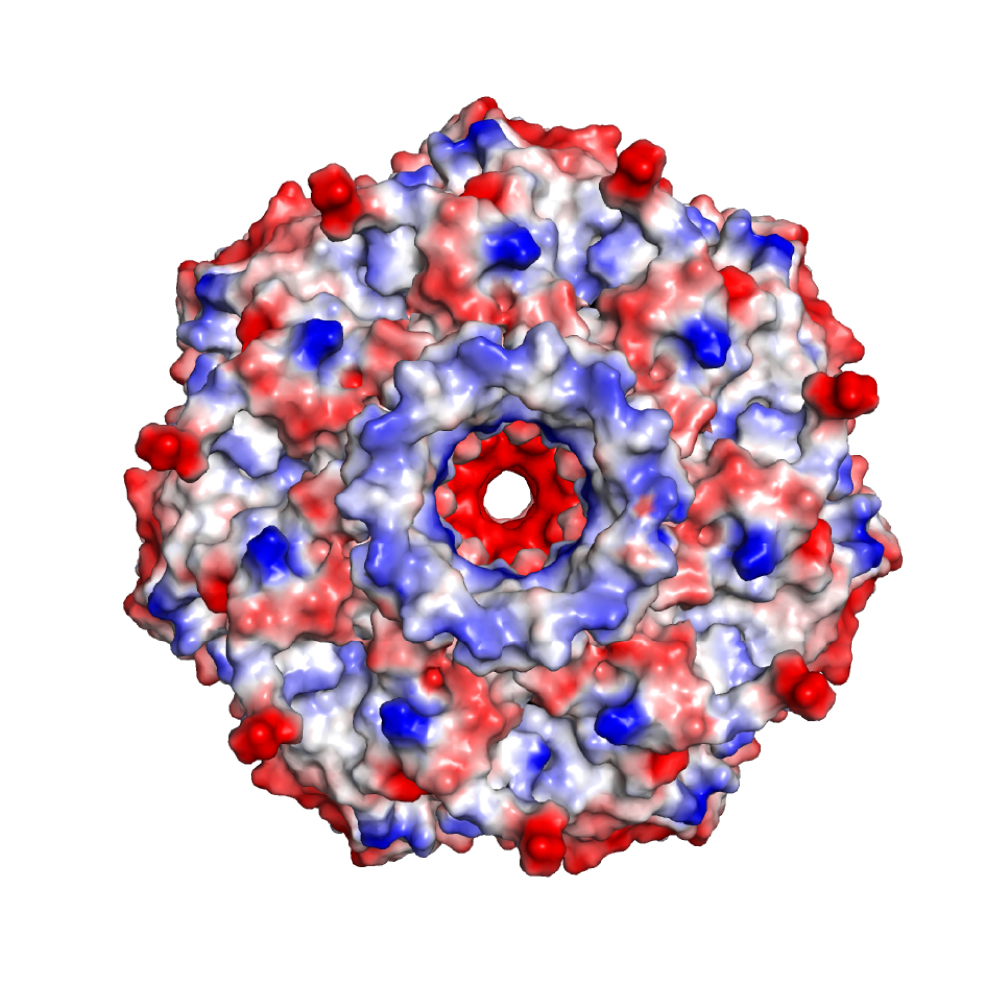

Supplement: S1 File — (ZIP) [file ppat.1013909.s010.zip › Fig 4/Fig 4B-2.jpg]

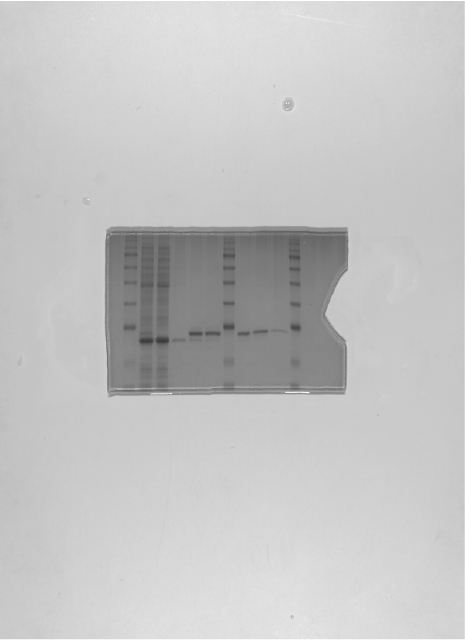

Supplement: S1 File — (ZIP) [file ppat.1013909.s010.zip › Fig 4/Fig 4D-a.tif]

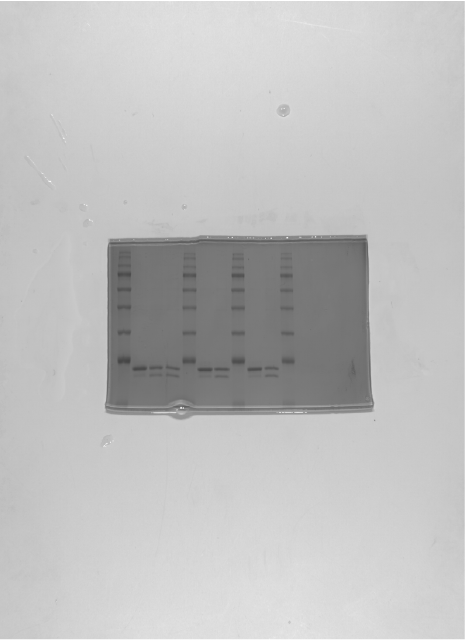

Supplement: S1 File — (ZIP) [file ppat.1013909.s010.zip › Fig 4/Fig 4D-b.tif]

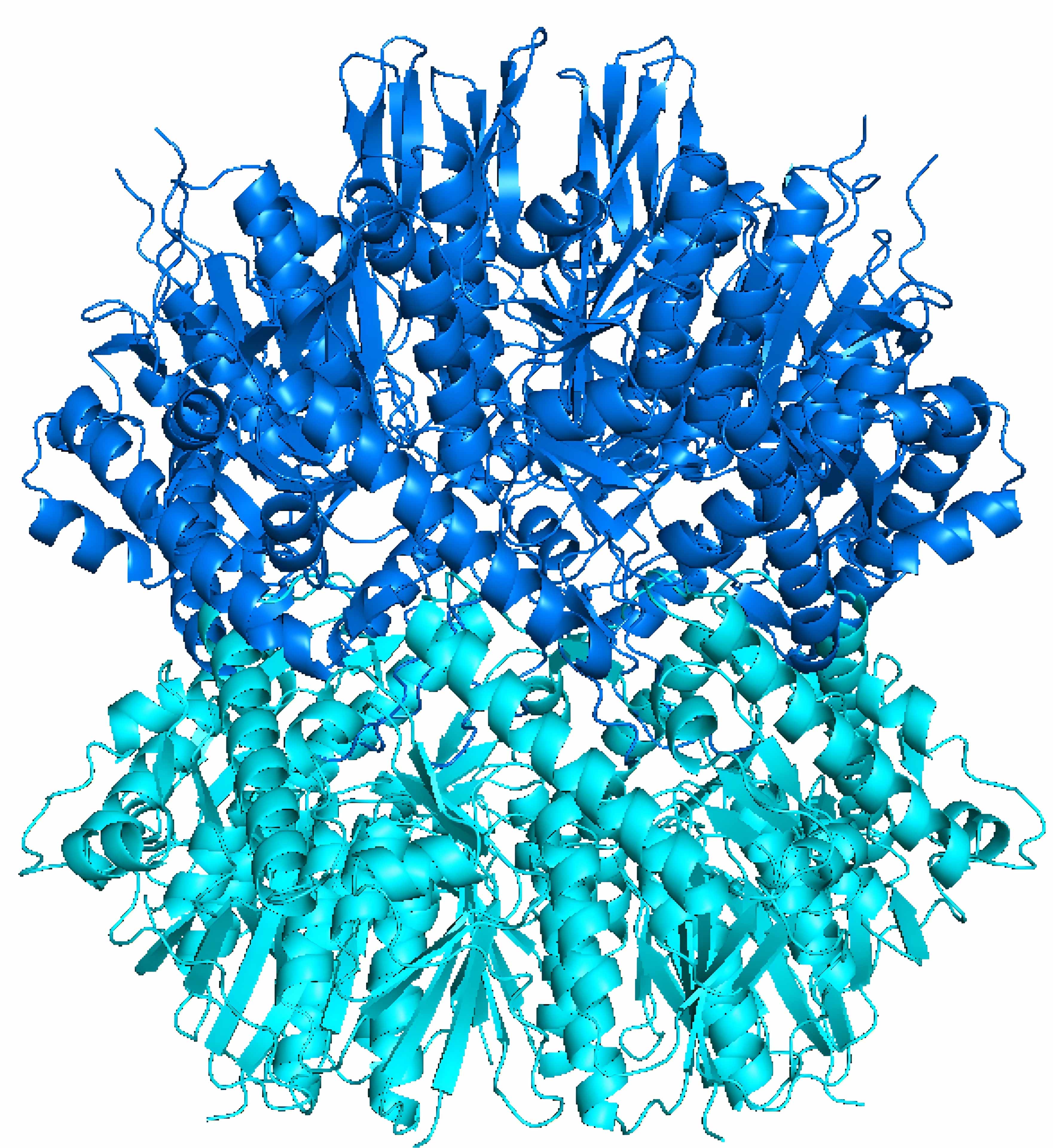

Supplement: S1 File — (ZIP) [file ppat.1013909.s010.zip › Fig 4/Fig 4F.jpg]

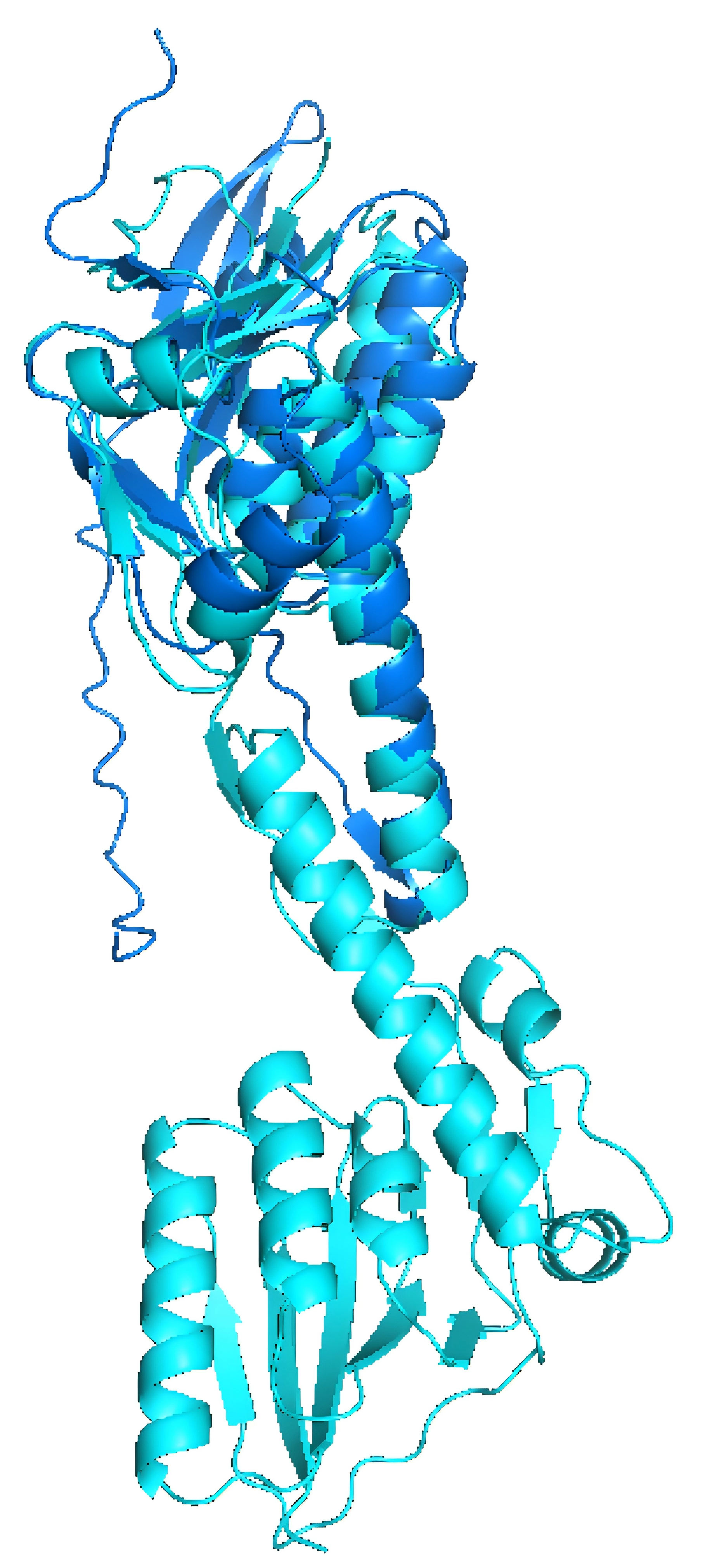

Supplement: S1 File — (ZIP) [file ppat.1013909.s010.zip › Fig 4/Fig 4G.jpg]

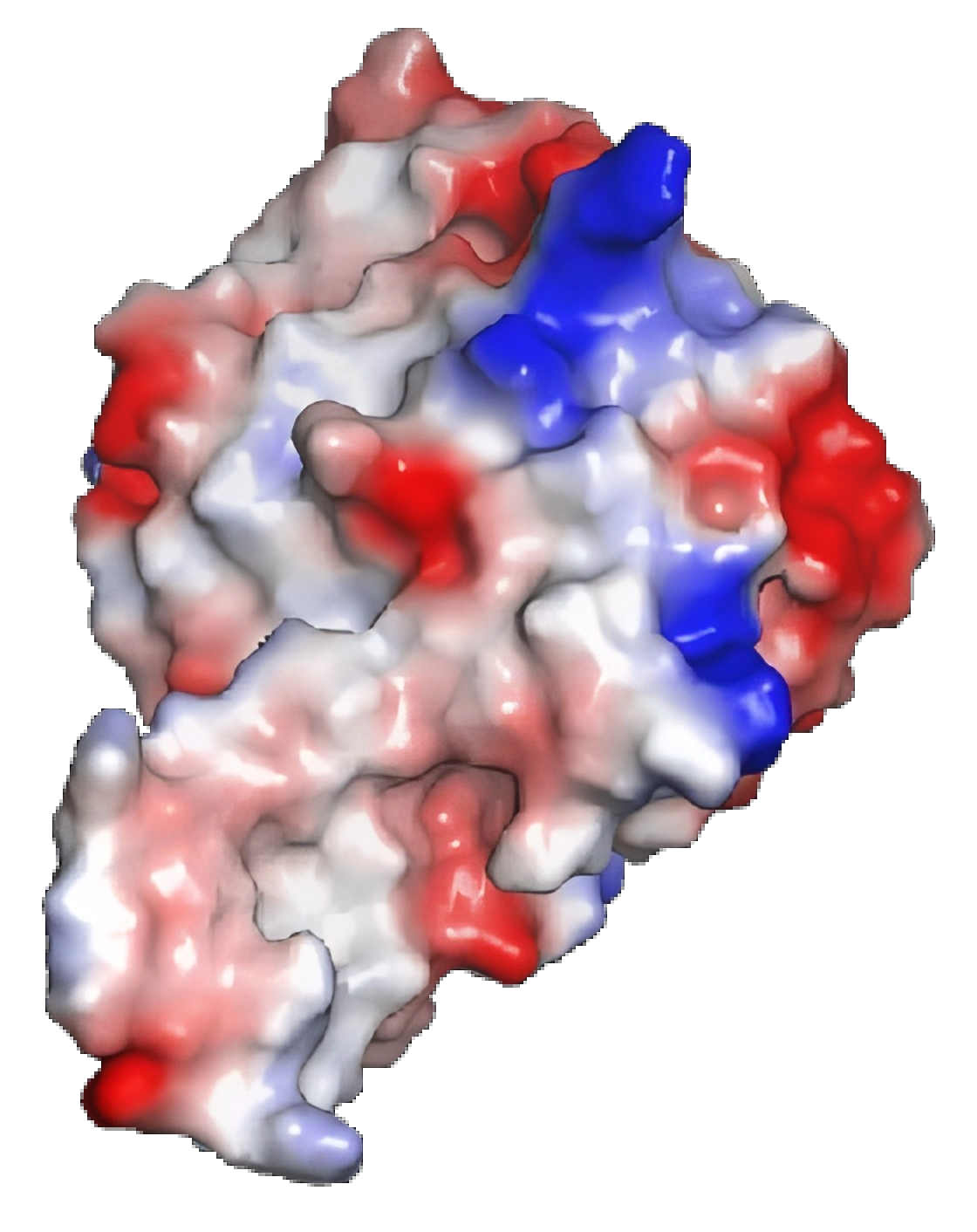

Supplement: S1 File — (ZIP) [file ppat.1013909.s010.zip › Fig 4/Fig 4H-1.jpg]

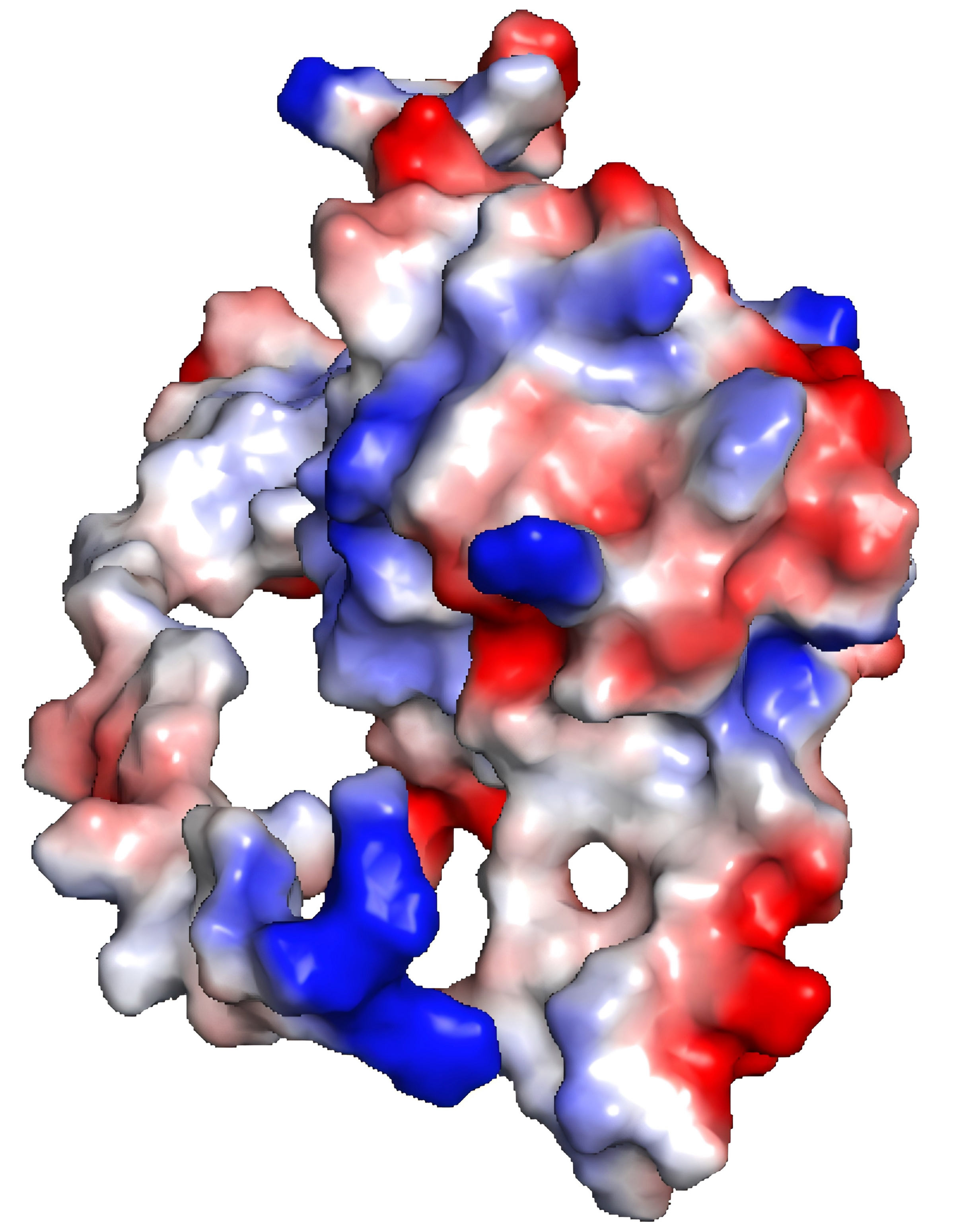

Supplement: S1 File — (ZIP) [file ppat.1013909.s010.zip › Fig 4/Fig 4H-2.jpg]

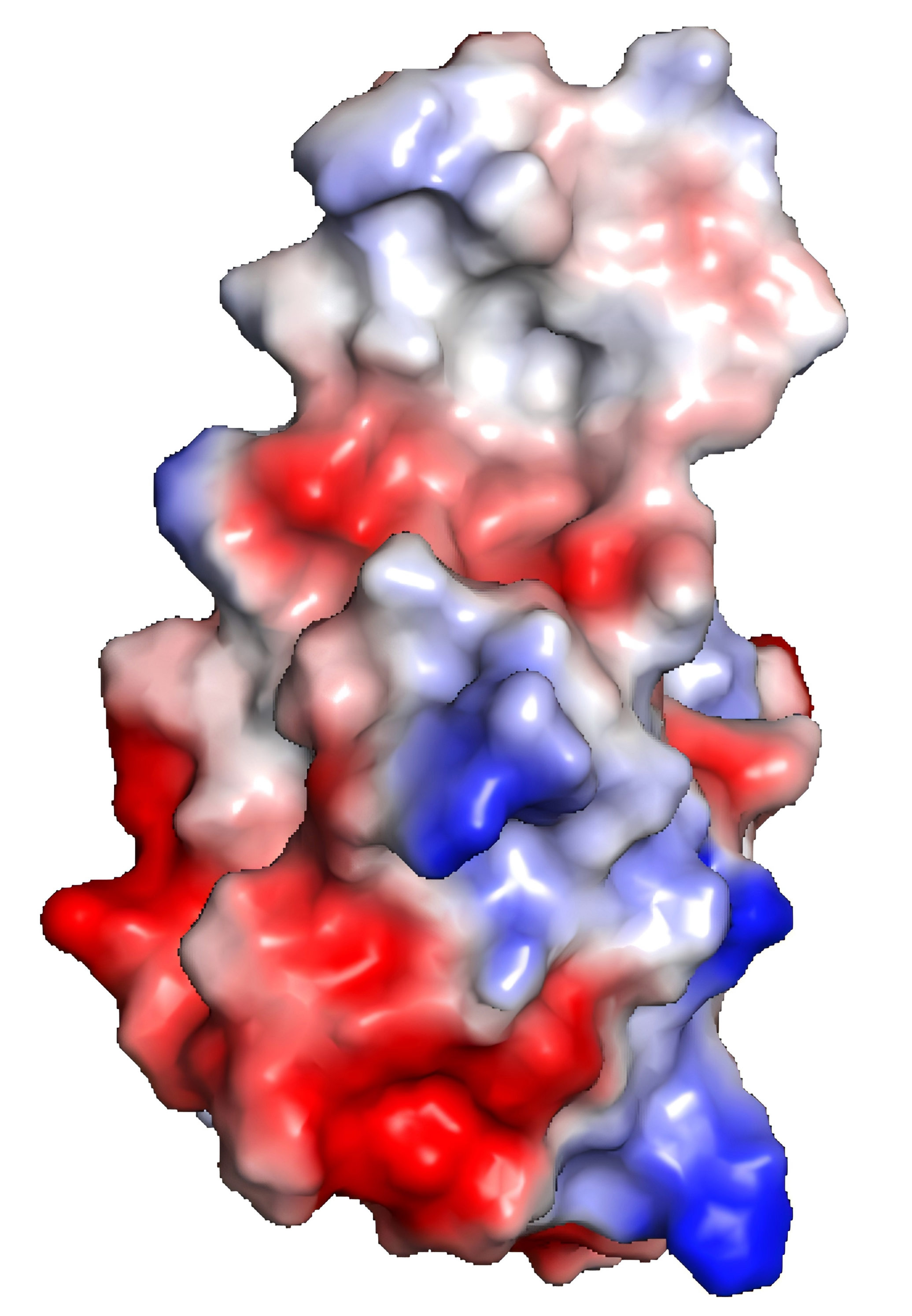

Supplement: S1 File — (ZIP) [file ppat.1013909.s010.zip › Fig 4/Fig 4H-3.jpg]
