# Supplementary material for: Structural and mechanistic insights into caseinolytic protease inhibition for antimicrobial development against Pseudomonas plecoglossicida
Source: PLoS Pathog. 2026 Feb 12;22(2):e1013909. doi: 10.1371/journal.ppat.1013909 (PMC12900304; doi:10.1371/journal.ppat.1013909)
Supplement: S1 File — (ZIP) [file S1A Fig.pdf › S1 Fig/S1A Fig.pdf]

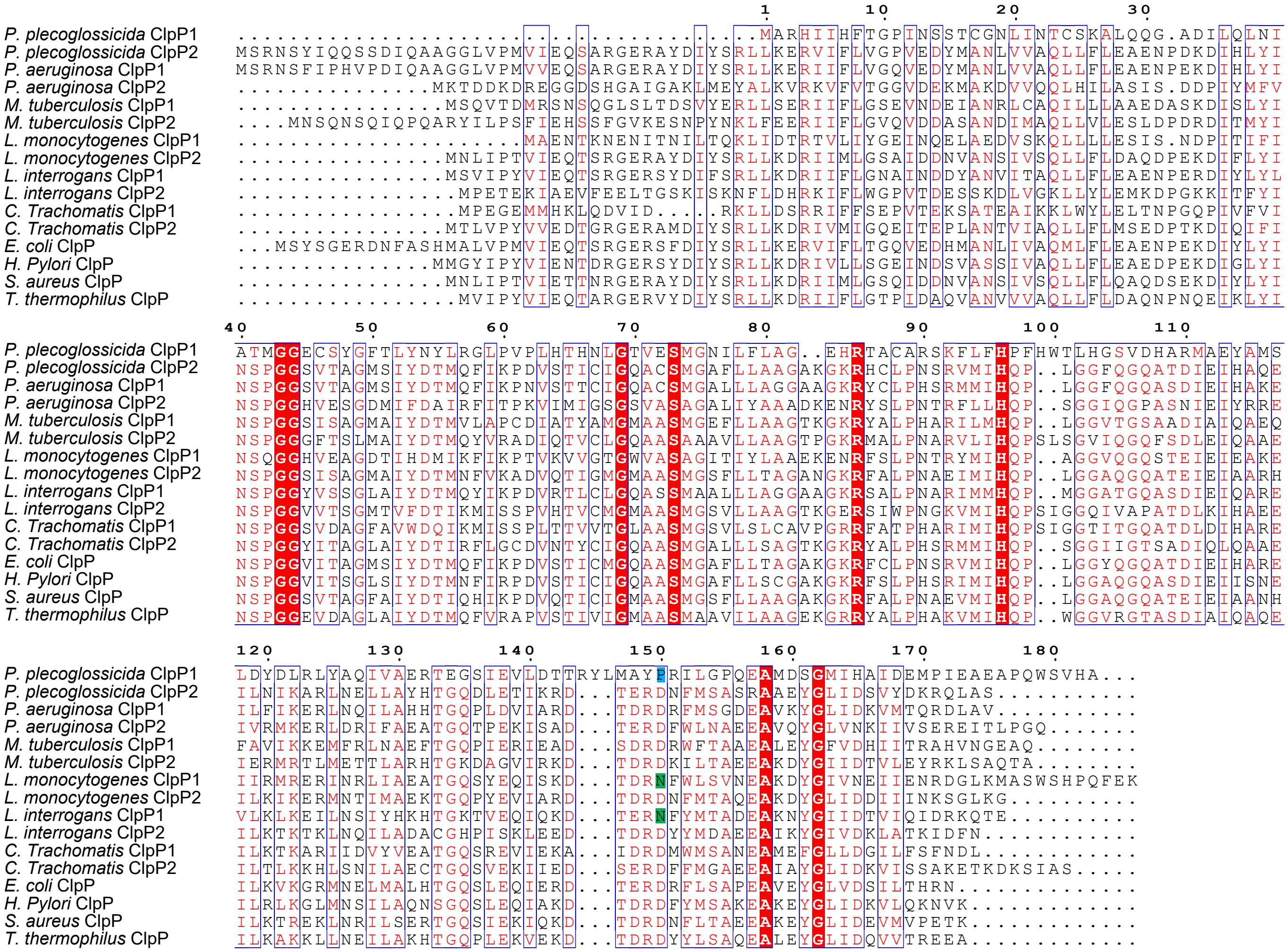
P. plecoglossicida ClpP1
P. plecoglossicida ClpP2
P. aeruginosa ClpP1
P. aeruginosa ClpP2
M. tuberculosis ClpP1
M. tuberculosis ClpP2
L. monocytogenes ClpP1
L. monocytogenes ClpP2
L. interrogans ClpP1
L. interrogans ClpP2
C. Trachomatis ClpP1
C. Trachomatis ClpP2
E. coli ClpP
H. Pylori ClpP
S. aureus ClpP
T. thermophilus ClpP
